# Efficacy and safety of intermittent preventive treatment and intermittent screening and treatment versus single screening and treatment with dihydroartemisinin–piperaquine for the control of malaria in pregnancy in Indonesia: a cluster-randomised, open-label, superiority trial

**DOI:** 10.1016/S1473-3099(19)30156-2

**Published:** 2019-09

**Authors:** Rukhsana Ahmed, Jeanne R Poespoprodjo, Din Syafruddin, Carole Khairallah, Cheryl Pace, Theda Lukito, Sylvia S Maratina, Puji B S Asih, Maria A Santana-Morales, Emily R Adams, Vera T Unwin, Christopher T Williams, Tao Chen, James Smedley, Duolao Wang, Brian Faragher, Richard N Price, Feiko O ter Kuile

**Affiliations:** a Department of Clinical Sciences, Liverpool School of Tropical Medicine, Liverpool, UK; b Malaria and Vector Resistance Laboratory, Eijkman Institute for Molecular Biology, Jakarta, Indonesia; c Mimika District Health Authority, Timika, Papua, Indonesia; d Timika Malaria Research Programme, Papuan Health and Community Development Foundation, Timika, Papua, Indonesia; e Department of Child Health, Faculty of Medicine, Public Health and Nursing, Universitas Gadjah Mada, Yogyakarta, Indonesia; f Department of Obstetrics and Gynecology, Pediatrics, Preventive Medicine and Public Health, Toxicology, Legal and Forensic Medicine and Parasitology, University Institute of Tropical Diseases and Public Health of the Canary Islands, University of la Laguna, Tenerife, Spain; g Network Biomedical Research on Tropical Diseases, RICET, Madrid, Spain; h Centre for Drugs and Diagnostics Research, Department of Tropical Disease Biology, Liverpool School of Tropical Medicine, Liverpool, UK; i Global and Tropical Health Division, Menzies School of Health Research and Charles Darwin University, Darwin, NT, Australia; j Centre for Tropical Medicine and Global Health, Nuffield Department of Medicine, University of Oxford, Oxford, UK; k Mahidol-Oxford Tropical Medicine Research Unit, Faculty of Tropical Medicine, Mahidol University, Bangkok, Thailand

## Abstract

**Background:**

*Plasmodium falciparum* and *Plasmodium vivax* infections are important causes of adverse pregnancy outcomes in the Asia-Pacific region. We hypothesised that monthly intermittent preventive treatment (IPT) or intermittent screening and treatment (IST) with dihydroartemisinin–piperaquine is more effective in reducing malaria in pregnancy than the existing single screening and treatment (SST) strategy, which is used to screen women for malaria infections at the first antenatal visit followed by passive case detection, with management of febrile cases.

**Methods:**

We did an open-label, three-arm, cluster-randomised, superiority trial in Sumba (low malaria transmission site) and Papua (moderate malaria *t*ransmission site), Indonesia. Eligible participants were 16–30 weeks pregnant. Clusters (antenatal clinics with at least ten new pregnancies per year matched by location, size, and malaria risk) were randomly assigned (1:1:1) via computer-generated lists to IPT, IST, or SST clusters. In IPT clusters, participants received the fixed-dose combination of dihydroartemisinin-piperaquine (4 and 18 mg/kg per day). In IST clusters, participants were screened with malaria rapid diagnostic tests once a month, whereas, in SST clusters, they were screened at enrolment only. In all groups, participants with fever were tested for malaria. Any participant who tested positive received dihydroartemisinin–piperaquine regardless of symptoms. The primary outcome was malaria infection in the mother at delivery. Laboratory staff were unaware of group allocation. Analyses included all randomly assigned participants contributing outcome data and were adjusted for clustering at the clinic level. This trial is complete and is registered with ISRCTN, number 34010937.

**Findings:**

Between May 16, 2013, and April 21, 2016, 78 clusters (57 in Sumba and 21 in Papua) were randomly assigned to SST, IPT, or IST clusters (26 clusters each). Of 3553 women screened for eligibility, 2279 were enrolled (744 in SST clusters, 681 in IPT clusters, and 854 in IST clusters). At enrolment, malaria prevalence was lower in IST (5·7%) than in SST (12·6%) and IPT (10·6%) clusters. At delivery, malaria prevalence was 20·2% (128 of 633) in SST clusters, compared with 11·6% (61 of 528) in IPT clusters (relative risk [RR] 0·59, 95% CI 0·42–0·83, p=0·0022) and 11·8% (84 of 713) in IST clusters (0·56, 0·40–0·77, p=0·0005). Conditions related to the pregnancy, the puerperium, and the perinatal period were the most common serious adverse events for the mothers, and infections and infestations for the infants. There were no differences between groups in serious adverse events in the mothers or in their infants.

**Interpretation:**

IST was associated with a lower prevalence of malaria than SST at delivery, but the prevalence of malaria in this group was also lower at enrolment, making interpretation of the effect of IST challenging. Further studies with highly sensitive malaria rapid diagnostic tests should be considered. Monthly IPT with dihydroartemisinin–piperaquine is a promising alternative to SST in areas in the Asia-Pacific region with moderate or high transmission of malaria.

**Funding:**

Joint Global Health Trials Scheme of the Medical Research Council, Department for International-Development, and the Wellcome Trust.

## Introduction

Approximately 70% of 125·2 million pregnancies in malaria-endemic areas occur in the Asia-Pacific region annually,^1^ where antenatal infections with *Plasmodium falciparum* and *Plasmodium vivax* are associated with adverse pregnancy outcomes.^2^, ^3^, ^4^ In the African region, a prevention strategy has been endorsed by WHO, including provision of a long-lasting insecticidal net (LLIN) and intermittent preventive treatment (IPT) in pregnancy, consisting of curative doses of sulfadoxine–pyrimethamine given at every scheduled antenatal visit in the second and third trimesters. However, in the Asia-Pacific region, few countries have chemoprevention strategies for malaria in pregnancy.^4^ Most provide LLINs as part of antenatal care and use single screening and treatment (SST) strategies in pregnancy consisting of screening participants for malaria infections at the first antenatal visit followed by passive case detection, with management of febrile cases.^5^, ^6^ The paucity of chemoprevention strategies reflects the dearth of prevention trials and widespread parasite resistance in Asia to sulfadoxine–pyrimethamine,^7^ the only antimalarial recommended by WHO for IPT.^8^

**Research in context**

**Evidence before this study**

We searched the Malaria in Pregnancy Library and PubMed from their inception to Sept 20, 2018, without language restrictions, for relevant trials of chemoprevention with intermittent preventive treatment (IPT) or intermittent screening and treatment (IST) of malaria in pregnancy. We restricted the search to areas in the Asia-Pacific region, Central and South America, the horn of Africa, and Madagascar, where *Plasmodium falciparum* and *Plasmodium vivax* are co-endemic. The following search terms were used: “(intermittent OR IPT OR prophylaxis OR prevention) AND (malaria OR plasmodium) AND (pregnan* OR trimester OR gestation)”. The names of each country in these regions were added as additional search terms. Two IPT trials were identified, one in the Solomon Islands and the other in Papua New Guinea, of which both had used sulfadoxine–pyrimethamine. No IST trials were identified. Our literature search confirmed that few intermittent screening trials or chemoprevention trials in pregnancy have been done outside of Africa. WHO does not have a prevention strategy for malaria in pregnancy in the Asia-Pacific region, where about 70% of the global number of pregnancies in malaria-endemic areas occur. Most countries in this region use a single screening and treatment (SST) strategy for malaria at the first antenatal visit.

**Added value of this study**

To our knowledge, this study is the first prevention trial to compare monthly IPT or IST with the antimalarial dihydroartemisinin-piperaquine against the existing SST strategy with dihydroartemisinin–piperaquine for the control of malaria in pregnancy in the Asia-Pacific region. The study was designed to support the Indonesian Ministry of Health and WHO in the development of strategies for the control of malaria in pregnancy in Indonesia and the wider Asia-Pacific region.

**Implications of all the available evidence**

Our results do not support a role for IST with the existing standard malaria rapid diagnostic tests or for IPT with dihydroartemisinin–piperaquine in areas in the Asia-Pacific region with lower malaria transmission. However, our results confirm earlier findings from east Africa and show that IPT with dihydroartemisinin–piperaquine should be considered as a potential strategy to reduce the risk of malaria infection, and the associated adverse consequences in pregnancy in areas in the Asia-Pacific region with moderate to high levels of malaria transmission and high levels of resistance to sulfadoxine–pyrimethamine.

Three completed trials in areas of high sulfadoxine–pyrimethamine resistance in Kenya and Uganda^9^, ^10^, ^11^ suggest that the fixed-dose, artemisinin-based combination therapy of dihydroartemisinin–piperaquine is a promising candidate to replace sulfadoxine–pyrimethamine for use in IPT. IPT with dihydroartemisinin–piperaquine was associated with much greater reductions in malaria infection and clinical malaria during pregnancy than was IPT with sulfadoxine–pyrimethamine.^10^

In areas with predominantly low malaria transmission, alternative strategies involving regular screening and treatment approaches should also be considered. For example, in refugee camps on the Thai–Myanmar border, the introduction of weekly screening for malaria and treatment of pregnant women who tested positive reduced maternal mortality from malaria substantially.^12^ Such intensive screening programmes are unlikely to be feasible under programmatic conditions. However, four trials in sub-Saharan Africa showed good feasibility with less intensive intermittent screening and treatment (IST) strategies involving malaria rapid diagnostic tests done 3–6 times during pregnancy.^13^

Limitations of these screening strategies include the failure to detect *P falciparum* infections that are predominantly sequestered in the placenta, or low-grade infections that are below the limit of detection by standard microscopy or malaria rapid diagnostic tests, which are particularly common with *P vivax*. Furthermore, because of the parasite's dormant forms in the liver, a single *P vivax* infection might cause multiple relapses during pregnancy, when radical cure with primaquine is contraindicated. The SST strategy has the additional limitation of potentially missing re-infections or asymptomatic parasitaemia in later stages of pregnancy.

Here, we report the results of the first trial in the Asia-Pacific region designed to compare the safety and efficacy of monthly IST or IPT with dihydroartemisinin–piperaquine with the standard SST strategy for decreasing the risk of malaria infection in pregnancy.

## Methods

### Study design and participants

We did an open-label, two-site, three-arm, cluster-randomised, superiority-trial in areas in eastern Indonesia that are co-endemic for *P falciparum* and *P vivax*: Sumba Island,^14^, ^15^ which has low malaria transmission, and southern Papua, Indonesia,^16^, ^17^, ^18^ which has moderate year-round transmission (appendix p 4).^19^, ^20^

Antenatal clinics were eligible for inclusion if they had at least ten new pregnancies per year and were located within 1·5 h drive from the study offices (appendix pp 4–5).

Pregnant women of any gravity attending their first antenatal visit were eligible if they had a viable pregnancy between 16 and 30 weeks' gestation, were residents in the study catchment areas, were willing to complete the study schedule and deliver their baby at the study clinics or hospital, and had not yet been screened for malaria. Exclusion criteria comprised high-risk pregnancies due to pre-existing conditions likely to cause complication in the current pregnancy (e.g. hypertension, diabetes, asthma, renal disease, liver disease, any spinal deformity), severe malaria at presentation, treatment with antimalarials in the previous month, HIV positivity, a family history of sudden death or any known cardiac condition, current use of medication known to prolong the QTc interval, a history of allergy to dihydroartemisinin–piperaquine, and residence outside study area or plans to move within 6 months.

Ethical approval was obtained from the Liverpool School of Tropical Medicine, the Eijkman Institute for Molecular Biology, and the National Institute of Health Research and Development (Litbangkes), Ministry of Health, Jakarta, Indonesia. Written informed consent was obtained from all participants. The trial protocol is provided in the appendix.

### Randomisation and masking

The 78 antenatal clinic clusters were matched in triplicate on the basis of location, size, and malaria transmission intensity (appendix p 5). Before the study, the randomisation sequence to allocate clusters to the three intervention arms (1:1:1) was computer generated by the study statistician at the Liverpool School of Tropical Medicine (appendix pp 5–6) and forwarded to Indonesia. The final allocation was achieved during a public ceremony in which local health officials drew one of three identical looking opaque sealed envelopes which assigned their cluster to one of the three study interventions (SST, IPT, or IST; appendix pp 5–6). Study participants, local study nurses and midwives, and the local study coordinators were aware of the treatment allocation. Laboratory staff and off-site study investigators, including the study statistician, remained masked to treatment allocation until after database lock, approval of the statistical analysis plan by the Data Monitoring and Ethical Committee, and completion of the analytical code on the basis of dummy allocation.

### Procedures

At enrolment, demographic, socioeconomic, and educational information, and data on ownership and use of LLINs, were collected, and medical and obstetric histories were taken. Gestational age was assessed by fundal height, and fetal viability confirmed by doppler ultrasonography. The pregnant women's axillary temperature, blood pressure, weight, and mid-upper arm circumference were measured, and a blood sample was taken for malaria microscopy, molecular malaria diagnostics (quantitative PCR [qPCR], nested PCR, and loop-mediated isothermal amplification [LAMP]; appendix p 7), immunological analyses, and measurement of haemoglobin concentration (Haemocue, HemoCue AB, Ängelholm, Sweden). In addition, malaria rapid diagnostic tests (First Response Malaria Ag pLDH–HRP2 Combo [I16FRC30]; Premier Medical Corporation, Nani Daman, India) were done at enrolment in all participants in the SST and IST groups, regardless of symptoms, and in symptomatic participants in the IPT group. All participants received an LLIN. Participants were assessed monthly until delivery. At each monthly follow-up visit, clinical, obstetric, and physical examinations were done, and a blood sample taken by fingerprick for malaria microscopy and LAMP–PCR (appendix pp 7–8). In addition, malaria rapid diagnostic tests were done from the same sample as used for microscopy and LAMP-PCR in all participants in the IST group, regardless of symptoms, but only in symptomatic participants in the SST and IPT groups. Participants were encouraged to make unscheduled visits or contact staff if they felt ill or were concerned about their pregnancy. Participants were assessed for adverse events during each scheduled and unscheduled visit.

Participants in the SST clusters were screened with malaria rapid diagnostic tests for malaria infection, regardless of symptoms, at their first antenatal (enrolment) visit only. At subsequent monthly visits, they were tested with malaria rapid diagnostic tests if they were febrile (axillary temperature ≥37·5°C) or had a history of fever in the previous 48 h. The procedures in the IST group were identical to those in the SST group, except that participants were screened with malaria rapid diagnostic tests at each scheduled monthly visit. Participants in the IPT group received 4 mg/kg per day dihydroartemisinin and 18 mg/kg per day piperaquine (in 40 mg/320 mg tablets; Eurartesim, Sigma-Tau, Rome, Italy) at each monthly visit, at which they were not screened for malaria, unless they were febrile or had a history of fever in the past 48 h. The dose was the same throughout pregnancy and consisted of the standard 3-day course of two tablets for participants weighing less than 36 kg, three tablets for participants weighing 36–75 kg, or four tablets for participants weighing 75 kg or more at enrolment. The first dose was provided with a glass of water as directly observed therapy in the clinic. Participants were provided with the remaining two doses to be taken at home. All participants were contacted on day 2 and visited on day 3 to assess adherence and tolerance. In case of vomiting within 30 min, the full dose was repeated. Additionally, all participants who were positive for malaria on rapid diagnostic tests (positive HRP2 or pLDH bands) in all groups were treated with dihydroartemisinin–piperaquine (the same 3-day weight-based treatment as used for the IPT group). Participants with a history of dihydroartemisinin–piperaquine intake in the previous 4 weeks received quinine–clindamycin (10 mg/kg twice daily for 7 days).

At delivery, a maternal blood sample was taken for the same malaria metrics, and placental and umbilical-cord blood samples for histology, malaria rapid diagnostic tests, microscopy, and LAMP and PCR (appendix pp 7–8). Newborns were weighed on a digital scale (±10 g) and their gestational age assessed by means of the modified Ballard score.^21^ The presence of jaundice and congenital anomalies detectable by surface examination were assessed at delivery, day 7, and the final visit at 6–8 weeks. In between scheduled visits, infants were followed up passively.

We extracted DNA from dried blood spots using the Chelex method and tested for malaria using LAMP Pan-kits. LAMP-positive samples and 5% of negative samples were then tested by qPCR for verification and species identification, and discordant samples retested with nested-PCR (appendix pp 7–8).

Electrocardiography was done in a subgroup of 33 participants in the IPT group (selected through convenience sampling) to establish whether previously documented transient QTc prolongation associated with piperaquine increased in magnitude with subsequent courses (appendix p 10).

### Outcomes

The primary outcome was malaria (any species) at delivery, defined as a composite of maternal malaria (detection of infection in peripheral blood with microscopy, rapid diagnostic test, or LAMP–PCR) or placental malaria (detection of infection in placental blood with microscopy, LAMP–PCR, or histology [active infection]; appendix pp 6–8).

Secondary outcomes at delivery comprised the individual components of the primary composite outcome (maternal or placental malaria), detected by any method and by each method separately. Placental malaria infection detected by histology was classified as active acute, active chronic, active any, past, or any (active or past). In post-hoc analyses, malaria infection in the peripheral blood was stratified further by species. In addition, maternal anaemia (any: haemoglobin level <11 g/dL; moderate: haemoglobin level <9 g/dL) was assessed at delivery.

Secondary outcomes during pregnancy comprised maternal malaria, detected with any method and by each method separately. This outcome was further stratified by patent infection (positive microscopy or malaria rapid diagnostic test) and sub-patent infection (negative microscopy and malaria rapid diagnostic test and positive LAMP–PCR). Morbidity outcomes assessed during pregnancy comprised clinical malaria (documented or history of fever plus positive malaria rapid diagnostic test or microscopy) and unscheduled clinic visits for any reason and for all reasons unrelated to malaria.

Newborn secondary efficacy outcomes included congenital malaria, mean cord haemoglobin concentration, fetal anaemia (haemoglobin <12·5 g/dL), mean birthweight, low birthweight (<2500 g), mean gestational age, preterm delivery (<37 weeks' gestation), mean birthweight for gestational age (Z scores), small for gestational age,^22^ fetal loss (spontaneous abortion at <28 weeks' gestation or stillbirth), and the composite outcomes of adverse livebirth (preterm, low birthweight, or small for gestational age) and adverse pregnancy (adverse livebirth or fetal loss). Other secondary efficacy outcomes in the infant included the incidence of clinical malaria and all-cause and non-malaria illness by the end of follow-up (age 6–8 weeks). Mortality outcomes included neonatal, perinatal, and mortality up to age 6–8 weeks.

Safety outcomes included serious adverse events in the mother or infant, overall and by system organ class and preferred Medical Dictionary of Regulatory Affairs term; maternal deaths; congenital anomalies; and QTc prolongation.

### Statistical analysis

The trial was initially designed to detect a 50% reduction in malaria at delivery with IPT or with IST relative to SST across both sites pooled. Following recommendations from the ethics committee in Indonesia on June 27, 2014, to stop recruitment in Sumba because of the unexpected low malaria prevalence in the area, a blinded interim re-estimation of sample size was done with the aim to provide the study with 80% power across both sites pooled and 85% power in Papua alone to detect at least a 50% reduction in the primary outcome (two-sided α value of 0·0167, intracluster correlation coefficient of 0·005; appendix p 9). The revised study required 2279 participants (1290 from Papua and 989 from Sumba), accounting for a 13% efficiency loss owing to varying cluster sizes and 20% loss to follow-up.

The modified intention-to-treat (ITT) population included all randomised participants with outcome data. We also assessed all efficacy outcomes in the per-protocol population, which included participants in the modified ITT population who attended every scheduled visit and took all study doses on each occasion. The safety-population included participants who received at least one dose of study drug in any of the study arms.

Generalised estimating equation (GEE) models, with treatment group as a predictor and clinic as a cluster effect, were used. Log binomial GEE models were used to obtain risk ratios (RRs) for binary outcomes (including the cumulative risk), and linear GEE models to obtain mean differences for continuous outcomes. The unadjusted analysis, stratified by site, was considered the primary analysis. Because matching was ineffective and the number of triplets small in each site, unmatched analysis of the matched data was done to maximise power as soon as it became clear from the comparison of the baseline data that the matching was not successful.^23^, ^24^ Separate models were run per site (pre-planned), and differences in treatment effects compared by means of the Altman-Bland method.^25^

Secondary, covariate-adjusted analyses of the efficacy endpoints were done with seven prespecified, individual-level covariates and one post-hoc, cluster-level covariate (prevalence of malaria infection at enrolment as a proxy for malaria transmission). The individual-level covariates were study site (overall models only), malaria status at enrolment (binary), season during pregnancy (terciles based on average rainfall during the last 6 months of pregnancy), socioeconomic status (terciles of Socioeconomic Index^27^], calculated with principal component analysis), gestational age at enrolment (binary, based on the median value), gravidity (primigravidae or secundigravidae *vs* multigravidae), and use of insecticide-treated net during pregnancy. Simple imputation was used for missing covariates (<1%); no imputation was used for missing outcome variables. These same covariates, as well as a post-hoc covariate for species on enrolment, were used for subgroup analyses by adding them as interaction terms with treatment group. GEE Poisson regression, with time of follow-up as an offset, was used to obtain incidence rate-ratios.

The analysis was done with SAS version 9.3 and Stata version 14. The Data Monitoring and Ethical Committee oversaw the study. A cost-effectiveness analysis will be published elsewhere. The trial was registered with ISRCTN, number ISRCTN34010937.

### Role of the funding source

The funders of the study had no role in study design, data collection, data analysis, data interpretation, or writing of the report. RA, CK, FOtK, TC, and DW had full access to all the data in the study. The corresponding author had final responsibility for the decision to submit for publication.

## Results

**Figure 1:**
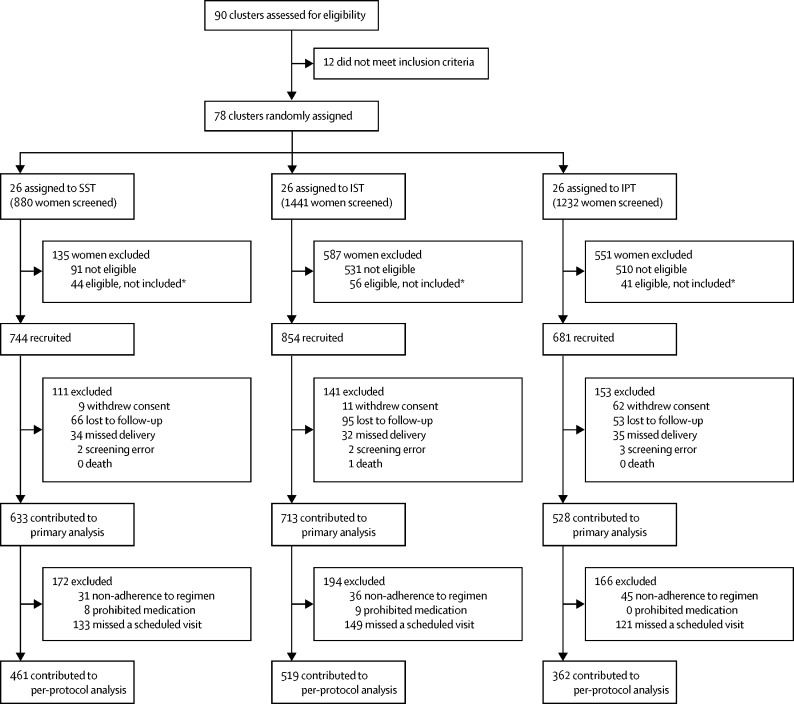
Trial profile IPT=intermittent preventive treatment. IST=intermittent screening and treatment. SST=single screening and treatment. *The number of recruited participants per cluster was restricted to a maximum of five per day to keep the number needed to follow-up manageable in subsequent visits. On some days, more than five participants were eligible, in which case they were chosen at random by drawing lots among all eligible participants who presented that morning.

Between May-16, 2013, and April-21, 2016 (when the required sample size was reached), 78 clusters (57 in Sumba and 21 in Papua) were randomly assigned to SST, IPT, or IST (26 clusters each). 3553 women were screened for inclusion, of whom 2279 (64·1%) were enrolled (989 from Sumba and 1290 from Papua; figure 1). The last delivery occurred on Oct-9, 2016, and the last infant follow-up on Nov-26, 2016. Overall, 7350 (85·4%) of 8609 scheduled antenatal visits were attended (appendix p 12). At baseline, 215 (9·4%) of 2279 participants had peripheral parasitaemia; the prevalence was similar in the SST (94 [12·6%] of 744) and IPT (72 [10·6%] of 681) groups, but lower in the IST group (49 [5·7%] of 854; table 1). Overall 418 (90%) of 463 cases of peripheral parasitaemia detected by LAMP–PCR were below the limit of detection for malaria rapid diagnostic tests.

**Table 1:** Baseline characteristics of enrolled participants

		**Sumba**			**Papua**			**Pooled sites**		
		SST (n=337)	IST (n=359)	IPT (n=293)	SST (n=407)	IST (n=495)	IPT (n=388)	SST (n=744)	IST (n=854)	IPT (n=681)
Maternal age, years		28·1 (6·1)	27·8 (5·9)	28 (6·0)	26 (6·1)	25·9 (6·7)	25·8 (6·0)	27 (6·2)	26·7 (6·4)	26·8 (6·1)
Residence, rural *vs* semi-urban		95·0% (320/337)	91·1% (327/359)	90·1% (264/293)	59·2% (241/407)	67·9% (336/495)	58·0% (225/388)	75·4% (561/744)	77·6% (663/854)	71·8% (489/681)
Marital status, single*vs married		62·3% (210/337)	65·2% (234/359)	63·1% (185/293)	34·6% (141/407)	27·1% (134/495)	35·3% (137/388)	47·2% (351/744)	43·1% (368/854)	47·3% (322/681)
Used a bednet previous night		22·3% (75/337)	25·3% (91/359)	22·5% (66/293)	49·4% (201/407)	45·7% (226/495)	53·1% (206/388)	37·1% (276/744)	37·1% (317/854)	39·9% (272/681)
Attended school		91·1% (307/337)	87·2% (313/359)	88·1% (258/293)	86·0% (350/407)	94·7% (469/495)	89·4% (347/388)	88·3% (657/744)	91·6% (782/854)	88·8% (605/681)
Schooling level†										
	Low	22·0% (74/337)	23·4% (84/359)	22·9% (67/293)	22·1% (90/407)	11·7% (58/495)	15·5% (60/388)	22·0% (164/744)	16·6% (142/854)	18·6% (127/681)
	Medium	30·9% (104/337)	25·9% (93/359)	26·3% (77/293)	18·2% (74/407)	16·2% (80/495)	13·4% (52/388)	23·9% (178/744)	20·3% (173/854)	18·9% (129/681)
	High	21·1% (71/337)	23·1% (83/359)	28·0% (82/293)	22·6% (92/407)	24·2% (120/495)	18·8% (73/388)	21·9% (163/744)	23·8% (203/854)	35·8% (244/681)
	Highest	26·1% (88/337)	27·6% (99/359)	22·9% (67/293)	37·1% (151/407)	47·9% (237/495)	52·3% (203/388)	32·1% (239/744)	39·3% (336/854)	39·6% (270/681)
Socioeconomic Index score, terciles										
	Low	34·4% (116/337)	32·0% (115/359)	33·4% (98/293)	43·5% (177/407)	30·1% (149/495)	26·8% (104/388)	39·4% (293/744)	30·9% (264/854)	29·7% (202/681)
	Medium	32·6% (110/337)	33·4% (120/359)	34·8% (102/293)	32·4% (132/407)	33·1% (164/495)	34·3% (133/388)	32·5% (242/744)	33·3% (284/854)	34·5% (235/681)
	High	32·9% (111/337)	34·5% (124/359)	31·7% (93/293)	24·1% (98/407)	36·8% (182/495)	38·9% (151/388)	28·1% (209/744)	35·8% (306/854)	35·8% (244/681)
Pregnancy number, gravidity										
	One	28·2% (95/337)	28·7% (103/359)	28·0% (82/293)	26·0% (106/407)	26·5% (131/495)	29·6% (115/388)	27·0% (201/744)	27·4% (234/854)	28·9% (197/681)
	Two	22·3% (75/337)	24·0% (86/359)	20·5% (60/293)	29·2% (119/407)	31·5% (156/495)	29·1% (113/388)	26·1% (194/744)	28·3% (242/854)	25·4% (173/681)
	Three or more	49·6% (167/337)	47·4% (170/359)	51·5% (151/293)	44·7% (182/407)	42·0% (208/495)	41·2% (160/388)	46·9% (349/744)	44·3% (378/854)	45·7% (311/681)
Gestational age, weeks		24·1 (4·6)	24·1 (4·3)	24·1 (4·6)	23·7 (5·3)	22·8 (5·1)	23·8 (4·5)	23·9 (5)	23·4 (4·8)	23·9 (4·6)
Weight, kg		51·9 (7·0)	51·3 (6·8)	52·0 (8·1)	56·7 (5·3)	56·0 (9·6)	57·8 (9·4)	54·5 (8·6)	54·0 (8·8)	55·3 (9·3)
Height, cm		152·4 (5·4)	152·5 (5·6)	151·8 (6·3)	152·7 (5·3)	152·8 (5·5)	152·8 (5·3)	152·6 (5·3)	152·7 (5·5)	152·3 (5·8)
Mid-upper arm circumference, cm		24·6 (2·8)	24·2 (2·4)	24·7 (2·7)	25·3 (3·1)	25·6 (3·4)	25·8 (3·2)	25·0 (3·0)	25·0 (3·1)	25·3 (3·0)
Haemoglobin, g/dL		11·0 (1·4)	11·0 (1·6)	11·1 (1·5)	11·0 (1·9)	11·7 (1·8)	11·4 (1·9)	11·0 (1·7)	11·4 (1·8)	11·3 (1·7)
Plasmodium infection										
	mRDT‡	0·3% (1/337)	0·0% (0/359)	0·0% (0/2)	7·4% (30/407)	3·0% (15/495)	50·0% (3/6)	4·2% (31/744)	1·8% 15/854)	37·5% (3/8)
	Microscopy	0·6% (2/337)	0·3% (1/358)	0·7% (2/293)	9·1% (37/407)	3·6% (18/495)	5·9% (23/387)	5·2% (39/744)	2·2% (19/853)	3·7% (25/680)
	LAMP–PCR	6·5% (22/337)	2·8% (10/359)	8·6% (25/290)	11·8% (48/407)	5·9% (29/495)	9·0% (35/387)	9·4% (70/744)	4·6% (39/854)	8·9% (60/677)
	Any§	6·5% (22/337)	3·1% (11/359)	8·5% (25/293)	17·7% (72/407)	7·7% (38/495)	12·1% (47/388)	12·6% (94/744)	5·7% (49/854)	10·6% (72/681)
Infecting species¶										
	*Plasmodium falciparum,* mono-infection	3·3% (11/337)	1·1% (4/359)	5·1% (15/292)	8·1% (33/407)	4·0% (20/495)	7·7% (30/388)	5·9% (44/744)	2·8% (24/854)	6·6% (45/680)
	*Plasmodium vivax,* mono-infection	2·4% (8/337)	1·7% (6/359)	2·1% (6/292)	5·2% (21/407)	2·4% (12/495)	1·8% (7/388)	3·9% (29/744)	2·1% (18/854)	1·9% (13/680)
	*Plasmodium malariae* or *Plasmodium ovale,* mono-infection	0·0% (0/337)	0·0% (0/359)	0·0% (0/292)	1·0% (4/407)	0·0% (0/495)	0·0% (0/388)	0·5% (4/744)	0·0% (0/854)	0·0% (0/680)
	Mixed infection	0·6% (2/337)	0·3% (1/359)	0·7% (2/292)	3·4% (14/407)	1·2% (6/495)	2·6% (10/388)	2·2% (16/744)	0·8% (7/854)	1·8% (12/680)

Data are mean (SD) or % (n/N). SST=single screen and treatment. IST=intermittent screen and treatment. IPT=intermittent preventive therapy. mRDT=malaria rapid diagnostic test. LAMP=loop-mediated isothermal amplification.

* Single includes single unmarried participants only as there were no divorced, separated, or widowed participants. In Sumba, many participants were not legally married, but co-habiting with their partner and considered married within their local communities.

† Low was defined as no schooling or primary school not completed, medium as primary school completed, high as junior high school completed, and highest as senior high school or tertiary education completed.

‡ Data reflect mRDTs done in symptomatic participants in the IPT group and all participants in the SST and IST groups.

§ Includes mRDT results from symptomatic participants and microscopy and LAMP–PCR results from all participants.

¶ Typing was done by PCR; if PCR was not successful, species was based on microscopy.

Median follow-up was 3·1 months (IQR 2·1–4·0), with a median number of scheduled follow-up visits of three (range 1–6; appendix p 12). The median number of dihydroartemisinin–piperaquine courses in the IPT group was three (range 0–6). Ultimately, 1874 (82·2%) of 2279 women contributed to the primary endpoint (figure 1). These proportions did not differ significantly overall, but in Papua the proportion of enrolled participants who contributed to the primary endpoint was significantly lower in IPT clusters (70·1%) than in SST (84·3%; p=0·0005) and IST (86·5%) clusters (p<0·0001), whereas in Sumba it was lower in IST clusters (79·4%) than in IPT (87·4%; p=0·017) or SST (86·1%; p=0·070) clusters (appendix pp 14, 31).

**Figure 2:**
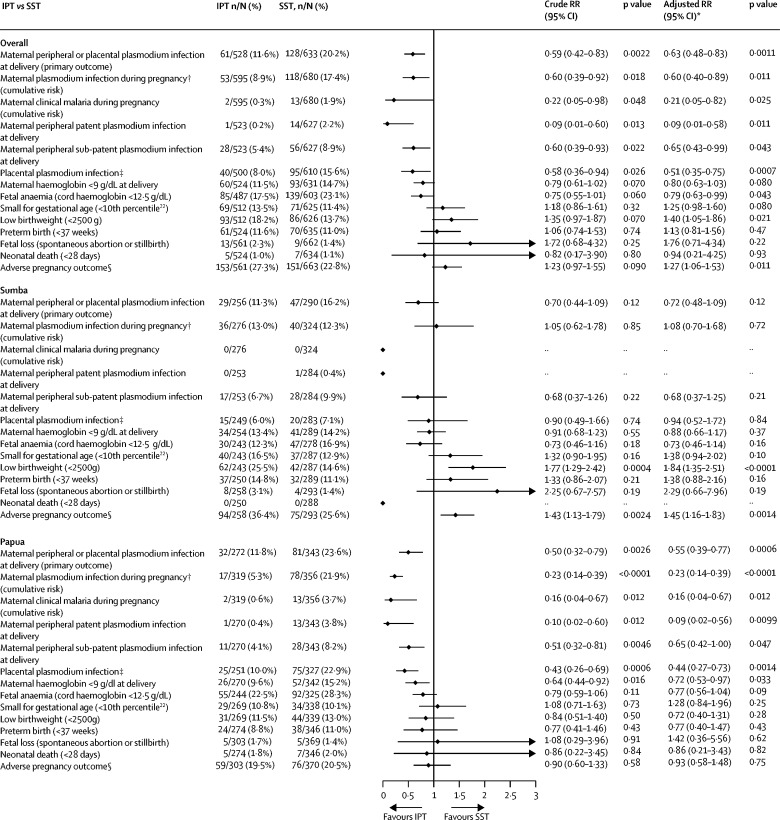
IPT versus SST for the primary outcome and key secondary outcomes, overall and by site (modified ITT population) ITT=intention to treat. LAMP=loop-mediated isothermal amplification. IPT=intermittent preventive treatment. SST=single screening and treatment. RR=relative risk. *Adjusted for site (in the overall models only) and six additional, prespecified, participant-level covariates. †Detected by LAMP, PCR, microscopy, or malaria rapid diagnostic test. ‡Detected by LAMP, PCR, microscopy, malaria rapid diagnostic test, or histology (active and past infection). §Defined as fetal loss, low birthweight, small for gestational age, or preterm birth.

**Figure 3:**
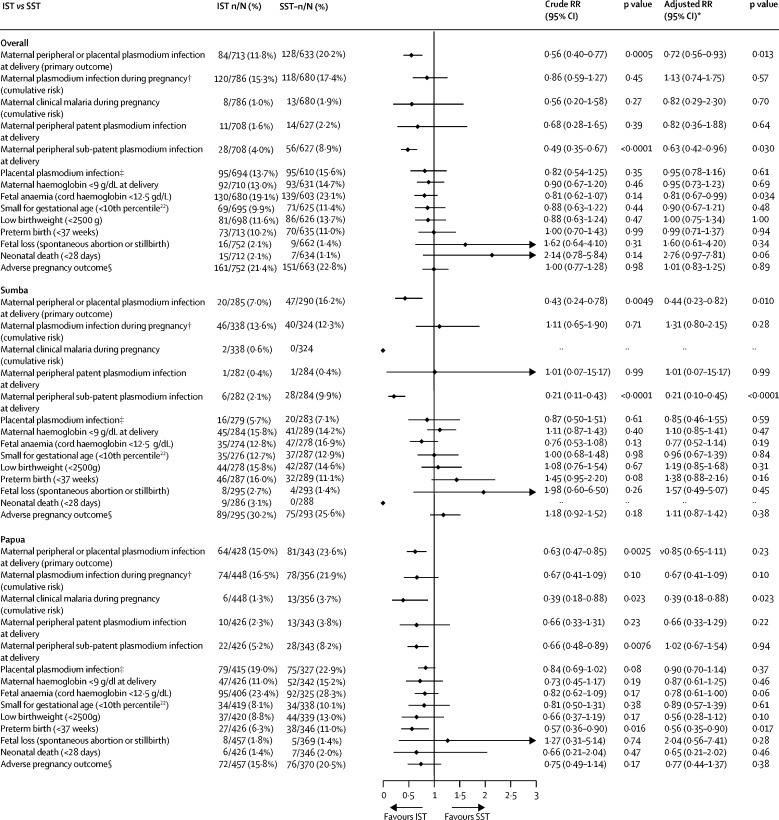
IST versus SST in the primary outcome and key secondary outcomes, overall and by site (modified ITT population) ITT=intention to treat. LAMP=loop-mediated isothermal amplification. IST=intermittent screening and treatment. SST=single screening and treatment. RR=relative risk. *Adjusted for site (in the overall models only) and six additional, prespecified, participant-level covariates. †Detected by LAMP, PCR, microscopy, or malaria rapid diagnostic test. ‡Detected by LAMP, PCR, microscopy, malaria rapid diagnostic test, or histology (active and past infection). §Defined as fetal loss, low birthweight, small for gestational age, or preterm birth.

**Figure 4:**
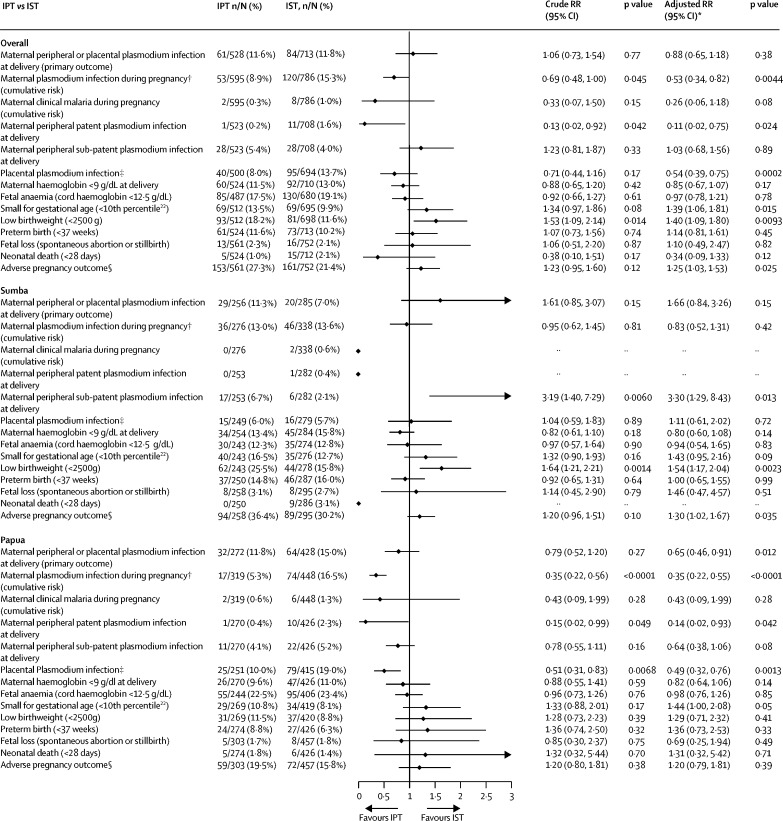
IPT versus IST in the primary outcome and key secondary outcomes, overall and by site (modified ITT population) LAMP=loop-mediated isothermal amplification. IPT=intermittent preventive treatment. IST=intermittent screening and treatment. RR=relative risk.*Adjusted for site (in the overall models only) and six additional, prespecified, participant-level covariates. †Detected by LAMP, PCR, microscopy, or malaria rapid diagnostic test. ‡Detected by LAMP, PCR, microscopy, malaria rapid diagnostic test, or histology (active and past infection). §Defined as fetal loss, low birthweight, small for gestational age, or preterm birth

The prevalence of malaria at delivery in the modified ITT population was 20·2% (128 of 633) in SST clusters compared with 11·6% (61 of 528) in IPT clusters (RR 0·59, 95% CI 0·42–0·83; p=0·0022) and 11·8% (84 of 713) in IST clusters (0·56, 0·40–0·77; p=0·0005; Figure 2, Figure 3). There was no significant difference in the prevalence of malaria at delivery between IPT and IST clusters (1·06, 0·73–1·54, p=0·77; figure 4). Similar results were obtained in covariate-adjusted analyses (Figure 2, Figure 3, Figure 4), across all subgroups (appendix pp 32–34), in the per-protocol population (appendix pp 35–37), and in a post-hoc sensitivity analysis with matched analysis (appendix p 15). Intracluster correlation coefficient values are shown in appendix p 16.

Analyses of secondary outcomes at delivery showed that relative to SST, IPT was associated with a reduction in patent infections and sub-patent infections in peripheral blood (figure 2; appendix p 38). For IST, the reduction was significant for sub-patent infections only (figure 3; appendix p 39). The prevalence of placental malaria detected by histology (active or past) or other methods was lower in IPT clusters than in SST clusters (appendix p 41). It was similar in IPT and IST clusters in the unadjusted analysis but lower in IST than in IPT clusters in the adjusted analysis (figure 4), owing to reductions in past infections with IPT (appendix p 43). There were no significant differences in malaria detected by placental histology between IST and SST (appendix p 42).

Analyses of secondary outcomes during pregnancy showed that the cumulative risk of incident malaria infection during pregnancy detected by at least one diagnostic method was lower in IPT than in SST clusters (figure 2). Similar results were seen for other definitions of antenatal malaria infection (appendix p 44). The cumulative risk of clinical malaria was also lower with IPT than with SST (figure 2). The cumulative risk of malaria detected by rapid diagnostic test was not significantly different between IPT and SST clusters (appendix p 42). In the IST clusters, by contrast, the antenatal incidence measures of malaria infection were similar to those in SST clusters (figure 3; appendix p 45). The cumulative proportion of participants with malaria infection detected by malaria rapid diagnostic test (including at enrolment) was also similar between IST and SST (34 [4·0%] of 854) and SST (39 [5·2%] of 744) groups (RR 0·91, 95% CI 0·36–2·32, p=0·84), despite the nearly four times higher number of screening events in IST than in SST clusters (2886 *vs* 744). The incidence of clinical malaria with IST was lower than with SST, but this difference was not significant (figure 3; appendix p 45). Relative to IST, IPT was significantly more effective in preventing malaria infections during pregnancy (figure 4; appendix p 46). Non-malaria outcomes were similar between the IPT and IST groups (appendix p 46).

There were some significant differences in the effects on the malaria infection outcomes by study site (Figure 2, Figure 3, Figure 4; appendix pp 17–25). For the primary endpoint, the p values of the interaction term depicting the difference between sites in the effect of IPT relative to IST were p=0·070 (unadjusted) and p=0·015 (adjusted; appendix p 17) with adjusted analyses showing no significant difference between the two groups in Sumba, but a relative risk reduction of 35% (0·65, 0·46-0·91, p=0·012, figure 2) in Papua. Similarly, the superior effects of IPT relative to IST on malaria infections detected in peripheral blood during pregnancy (figure 4), and in peripheral blood (appendix p 40) and placental blood at delivery (appendix p 43), were also evident only in Papua (appendix pp 19–23). The superior effect of IPT relative to SST on malaria infections in peripheral blood during pregnancy (figure 2) or in placental blood at delivery (appendix p 41) was also evident only in Papua (appendix pp 21–23). There were no significant differences by study site in the effect of IPT versus SST or IPT versus IST on the incidence of non-malaria and all-cause sick visits (appendix p 23).

The analysis of secondary morbidity outcomes showed no significant differences in any anaemia (haemoglobin <11 g/dL) or mean maternal haemoglobin levels (appendix p 27) between groups when both sites were pooled, but in Papua, participants in IPT clusters had a lower prevalence of moderate anaemia (haemoglobin <9 g/dL) than in SST clusters (figure 2). IPT, but not IST, was associated with significantly higher mean haemoglobin concentrations in cord blood (p=0·020; appendix p 25). Relative to SST, IPT and IST did not significantly improve adverse pregnancy outcomes when both sites were pooled (Figure 2, Figure 3), but in Sumba, the risk of adverse pregnancy outcomes was significantly higher in the IPT group than in the SST group (figure 2). This difference was not apparent in Papua (figure 2; appendix p 25).

The main adverse events associated with dihydroartemisinin–piperaquine were nausea, headache, and vomiting within 7 days after drug intake. In the IPT group, nausea occurred after 63 (3%) of 2058 courses, headache after 68 (3%) of 2050 courses, and vomiting after 87 (4%) of 2058 courses (appendix p 29). There was no difference between groups in the number of serious adverse events in mothers (table 2). There were three maternal deaths, two in the IST group and one in the SST group; all were considered unrelated to the intervention or malaria (appendix p 30). The prevalence of serious adverse events in infants (table 2), and the risk of congenital malformations, were very similar between groups (appendix pp 47–49).

**Table 2:** Serious adverse events

	IPT (n=681 women, n=524 infants)*			**IST (n=854 women, n=712 infants)**†			**SST (n=744 women, n=634 infants)**‡		
	Number of participants with event (%)	Total number of events	Incidence per 100 person-years (95% CI)	Number of participants with event (%)	Total number of events	Incidence per 100 person-years (95% CI)	Number of participants with event (%)	Total number of events	Incidence per 100 person-years (95% CI)
**Mothers**									
Any serious adverse event	56 (8·2%)	85	54·2 (43·3–67·1)	77 (9·0%)	105	47·2 (38·6–57·2)	71 (9·5%)	113	60·4 (49·8–72·6)
Pregnancy, puerperium and perinatal conditions	47 (6·9%)	62	39·6 (30·3–50·7)	60 (7·0%)	71	31·9 (25–40·3)	51 (6·9%)	65	34·7 (26·8–44·3)
Infections and infestations	8 (1·2%)	8	5·1 (2·2–10·1)	9 (1·1%)	11	4·9 (2·5–8·9)	17 (2·3%)	26	13·9 (9·1–20·4)
Gastrointestinal disorders	2 (0·3%)	3	1·9 (0·4–5·6)	1 (0·1%)	2	0·9 (0·1–3·3)	2 (0·3%)	3	1·6 (0·3–4·7)
Surgical and medical procedures	0	0	0 (0–0)	1 (0·1%)	1	0·4 (0–2·5)	1 (0·1%)	1	0·5 (0–3)
Blood and lymphatic system disorders	7 (1·0%)	7	4·5 (1·8–9·2)	12 (1·4%)	14	6·3 (3·4–10·6)	15 (2·0%)	15	8 (4·5–13·2)
Nervous system disorders	0	0	0 (0–0)	0	0	0 (0–0)	1 (0·1%)	2	1·1 (0·1–3·9)
Reproductive system and breast disorders	1 (0·1%)	1	0·6 (0–3·6)	1 (0·1%)	1	0·4 (0–2·5)	0	0	0 (0–0)
Injury, poisoning and procedural complications	0	0	0 (0–0)	1 (0·1%)	1	0·4 (0–2·5)	0	0	0 (0–0)
Respiratory, thoracic and mediastinal disorders	1 (0·1%)	1	0·6 (0–3·6)	0	0	0 (0–0)	0	0	0 (0–0)
Vascular disorders	1 (0·1%)	1	0·6 (0–3·6)	0	0	0 (0–0)	0	0	0 (0–0)
Metabolism and nutrition disorders	1 (0·1%)	1	0·6 (0–3·6)	2 (0·2%)	2	0·9 (0·1–3·3)	0	0	0 (0–0)
Hepatobiliary disorders	0	0	0 (0–0)	1 (0·1%)	1	0·4 (0–2·5)	0	0	0 (0–0)
Cardiac disorders	0	0	0 (0–0)	1 (0·1%)	1	0·4 (0–2·5)	1 (0·1%)	1	0·5 (0–3)
Neoplasms benign, malignant and unspecified (incl cysts and polyps)	1 (0·1%)	1	0·6 (0–3·6)	0	0	0 (0–0)	0	0	0 (0–0)
**Infants**									
Any serious adverse event	40 (7·6%)	56	88 (66·5–114·3)	53 (7·4%)	75	89·5 (70·4–112·2)	51 (8·0%)	69	86·5 (67·3–109·5)
Congenital, familial and genetic disorders	11 (2·1%)	11	17·3 (8·6–30·9)	18 (2·5%)	19	22·7 (13·7–35·4)	19 (3·0%)	19	23·8 (14·3–37·2)
Pregnancy, puerperium and perinatal conditions	6 (1·1%)	6	9·4 (3·5–20·5)	14 (2·0%)	14	16·7 (9·1–28)	10 (1·6%)	12	15 (7·8–26·3)
Infections and infestations	18 (3·4%)	19	29·9 (18–46·6)	23 (3·2%)	23	27·5 (17·4–41·2)	24 (3·8%)	25	31·4 (20·3–46·3)
Gastrointestinal disorders	0	0	0 (0–0)	1 (0·1%)	1	1·2 (0–6·7)	2 (0·3%)	2	2·5 (0·3–9·1)
Surgical and medical procedures	0	0	0 (0–0)	1 (0·1%)	1	0 (0–0)	0	0	0 (0–0)
Blood and lymphatic system disorders	1 (0·2%)	1	1·6 (0–8·8)	2 (0·3%)	2	2·4 (0·3–8·6)	0	0	0 (0–0)
Nervous system disorders	1 (0·2%)	1	1·6 (0–8·8)	1 (0·1%)	1	1·2 (0–6·7)	0	0	0 (0–0)
Reproductive system and breast disorders	1 (0·2%)	1	1·6 (0–8·8)	0	0	0 (0–0)	0	0	0 (0–0)
Respiratory, thoracic, and mediastinal disorders	13 (2·5%)	13	20·4 (10·9–34·9)	9 (1·3%)	9	10·7 (4·9–20·4)	10 (1·6%)	10	12·5 (6–23·1)
Metabolism and nutrition disorders	0	0	0 (0–0)	3 (0·4%)	3	3·6 (0·7–10·5)	0	0	0 (0–0)
Hepatobiliary disorders	0	0	0 (0–0)	0	0	0 (0–0)	1 (0·2%)	1	1·3 (0–7)
General disorders and administration site conditions	2 (0·4%)	3	4·7 (1–13·8)	2 (0·3%)	2	2·4 (0·3–8·6)	0	0	0 (0–0)
Neoplasms benign, malignant, and unspecified (including cysts and polyps)	1 (0·2%)	1	1·6 (0–8·8)	0	0	0 (0–0)	0 (0·0%)	0	0 (0–0)

All SAEs were coded using the Medical Dictionary of Regulatory Affairs and are presented here according to their system organ class, the highest level in the dictionary. IPT=intermittent preventive treatment. IST=intermittent screening and treatment. SST=single screening and treatment.

§ Excluding twin births, including liveborn and stillborn infants.

* Total follow-up was 222·2 years in women and 83·8 years in infants.

† Total follow-up was 156·6 years in women and 63·6 years in infants.

‡ Total follow-up was 187·1 years in women and 79·7 years in infants.

In total, 33 participants in the IPT group were enrolled in the nested cardiac monitoring. Dihydroartemisinin–piperaquine was associated with a mean QTcF prolongation of 20 ms (SD 19·6) and a mean QTcB prolongation of 14·8 ms (17·6). Neither the mean QTcF nor mean QTcB increased with the total number of dihydroartemisinin–piperaquine courses administered (appendix pp 52–53).

## Discussion

This trial highlights that in areas co-endemic for both *P falciparum* and *P vivax* in Indonesia, IPT with dihydroartemisinin–piperaquine compared with the predominantly passive detection afforded by the existing standard SST strategy resulted in a reduction of about 41% in the prevalence of malaria infection at delivery, a similar reduction in its incidence during pregnancy, and a 78% reduction in the incidence of clinical malaria during pregnancy. The effect was evident for both *P falciparum* and *P vivax* infections (appendix p 38), suggesting that monthly dihydroartemisinin–piperaquine was able to successfully delay *P vivax* relapses in the absence of primaquine, which is contraindicated during pregnancy. Of note was the marked difference in the efficacy of IPT between the two study sites. The beneficial effect was evident only in Papua, the higher transmission site, where the prevalence at delivery was reduced by 50% and the antenatal malaria incidence by 77%, similar to the reductions in similar outcomes observed in previous trials with IPT with dihydroartemisinin–piperaquine in western Kenya^9^ and Uganda,^10^ in which the comparator was IPT with sulfadoxine–pyrimethamine. The greatest reductions were observed for patent parasitaemia (malaria rapid diagnostic test or microscopy positive), which was 91% lower with IPT than with SST at delivery, whereas for subpatent infections, the reduction was 40%. Infants in the IPT cluster had significantly higher mean haemoglobin levels at birth, but otherwise IPT was not associated with improvements in birth outcomes.

Compared with SST, the effect of IST was not consistent. Although IST was associated with a 44% lower prevalence of the primary outcome at delivery, the prevalence of malaria was already 55% lower at enrolment. Furthermore, the effect was only evident at delivery, with no evidence that IST was associated with reductions in the incidence of parasitaemia during pregnancy. Few participants in the SST and IST groups tested positive on malaria rapid diagnostic tests at enrolment or during pregnancy, and contrary to expectations, IST did not detect more infections than SST, despite the four times greater number of screening events. Because the number of participants who tested positive for malaria by malaria rapid diagnostic tests were similar, a similarly low number of participants in the IST and SST groups required treatment and thus benefited from the potential post-treatment prophylactic effect of piperaquine. The observed differences between these two groups in the primary outcome at delivery might thus reflect the lower transmission intensity in the IST clusters that was evident at enrolment or other unknown confounding effects rather than a true intervention effect.

Although, there was no difference between IPT and IST in the composite primary endpoint overall, analyses that adjusted for differences in baseline malaria showed a significant difference in treatment effect between Sumba and Papua. There was no significant difference between the two groups in Sumba, but a 35% reduction with IPT versus IST in the primary outcome in Papua in the adjusted analysis. IPT was also more effective than IST in reducing malaria infection during pregnancy in Papua. The lack of a difference in the effects of IPT versus IST in Sumba might reflect the lower transmission intensity compared to Papua. It may also reflect the nearly threefold difference between the IPT and IST clusters in malaria risk that was already evident at enrolment in Sumba.

Approximately 90% of infections were below the limit of detection for malaria rapid diagnostic tests. The brand used in the trial did well in the WHO product testing of malaria rapid diagnostic tests and was the best-performing malaria rapid diagnostic test to screen for malaria in asymptomatic pregnant women in our previous diagnostic study in Sumba;^14^ although it had an overall sensitivity of 32% to detect PCR-positive infections, and only 13% for *P vivax* mono-infections.^14^ The tests were purchased directly from the manufacturer and stored and used according to the manufacturer's instructions.

Dihydroartemisinin–piperaquine was well tolerated, with only one participant in the IPT group vomiting within 30 min after any dose, an adverse event rate that was similar to that reported with sulfadoxine–pyrimethamine in sub-Saharan Africa.^9^, ^10^, ^11^ The main adverse events were later vomiting, nausea, and headache within 3 days after drug intake. Overall, among the participants who took dihydroartemisinin–piperaquine, almost 90% complied with the 3-day regimen each time it was administered. The magnitude of QTc prolongation associated with piperaquine was consistent with that seen in other studies with a single course of dihydroartemisinin–piperaquine and similar to previous trials of IPT with dihydroartemisinin–piperaquine in Uganda.^9^, ^10^ There was no evidence that QTc prolongation increased with subsequent monthly courses, despite the potential for dose accumulation of piperaquine when dihydroartemisinin–piperaquine is given monthly.^27^ In Sumba, there was a higher number of neonatal deaths in the IST clusters than in the other clusters, which was unexplained and could have occurred by chance given the deaths were not related to dihydroartemisinin–piperaquine use and this difference was not observed in Papua. The risk of low birthweight was higher in IPT clusters than in SST or IST clusters, which was probably a chance finding given that this difference was observed only in Sumba.

In a linked feasibility analysis, monthly screening with malaria rapid diagnostic tests was found to be well accepted by asymptomatic participants and providers.^28^ By contrast, in this current study, the withdrawal rate was relatively high in the IPT cluster, particularly in Papua, where 14% of participants withdrew, compared with 0% and 2% in the IST and SST clusters, respectively. High rates of withdrawal from IPT were related to concerns about dihydroartemisinin–piperaquine causing potential harm to the mother and baby and being a potential driver of drug resistance.^28^ The concept of using dihydroartemisinin–piperaquine for chemoprevention in asymptomatic individuals is new in this region, where to date it has been used only for case management of febrile patients with acute malaria. There is an increasing interest in use of dihydroartemisinin–piperaquine for chemoprevention,^9^, ^10^, ^13^ as well as for mass drug administration. In this context it is imperative that careful consideration be given to the optimal use of antimalarials for both treatment and prevention, ideally with drugs that generate opposing selection pressures on the same target.^29^ Further feasibility studies with dihydroartemisinin–piperaquine as a monthly IPT are also warranted before its implementation in the region.

The study has several important limitations. First, we used a cluster-randomised design, which, owing to the modest number of assignment units per arm (26 clinic clusters), had a greater potential for bias than trials based on randomisation of individuals. The lower prevalence of malaria at enrolment in the IST arm occurred by chance in both Sumba and Papua, despite our attempt to balance the randomisation by malaria transmission using locally available annual parasite incidence data from the government (appendix p 54). The unequal distribution is a potential cause of bias because of the strong correlation between malaria infection at enrolment and delivery. This is likely to have resulted in overestimation of the effect of IST relative to SST and underestimation of the effect of IPT relative to IST. Second, the study was not powered to detect differences in birth outcomes. Furthermore, in Sumba, the malaria transmission intensity was lower than in previous years, limiting the power to detect differences in infection outcomes, especially for patent infections, which were detected in only 0·5% of participants at enrolment compared with 6·5% in Papua. Patent infections are most likely to be associated with adverse pregnancy outcomes.^3^, ^30^, ^31^, ^32^, ^33^, ^34^, ^35^ Third, only 82% of participants contributed to the primary endpoint, which required collection of maternal and placental blood within a few hours of delivery. Lastly, because HRP2 can remain detectable for up to 1 month after parasite clearance in patients with clinical malaria, malaria rapid diagnostic tests can remain positive for several weeks. This is unlikely to have affected our findings as there were no cases with a positive malaria rapid diagnostic test and a negative qPCR within 1 month.

In conclusion, the effect of IST relative to SST was difficult to ascertain as the 44% difference detected at delivery was already evident at baseline (55% difference) and very few participants tested positive by malaria rapid diagnostic test in this setting, where about 90% of infections were below the limit of detection. Further studies with highly sensitive malaria rapid diagnostic tests should be considered. By contrast, our results suggest that in areas in the Asia-Pacific region with moderate transmission and high-grade sulfadoxine–pyrimethamine and chloroquine resistance, the strategy of monthly IPT with dihydroartemisinin–piperaquine could be an effective alternative to the existing policy of single screening and treatment.

## Data sharing

All individual-participant data collected during this trial will be available to access, after de-identification. Data and documents, including the study protocol and statistical analysis plan will be available. Data access will be provided to researchers after a proposal has been approved by an independent review committee identified for this purpose. An agreement on how to collaborate will be reached based on any overlap between the proposal and any ongoing efforts. Data will be available beginning at 3 months after publication of this Article. Proposals should be directed to feiko.terkuile@lstmed.ac.uk; to gain access, data requesters will need to sign a data access agreement, and the de-identified database will be transferred by email.
