# Supplementary material for: Efficacy and safety of intermittent preventive treatment and intermittent screening and treatment versus single screening and treatment with dihydroartemisinin–piperaquine for the control of malaria in pregnancy in Indonesia: a cluster-randomised, open-label, superiority trial
Source: Lancet Infect Dis. 2019 Sep;19(9):973–87. doi: 10.1016/S1473-3099(19)30156-2 (PMC6715823; doi:10.1016/S1473-3099(19)30156-2)
Supplement: Supplementary appendix [file mmc1.pdf]

THE LANCET Infectious Diseases

Supplementary webappendix

This webappendix formed part of the original submission and has been peer reviewed. We post it as supplied by the authors.

Supplement to: Ahmed R, Poespoprodjo JR, Syafruddin D, et al. Efficacy and safety of intermittent preventive treatment and intermittent screening and treatment versus single screening and treatment with dihydroartemisinin–piperaquine for the control of malaria in pregnancy in Indonesia: a cluster-randomised, open-label, superiority trial. *Lancet Infect Dis* 2019; published online July 25.

SUPPLEMENTAL CONTENT

Ahmed, R., et al: Intermittent preventive treatment (IPT), intermittent screening and treatment (IST) and single screening and treatment (SST) with dihydroartemisinin-piperaquine for the control of malaria in pregnancy in Indonesia: A cluster-randomised, open-label superiority trial

Contents

eMethods 4

Details of study site and antenatal clinic 4

Details of randomisation and public ceremony 5

Definitions of morbidity endpoints 6

Assessment of compliance and tolerance to DP intake 6

Laboratory methods 6

mRDTs, malaria microscopy, haemoglobin assessment and histopathology 6

Molecular methods 7

Sample size and power calculations 9

Original sample size calculations 9

Revised calculations following the interim power and sample size re-estimation 9

Cardiac monitoring 10

Other sub studies 11

eResults 12

eTables 12

eTable 2: Follow-up visits schedule (intention to treat population) 12

eTable 3: Proportion of women with missing data for the primary outcome by treatment arm 14

eTable 4: Results from GEE model analysis of the primary outcome with and without breaking the matching, overall and by site 15

eTable 5: Intra-cluster correlation coefficient (ICC) for primary endpoint 16

eTable 6: Site-treatment interaction P-values for malaria at the time of delivery (primary outcome) and key secondary outcomes 17

eTable 7: Site-treatment interaction P-values for secondary outcomes related to malaria at the time of delivery 19

eTable 8: Site-treatment interaction P-values for secondary outcomes related to placental malaria 21

eTable 9: Site-treatment interaction P-values for malaria outcomes and non-malaria sick visits during pregnancy 23

eTable 10: Site-treatment interaction P-values for key secondary outcomes newborn 25

eTable 11: Maternal and fetal mean haemoglobin, birthweight, gestational age and mean birthweight-for gestational age Z-score by treatment group and site 27

eTable 12: Adverse events associated with drug tolerability 29

eTable 13: Serious Adverse Events: case descriptions of maternal deaths 30

eFigure detailed trial profile 31

eFigure 1: Trial profile by arm and overall 31

eFigures efficacy outcomes 32

eFigure 2: Subgroup analysis of the effect on the primary outcomes in the ITT population (IPT vs SST) 32

eFigure 3: Subgroup analysis of the effect on the primary outcomes in the ITT population (IST vs SST) 33

eFigure 4: Subgroup analysis of the effect on the primary outcomes in the ITT population (IPT vs IST) 34

eFigure 5: Malaria at delivery (primary outcome) and key secondary outcomes in the per protocol population (IPT vs SST) 35

eFigure 6: Malaria at delivery (primary outcome) and key secondary outcomes in the per protocol population (IST vs SST) 36

eFigure 7: Malaria at delivery (primary outcome) and key secondary outcomes in the per protocol population (IPT vs IST) 37

eFigure 8: Malaria at delivery by treatment group and site in the intention-to-treat population (IPT vs SST) 38

eFigure 9: Malaria at delivery by treatment group and site in the intention-to-treat population (IST vs SST) 39

eFigure 10: Malaria at delivery by treatment group and site in the intention-to-treat population (IPT vs IST) 40

eFigure 11: Placental malaria by treatment group and site in the intention-to-treat population (IPT vs SST) 41

eFigure 12: Placental malaria by treatment group and site in the intention-to-treat population (IST vs SST) 42

eFigure 13: Placental malaria by treatment group and site in the intention-to-treat population (IPT vs IST) 43

eFigure 14: Malaria and non-malaria sick visits during pregnancy by treatment group in the intention-to-treat population (IPT vs SST) 44

eFigure 15: Malaria and non-malaria sick visits during pregnancy by treatment group in the intention-to-treat population (IST vs SST) 45

eFigure 16: Malaria and non-malaria sick visits during pregnancy by treatment group in the intention-to-treat population (IPT vs IST) 46

eFigure 17: Secondary outcomes newborn: birth outcomes and neonatal follow-up (IPT vs SST)) 47

eFigure 18: Secondary outcomes newborn: birth outcomes and neonatal follow-up (IST vs SST) 48

eFigure 19: Secondary outcomes newborn: birth outcomes and neonatal follow-up (IPT vs IST) 49

Cardiac Monitoring results 50

Results (text) 50

eTable 14: Cardiac monitoring; mean and mean change in QTcF and QTcB at baseline and 4 to 6 hours after the third dose of each course 52

eFigure 20: Cardiac monitoring; mean (95% CI) QTc at baseline (before the first course of IPT-DP) and 4-6 hours after the last dose of each course by visit number (left panel) and mean (95% CI) change in QTc after each course compared to baseline using Fridericia's method (top panels) and Bazett's method (bottom panels) 53

eReferences 54

eMethods

The protocol is available as supplemental information.

Details of study site and antenatal clinic

The study area consisted of several sub-districts. In each sub-district, there is one community health centre ("*Puskesmas*") that serves a population of around 30,000 people. Under each *Puskesmas* maternal health services are provided through sub-health Posts (*Pustu*) based in the communities and covering about 2-3 villages with a population size of 500-1500 people per village and through community integrated services ("*Posyandu*") held monthly in the village. Most women receive antenatal care through the *Pustu* and *Posyandu*, while most facility-based deliveries occur in the *Puskesmas*, or in the district hospital.

Each *Puskesmas* provides a monthly report to the district health office of the malaria smear positive cases including data from the *Pustu* and *Posyandu*. Based on this data the annual parasite incidence (API) is calculated as the annual number of positive malaria slides x 1000 / Total population for each village within the catchment area of each *Puskesmas*.

Malaria epidemiology

In Papua Indonesia, the study was conducted in Mimika district in southern Papua with its capital Timika. Modelling studies based on cross-sectional survey data suggested that in 2010 malaria transmission in most of this district is intermediate ($PfPR_{2-10}$ predicted prevalence of 5-40%).[1,2] In 2013, the annual incidence of parasitaemia was 450/1000, with *P. falciparum* and *P. vivax* respectively causing 60% and 40% of cases, without significant seasonal fluctuation.[1-3] In the same year, a community-based cross-sectional survey involving 2,830 individuals of all ages found that 37.7% had detectable malaria parasitaemia in the peripheral blood by microscopy or polymerase chain reaction (PCR), and 13.9% by microscopy alone. Approximately 99% of these infections were due to *P.falciparum* and *P.vivax* mono-infections, and the remaining due to *P.malariae*. Although most infections were asymptomatic, those with any parasitaemia, including sub-microscopic infections, were at significant risk of anaemia.[3]

In Sumba, the study was conducted in south-west Sumba district. Modelling studies based on cross-sectional survey data suggested that in 2010 malaria transmission in most of this district was low (PfPR 2–10 predicted prevalence of <5%), although some areas have intermediate levels of transmission defined as $PfPR_{2–10}$ 5-40%.[1,2] In a large cross-sectional survey in 2007 involving 8,870 individuals of all ages, the prevalence of malaria by expert microscopy (any species) was 6.8% in the rainy season and 4.9% in the dry season.[4] The seasonal variation in malaria prevalence reflected changes in the prevalence of *P.falciparum* infection, which was higher in the rainy vs dry season (4.9% vs 2.9%). There were no seasonal differences in the prevalence of *P.vivax* (~2.2%) and *P. malariae* (~0.1%).[4] In the same area, a screening study in 2012 found that approximately 3.2% of pregnant women were positive by RDT and 6.6% by PCR during routine scheduled antenatal clinic visits.[5]

Antimalarial drug resistance

In Papua, both *P.falciparum* and *P.vivax* parasites in Mimika district are highly resistant to sulfadoxine-pyrimethamine and chloroquine.[6] Prior to the switch to DP as first-line treatment in March 2006, at least 95% of patients with *P.falciparum* malaria treated with chloroquine mono-

therapy had recrudescent infections by day 28[7] and this was approximately 50% for the combination of chloroquine plus sulfadoxine-pyrimethamine.[7-9] High-grade chloroquine resistant *P.vivax* was first reported from Papua, Indonesia and Papua New Guinea in the late 1980, early 1990s.[8,10,11] A recent review of its impact[12] showed that in Papua, 60–90% of patients had recurrent malaria within 28 days[6,7,12] and 2% of patients infected with *P.vivax* treated with chloroquine monotherapy subsequently require admission to hospital.[6,13] In 2006 to 2008, 61.8% of *P.vivax* parasites carried a quadruple mutant genotype in the genes encoding for sulfadoxine-pyrimethamine resistance.[14] The clinical efficacy of DP against both *P.falciparum* and *P.vivax* in Papua remains excellent 9 years after extensive use since its introduction in March 2006.[15]

In Sumba, chloroquine resistant *P.falciparum* is widespread. In a survey in 2007, the prevalence of the 76T allele of the pfcrt gene was 89% and half of these parasites also carried pfmdr1 mutant alleles.[16] Although recent data is lacking, it is believed that the levels of resistance to sulfadoxine-pyrimethamine in Sumba is low, in contrast to Papua and other parts of Indonesia. In 2007, only 1% of *P.falciparum* isolates carried the double dhfr/dhps mutant genotype, and none the quadruple or quintuple mutant genotype.[16] A single therapeutic study in 2010 in patient with *P.vivax* malaria, showed that 98% of patients with *P.vivax* infections were successfully treated with sulfadoxine-pyrimethamine and only 3.3% of *P.vivax* parasites carried the quadruple mutant genotype reflecting sulfadoxine-pyrimethamine resistance, compared to 61.8% in Papua.[14] Over 99% *P. falciparum* isolates carried wild type K13 markers.[17]

Details of randomisation and public ceremony

Randomisation

The ANC clinics constituted the units of randomisation and were identified in advance by the lead investigators based in Indonesia (RA, JRR, DS) and their clinic identification number provided to the trial statistician (BF) based in the UK. A 1:1:1 allocation ratio was used. To minimize imbalances across treatment groups with respect to baseline malaria prevalence and risk factors for malaria, multivariate matching was used, based on the Government's annual parasite incidence (API) data for the two years preceding the study, geographical area (site [Sumba and Papua] and then sub-district within each site), and clinic size (prior annual number of new ANC attendees). In this way, the 78 eligible clinics was blocked into 26 sets of 3 matched clusters. A total of 20 randomisations were then generated using computer-generated random numbers, in which the three clinics in each triple were allocated to the three (arbitrarily labelled) groups A, B and C. For each randomisation, an imbalance score was calculated based on prevalence of positive RDTs in previous 12-months, geographical area and clinic size. The three randomisations with the "best" (smallest) imbalance score were then selected and each allocated a new computer-generated random number - the randomisation allocated the largest of these numbers was used in the study. A list with the dummy allocation for each cluster (as A, B, C) was then sent by the trial statistician based in the UK to the principal investigators in Indonesia.

Public ceremony

A public randomisation ceremony, organised by the principal investigators, was held in each of the two sites, attended by District health officials and village representatives. A district health official not involved in the study, first drew one of three identical looking opaque sealed envelopes which contained the dummy allocation from one box. The content of each envelope containing the dummy allocation for each cluster was then displayed to the audience. A second health official, then drew,

from the second box, one of three other identical looking opaque sealed envelopes containing the actual allocation. Prior to opening this second set of envelopes, the health official labelled each with dummy code from the first set of envelops (A B or C) after which they were opened to reveal the allocated assignment.

Definitions of morbidity endpoints

Birthweight data

The aim of the study was to measure birthweight within 24 hours after birth. Birth weights taken 24-48h hours (n=8, 0.4%), and 48-168 hours after delivery (n=2, 0.1%) were corrected for the physiological fall in birth weight in breastfed infants occurring in the first days following delivery[18,19] by a factor +2% and +4%, respectively to obtain the estimated weight at birth.[20,21] All analyses used corrected birthweight unless indicated otherwise. Low birth weight was defined as the corrected birthweight <2,500 gram.

Gestational age and preterm

Gestational age was assessed at enrolment using the date of the last menstrual period, fundal height, and at delivery using the modified Ballard score. If more than one gestational age measurement was available we used estimates in the following order of preference: Neonatal clinical exam within 96 hours of delivery (modified Ballard score), last menstrual period (if known), and fundal height at enrolment. Preterm was defined as a gestational age of less than 37 completed weeks.

Small for gestational age (SGA)

SGA was defined as birthweight below the tenth percentile of an external reference population for a given gestational age and sex. Small for gestational age (SGA) was defined as birthweight below the tenth percentile for a given gestational age and sex, using the new INTERGROWTH reference population,[22] which was also used to calculate the birthweight-for-gestational age Z-scores.

Congenital malaria

Any asexual malaria parasitaemia detected by microscopy, RDT or Loop-mediated isothermal amplification (LAMP) or PCR in cord blood or in the peripheral blood within 7 days of birth.

Assessment of compliance and tolerance to DP intake

A reminder phone text message or phone call was made on the day of the 2nd and 3rd dose of each course to participants who were given DP to take at home. If women were not contactable by phone, a field staff visited participant's homes to ensure the study drug was taken. On day 3, a home visit was made to all women who received DP to check compliance and whether the participant experienced any side effects.

Laboratory methods

mRDTs, malaria microscopy, haemoglobin assessment and histopathology

The mRDTs used targeted histidine-rich protein-2 (HRP2) and parasite lactate dehydrogenase (pLDH) (sensitivity to detect 200 parasites/µl: P.falciaprum=85%, P.vivax=74%) (First Response Malaria Ag pLDH/HRP2 Combo [I16FRC30], Premier Medical Corporation Ltd, India).[23] All mRDT-positive women (positive HRP2- or pLDH-bands) in any arms were treated with dihydroartemisinin-piperaquine. Women with a history of dihydroartemisinin-piperaquine intake in the previous 4 weeks received quinine-clindamycin.

Malaria smears were not used for point of care. All smears were read first by an expert microscopist on site who was blind to the mRDT results. All positives and a random selection of 10% of negative smear results (randomly selected) were read by the senior expert microscopist at the Eijkman Institute, Jakarta who was blinded to the results of the first reading. If one of the two smears were declared positive, LAMP/PCR findings were used. Malaria infection was defined as the presence of asexual *Plasmodium* parasites (any species) in a thick blood smear. Parasite densities were counted against 300 white blood cells and expressed per 8,000 parasites per microlitre. Smears were declared negative if no parasites were detected after examining 200 high power fields. Thin smear was used to identify malaria species (PCR confirmed species were subsequently used in the analysis). Placental incision smears (thick and thin) were read and parasite density calculated similar to maternal peripheral smears. Haemoglobin levels were determined using portable HemoCue Hb 201+ (HemoCue AB, Ängelholm Sweden) machines following manufacture instructions. Malaria rapid diagnostic test (RDT) was performed as per the manufacturer's instruction. Tissue samples for placental histopathology were collected from the maternal side of the placenta and fixed with 10% neutral buffered formalin and then processed, stained with hemotoxin-eosin, and examined under standard light and polarized microscope following standard procedures.[24] Histopathological slides were read in duplicate by two independent readers who were blinded to the placental and maternal smear results, and any discrepant results were resolved by one of the investigators (RA).

Molecular methods

DNA extraction

Dried blood spots (DBSs) for LAMP and PCR assays were collected on Whatman's #3 filter paper, air dried and placed in individual plastic bags with desiccant and stored at room temperature. From these spots, genomic DNA (gDNA) was extracted using chelex-100 ion exchanger (Biorad Laboratories, Hercules, CA). Briefly, 6 mm filter paper disc punches were incubated in 0.5% saponin in PBS overnight, centrifuged for 10 minutes at 12000 rpm, supernatant discarded, washed in PBS, centrifuged for 5 minutes at 12000 rpm and the supernatant discarded (this procedure was repeated 3 times). The sample was then heated at 100 ^{o}C in 150 µl of 20% Chelex 100-Ion Exchanger for 10 minutes and centrifuged for 10 minutes at 12000 rpm. The resultant 100 µl supernatant was stored at -20 ^{o}C.

Loop mediated isothermal amplification (LAMP)

LAMP assays were conducted at the Eijkman institute in Jakarta using to the Eiken Loopamp™ MALARIA Pan Detection kit procedures (Eiken Chemical Company, Japan). 15 µL of DNA plus 15 µL of water was added to the malaria *Pan* reaction tube with one negative and one positive control included in each 16 reactions; primers, buffers and enzymes were reconstituted by inverting the samples in the lid of the reaction tubes, tubes were briefly spun and incubated at 65 °C for 40 minutes before polymerase inactivation at 80 °C for 5 minutes.[25,26] The limit of detection of LAMP assays is ~1 parasite/ µl.

Real-time PCR (quantitative PCR)

A multiplex real-time PCR was used to simultaneously to detect the four main species of *Plasmodium*: *falciparum*, *vivax, ovale* and *malariae.* Primers and probes were used at identical concentrations as previously published [27,28] in total reaction volume of 10 µL. Each reaction contained 2 µL of gDNA, 1x Quantifast Pathogen PCR Master Mix and primer/probe concentrations as outlined in eTable 1. Amplification and real-time measurements were carried out using the Rotor-

Gene Q 5plex HRM Platform (Qiagen, Hilden Germany) in a 72-optical tube format. The thermal cycling profile was as follows: 95°C for 10 minutes, followed by 95°C for 15 seconds and then 60°C for 60 seconds for 38 cycles. Cycle threshold (Ct) values were calculated and analysed with the Rotorgene Q series software version 1.7 (Qiagen Inc, Valencia, CA, USA). Positive DNA controls for each species (provided by Malaria Reference Laboratory, Public Health England) and non-template controls (NTCs) were also included. The limit of detection of each of these primer/probe sets are between 0.1-1 parasite/ µl.

eTable 1: Primer/probe concentrations used in the real-time PCR (qPCR)

	Sequence 5'-3'	Reaction Concentration (nM)	Source
Pan reverse	AACCCAAAGACTTTGATTTCTCATAA	200	Eurofins
MAL FP	CCGACTAGGTGTTGGATGATAGAGTAAA	50	Eurofins
MAL probe	ATTO700-CTATCTAAAAGAAACACTCAT-MGBEDQ	80	Eurogentec
OVA FP	CCGACTAGGTTTTGGATGAAAGATTTTT	50	Eurofins
OVA Probe	Cy5-CGAAAGGAATTTTCTTATT-MGBEDQ	80	Eurogentec
FAL FP	ATTGCTTTTGAGAGGTTTTGTTACTTT	400	Eurofins
FAL RP	GCTGTAGTATTCAAACACAATGAACTCAA	400	Eurofins
FAL probe	FAM-CATAACAGACGGGTAGTCAT-MGBQ	200	Thermo
VIV FP	GCAACGCTTCTAGCTTAATCCAC	400	Eurofins
VIV RP	CAAGCCGAAGCAAAGAAAGTCC	400	Eurofins
VIV probe	VIC-ACTTTGTGCGCATTTTGCTA-MGBQ	200	Thermo

Nested PCR (nPCR)

Nested PCR was performed using previously described primers and reaction conditions.[29] The limit of detection of this assay is estimated to be 6-10 parasites/ µl.

Definition of LAMP/PCR positivity

All samples were first tested for malaria using Loop mediated isothermal amplification (LAMP) in the laboratories of the Eijkman Institute in Jakarta Indonesia. The multiplex real-time PCR was then used to determine the species. In addition, a random sample of 5% of the LAMP negatives was also tested using qPCR. Any LAMP-qPCR discordant samples were tested using nested PCR. The following algorithm was used to define LAMP/PCR positivity (see Figure 1 Definition of PCR/LAMP positivity).

Definition of PCR/LAMP positivity

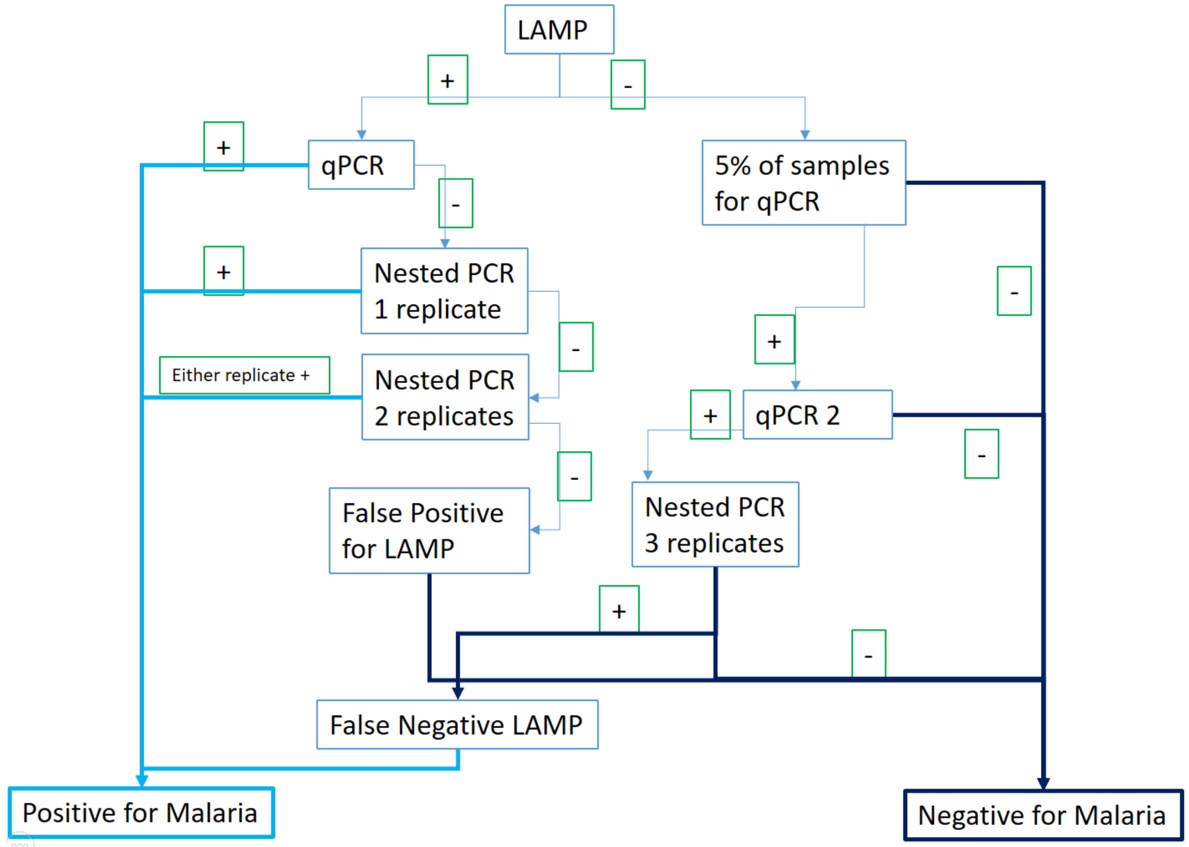

Sample size and power calculations

Original sample size calculations

The trial was originally designed to detect a 50% or greater reduction in malaria infection at delivery, from 10.0% in the SST group to 5.0% in any of the interventions group with 90% power, 2-sided alpha of 0.025, and an assumed ICC of 0.002, and accounting for a 13% efficiency loss due to varying cluster sizes, and 20% loss to follow-up. This required 3,198 women (1,066 per arm) from 78 clusters (26 per arm, 7 in Papua and 19 in Sumba) with an average of 41 women.

Revised calculations following the interim power and sample size re-estimation

Following approximately 14 months of recruitment, by which time 989 women had been recruited in the Sumba site and 476 from Papua (total 1,465), the primary Research Ethics Committee in Indonesia advised to stop recruitment in Sumba because of the low pooled event rate of the primary outcome (approximately 3.4%) and to continue recruiting in the Papua site with more moderate transmission (pooled event rate approximately 20%). An interim sample re-estimation was then conducted in a blinded manner using the observed pooled event rate in each site across the 3 arms and the observed ICC values.

The pooled event rate of the primary endpoint was then used to estimate the frequency in the control arm (SST), by assuming a 50% reduction in the IPT arm relative to the control arm (SST) (RR=0.50, as per protocol), and assuming no reduction in the IST arm (RR=1.0) (based on new data from recently completed IST trials in Kenya and Malawi).[30,31] For example, if the observed pooled prevalence was 10%, then this was assumed to be a combination of SST=12%, IST=12% and IPT=6%

(0.5 x 12%), and an equal distribution of women by arm (1:1:1 allocation). Similarly, for a prevalence of 20% this was assumed to be the summary of 24% in the SST and IST arms and 12% in the IPT arm.

Power and sample size calculations where then conducted using NCSS/PASS to estimate the required extension that would provide at least 80% power to detect a 50% reduction in the primary endpoint across the two sites pooled using the 'metapow' command in Stata, and 80% to 90% power to detect a similar reduction in Papua alone, while allowing for 13% loss in efficiency due to cluster size variation and 20% loss to follow-up.

This suggested that in Papua a total of 1,290 women overall and 903 completers (301/arm, 43 in 7 clusters) were required on top of the 989 women recruited in Sumba (i.e. 2,279) to achieve at least 80% power overall across the two study sites pooled (alpha 0.0167, ICC=0.005). This sample size was also estimated to provide 87% power to detect a 50% reduction from 24% to 12% in Papua alone and also had 80% power to detect a 50% difference if the prevalence of malaria was only 21% or if the ICC was 0.01 instead of 0.005 (NCSS/PASS). Analysis

Cardiac monitoring

Electrocardiography was performed in a subgroup of women in the IPT-DP arm to determine whether previously documented transient QTc prolongation associated with DP increases in magnitude with subsequent courses. The study was conducted in the Papua site Indonesia. Written informed consent was obtained from all women.

Pregnant women enrolled in the IPT arm of the main trial willing to complete the study schedule were eligible. Following enrolment, women had an ECG measured at baseline and then again 4-6 hours after taking the 3rd dose of each course of DP. This timing was chosen as it represents the time of the expected maximum concentration of DP (anticipated Tmax), which has been shown to correlate with the expected maximal prolongation of the QT interval.[32] With each subsequent monthly treatment course, the ECG was repeated 4-6 hours after the 3rd dose. All ECGs were done in triplicate 30 to 60 seconds apart. ECGs were read on site, and again by a cardiologist at Cardiabase, the Banook Group, France and results reported back to the study team in Indonesia. SAEs (QTcF > 480 ms or delta QTcF from baseline >60 msec) were reported in an expedited manner. Any woman with a QTcF >480 ms or delta QTcF from baseline >60 msec were withdrawn from receiving additional doses of DP, but followed per study protocol.

It was estimated that 33 women were required to allow detection of a 20ms difference in QTc from baseline following exposure to DP, assuming an estimated standard deviation of 30 ms, with 90% power, at a significance level 0.05 using a two-sided one-sample t-test and allowing for 20% loss to follow-up.

The primary endpoint was the change in QTc from baseline (hour 0; i.e. prior to the first course of DP) to 4-6 hours following receipt of the third dose with each course of DP.

The mean of the triplicate ECGs measurements taken 30 to 60 seconds apart were used for analysis. The primary analysis was based on Fridericia's method to obtain heart rate corrected QTc intervals (observed QT interval divided by cube root of RR interval, in seconds [$QT / (RR)^{0.33}$]). A sensitivity analysis was conducted using the same analytical approach but now using the Bazett's method to obtain QTc intervals (observed QT interval divided by the square root of RR interval, in seconds [QT /

$(RR)^{0.5}$]). QTcB value using Bazett's correction were not considered for clinical care, but were also calculated for data analysis.

Other sub studies

As indicated in the original trial protocol, the study included a second main objective "To determine the acceptability, feasibility and cost effectiveness of SST, IST and IPT alongside the randomised control trial." The results of the acceptability and feasibility studies have been published previously.[33-35] The cost-effectiveness analysis is ongoing and will also be published elsewhere.

eResults

eTables

eTable 2: Follow-up visits schedule (intention to treat population)

	Sumba			Papua			Overall		
	IST (n=359)	IPT (n=293)	SST (n=337)	IST (n=495)	IPT (n=388)	SST (n=407)	IST (n=854)	IPT (n=681)	SST (n=744)
Possible No. of scheduled visits adjusted for early delivery, including enrolment, excluding delivery[a,b] No. (%)									
1	3 (0.8%)	7 (2.4%)	3 (0.9%)	12 (2.4%)	6 (1.5%)	15 (3.7%)	15 (1.8%)	13 (1.9%)	18 (2.4%)
2	43 (12.0%)	40 (13.7%)	43 (12.8%)	50 (10.1%)	42 (10.8%)	67 (16.5%)	93 (10.9%)	82 (12.0%)	110 (14.8%)
3	105 (29.3%)	94 (32.1%)	116 (34.4%)	106 (21.4%)	102 (26.3%)	84 (20.6%)	211 (24.7%)	196 (28.8%)	200 (26.9%)
4	129 (35.9%)	80 (27.3%)	99 (29.4%)	129 (26.1%)	126 (32.5%)	125 (30.7%)	258 (30.2%)	206 (30.2%)	224 (30.1%)
5	62 (17.3%)	55 (18.8%)	57 (16.9%)	141 (28.5%)	91 (23.5%)	92 (22.6%)	203 (23.8%)	146 (21.4%)	149 (20.0%)
6	17 (4.7%)	17 (5.8%)	19 (5.6%)	57 (11.5%)	21 (5.4%)	24 (5.9%)	74 (8.7%)	38 (5.6%)	43 (5.8%)
Total	1332	1066	1232	1993	1481	1505	3325	2547	2737
Achieved number of scheduled visits, including enrolment, excluding delivery, No. (%)									
1	21 (5.8%)	17 (5.8%)	13 (3.9%)	47 (9.5%)	69 (17.8%)	53 (13.0%)	68 (8.0%)	86 (12.6%)	66 (8.9%)
2	77 (21.4%)	63 (21.5%)	82 (24.3%)	86 (17.4%)	91 (23.5%)	87 (21.4%)	163 (19.1%)	154 (22.6%)	169 (22.7%)
3	96 (26.7%)	96 (32.8%)	111 (32.9%)	113 (22.8%)	97 (25.0%)	87 (21.4%)	209 (24.5%)	193 (28.3%)	198 (26.6%)
4	115 (32.0%)	67 (22.9%)	80 (23.7%)	132 (26.7%)	92 (23.7%)	106 (26.0%)	247 (28.9%)	159 (23.3%)	186 (25.0%)
5	41 (11.4%)	39 (13.3%)	42 (12.5%)	84 (17.0%)	32 (8.2%)	58 (14.3%)	125 (14.6%)	71 (10.4%)	100 (13.4%)
6	9 (2.5%)	11 (3.8%)	9 (2.7%)	33 (6.7%)	7 (1.8%)	16 (3.9%)	42 (4.9%)	18 (2.6%)	25 (3.4%)
Total	1182	960	1094	1704	1112	1298	2886	2072	2392
Number of DP courses received, No. (%)									
0	357 (99.4%)	0 (0.0%)	335 (99.4%)	460 (92.9%)	3 (0.8%)	366 (89.9%)	817 (95.6%)	3 (0.4%)	701 (94.2%)
1	2 (0.6%)	17 (5.8%)	2 (0.6%)	31 (6.3%)	70 (18.0%)	37 (9.1%)	33 (3.9%)	87 (12.8%)	39 (5.2%)
2	0 (0.0%)	63 (21.5%)	0 (0.0%)	4 (0.8%)	90 (23.2%)	3 (0.7%)	4 (0.5%)	153 (22.5%)	3 (0.4%)
3	0 (0.0%)	96 (32.8%)	0 (0.0%)	0 (0.0%)	97 (25.0%)	1 (0.3%)	0 (0.0%)	193 (28.3%)	1 (0.1%)
4	0 (0.0%)	67 (22.9%)	0 (0.0%)	0 (0.0%)	90 (23.2%)	0 (0.0%)	0 (0.0%)	157 (23.1%)	0 (0.0%)

	Sumba			Papua			Overall		
	IST (n=359)	IPT (n=293)	SST (n=337)	IST (n=495)	IPT (n=388)	SST (n=407)	IST (n=854)	IPT (n=681)	SST (n=744)
5	0 (0.0%)	39 (13.3%)	0 (0.0%)	0 (0.0%)	31 (8.0%)	0 (0.0%)	0 (0.0%)	70 (10.3%)	0 (0.0%)
6	0 (0.0%)	11 (3.8%)	0 (0.0%)	0 (0.0%)	7 (1.8%)	0 (0.0%)	0 (0.0%)	18 (2.6%)	0 (0.0%)
Total	2	960	2	39	1098	46	41	2058	48
Person days contributed till delivery or till lost to follow-up, median (IQR)									
	92 (64-113)	90 (64-117)	91 (64-120)	105 (69-131)	87 (45.5-115)	97 (57-113)	98 (65-125)	88 (57-116)	94 (64-126)

a. The number of monthly scheduled visits was dependent on the gestational age at enrolment.
b. Adjusted for early delivery (i.e. excludes all planned antenatal visits that could not have occurred because the pregnancy ended before that scheduled date)
IQR=interquartile range

eTable 3: Proportion of women with missing data for the primary outcome by treatment arm

	no/No (%) of patients with missing primary endpoint			Risk Ratio (95% CI), p-value		
Outcome	IPT	IST	SST	IPT vs SST	IST vs SST	IPT vs IST
Primary endpoint (malaria infection at delivery)						
Overall	153/681 (22.5)	141/854 (16.5)	111/744 (14.9)	1.36 (0.95, 1.94), 0.09	1.27 (0.89, 1.80), 0.18	1.04 (0.75-1.47), 0.79
Sumba	37/293 (12.6)	74/359 (20.6)	47/337 (13.9)	0.90 (0.55, 1.49), 0.69	1.57 (0.97-2.55), 0.07	0.58 (0.37-0.91), 0.0167
Papua	116/388 (29.9)	67/495 (13.5)	64/407 (15.7)	1.90 (1.32-2.72), 0.0005	0.87 (0.58, 1.32), 0.53	2.18 (1.62-2.95), <0.0001

IPT=intermittent preventive treatment during pregnancy with dihydroartemisinin–piperaquine. IST=intermittent screening and treatment during pregnancy with dihydroartemisinin–piperaquine. SST=Single screening and treatment during pregnancy with dihydroartemisinin–piperaquine.

eTable 4: Results from GEE model analysis of the primary outcome with and without breaking the matching, overall and by site

Site	Comparisons	Result with breaking the matching (primary analysis, main text)	Result without breaking the matching*
Overall	IPT vs. SST	0.59 (0.42, 0.83), p=0.0022	0.65 (0.44, 0.95), p=0.0264
	IST vs. SST	0.56 (0.40, 0.77), p=0.0005	0.57 (0.39, 0.83), p=0.0037
	IPT vs. IST	1.06 (0.73, 1.54), p=0.7657	1.14 (0.85, 1.54), p=0.3837
Sumba	IPT vs. SST	0.70 (0.44, 1.09), p=0.1151	0.71 (0.45, 1.13), p=0.1482
	IST vs. SST	0.43 (0.24, 0.78), p=0.0049	0.55 (0.33, 0.91), p=0.0189
	IPT vs. IST	1.61 (0.85, 3.07), p=0.1467	1.30 (0.91, 1.88), p=0.1531
Papua	IPT vs. SST	0.50 (0.32, 0.79), p=0.0026	0.51 (0.27, 0.94), p=0.0316
	IST vs. SST	0.63 (0.47, 0.85), p=0.0025	0.64 (0.47, 0.87), p=0.0050
	IPT vs. IST	0.79 (0.52, 1.20), p=0.2663	0.79 (0.55, 1.15), p=0.2205

* Primary endpoint was first summarized at cluster level to generate 78 proportions of patients with a primary endpoint, which were then compared using GEE model after taking matching factor into account.[36]

eTable 5: Intra-cluster correlation coefficient (ICC) for primary endpoint

	SST arm only	All arms pooled
Overall:	0.0167	0.0244
Sumba:	-0.00044*	0.0215
Papua	0.0123	0.0167

*Set to zero

eTable 6: Site-treatment interaction P-values for malaria at the time of delivery (primary outcome) and key secondary outcomes

		ITT		PPP	
Groups	Outcome	Crude	Adjusted	Crude	Adjusted
IPT vs SST	Maternal peripheral or placental Plasmodium infection at delivery (primary outcome)	0.31	0.31	0.77	0.85
	Maternal Plasmodium infection during pregnancy (LAMP/PCR/smear/RDT) (cumulative risk)	<.0001	<.0001	0.0115	0.0184
	Maternal clinical malaria during pregnancy (cumulative risk)	†	†	†	†
	Maternal peripheral patent Plasmodium infection at delivery	†	†	†	†
	Maternal peripheral sub-patent Plasmodium infection at delivery	0.46	0.90	0.77	0.83
	Placental Plasmodium infection (LAMP/PCR/smear/RDT/histology [active+past])	0.06	0.06	0.38	0.40
	Maternal Hb<9 g/dl at delivery	0.14	0.33	0.09	0.20
	Fetal anaemia (cord Hb<10 g/dL)	0.79	0.85	0.70	0.94
	Small for gestational age (<10th percentile INTERGROWTH)	0.47	0.81	0.17	0.41
	Low birth weight (<2,500g)	0.0150	0.0063	0.0091	0.0056
	Preterm birth (<37 weeks)	0.17	0.14	0.46	0.41
	Fetal loss (spontaneous abortion or stillbirth)	0.42	0.61	0.29	0.47
	Neonatal death (<28 days)	†	†	†	†
	Adverse pregnancy outcome (fetal loss, LBW/SGA/PT)	0.0457	0.09	0.05	0.08
IST vs SST	Maternal peripheral or placental Plasmodium infection at delivery (primary outcome)	0.26	0.06	0.11	0.0381
	Maternal Plasmodium infection during pregnancy (LAMP/PCR/smear/RDT) (cumulative risk)	0.17	0.06	0.09	0.43
	Maternal clinical malaria during pregnancy (cumulative risk)	†	†	†	†
	Maternal peripheral patent Plasmodium infection at delivery	0.76	0.76	0.90	0.90
	Maternal peripheral sub-patent Plasmodium infection at delivery	0.0043	0.0004	0.0038	0.0006
	Placental Plasmodium infection (LAMP/PCR/smear/RDT/histology [active+past])	0.91	0.86	0.97	0.71
	Maternal Hb<9 g/dl at delivery	0.12	0.31	0.0202	0.28
	Fetal anaemia (cord Hb<10 g/dL)	0.72	0.95	0.73	0.36
	Small for gestational age (<10th percentile INTERGROWTH)	0.49	0.79	0.34	0.75
	Low birth weight (<2,500g)	0.16	0.05	0.0474	0.0227
	Preterm birth (<37 weeks)	0.0032	0.0072	0.0403	0.07
	Fetal loss (spontaneous abortion or stillbirth)	0.64	0.77	0.60	0.81
	Neonatal death (<28 days)	†	†	†	†
	Adverse pregnancy outcome (fetal loss, LBW/SGA/PT)	0.07	0.25	0.10	0.29
IPT vs IST	Maternal peripheral or placental Plasmodium infection at delivery (primary outcome)	0.07	0.0154	0.12	0.08
	Maternal Plasmodium infection during pregnancy (LAMP/PCR/smear/RDT) (cumulative risk)	0.0018	0.0091	0.20	0.08

Maternal clinical malaria during pregnancy (cumulative risk)	†	†	†	†
Maternal peripheral patent Plasmodium infection at delivery	†	†	†	†
Maternal peripheral sub-patent Plasmodium infection at delivery	0.0021	0.0025	0.0057	0.0106
Placental Plasmodium infection (LAMP/PCR/smear/RDT/histology [active+past])	0.06	0.0299	0.35	0.28
Maternal Hb<9 g/dl at delivery	0.81	0.90	0.93	0.67
Fetal anaemia (cord Hb<10 g/dL)	0.98	0.90	0.53	0.45
Small for gestational age (<10th percentile INTERGROWTH)	0.97	0.98	0.70	0.63
Low birth weight (<2,500g)	0.44	0.58	0.46	0.74
Preterm birth (<37 weeks)	0.28	0.43	0.37	0.50
Fetal loss (spontaneous abortion or stillbirth)	0.68	0.34	0.43	0.19
Neonatal death (<28 days)	†	†	†	†
Adverse pregnancy outcome (fetal loss, LBW/SGA/PT)	0.99	0.73	0.71	0.48

ITT=intention to treat population, PPP=per protocol population, Crude=unadjusted for co-variates, IST=intermittent screening and treatment during pregnancy with dihydroartemisinin–piperaquine. IPT=intermittent preventive treatment during pregnancy with dihydroartemisinin–piperaquine. SST=Single screening and treatment during pregnancy with dihydroartemisinin–piperaquine; py=person years. P-values for interaction terms were obtained post-hoc using the Altman-Bland method.[37]
* Outcomes represents binary outcome except for the data for the incidence per 100 person-years. † The p-value for interaction could not be computed because the relative risk in at least one of the study sites could not be computed because of zero events in at least one of the study arms.

eTable 7: Site-treatment interaction P-values for secondary outcomes related to malaria at the time of delivery

Groups	Outcome	ITT		PPP	
		Crude	Adjusted	Crude	Adjusted
IPT vs SST	Maternal peripheral Plasmodium infection at delivery (RDT)	†	†	†	†
	Maternal peripheral Plasmodium infection at delivery (smear)	†	†	†	†
	Maternal peripheral Plasmodium infection at delivery (LAMP/PCR)	0.13	0.28	0.30	0.54
	Maternal peripheral Plasmodium infection at delivery (LAMP/PCR/smear/RDT)	0.12	0.18	0.24	0.36
	Maternal peripheral *P.falciparum* infection at delivery (PCR confirmed)	0.18	0.29	0.38	0.54
	Maternal peripheral *P.vivax* mono-infection at delivery (PCR confirmed)	0.37	0.58	0.25	0.23
	Maternal peripheral patent Plasmodium infection at delivery	†	†	†	†
	Maternal peripheral sub-patent Plasmodium infection at delivery	0.46	0.90	0.77	0.83
	Maternal peripheral or placental patent Plasmodium infection at delivery	†	†	†	†
	Maternal peripheral or placental sub-patent Plasmodium infection at delivery	0.62	0.90	0.76	0.67
IST vs SST	Maternal peripheral Plasmodium infection at delivery (RDT)	†	†	†	†
	Maternal peripheral Plasmodium infection at delivery (smear)	0.20	0.19	0.29	0.29
	Maternal peripheral Plasmodium infection at delivery (LAMP/PCR)	0.0056	0.0009	0.0059	0.0050
	Maternal peripheral Plasmodium infection at delivery (LAMP/PCR/smear/RDT)	0.13	0.0152	0.07	0.0194
	Maternal peripheral *P.falciparum* infection at delivery (PCR confirmed)	0.22	0.0135	0.14	0.0338
	Maternal peripheral *P.vivax* mono-infection at delivery (PCR confirmed)	0.95	0.71	0.54	0.87
	Maternal peripheral patent Plasmodium infection at delivery	0.76	0.76	0.90	0.90
	Maternal peripheral sub-patent Plasmodium infection at delivery	0.0043	0.0004	0.0038	0.0006
	Maternal peripheral or placental patent Plasmodium infection at delivery	0.66	0.66	0.90	0.90
	Maternal peripheral or placental sub-patent Plasmodium infection at delivery	0.08	0.0237	0.0314	0.0149
IPT vs IST	Maternal peripheral Plasmodium infection at delivery (RDT)	†	†	†	†
	Maternal peripheral Plasmodium infection at delivery (smear)	†	†	†	†
	Maternal peripheral Plasmodium infection at delivery (LAMP/PCR)	0.0003	0.0005	0.0019	0.0050
	Maternal peripheral Plasmodium infection at delivery (LAMP/PCR/smear/RDT)	0.0084	0.0023	0.0172	0.0120
	Maternal peripheral *P.falciparum* infection at delivery (PCR confirmed)	0.0280	0.0038	0.05	0.0250
	Maternal peripheral *P.vivax* mono-infection at delivery (PCR confirmed)	0.45	0.50	0.16	0.27
	Maternal peripheral patent Plasmodium infection at delivery	†	†	†	†
	Maternal peripheral sub-patent Plasmodium infection at delivery	0.0021	0.0025	0.0057	0.0106
	Maternal peripheral or placental patent Plasmodium infection at delivery	†	†	†	†
	Maternal peripheral or placental sub-patent Plasmodium infection at delivery	0.06	0.0403	0.12	0.12

ITT=intention to treat population, PPP=per protocol population, Crude=unadjusted for co-variates, IST=intermittent screening and treatment during pregnancy with dihydroartemisinin–piperaquine. IPT=intermittent preventive treatment during pregnancy with dihydroartemisinin–piperaquine. SST=Single screening and treatment during pregnancy with dihydroartemisinin–piperaquine; py=person years. P-values for interaction terms were obtained post-hoc using the Altman-Bland method.[37]
* Outcomes represents binary outcome except for the data for the incidence per 100 person-years. † The p-value for interaction could not be computed because the relative risk in at least one of the study sites could not be computed because of zero events in at least one of the study arms.

eTable 8: Site-treatment interaction P-values for secondary outcomes related to placental malaria

		ITT		PPP	
Groups	Outcome	Crude	Adjusted	Crude	Adjusted
IPT vs SST	Placental Plasmodium infection (LAMP/PCR/smear/RDT/histology [active])	0.47	0.69	0.72	0.58
	Placental Plasmodium infection (LAMP/PCR/smear/RDT/histology [active+past])	0.06	0.06	0.38	0.40
	Placental malaria (smear)	†	†	†	†
	Placental malaria (RDT:HRP2 or pLDH)	†	†	†	†
	Placental malaria (LAMP/PCR)	0.59	0.90	0.72	0.60
	Placental malaria (histology: active-any)	0.66	0.72	0.16	0.18
	Placental malaria (histology: past)	0.0035	0.0014	0.0085	0.0039
	Placental malaria (histology: active-acute)	0.27	0.24	0.0276	0.0304
	Placental malaria (histology: active-chronic)	0.50	0.48	†	†
	Placental malaria (histology: active or past)	0.0121	0.0109	0.36	0.28
IST vs SST	Placental Plasmodium infection (LAMP/PCR/smear/RDT/histology [active])	0.73	0.79	0.85	0.74
	Placental Plasmodium infection (LAMP/PCR/smear/RDT/histology [active+past])	0.91	0.86	0.97	0.71
	Placental malaria (smear)	†	†	†	†
	Placental malaria (RDT:HRP2 or pLDH)	†	†	†	†
	Placental malaria (LAMP/PCR)	0.95	0.37	0.83	0.77
	Placental malaria (histology: active-any)	0.96	0.79	0.15	0.16
	Placental malaria (histology: past)	0.27	0.45	0.26	0.46
	Placental malaria (histology: active-acute)	0.63	0.29	0.23	0.30
	Placental malaria (histology: active-chronic)	0.79	0.83	0.54	0.73
	Placental malaria (histology: active or past)	0.78	0.76	0.62	0.27
IPT vs IST	Placental Plasmodium infection (LAMP/PCR/smear/RDT/histology [active])	0.68	0.53	0.87	0.83
	Placental Plasmodium infection (LAMP/PCR/smear/RDT/histology [active+past])	0.06	0.0299	0.35	0.28
	Placental malaria (smear)	†	†	†	†
	Placental malaria (RDT:HRP2 or pLDH)	†	†	†	†
	Placental malaria (LAMP/PCR)	0.61	0.24	0.90	0.87
	Placental malaria (histology: active-any)	0.71	0.94	0.74	0.77
	Placental malaria (histology: past)	0.0029	<.0001	0.0194	0.0013
	Placental malaria (histology: active-acute)	0.17	0.0358	0.36	0.20
	Placental malaria (histology: active-chronic)	0.43	0.44	†	†
	Placental malaria (histology: active or past)	0.0179	0.0201	0.19	0.06

ITT=intention to treat population, PPP=per protocol population, Crude=unadjusted for co-variates, IST=intermittent screening and treatment during pregnancy with dihydroartemisinin–piperaquine. IPT=intermittent preventive treatment during pregnancy with dihydroartemisinin–piperaquine. SST=Single screening and treatment during pregnancy with dihydroartemisinin–piperaquine; py=person years. P-values for interaction terms were obtained post-hoc using the Altman-Bland method.[37]
* Outcomes represents binary outcome except for the data for the incidence per 100 person-years. † The p-value for interaction could not be computed because the relative risk in at least one of the study sites could not be computed because of zero events in at least one of the study arms.

eTable 9: Site-treatment interaction P-values for malaria outcomes and non-malaria sick visits during pregnancy

		ITT		PPP	
Groups	Outcome	Crude	Adjusted	Crude	Adjusted
IPT vs SST	Maternal Plasmodium infection during pregnancy (LAMP/PCR/smear/RDT) (cumulative risk)	<.0001	<.0001	0.0115	0.0184
	Maternal Plasmodium infection during pregnancy (LAMP/PCR/smear/RDT) (incidence/100 py)*	<.0001	<.0001	0.0024	0.0026
	Maternal Plasmodium infection during pregnancy (fever-RDT) (cumulative risk)	†	†	†	†
	Maternal Plasmodium infection during pregnancy (fever-RDT) (incidence/100 py)*	†	†	†	†
	Maternal Plasmodium infection during pregnancy (smear) (cumulative risk)	†	†	†	†
	Maternal Plasmodium infection during pregnancy (smear) (incidence/100 py)*	†	†	†	†
	Maternal Plasmodium infection during pregnancy (LAMP/PCR) (cumulative risk)	<.0001	<.0001	0.0002	0.0002
	Maternal Plasmodium infection during pregnancy (LAMP/PCR) (incidence/100 py)*	<.0001	<.0001	0.0001	0.0003
	Maternal patent Plasmodium infection during pregnancy (cumulative risk)	†	†	†	†
	Maternal patent Plasmodium infection during pregnancy (incidence/100 py)*	†	†	†	†
	Maternal sub-patent Plasmodium infection during pregnancy (cumulative risk)	0.0002	0.0002	0.0013	0.0005
	Maternal sub-patent Plasmodium infection during pregnancy (incidence/100 py)*	0.0006	0.0006	0.0016	0.0019
	Maternal clinical malaria during pregnancy (cumulative risk)	†	†	†	†
	Maternal clinical malaria during pregnancy (incidence/100py)*	†	†	†	†
	Maternal non-malaria sick visits during pregnancy (cumulative risk)	0.26	0.16	0.75	0.80
	Maternal non-malaria sick visits during pregnancy (incidence/100py)*	0.23	0.11	0.64	0.56
	Maternal all-cause sick visits during pregnancy (cumulative risk)	0.11	0.08	0.47	0.49
	Maternal all-cause sick visits during pregnancy (incidence/100py)*	0.09	0.0475	0.40	0.44
IST vs SST	Maternal Plasmodium infection during pregnancy (LAMP/PCR/smear/RDT) (cumulative risk)	0.17	0.06	0.09	0.43
	Maternal Plasmodium infection during pregnancy (LAMP/PCR/smear/RDT) (incidence/100 py)*	0.11	0.41	0.08	0.45
	Maternal Plasmodium infection during pregnancy (fever-RDT) (cumulative risk)	†	†	†	†
	Maternal Plasmodium infection during pregnancy (fever-RDT) (incidence/100 py)*	†	†	†	†
	Maternal Plasmodium infection during pregnancy (smear) (cumulative risk)	0.13	0.13	0.21	0.21
	Maternal Plasmodium infection during pregnancy (smear) (incidence/100 py)*	0.12	0.19	0.19	0.54
	Maternal Plasmodium infection during pregnancy (LAMP/PCR) (cumulative risk)	0.08	0.0212	0.0379	0.15
	Maternal Plasmodium infection during pregnancy (LAMP/PCR) (incidence/100 py)*	0.06	0.23	0.0481	0.28
	Maternal patent Plasmodium infection during pregnancy (cumulative risk)	0.07	0.0408	0.17	0.43
	Maternal patent Plasmodium infection during pregnancy (incidence/100 py)*	0.05	0.11	0.15	0.47
	Maternal sub-patent Plasmodium infection during pregnancy (cumulative risk)	0.17	0.20	0.0447	0.0157
	Maternal sub-patent Plasmodium infection during pregnancy (incidence/100 py)*	0.20	0.35	0.10	0.28
	Maternal clinical malaria during pregnancy (cumulative risk)	†	†	†	†
	Maternal clinical malaria during pregnancy (incidence/100py)*	†	†	†	†

	Maternal non-malaria sick visits during pregnancy (cumulative risk)	0.07	0.0070	0.23	0.13
	Maternal non-malaria sick visits during pregnancy (incidence/100py)*	0.0475	0.0012	0.16	0.09
	Maternal all-cause sick visits during pregnancy (cumulative risk)	0.0246	0.0023	0.18	0.07
	Maternal all-cause sick visits during pregnancy (incidence/100py)*	0.0086	0.0028	0.09	0.0441
IPT vs IST	Maternal Plasmodium infection during pregnancy (LAMP/PCR/smear/RDT) (cumulative risk)	0.0018	0.0091	0.20	0.08
	Maternal Plasmodium infection during pregnancy (LAMP/PCR/smear/RDT) (incidence/100 py)*	0.0025	0.0003	0.06	0.0137
	Maternal Plasmodium infection during pregnancy (fever-RDT) (cumulative risk)	†	†	†	†
	Maternal Plasmodium infection during pregnancy (fever-RDT) (incidence/100 py)*	†	†	†	†
	Maternal Plasmodium infection during pregnancy (smear) (cumulative risk)	†	†	†	†
	Maternal Plasmodium infection during pregnancy (smear) (incidence/100 py)*	†	†	†	†
	Maternal Plasmodium infection during pregnancy (LAMP/PCR) (cumulative risk)	0.0019	0.0102	0.0461	0.0219
	Maternal Plasmodium infection during pregnancy (LAMP/PCR) (incidence/100 py)*	0.0141	0.0067	0.0287	0.0105
	Maternal patent Plasmodium infection during pregnancy (cumulative risk)	†	†	†	†
	Maternal patent Plasmodium infection during pregnancy (incidence/100 py)*	†	†	†	†
	Maternal sub-patent Plasmodium infection during pregnancy (cumulative risk)	0.0061	0.0106	0.14	0.24
	Maternal sub-patent Plasmodium infection during pregnancy (incidence/100 py)*	0.0131	0.0086	0.06	0.0317
	Maternal clinical malaria during pregnancy (cumulative risk)	†	†	†	†
	Maternal clinical malaria during pregnancy (incidence/100py)*	†	†	†	†
	Maternal non-malaria sick visits during pregnancy (cumulative risk)	0.84	0.60	0.58	0.46
	Maternal non-malaria sick visits during pregnancy (incidence/100py)*	0.88	0.60	0.64	0.57
	Maternal all-cause sick visits during pregnancy (cumulative risk)	0.87	0.58	0.73	0.54
	Maternal all-cause sick visits during pregnancy (incidence/100py)*	0.81	0.78	0.66	0.48

ITT=intention to treat population, PPP=per protocol population, Crude=unadjusted for co-variates, IST=intermittent screening and treatment during pregnancy with dihydroartemisinin–piperaquine. IPT=intermittent preventive treatment during pregnancy with dihydroartemisinin–piperaquine. SST=Single screening and treatment during pregnancy with dihydroartemisinin–piperaquine; py=person years. P-values for interaction terms were obtained post-hoc using the Altman-Bland method.[37]
* Outcomes represents binary outcome except for the data for the incidence per 100 person-years. † The p-value for interaction could not be computed because the relative risk in at least one of the study sites could not be computed because of zero events in at least one of the study arms.

eTable 10: Site-treatment interaction P-values for key secondary outcomes newborn

		ITT		PPP	
Groups	Outcome	Crude	Adjusted	Crude	Adjusted
IPT vs SST	Congenital malaria (LAMP/PCR/smear/RDT): (age <=7 days)	0.34	0.50	0.57	0.71
	Fetal anaemia (cord Hb<10 g/dL)	0.79	0.85	0.70	0.94
	Small for gestational age (<10th percentile INTERGROWTH)	0.47	0.81	0.17	0.41
	Low birth weight (<2,500g)	0.0150	0.0063	0.0091	0.0056
	Preterm birth (<37 weeks)	0.17	0.14	0.46	0.41
	Adverse livebirth outcome (LBW/SGA/PT)	0.08	0.13	0.08	0.10
	Fetal loss (spontaneous abortion or stillbirth)	0.42	0.61	0.29	0.47
	Adverse pregnancy outcome (fetal loss, LBW/SGA/PT)	0.0457	0.09	0.05	0.08
	Perinatal death	0.49	0.72	0.10	0.17
	Neonatal death (<28 days)	†	†	†	†
	Infant mortality by end of follow-up (6-8 weeks of age)	†	†	†	†
	Adverse pregnancy outcome (fetal loss, LBW/SGA/PT, neonatal death by 4 weeks)	0.0367	0.07	0.0487	0.0430
	Adverse pregnancy outcome (fetal loss, LBW/SGA/PT, infant death by 6-8 weeks)	0.0326	0.06	0.0444	0.0385
IST vs SST	Congenital malaria (LAMP/PCR/smear/RDT): (age <=7 days)	0.08	0.14	0.05	0.12
	Fetal anaemia (cord Hb<10 g/dL)	0.72	0.95	0.73	0.36
	Small for gestational age (<10th percentile INTERGROWTH)	0.49	0.79	0.34	0.75
	Low birth weight (<2,500g)	0.16	0.05	0.0474	0.0227
	Preterm birth (<37 weeks)	0.0032	0.0072	0.0403	0.07
	Adverse livebirth outcome (LBW/SGA/PT)	0.07	0.22	0.07	0.19
	Fetal loss (spontaneous abortion or stillbirth)	0.64	0.77	0.60	0.81
	Adverse pregnancy outcome (fetal loss, LBW/SGA/PT)	0.07	0.25	0.10	0.29
	Perinatal death	0.14	0.44	0.0428	0.16
	Neonatal death (<28 days)	†	†	†	†
	Infant mortality by end of follow-up (6-8 weeks of age)	†	†	†	†
	Adverse pregnancy outcome (fetal loss, LBW/SGA/PT, neonatal death by 4 weeks)	0.0438	0.20	0.07	0.11
	Adverse pregnancy outcome (fetal loss, LBW/SGA/PT, infant death by 6-8 weeks)	0.0466	0.21	0.07	0.12
IPT vs IST	Congenital malaria (LAMP/PCR/smear/RDT): (age <=7 days)	0.43	0.43	0.24	0.27
	Fetal anaemia (cord Hb<10 g/dL)	0.98	0.90	0.53	0.45

Small for gestational age (<10th percentile INTERGROWTH)	0.97	0.98	0.70	0.63
Low birth weight (<2,500g)	0.44	0.58	0.46	0.74
Preterm birth (<37 weeks)	0.28	0.43	0.37	0.50
Adverse livebirth outcome (LBW/SGA/PT)	0.83	0.91	0.96	0.74
Fetal loss (spontaneous abortion or stillbirth)	0.68	0.34	0.43	0.19
Adverse pregnancy outcome (fetal loss, LBW/SGA/PT)	0.99	0.73	0.71	0.48
Perinatal death	0.37	0.69	0.75	0.87
Neonatal death (<28 days)	†	†	†	†
Infant mortality by end of follow-up (6-8 weeks of age)	0.09	0.11	0.11	0.11
Adverse pregnancy outcome (fetal loss, LBW/SGA/PT, neonatal death by 4 weeks)	0.97	0.76	0.76	0.57
Adverse pregnancy outcome (fetal loss, LBW/SGA/PT, infant death by 6-8 weeks)	0.95	0.69	0.67	0.49

ITT=intention to treat population, PPP=per protocol population, Crude=unadjusted for co-variates, IST=intermittent screening and treatment during pregnancy with dihydroartemisinin–piperaquine. IPT=intermittent preventive treatment during pregnancy with dihydroartemisinin–piperaquine. SST=Single screening and treatment during pregnancy with dihydroartemisinin–piperaquine; py=person years

* Outcomes represents binary outcome except for the data for the incidence per 100 person-years. † The p-value for interaction could not be computed because the relative risk in at least one of the study site could not be computed because of zero events in at least one of the study arms.

eTable 11: Maternal and fetal mean haemoglobin, birthweight, gestational age and mean birthweight-for gestational age Z-score by treatment group and site

	Number of women, mean (SD)				mean difference (95% CI), p-value		
	IPT	IST	SST		IPT vs SST	IST vs SST	IPT vs IST
**Maternal haemoglobin (g/dl) at delivery**							
Overall	490	659	589	Unadjusted	0.21 (-0.09, 0.51), p=0.1623	0.27 (-0.05, 0.60), p=0.0948	-0.06 (-0.39, 0.27), p=0.7148
	11.2 (2.0)	11.1 (2.0)	10.9 (2.0)	Adjusted [a]	0.16 (-0.10, 0.427), p=0.2316	0.18 (-0.16, 0.51), p=0.2969	-0.02 (-0.35, 0.31), p=0.9218
Sumba	240	264	277	Unadjusted	0.04 (-0.20, 0.28), p=0.7213	-0.02 (-0.29, 0.26), p=0.9009	0.06 (-0.22, 0.34), p=0.6730
	10.9 (1.9)	10.6 (1.7)	10.7 (1.8)	Adjusted [a]	0.03 (-0.21, 0.27), p=0.8160	-0.07 (-0.36, 0.22), p=0.6169	0.10 (-0.21, 0.41), p=0.5174
Papua	250	395	312	Unadjusted	0.37 (-0.04, 0.79), p=0.0782	0.32 (-0.14, 0.78), p=0.1754	0.05 (-0.22, 0.33), p=0.7044
	11.4 (1.9)	11.4 (2.1)	11.1 (2.2)	Adjusted [a]	0.26 (-0.13, 0.64), p=0.1979	0.16 (-0.27, 0.58), p=0.4626	0.10 (-0.15, 0.34), p=0.4496
**Fetal haemoglobin (g/dL) (cord blood)**							
Overall	495	690	610	Unadjusted	0.70 (0.11, 1.29), p=0.0200	0.22 (-0.40, 0.83), p=0.4901	0.48 (-0.13, 1.10), p=0.1221
	15.7 (3.4)	15.1 (3.4)	15.0 (3.5)	Adjusted [a]	0.63 (0.28, 0.99), p=0.0004	0.23 (-0.10, 0.56), p=0.1777	0.41 (-0.01, 0.81), p=0.0530
Sumba	248	279	283	Unadjusted	0.55 (0.00, 1.09), p=0.0485	0.17 (-0.35, 0.69), p=0.5213	0.38 (-0.22, 0.98), p=0.2158
	16.3 (3.4)	16.0 (3.4)	15.8 (3.5)	Adjusted [a]	0.56 (0.05, 1.07), p=0.0320	0.25 (-0.30, 0.80), p=0.3737	0.31 (-0.36, 0.98), p=0.3618
Papua	247	411	327	Unadjusted	0.82 (0.23, 1.41), p=0.0063	0.27 (-0.25, 0.79), p=0.3115	0.55 (-0.08, 1.18), p=0.0858
	15.0 (3.2)	14.5 (3.2)	14.3 (3.3)	Adjusted [a]	0.65 (0.26, 1.04), p=0.0011	0.33 (0.08, 0.59), p=0.0106	0.32 (-0.11, 0.74), p=0.1442
**Birth weight (grams)**							
Overall	512	698	626	Unadjusted	-77 (-150, -3), p=0.0420	-6 (-81, 69), p=0.8736	-71 (-151, 10), p=0.0871
	2899 (437)	2980 (459)	2971 (469)	Adjusted [a]	-80 (-135, -24), p=0.0048	-17 (-78, 45), p=0.6008	-63 (-114, -12), p=0.0159
Sumba	243	278	287	Unadjusted	-129 (-203, -55), p=0.0007	-42 (-131, 47), p=0.3585	-87 (-164, -10), p=0.0265
	2786 (392)	2878 (457)	2917 (422)	Adjusted [a]	-119 (-190, -48), p=0.0010	-58 (-143, 27), p=0.1778	-61 (-142, 21), p=0.1440
Papua	269	420	339	Unadjusted	-6 (-88, 77), p=0.8927	35 (-49, 119), p=0.4085	-41 (-122, 40), p=0.3193
	3002 (450)	3048 (448)	3017 (500)	Adjusted [a]	-45 (-125, 35), p=0.2727	-1 (-64, 62), p=0.9759	-44 (-112, 25), p=0.2108
**Gestational age at birth (weeks)**							
Overall	536	727	643	Unadjusted	-0.07 (-0.23, 0.09), p=0.3890	0.03 (-0.16, 0.22), p=0.7525	-0.10 (-0.29, 0.09), p=0.2913
	37.7 (1.5)	38.3 (1.7)	37.8 (1.5)	Adjusted [a]	-0.10 (-0.24, 0.05), p=0.1946	-0.01 (-0.20, 0.19), p=0.9465	-0.09 (-0.27, 0.09), p=0.3307
Sumba	257	293	293	Unadjusted	-0.15 (-0.33, 0.03), p=0.0910	-0.02 (-0.22, 0.19), p=0.8650	-0.14 (-0.33, 0.05), p=0.1560

	Number of women, mean (SD)				mean difference (95% CI), p-value		
	**IPT**	**IST**	**SST**		**IPT vs SST**	**IST vs SST**	**IPT vs IST**
	37.6 (1.6)	38.3 (1.6)	37.8 (1.2)	Adjusted [a]	-0.18 (-0.36, 0.00), p=0.0482	0.00 (-0.28, 0.29), p=0.9930	-0.18 (-0.47, 0.10), p=0.2089
Papua	279	434	350	Unadjusted	0.06 (-0.17, 0.29), p=0.6100	0.08 (-0.23, 0.39), p=0.6187	-0.02 (-0.29, 0.25), p=0.8882
	37.8 (1.4)	38.3 (1.7)	37.8 (1.7)	Adjusted [a]	-0.07 (-0.27, 0.13), p=0.4931	-0.06 (-0.32, 0.19), p=0.6356	-0.01 (-0.24, 0.23), p=0.9457
**Birthweight for gestational age (Z-score)**							
Overall	512	696	626	Unadjusted	-0.16 (-0.33, 0.00), p=0.0551	0.00 (-0.15, 0.16), p=0.9578	-0.17 (-0.33, 0.00), p=0.0478
	-0.19 (1.06)	-0.02 (1.06)	-0.03 (1.09)	Adjusted [a]	-0.18 (-0.31, -0.05), p=0.0055	-0.04 (-0.18, 0.10), p=0.5687	-0.14 (-0.25, -0.03), p=0.0140
Sumba	243	276	287	Unadjusted	-0.25 (-0.44, -0.06), p=0.0093	-0.04 (-0.25, 0.17), p=0.7219	-0.22 (-0.41, -0.02), p=0.0283
	-0.40 (1.00)	-0.17 (1.05)	-0.14 (0.99)	Adjusted [a]	-0.23 (-0.39, -0.07), p=0.0058	-0.11 (-0.30, 0.08), p=0.2719	-0.12 (-0.31, 0.07), p=0.1998
Papua	269	420	339	Unadjusted	-0.08 (-0.26, 0.09), p=0.3666	0.02 (-0.13, 0.17), p=0.8313	-0.10 (-0.25, 0.05), p=0.2049
	-0.01 (1.09)	0.09 (1.06)	0.07 (1.15)	Adjusted [a]	-0.14 (-0.32, 0.04), p=0.1236	-0.04 (-0.18, 0.11), p=0.6330	-0.10 (-0.23, 0.03), p=0.1213

ITT population; [a] adjusted for site, gravidity, malaria at enrolment by PCR, rain/seasonality six months prior to delivery, ITN use, Hb at enrolment (for Hb outcome), gestational age at enrolment, and educational status.

eTable 12: Adverse events associated with drug tolerability

	IST [a]			IPT [a]			SST [a]		
**Within 30 minutes following drug administration**									
Vomiting initial dose	0/27 (0%)			1/678 (0.1%)			0/35 (0%)		
Vomiting repeat dose	0/0 (0%)			0/1 (0%)			0/0 (0%)		
**Tolerability 1-7 days following drug administration**									
Number of women (number of courses)	27 (29)			678 (2058)[a]			35 (35)		
	Women (%)	Events	IR (95% CI)[b]	Women (%)	Events	IR (95% CI)[b]	Women (%)	Events	IR (95% CI)[b]
Any reported drug tolerability event	2 (0.3%)	6	20.7 (7.6-45.0)	135 (19.9%)	238	11.6 (10.1-13.1)	2 (0.3%)	2	5.7 (0.7-20.6)
Pyrexia	1 (0.1%)	1	3.4 (0.1-19.2)	7 (1.0%)	8	0.4 (0.2-0.8)	0 (0%)	0	0.0 (0.0-0.0)
Asthenia	1 (0.1%)	1	3.4 (0.1-19.2)	5 (0.7%)	5	0.2 (0.1-0.6)	0 (0%)	0	0.0 (0.0-0.0)
Headache	2 (0.3%)	2	6.9 (0.8-24.9)	58 (8.6%)	68	3.3 (2.6-4.2)	0 (0%)	0	0.0 (0.0-0.0)
Abdominal pain[c]	0 (0%)	0	0.0 (0.0-0.0)	3 (0.4%)	3	0.1 (0.0-0.4)	0 (0%)	0	0.0 (0.0-0.0)
Myalgia	0 (0%)	0	0.0 (0.0-0.0)	1 (0.1%)	1	0.0 (0.0-0.3)	0 (0%)	0	0.0 (0.0-0.0)
Nausea	1 (0.1%)	1	3.4 (0.1-19.2)	53 (7.8%)	63	3.1 (2.4-3.9)	1 (0.1%)	1	2.9 (0.1-15.9)
Rash[d]	0 (0%)	0	0.0 (0.0-0.0)	0 (0%)	0	0.0 (0.0-0.0)	0 (0%)	0	0.0 (0.0-0.0)
Diarrhoe[e]	0 (0%)	0	0.0 (0.0-0.0)	3 (0.4%)	3	0.1 (0.0-0.4)	0 (0%)	0	0.0 (0.0-0.0)
Vomiting[f]	1 (0.1%)	1	3.4 (0.1-19.2)	71 (10.5%)	87	4.2 (3.4-5.2)	1 (0.1%)	1	2.9 (0.1-15.9)
Dizziness	0 (0%)	0	0.0 (0.0-0.0)	23 (3.4%)	25	1.2 (0.8-1.8)	0 (0%)	0	0.0 (0.0-0.0)

[a] For IPT this is based on prompted questions during follow-up visits at home. For IST and SST this includes both treatments based on RDT positivity detected during a scheduled screening event, and data from treatment given to RDT positive women presenting for unscheduled sick visits (there were none in the IPT arm).
[b] incidence rate per 100 person-years
[c] includes MeDRA's preferred terms for 'abdominal pain', 'abdominal pain lower' and 'abdominal pain upper'
[d] includes MeDRA's preferred terms for 'rash pruritic' and 'rash macular'
[e] includes MeDRA's preferred terms for 'diarrhoea' and 'diarrhoea haemorrhagic'
[f] Late vomiting (>30 minutes following drug administration). All of these events occurred within the first 3 days after the start of drug intake, i.e. during or within the 24h after drug intake but excluding the first 30 minutes.

eTable 13: Serious Adverse Events: case descriptions of maternal deaths

Arm	
**IST**	**Post-partum haemorrhage**<br>A 33-year-old gravidae 3, enrolled in IST arm presented at 36 weeks gestation to the Timika General Hospital with contractions and fever. She gave birth to a live baby after four hours by spontaneous vaginal delivery. After delivery, she had retention of the placenta and died due to post-partum haemorrhage on the same day. She had been enrolled into the study two months earlier at 28 weeks of gestation by fundal height examination. She had one previous live birth and one abortion. Her physical condition and vital signs at enrolment were normal and fetal viability was confirmed by Doppler. The mRDT was negative and her Hb was 9.5 g/dL. She received ferrous sulfate tablets 200mg/day and calcium supplement 500mg/day. The physical examination at her first scheduled antenatal follow up visit at one month was normal. Her mRDT at that visit was negative. Because all her RDTs were negative throughout her antenatal follow-up, she did not receive DP during her pregnancy.
**IST**	**Spontaneous abortion in field followed by maternal death at home**<br>A 31-year-old primigravidae enrolled in IST arm had a post-partum death. The study staff became aware of her death three weeks later when she failed to attend the scheduled follow up visit. The history of event was as follows: In the afternoon of the previous day she left her home to go to the field about 10 kilometres from her village. When she left home, she was in good condition according to her sister. While in the field, she went into labour and gave birth spontaneously to a live born baby. She wrapped the baby, with the umbilical cord intact, using her clothes and went to a house in the nearest village to find help. The baby had died by the time she arrived. The residents of the house got the village traditional birth attendant to deliver her placenta. There was no history of post-partum bleeding and the following day she went back home. While at home she fainted. The family decided to take her to the local hospital, but she died on the way. She was enrolled into the study on 2 weeks before her death. At enrolment, her gestational age by fundal height examination was 26 weeks and fetal heart was detected by Doppler. Her physical examination was normal. Her haemoglobin was 10.3g/dL and mRDT was negative. She did not receive DP during her pregnancy and she did not take any other medications.
**SST**	**Septic shock secondary to pneumonia**<br>A 20-year-old primigravida enrolled in SST arm presented to hospital emergency room with haemoptysis and dyspnoea. She had vomited about 50 cc of blood about three hours before she came to hospital. There was no previous history of dyspnoea or haemoptysis. She was admitted comatose with a diagnosis of septic shock secondary to pneumonia. Soon after admission her condition deteriorated, and she was declared dead 46 minutes later. The blood sugar on admission was 170 mg/dl and malaria microscopy were negative. She received the following medication intravenously: Asering 1500 cc/24 hours, ceftriaxone 2x2g, antrain 1g/8hrs, ranitidine 50mg/8hrs, tranexamic acid 1000mg/8hrs. She was enrolled into the study five months earlier at 17 weeks gestation estimated by fundal height. Her physical examination was normal. The mRDT was negative. She subsequently attended three scheduled ANC visits. Her medical and obstetric history and examinations were all normal during these visits. She received supplementation with ferrous sulfate 200mg daily and calcium (kalk) 500mg daily. She did not receive DP during her pregnancy.

eFigure detailed trial profile

eFigure 1: Trial profile by arm and overall

STOPMIP Indonesia trial

	Sumba	Timika	Overall
Clusters assessed for eligibility	69	21	90
Excluded	12	0	12
not meeting inclusion criteria	12	0	12
decline to participate	0	0	0
Clusters randomised	57	21	78

	Sumba SSTp	Sumba ISTp	Sumba IPTp	Sumba All	Timika SSTp	Timika ISTp	Timika IPTp	Timika All	Overall SSTp	Overall ISTp	Overall IPTp	Overall All
Clusters randomised	19	19	19	57	7	7	7	21	26	26	26	78
Women screened	428	465	355	1248	452	976	877	2305	880	1441	1232	3553
Not eligible	53 (12%)	64 (14%)	39 (11%)	156 (13%)	38 (8%)	467 (48%)	471 (54%)	976 (42%)	91 (10%)	531 (37%)	510 (41%)	1132 (32%)
Eligible, not included*	37 (9%)	42 (9%)	23 (6%)	102 (8%)	7 (2%)	14 (1%)	18 (2%)	39 (2%)	44 (5%)	56 (4%)	41 (3%)	141 (4%)
Recruited (ITT population)	337 (79%)	359 (77%)	293 (83%)	989 (79%)	407 (90%)	495 (51%)	388 (44%)	1290 (56%)	744 (85%)	854 (59%)	681 (55%)	2279 (64%)
Received intervention >=once‡	337 (100%)	359 (100%)	293 (100%)	989 (100%)	407 (100%)	495 (100%)	385 (99%)	1287 (100%)	744 (100%)	854 (100%)	678 (100%)	2276 (100%)
ITT population	337	359	293	989	407	495	388	1290	744	854	681	2279
Contributed to primary analysis	290 (86%)	285 (79%)	256 (87%)	831 (84%)	343 (84%)	428 (86%)	272 (70%)	1043 (81%)	633 (85%)	713 (83%)	528 (78%)	1874 (82%)
Number excluded	47 (14%)	74 (21%)	37 (13%)	158 (16%)	64 (16%)	67 (14%)	116 (30%)	247 (19%)	111 (15%)	141 (17%)	153 (22%)	405 (18%)
Reason for exclusion												
withdrew consent	2 (0.6%)	11 (3%)	9 (3%)	22 (2%)	7 (2%)	0 (0%)	53 (14%)	60 (5%)	9 (1.2%)	11 (1.3%)	62 (9%)	82 (4%)
lost to follow-up	42 (12%)	58 (16%)	27 (9%)	127 (13%)	24 (6%)	37 (7%)	26 (7%)	87 (7%)	66 (9%)	95 (11%)	53 (8%)	214 (9%)
missed delivery	3 (0.9%)	2 (0.6%)	1 (0.3%)	6 (0.6%)	31 (8%)	30 (6%)	34 (9%)	95 (7%)	34 (5%)	32 (4%)	35 (5%)	101 (4%)
screening error	0 (0%)	2 (0.6%)	0 (0%)	2 (0.2%)	2 (0.5%)	0 (0%)	3 (0.8%)	5 (0.4%)	2 (0.3%)	2 (0.2%)	3 (0.4%)	7 (0.3%)
death	0 (0%)	1 (0.3%)	0 (0%)	1 (0.1%)	0 (0%)	0 (0%)	0 (0%)	0 (0%)	0 (0%)	1 (0.1%)	0 (0%)	1 (0.04%)
Contributed to PP analysis$	222 (66%)	228 (64%)	195 (67%)	645 (65%)	239 (59%)	291 (59%)	167 (43%)	697 (54%)	461 (62%)	519 (61%)	362 (53%)	1342 (59%)
Number excluded	115 (34%)	131 (36%)	98 (33%)	344 (35%)	168 (41%)	204 (41%)	221 (57%)	593 (46%)	283 (38%)	335 (39%)	319 (47%)	937 (41%)
Reason for exclusion												
missing primary endpoint (see ITT)	47 (14%)	74 (21%)	37 (13%)	158 (16%)	64 (16%)	67 (14%)	116 (30%)	247 (19%)	111 (15%)	141 (17%)	153 (22%)	405 (18%)
non-adherance to regimen	2 (0.6%)	11 (3%)	14 (5%)	27 (3%)	29 (7%)	25 (5%)	31 (8%)	85 (7%)	31 (4%)	36 (4%)	45 (7%)	112 (5%)
prohibited medication	2 (0.6%)	5 (1.4%)	0 (0%)	7 (0.7%)	6 (1.5%)	4 (0.8%)	0 (0%)	10 (0.8%)	8 (1.1%)	9 (1.1%)	0 (0%)	17 (0.7%)
missed a scheduled visit	64 (19%)	41 (11%)	47 (16%)	152 (15%)	69 (17%)	108 (22%)	74 (19%)	251 (19%)	133 (18%)	149 (17%)	121 (18%)	403 (18%)

ITT=Intention to treat, PP=per protocol.

*The number of recruited participants per clinic cluster was restricted to a maximum of 5 per day to keep the follow-up numbers manageable in subsequent visits. On some days, more than 5 participants were eligible, in which case they were chosen at random by drawing lots among all eligible participants who presented that morning. ‡ At enrolment all participants received dihydroartemisinin-piperaquine. No participants received quinine-clindamycin. $ The numbers reflect the participants contributing to the per protocol analysis for the primary outcome.

Participants lost to follow-up prior to delivery and participants who withdrew consent were included in the ITT population and contributed to the antenatal follow-up analyses (e.g. incidence of malaria).

eFigures efficacy outcomes

eFigure 2: Subgroup analysis of the effect on the primary outcomes in the ITT population (IPT vs SST)

IPT vs SST	Subgroup	IPT n/N (%)	SST n/N (%)	Crude RR (95% CI)	P-value	P-value for interaction
Gravidity (pregnancy number)						
	>=Third	30/243 (12.3)	57/296 (19.3)	0.64 (0.43, 0.96)	0.0309	0.63
	First or second	31/285 (10.9)	71/337 (21.1)	0.55 (0.34, 0.89)	0.0155	
.						
ITN use during pregnancy						
	ITN non-user	3/40 (7.5)	7/38 (18.4)	0.54 (0.18, 1.64)	0.2752	0.52
	ITN user	58/488 (11.9)	121/595 (20.3)	0.61 (0.43, 0.86)	0.0051	
.						
Malaria status at enrolment						
	Negative	50/469 (10.7)	100/548 (18.2)	0.59 (0.42, 0.82)	0.0015	0.98
	Positive	11/59 (18.6)	28/85 (32.9)	0.58 (0.29, 1.18)	0.1318	
.						
Species at enrolment*						
	P.falciparum mono-infection	7/37 (18.9)	7/35 (20.0)	0.95 (0.33, 2.72)	0.9258	0.32
	P.vivax mono-infection	2/10 (20.0)	12/26 (46.2)	0.43 (0.14, 1.34)	0.1467	
.						
socioeconomic status (terciles)						
	Lowest	29/154 (18.8)	57/242 (23.6)	0.81 (0.53, 1.23)	0.3254	0.09
	Middle	9/176 (5.1)	35/208 (16.8)	0.31 (0.16, 0.62)	0.0009	
	Highest	23/198 (11.6)	36/183 (19.7)	0.59 (0.38, 0.91)	0.0170	
.						
Season: rainfall 6m before delivery (terciles)						
	Lowest	26/226 (11.5)	51/277 (18.4)	0.62 (0.40, 0.96)	0.0319	0.06
	Middle	23/127 (18.1)	36/164 (22.0)	0.82 (0.52, 1.30)	0.3920	
	Highest	12/175 (6.9)	41/192 (21.4)	0.32 (0.16, 0.66)	0.0021	
.						
Gestational age at enrolment						
	Later (> median)	28/266 (10.5)	70/314 (22.3)	0.47 (0.31, 0.74)	0.0008	0.35
	Earlier (<=median)	33/262 (12.6)	58/319 (18.2)	0.70 (0.47, 1.06)	0.0910	
.						

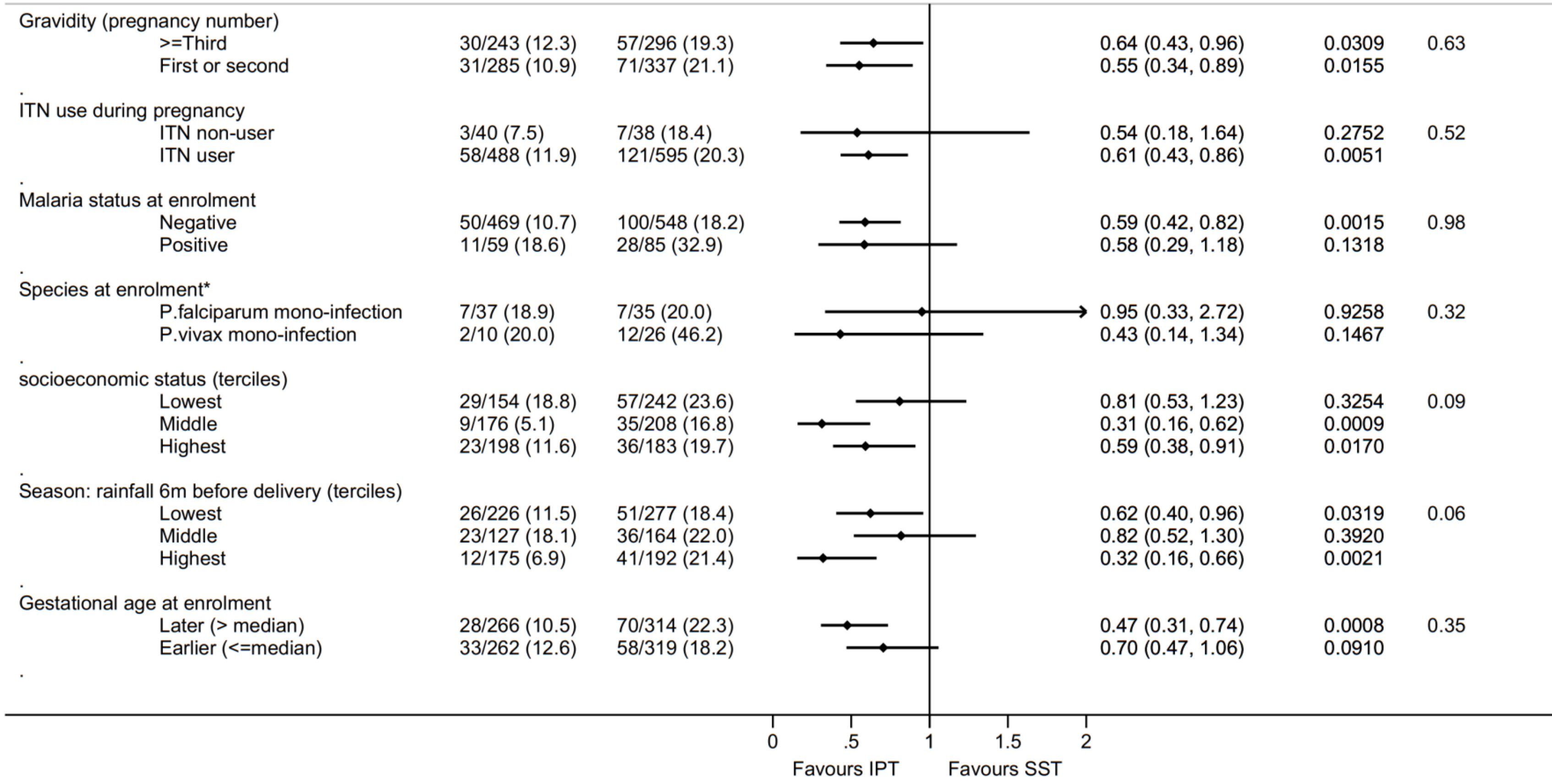

RR=relative risk, CI=confidence interval, ITN=insecticide treated net. P-values for interaction terms were obtained using the Altman-Bland method[37] for binary variables and by interaction term models for outcomes 3 or more categories. * Subgroup analysis defined post-hoc. Mixed species and other species excluded because of small numbers.

eFigure 3: Subgroup analysis of the effect on the primary outcomes in the ITT population (IST vs SST)

IST vs SST	Subgroup	IST n/N (%)	SST n/N (%)	Crude RR (95% CI)	P-value	P-value for interaction
Gravidity (pregnancy number)						
	>=Third	34/319 (10.7)	57/296 (19.3)	0.55 (0.37, 0.83)	0.0048	0.97
	First or second	50/394 (12.7)	71/337 (21.1)	0.56 (0.36, 0.87)	0.0107	
.						
ITN use during pregnancy						
	ITN non-user	11/70 (15.7)	7/38 (18.4)	0.91 (0.43, 1.92)	0.8032	0.20
	ITN user	73/643 (11.4)	121/595 (20.3)	0.53 (0.37, 0.76)	0.0006	
.						
Malaria status at enrolment						
	Negative	75/672 (11.2)	100/548 (18.2)	0.60 (0.44, 0.83)	0.0018	0.90
	Positive	9/41 (22.0)	28/85 (32.9)	0.63 (0.31, 0.93)	0.1893	
.						
Species at enrolment*						
	P.falciparum mono-infection	4/20 (20.0)	7/35 (20.0)	0.95 (0.31, 2.91)	0.9231	0.06
	P.vivax mono-infection	1/14 (7.1)	12/26 (46.2)	0.15 (0.03, 0.68)	0.0143	
.						
socioeconomic status (terciles)						
	Lowest	36/218 (16.5)	57/242 (23.6)	0.71 (0.46, 1.09)	0.1165	0.16
	Middle	15/234 (6.4)	35/208 (16.8)	0.36 (0.19, 0.69)	0.0023	
	Highest	33/261 (12.6)	36/183 (19.7)	0.63 (0.39, 1.03)	0.0652	
.						
Season: rainfall 6m before delivery (terciles)						
	Lowest	45/334 (13.5)	51/277 (18.4)	0.73 (0.52, 1.02)	0.0688	0.09
	Middle	14/165 (8.5)	36/164 (22.0)	0.38 (0.23, 0.63)	0.0002	
	Highest	25/214 (11.7)	41/192 (21.4)	0.47 (0.27, 0.81)	0.0070	
.						
Gestational age at enrolment						
	Later (> median)	37/317 (11.7)	70/314 (22.3)	0.52 (0.35, 0.76)	0.0009	0.46
	Earlier (<=median)	47/396 (11.9)	58/319 (18.2)	0.64 (0.43, 0.95)	0.0259	
.						

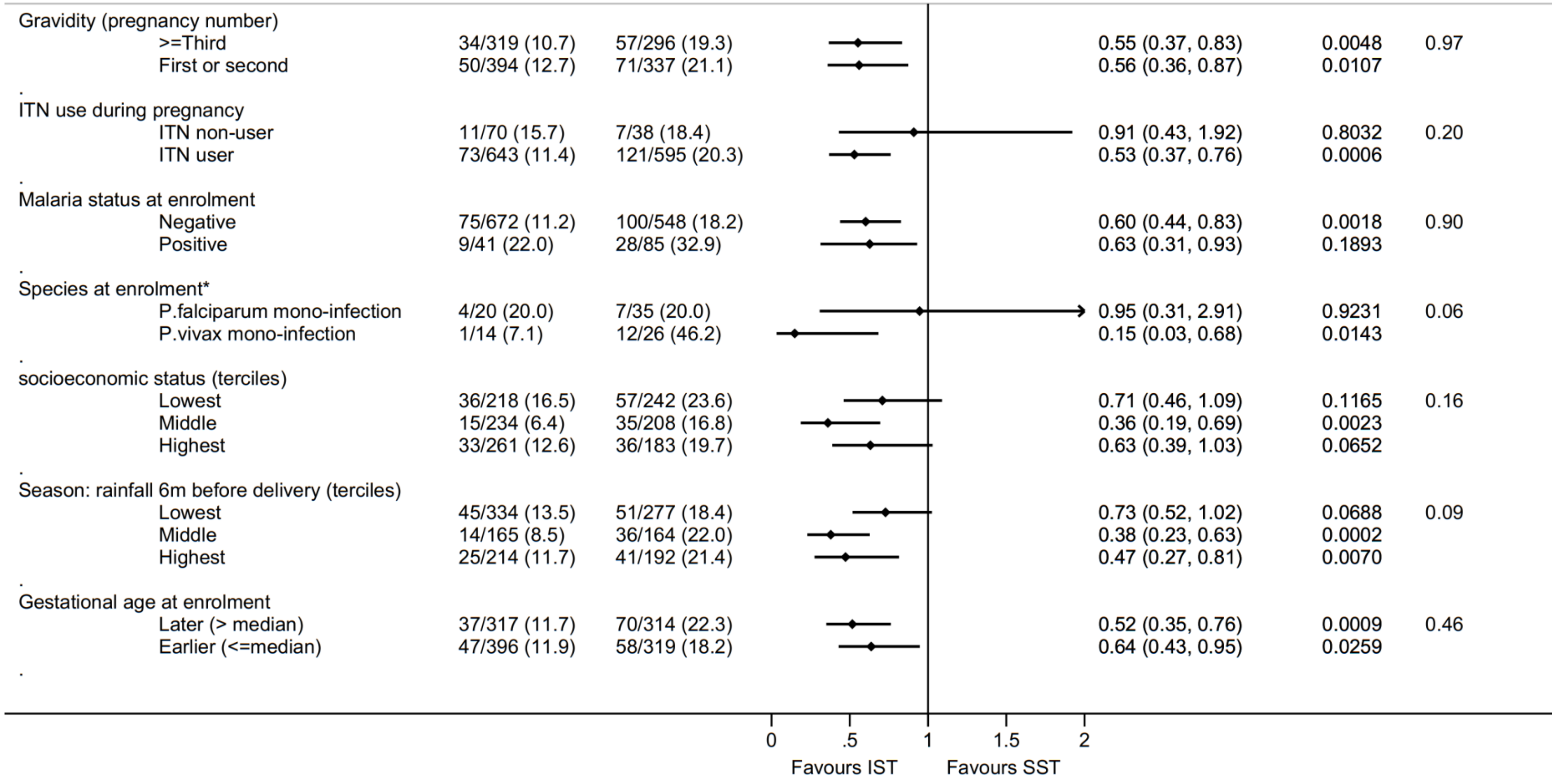

RR=relative risk, CI=confidence interval, ITN=insecticide treated net. P-values for interaction terms were obtained using the Altman-Bland method[37] for binary variables and by interaction term models for outcomes 3 or more categories. * Subgroup analysis defined post-hoc. Mixed species and other species excluded because of small numbers.

eFigure 4: Subgroup analysis of the effect on the primary outcomes in the ITT population (IPT vs IST)

IPT vs IST	Subgroup	IPT n/N (%)	IST n/N (%)	Crude RR (95% CI)	P-value	P-value for interaction
Gravidity (pregnancy number)						
	>=Third	30/243 (12.3)	34/319 (10.7)	1.16 (0.74, 1.83)	0.5178	0.62
	First or second	31/285 (10.9)	50/394 (12.7)	0.98 (0.61, 1.59)	0.9491	
.						
ITN use during pregnancy						
	ITN non-user	3/40 (7.5)	11/70 (15.7)	0.59 (0.23, 1.51)	0.2721	0.20
	ITN user	58/488 (11.9)	73/643 (11.4)	1.15 (0.77, 1.74)	0.4926	
.						
Malaria status at enrolment						
	Negative	50/469 (10.7)	75/672 (11.2)	0.98 (0.69, 1.39)	0.9023	0.92
	Positive	11/59 (18.6)	9/41 (22.0)	0.93 (0.40, 2.15)	0.8667	
.						
Species at enrolment*						
	P.falciparum mono-infection	7/37 (18.9)	4/20 (20.0)	1.01 (0.34, 2.95)	0.9920	0.31
	P.vivax mono-infection	2/10 (20.0)	1/14 (7.1)	2.91 (0.50, 17.03)	0.2369	
.						
socioeconomic status (terciles)						
	Lowest	29/154 (18.8)	36/218 (16.5)	1.14 (0.72, 1.81)	0.5711	0.94
	Middle	9/176 (5.1)	15/234 (6.4)	0.87 (0.37, 2.06)	0.7504	
	Highest	23/198 (11.6)	33/261 (12.6)	0.94 (0.55, 1.58)	0.8050	
.						
Season: rainfall 6m before delivery (terciles)						
	Lowest	26/226 (11.5)	45/334 (13.5)	0.86 (0.57, 1.29)	0.4584	0.0382
	Middle	23/127 (18.1)	14/165 (8.5)	2.16 (1.19, 3.91)	0.0108	
	Highest	12/175 (6.9)	25/214 (11.7)	0.68 (0.31, 1.48)	0.3308	
.						
Gestational age at enrolment						
	Later (> median)	28/266 (10.5)	37/317 (11.7)	0.92 (0.57, 1.50)	0.7352	0.59
	Earlier (<=median)	33/262 (12.6)	47/396 (11.9)	1.11 (0.69, 1.77)	0.6739	
.						

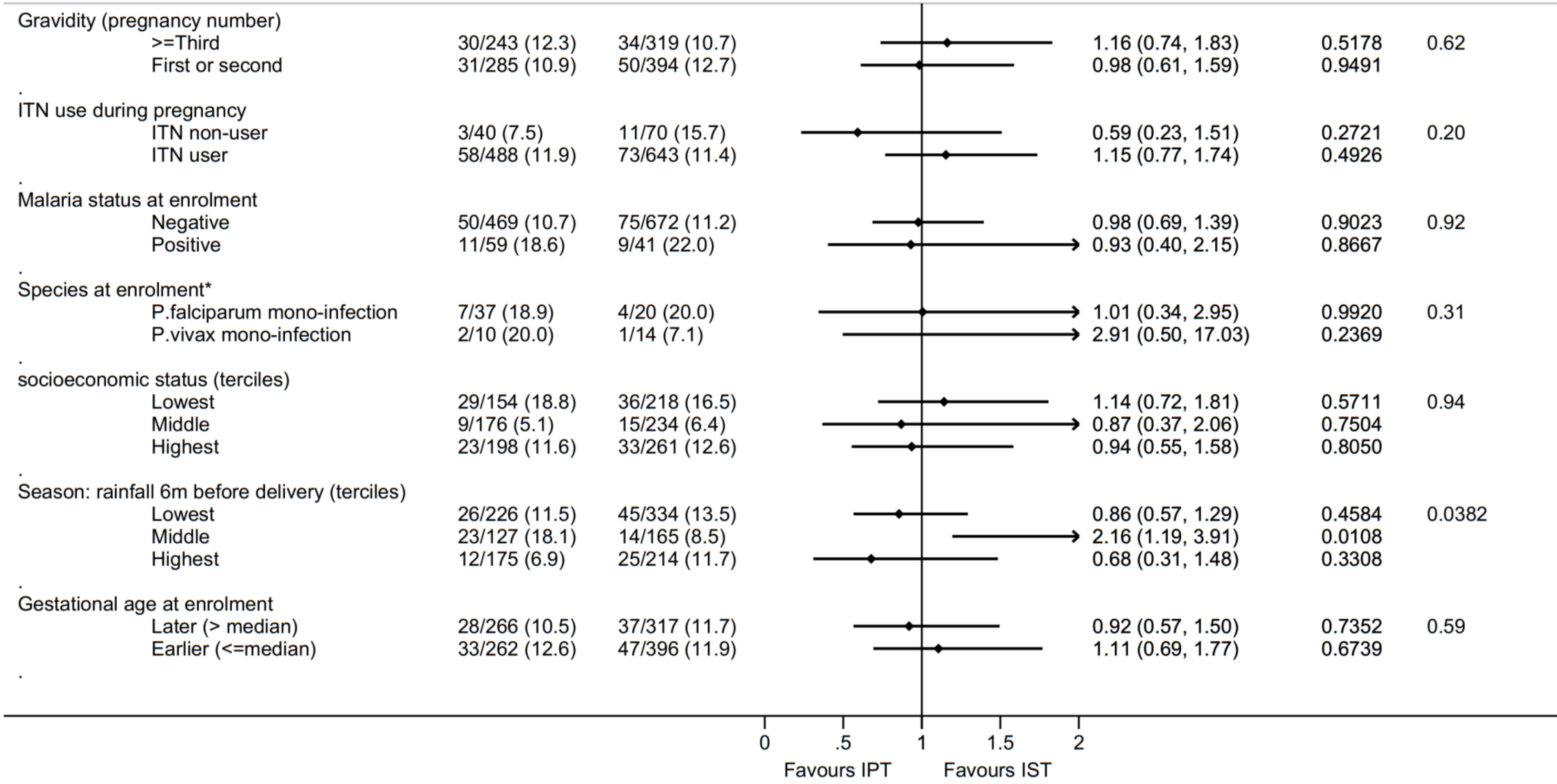

RR=relative risk, CI=confidence interval, ITN=insecticide treated net. P-values for interaction terms were obtained using the Altman-Bland method[37] for binary variables and by interaction term models for outcomes 3 or more categories. * Subgroup analysis defined post-hoc. Mixed species and other species excluded because of small numbers.

eFigure 5: Malaria at delivery (primary outcome) and key secondary outcomes in the per protocol population (IPT vs SST)

IPT vs SST	IPT n/N (%)	SST, n/N (%)	Crude RR (95% CI)	P-value	Adjusted RR (95% CI)	P-value
Overall						
Maternal peripheral or placental Plasmodium infection at delivery (primary outcome)	35/362 (9.7)	91/461 (19.7)	0.50 (0.33, 0.74)	0.0005	0.56 (0.39, 0.81)	0.0019
Maternal Plasmodium infection during pregnancy (LAMP/PCR/smear/RDT) (cumulative risk)	39/375 (10.4)	87/494 (17.6)	0.63 (0.39, 1.02)	0.06	0.68 (0.45, 1.04)	0.07
Maternal clinical malaria during pregnancy (cumulative risk)	2/375 (0.5)	9/494 (1.8)	0.32 (0.07, 1.52)	0.15	0.33 (0.08, 1.49)	0.15
Maternal peripheral patent Plasmodium infection at delivery	0/358 (0.0)	8/455 (1.8)	.†	.	.†	.
Maternal peripheral sub-patent Plasmodium infection at delivery	18/358 (5.0)	40/455 (8.8)	0.57 (0.33, 1.02)	0.06	0.64 (0.37, 1.10)	0.11
Placental Plasmodium infection (LAMP/PCR/smear/RDT/histo[active+past])	24/348 (6.9)	65/443 (14.7)	0.50 (0.31, 0.83)	0.0075	0.51 (0.34, 0.77)	0.0012
Maternal Hb<9 g/dl at delivery	37/358 (10.3)	70/459 (15.3)	0.64 (0.45, 0.93)	0.0174	0.68 (0.51, 0.90)	0.0081
Fetal anaemia (cord Hb<12.5 g/dL)	57/340 (16.8)	99/440 (22.5)	0.74 (0.54, 1.01)	0.06	0.81 (0.64, 1.02)	0.08
Small for gestational age (<10th perc INTERGROWTH)	47/350 (13.4)	51/456 (11.2)	1.20 (0.84, 1.73)	0.32	1.21 (0.90, 1.63)	0.20
Low birth weight (<2,500g)	68/350 (19.4)	58/457 (12.7)	1.54 (1.05, 2.26)	0.0278	1.59 (1.14, 2.22)	0.0059
Preterm birth (<37 weeks)	45/357 (12.6)	43/464 (9.3)	1.34 (0.87, 2.06)	0.19	1.36 (0.89, 2.08)	0.16
Fetal loss (spontaneous abortion or stillbirth)	7/372 (1.9)	3/477 (0.6)	2.96 (0.83, 10.58)	0.10	3.19 (0.90, 11.29)	0.07
Neonatal death (<28 days)	2/357 (0.6)	4/463 (0.9)	0.66 (0.10, 4.41)	0.66	0.64 (0.10, 4.19)	0.65
Adverse pregnancy outcome (fetal loss, LBW/SGA/PT)	103/372 (27.7)	100/477 (21.0)	1.31 (0.99, 1.73)	0.06	1.38 (1.09, 1.73)	0.0073
.						
Sumba						
Maternal peripheral or placental Plasmodium infection at delivery (primary outcome)	18/195 (9.2)	39/222 (17.6)	0.52 (0.31, 0.89)	0.0162	0.55 (0.33, 0.89)	0.0153
Maternal Plasmodium infection during pregnancy (LAMP/PCR/smear/RDT) (cumulative risk)	27/201 (13.4)	30/236 (12.7)	1.07 (0.58, 1.96)	0.83	1.10 (0.64, 1.91)	0.73
Maternal clinical malaria during pregnancy (cumulative risk)	0/201 (0.0)	0/236 (0.0)	.†	.	.†	.
Maternal peripheral patent Plasmodium infection at delivery	0/193 (0.0)	1/216 (0.5)	.†	.	.†	.
Maternal peripheral sub-patent Plasmodium infection at delivery	12/193 (6.2)	22/216 (10.2)	0.61 (0.29, 1.30)	0.20	0.59 (0.28, 1.26)	0.17
Placental Plasmodium infection (LAMP/PCR/smear/RDT/histo[active+past])	9/190 (4.7)	17/215 (7.9)	0.67 (0.34, 1.31)	0.24	0.65 (0.33, 1.30)	0.23
Maternal Hb<9 g/dl at delivery	24/193 (12.4)	31/221 (14.0)	0.84 (0.53, 1.33)	0.46	0.81 (0.52, 1.27)	0.36
Fetal anaemia (cord Hb<12.5 g/dL)	22/186 (11.8)	36/214 (16.8)	0.71 (0.45, 1.12)	0.14	0.72 (0.47, 1.12)	0.14
Small for gestational age (<10th perc INTERGROWTH)	32/184 (17.4)	27/220 (12.3)	1.44 (0.94, 2.20)	0.10	1.49 (0.99, 2.23)	0.05
Low birth weight (<2,500g)	49/184 (26.6)	27/220 (12.3)	2.19 (1.37, 3.48)	0.0010	2.30 (1.46, 3.62)	0.0003
Preterm birth (<37 weeks)	31/190 (16.3)	24/222 (10.8)	1.48 (0.90, 2.43)	0.12	1.54 (0.93, 2.55)	0.10
Fetal loss (spontaneous abortion or stillbirth)	5/195 (2.6)	1/223 (0.4)	5.71 (0.72, 45.49)	0.10	6.03 (0.71, 51.14)	0.10
Neonatal death (<28 days)	0/190 (0.0)	0/221 (0.0)	.†	.	.†	.
Adverse pregnancy outcome (fetal loss, LBW/SGA/PT)	72/195 (36.9)	53/223 (23.8)	1.56 (1.15, 2.10)	0.0038	1.59 (1.18, 2.12)	0.0020
.						
Papua						
Maternal peripheral or placental Plasmodium infection at delivery (primary outcome)	17/167 (10.2)	52/239 (21.8)	0.47 (0.27, 0.82)	0.0075	0.51 (0.31, 0.83)	0.0071
Maternal Plasmodium infection during pregnancy (LAMP/PCR/smear/RDT) (cumulative risk)	12/174 (6.9)	57/258 (22.1)	0.30 (0.14, 0.65)	0.0024	0.37 (0.18, 0.76)	0.0069
Maternal clinical malaria during pregnancy (cumulative risk)	2/174 (1.1)	9/258 (3.5)	0.31 (0.07, 1.37)	0.12	0.31 (0.07, 1.37)	0.12
Maternal peripheral patent Plasmodium infection at delivery	0/165 (0.0)	7/239 (2.9)	.†	.	.†	.
Maternal peripheral sub-patent Plasmodium infection at delivery	6/165 (3.6)	18/239 (7.5)	0.53 (0.29, 0.95)	0.0329	0.66 (0.35, 1.24)	0.19
Placental Plasmodium infection (LAMP/PCR/smear/RDT/histo[active+past])	15/158 (9.5)	48/228 (21.1)	0.45 (0.26, 0.79)	0.0052	0.45 (0.26, 0.79)	0.0053
Maternal Hb<9 g/dl at delivery	13/165 (7.9)	39/238 (16.4)	0.47 (0.29, 0.78)	0.0030	0.57 (0.41, 0.79)	0.0006
Fetal anaemia (cord Hb<12.5 g/dL)	35/154 (22.7)	63/226 (27.9)	0.79 (0.56, 1.12)	0.18	0.74 (0.55, 0.99)	0.0416
Small for gestational age (<10th perc INTERGROWTH)	15/166 (9.0)	24/236 (10.2)	0.93 (0.59, 1.46)	0.75	1.13 (0.68, 1.88)	0.63
Low birth weight (<2,500g)	19/166 (11.4)	31/237 (13.1)	0.89 (0.54, 1.45)	0.63	0.77 (0.41, 1.44)	0.42
Preterm birth (<37 weeks)	14/167 (8.4)	19/242 (7.9)	1.01 (0.41, 2.46)	0.99	1.01 (0.42, 2.41)	0.99
Fetal loss (spontaneous abortion or stillbirth)	2/177 (1.1)	2/254 (0.8)	1.31 (0.23, 7.47)	0.76	2.23 (0.45, 11.05)	0.32
Neonatal death (<28 days)	2/167 (1.2)	4/242 (1.7)	0.87 (0.18, 4.21)	0.86	0.89 (0.18, 4.31)	0.88
Adverse pregnancy outcome (fetal loss, LBW/SGA/PT)	31/177 (17.5)	47/254 (18.5)	0.88 (0.54, 1.45)	0.61	0.88 (0.48, 1.61)	0.67
.						

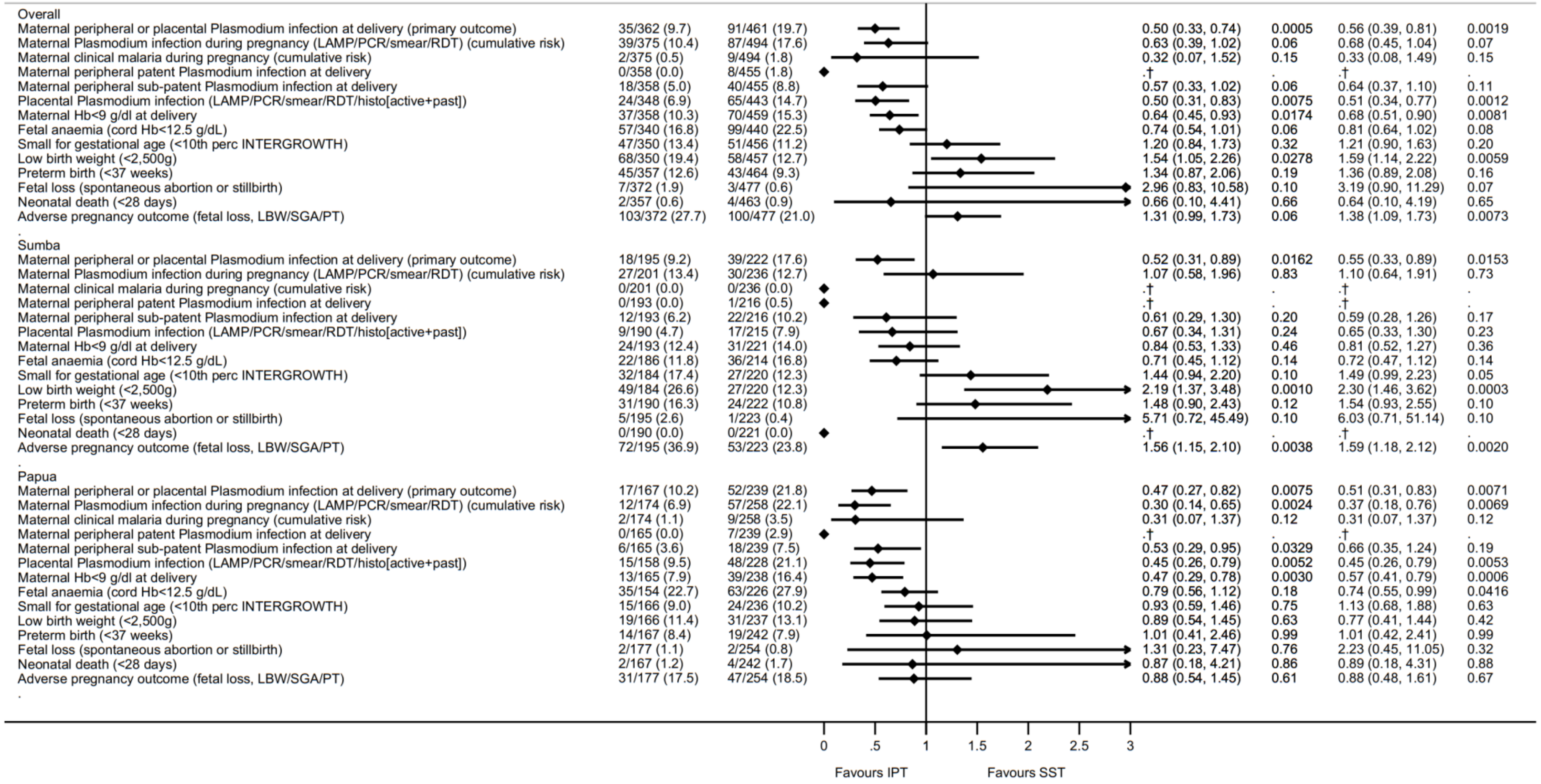

RR=Relative Risk, IRR=Incidence rate ratio, Adjusted RR or IRR obtained using the same covariates as in Figure 2 of the main text. * Data represents n/N (%) except for the data for the incidence per 100 person-years which represent the number of women with an event, the number of events, the follow-up person time and in brackets, the incidence rate per 100 person-years. † RR/IRR and p-value could not be computed because of zero events in at least one of the arms.

eFigure 6: Malaria at delivery (primary outcome) and key secondary outcomes in the per protocol population (IST vs SST)

IST vs SST	IST n/N (%)	SST, n/N (%)	Crude RR (95% CI)	P-value	Adjusted RR (95% CI)	P-value
Overall						
Maternal peripheral or placental Plasmodium infection at delivery (primary outcome)	61/519 (11.8)	91/461 (19.7)	0.57 (0.39, 0.83)	0.0034	0.71 (0.52, 0.96)	0.0250
Maternal Plasmodium infection during pregnancy (LAMP/PCR/smear/RDT) (cumulative risk)	84/556 (15.1)	87/494 (17.6)	0.84 (0.56, 1.25)	0.39	1.06 (0.70, 1.60)	0.78
Maternal clinical malaria during pregnancy (cumulative risk)	6/556 (1.1)	9/494 (1.8)	0.59 (0.18, 1.89)	0.37	0.73 (0.28, 1.91)	0.52
Maternal peripheral patent Plasmodium infection at delivery	8/514 (1.6)	8/455 (1.8)	0.89 (0.33, 2.43)	0.82	1.04 (0.25, 4.28)	0.95
Maternal peripheral sub-patent Plasmodium infection at delivery	21/514 (4.1)	40/455 (8.8)	0.48 (0.30, 0.76)	0.0017	0.68 (0.41, 1.12)	0.13
Placental Plasmodium infection (LAMP/PCR/smear/RDT/histo[active+past])	63/508 (12.4)	65/443 (14.7)	0.82 (0.53, 1.28)	0.39	0.85 (0.67, 1.08)	0.18
Maternal Hb<9 g/dl at delivery	65/516 (12.6)	70/459 (15.3)	0.76 (0.57, 1.01)	0.06	0.88 (0.72, 1.08)	0.23
Fetal anaemia (cord Hb<12.5 g/dL)	97/497 (19.5)	99/440 (22.5)	0.86 (0.64, 1.16)	0.32	0.87 (0.69, 1.09)	0.22
Small for gestational age (<10th perc INTERGROWTH)	51/501 (10.2)	51/456 (11.2)	0.91 (0.64, 1.29)	0.59	0.92 (0.69, 1.23)	0.57
Low birth weight (<2,500g)	61/504 (12.1)	58/457 (12.7)	0.99 (0.69, 1.42)	0.95	1.03 (0.74, 1.44)	0.86
Preterm birth (<37 weeks)	54/514 (10.5)	43/464 (9.3)	1.18 (0.77, 1.83)	0.45	1.10 (0.75, 1.61)	0.65
Fetal loss (spontaneous abortion or stillbirth)	11/530 (2.1)	3/477 (0.6)	3.27 (0.91, 11.74)	0.07	4.39 (1.13, 17.03)	0.0324
Neonatal death (<28 days)	10/513 (1.9)	4/463 (0.9)	2.20 (0.89, 5.46)	0.09	1.85 (0.80, 4.27)	0.15
Adverse pregnancy outcome (fetal loss, LBW/SGA/PT)	117/530 (22.1)	100/477 (21.0)	1.09 (0.82, 1.43)	0.56	1.08 (0.86, 1.36)	0.52
.						
Sumba						
Maternal peripheral or placental Plasmodium infection at delivery (primary outcome)	15/228 (6.6)	39/222 (17.6)	0.38 (0.18, 0.78)	0.0085	0.40 (0.20, 0.79)	0.0089
Maternal Plasmodium infection during pregnancy (LAMP/PCR/smear/RDT) (cumulative risk)	37/252 (14.7)	30/236 (12.7)	1.17 (0.66, 2.07)	0.59	1.24 (0.76, 2.03)	0.38
Maternal clinical malaria during pregnancy (cumulative risk)	1/252 (0.4)	0/236 (0.0)	.†	.	.†	.
Maternal peripheral patent Plasmodium infection at delivery	1/225 (0.4)	1/216 (0.5)	0.98 (0.06, 14.75)	0.99	0.98 (0.06, 14.75)	0.99
Maternal peripheral sub-patent Plasmodium infection at delivery	4/225 (1.8)	22/216 (10.2)	0.18 (0.07, 0.47)	0.0005	0.19 (0.06, 0.53)	0.0017
Placental Plasmodium infection (LAMP/PCR/smear/RDT/histo[active+past])	13/224 (5.8)	17/215 (7.9)	0.82 (0.45, 1.51)	0.53	0.74 (0.44, 1.26)	0.27
Maternal Hb<9 g/dl at delivery	35/227 (15.4)	31/221 (14.0)	1.08 (0.80, 1.45)	0.63	1.05 (0.76, 1.47)	0.76
Fetal anaemia (cord Hb<12.5 g/dL)	33/220 (15.0)	36/214 (16.8)	0.89 (0.63, 1.25)	0.49	0.95 (0.65, 1.39)	0.78
Small for gestational age (<10th perc INTERGROWTH)	28/218 (12.8)	27/220 (12.3)	1.05 (0.66, 1.66)	0.84	1.00 (0.64, 1.54)	0.98
Low birth weight (<2,500g)	36/220 (16.4)	27/220 (12.3)	1.34 (0.85, 2.13)	0.21	1.47 (0.94, 2.31)	0.09
Preterm birth (<37 weeks)	38/226 (16.8)	24/222 (10.8)	1.56 (0.97, 2.50)	0.07	1.46 (0.87, 2.44)	0.15
Fetal loss (spontaneous abortion or stillbirth)	5/231 (2.2)	1/223 (0.4)	5.04 (0.62, 40.91)	0.13	3.99 (0.46, 34.49)	0.21
Neonatal death (<28 days)	8/225 (3.6)	0/221 (0.0)	.†	.	.†	.
Adverse pregnancy outcome (fetal loss, LBW/SGA/PT)	71/231 (30.7)	53/223 (23.8)	1.30 (0.96, 1.75)	0.09	1.23 (0.91, 1.66)	0.19
.						
Papua						
Maternal peripheral or placental Plasmodium infection at delivery (primary outcome)	46/291 (15.8)	52/239 (21.8)	0.73 (0.53, 1.00)	0.05	0.87 (0.67, 1.14)	0.31
Maternal Plasmodium infection during pregnancy (LAMP/PCR/smear/RDT) (cumulative risk)	47/304 (15.5)	57/258 (22.1)	0.60 (0.35, 1.02)	0.06	0.91 (0.50, 1.66)	0.75
Maternal clinical malaria during pregnancy (cumulative risk)	5/304 (1.6)	9/258 (3.5)	0.51 (0.20, 1.31)	0.16	0.51 (0.20, 1.31)	0.16
Maternal peripheral patent Plasmodium infection at delivery	7/289 (2.4)	7/239 (2.9)	0.81 (0.36, 1.86)	0.63	0.81 (0.36, 1.86)	0.63
Maternal peripheral sub-patent Plasmodium infection at delivery	17/289 (5.9)	18/239 (7.5)	0.86 (0.56, 1.32)	0.47	1.31 (0.90, 1.91)	0.16
Placental Plasmodium infection (LAMP/PCR/smear/RDT/histo[active+past])	50/284 (17.6)	48/228 (21.1)	0.84 (0.61, 1.15)	0.27	0.84 (0.60, 1.16)	0.28
Maternal Hb<9 g/dl at delivery	30/289 (10.4)	39/238 (16.4)	0.62 (0.44, 0.89)	0.0086	0.84 (0.66, 1.07)	0.16
Fetal anaemia (cord Hb<12.5 g/dL)	64/277 (23.1)	63/226 (27.9)	0.81 (0.56, 1.18)	0.28	0.75 (0.55, 1.02)	0.07
Small for gestational age (<10th perc INTERGROWTH)	23/283 (8.1)	24/236 (10.2)	0.77 (0.49, 1.19)	0.24	0.90 (0.55, 1.46)	0.66
Low birth weight (<2,500g)	25/284 (8.8)	31/237 (13.1)	0.67 (0.41, 1.11)	0.12	0.55 (0.26, 1.13)	0.10
Preterm birth (<37 weeks)	16/288 (5.6)	19/242 (7.9)	0.69 (0.37, 1.28)	0.24	0.69 (0.37, 1.28)	0.24
Fetal loss (spontaneous abortion or stillbirth)	6/299 (2.0)	2/254 (0.8)	2.48 (0.47, 13.01)	0.28	5.50 (1.31, 23.02)	0.0197
Neonatal death (<28 days)	2/288 (0.7)	4/242 (1.7)	0.50 (0.20, 1.25)	0.13	0.52 (0.21, 1.28)	0.15
Adverse pregnancy outcome (fetal loss, LBW/SGA/PT)	46/299 (15.4)	47/254 (18.5)	0.82 (0.52, 1.30)	0.40	0.84 (0.45, 1.57)	0.59
.						

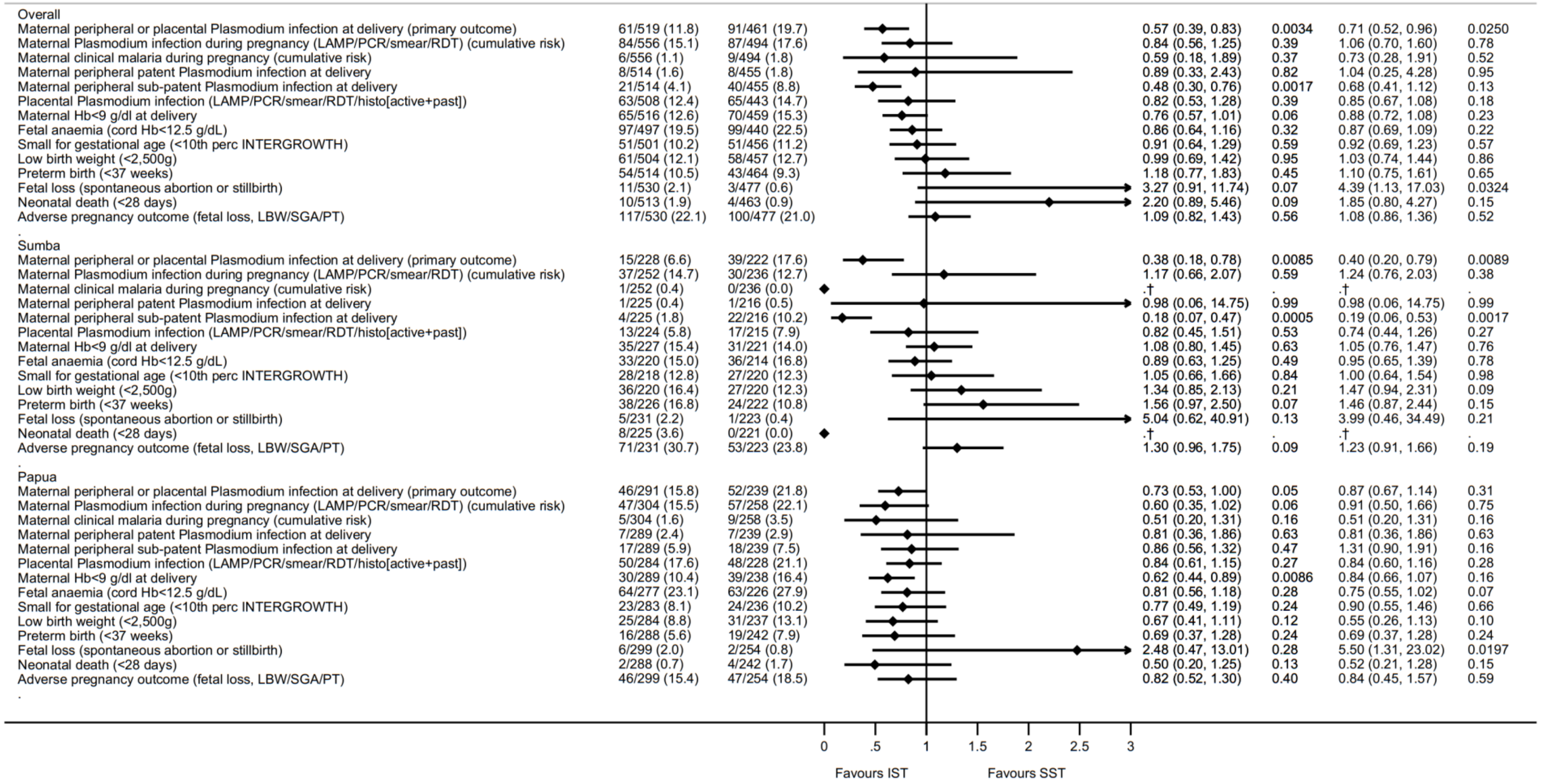

RR=Relative Risk, IRR=Incidence rate ratio, Adjusted RR or IRR obtained using the same covariates as in Figure 2 of the main text. * Data represents n/N (%) except for the data for the incidence per 100 person-years which represent the number of women with an event, the number of events, the follow-up person time and in brackets, the incidence rate per 100 person-years. † RR/IRR and p-value could not be computed because of zero events in at least one of the arms.

eFigure 7: Malaria at delivery (primary outcome) and key secondary outcomes in the per protocol population (IPT vs IST)

IPT vs IST	IPT n/N (%)	IST, n/N (%)	Crude RR (95% CI)	P-value	Adjusted RR (95% CI)	P-value
Overall						
Maternal peripheral or placental Plasmodium infection at delivery (primary outcome)	35/362 (9.7)	61/519 (11.8)	0.87 (0.55, 1.37)	0.54	0.79 (0.52, 1.21)	0.28
Maternal Plasmodium infection during pregnancy (LAMP/PCR/smear/RDT) (cumulative risk)	39/375 (10.4)	84/556 (15.1)	0.75 (0.49, 1.15)	0.19	0.65 (0.41, 1.02)	0.06
Maternal clinical malaria during pregnancy (cumulative risk)	2/375 (0.5)	6/556 (1.1)	0.55 (0.11, 2.73)	0.46	0.46 (0.11, 1.94)	0.29
Maternal peripheral patent Plasmodium infection at delivery	0/358 (0.0)	8/514 (1.6)	.†	.	.†	.
Maternal peripheral sub-patent Plasmodium infection at delivery	18/358 (5.0)	21/514 (4.1)	1.21 (0.67, 2.20)	0.53	0.94 (0.53, 1.66)	0.83
Placental Plasmodium infection (LAMP/PCR/smear/RDT/histo[active+past])	24/348 (6.9)	63/508 (12.4)	0.61 (0.36, 1.06)	0.08	0.60 (0.39, 0.93)	0.0213
Maternal Hb<9 g/dl at delivery	37/358 (10.3)	65/516 (12.6)	0.85 (0.58, 1.24)	0.40	0.77 (0.56, 1.04)	0.09
Fetal anaemia (cord Hb<12.5 g/dL)	57/340 (16.8)	97/497 (19.5)	0.86 (0.62, 1.19)	0.36	0.94 (0.72, 1.22)	0.62
Small for gestational age (<10th perc INTERGROWTH)	47/350 (13.4)	51/501 (10.2)	1.33 (0.90, 1.96)	0.16	1.32 (0.97, 1.79)	0.08
Low birth weight (<2,500g)	68/350 (19.4)	61/504 (12.1)	1.56 (1.11, 2.18)	0.0102	1.55 (1.23, 1.95)	0.0002
Preterm birth (<37 weeks)	45/357 (12.6)	54/514 (10.5)	1.13 (0.73, 1.74)	0.58	1.24 (0.80, 1.92)	0.33
Fetal loss (spontaneous abortion or stillbirth)	7/372 (1.9)	11/530 (2.1)	0.90 (0.35, 2.30)	0.83	0.73 (0.27, 1.98)	0.53
Neonatal death (<28 days)	2/357 (0.6)	10/513 (1.9)	0.30 (0.05, 1.93)	0.20	0.35 (0.04, 2.83)	0.32
Adverse pregnancy outcome (fetal loss, LBW/SGA/PT)	103/372 (27.7)	117/530 (22.1)	1.20 (0.90, 1.61)	0.21	1.27 (1.01, 1.60)	0.0384
.						
Sumba						
Maternal peripheral or placental Plasmodium infection at delivery (primary outcome)	18/195 (9.2)	15/228 (6.6)	1.39 (0.63, 3.10)	0.42	1.37 (0.63, 2.98)	0.43
Maternal Plasmodium infection during pregnancy (LAMP/PCR/smear/RDT) (cumulative risk)	27/201 (13.4)	37/252 (14.7)	0.91 (0.56, 1.50)	0.71	0.89 (0.52, 1.51)	0.65
Maternal clinical malaria during pregnancy (cumulative risk)	0/201 (0.0)	1/252 (0.4)	.†	.	.†	.
Maternal peripheral patent Plasmodium infection at delivery	0/193 (0.0)	1/225 (0.4)	.†	.	.†	.
Maternal peripheral sub-patent Plasmodium infection at delivery	12/193 (6.2)	4/225 (1.8)	3.46 (1.12, 10.71)	0.0312	3.22 (0.93, 11.10)	0.06
Placental Plasmodium infection (LAMP/PCR/smear/RDT/histo[active+past])	9/190 (4.7)	13/224 (5.8)	0.81 (0.44, 1.49)	0.50	0.88 (0.45, 1.70)	0.70
Maternal Hb<9 g/dl at delivery	24/193 (12.4)	35/227 (15.4)	0.78 (0.50, 1.22)	0.27	0.77 (0.48, 1.25)	0.29
Fetal anaemia (cord Hb<12.5 g/dL)	22/186 (11.8)	33/220 (15.0)	0.80 (0.47, 1.35)	0.40	0.76 (0.44, 1.33)	0.34
Small for gestational age (<10th perc INTERGROWTH)	32/184 (17.4)	28/218 (12.8)	1.37 (0.84, 2.23)	0.20	1.50 (0.90, 2.49)	0.12
Low birth weight (<2,500g)	49/184 (26.6)	36/220 (16.4)	1.63 (1.19, 2.24)	0.0026	1.56 (1.18, 2.07)	0.0019
Preterm birth (<37 weeks)	31/190 (16.3)	38/226 (16.8)	0.95 (0.66, 1.38)	0.79	1.05 (0.68, 1.63)	0.82
Fetal loss (spontaneous abortion or stillbirth)	5/195 (2.6)	5/231 (2.2)	1.13 (0.36, 3.55)	0.83	1.51 (0.33, 6.96)	0.60
Neonatal death (<28 days)	0/190 (0.0)	8/225 (3.6)	.†	.	.†	.
Adverse pregnancy outcome (fetal loss, LBW/SGA/PT)	72/195 (36.9)	71/231 (30.7)	1.20 (0.93, 1.54)	0.16	1.29 (0.98, 1.72)	0.07
.						
Papua						
Maternal peripheral or placental Plasmodium infection at delivery (primary outcome)	17/167 (10.2)	46/291 (15.8)	0.64 (0.38, 1.10)	0.11	0.58 (0.34, 1.01)	0.06
Maternal Plasmodium infection during pregnancy (LAMP/PCR/smear/RDT) (cumulative risk)	12/174 (6.9)	47/304 (15.5)	0.51 (0.24, 1.07)	0.07	0.41 (0.20, 0.81)	0.0109
Maternal clinical malaria during pregnancy (cumulative risk)	2/174 (1.1)	5/304 (1.6)	0.68 (0.13, 3.44)	0.64	0.68 (0.13, 3.44)	0.64
Maternal peripheral patent Plasmodium infection at delivery	0/165 (0.0)	7/289 (2.4)	.†	.	.†	.
Maternal peripheral sub-patent Plasmodium infection at delivery	6/165 (3.6)	17/289 (5.9)	0.62 (0.39, 0.98)	0.0427	0.50 (0.25, 1.01)	0.05
Placental Plasmodium infection (LAMP/PCR/smear/RDT/histo[active+past])	15/158 (9.5)	50/284 (17.6)	0.54 (0.29, 0.99)	0.0467	0.54 (0.29, 0.99)	0.0471
Maternal Hb<9 g/dl at delivery	13/165 (7.9)	30/289 (10.4)	0.76 (0.46, 1.27)	0.29	0.68 (0.48, 0.97)	0.0312
Fetal anaemia (cord Hb<12.5 g/dL)	35/154 (22.7)	64/277 (23.1)	0.98 (0.70, 1.36)	0.88	0.98 (0.70, 1.38)	0.91
Small for gestational age (<10th perc INTERGROWTH)	15/166 (9.0)	23/283 (8.1)	1.21 (0.80, 1.83)	0.37	1.27 (0.81, 1.99)	0.30
Low birth weight (<2,500g)	19/166 (11.4)	25/284 (8.8)	1.32 (0.84, 2.08)	0.23	1.42 (0.86, 2.34)	0.18
Preterm birth (<37 weeks)	14/167 (8.4)	16/288 (5.6)	1.46 (0.62, 3.45)	0.39	1.46 (0.62, 3.42)	0.38
Fetal loss (spontaneous abortion or stillbirth)	2/177 (1.1)	6/299 (2.0)	0.53 (0.12, 2.35)	0.40	0.41 (0.12, 1.42)	0.16
Neonatal death (<28 days)	2/167 (1.2)	2/288 (0.7)	1.75 (0.31, 9.91)	0.53	1.73 (0.31, 9.70)	0.54
Adverse pregnancy outcome (fetal loss, LBW/SGA/PT)	31/177 (17.5)	46/299 (15.4)	1.07 (0.63, 1.81)	0.80	1.04 (0.62, 1.77)	0.87
.						

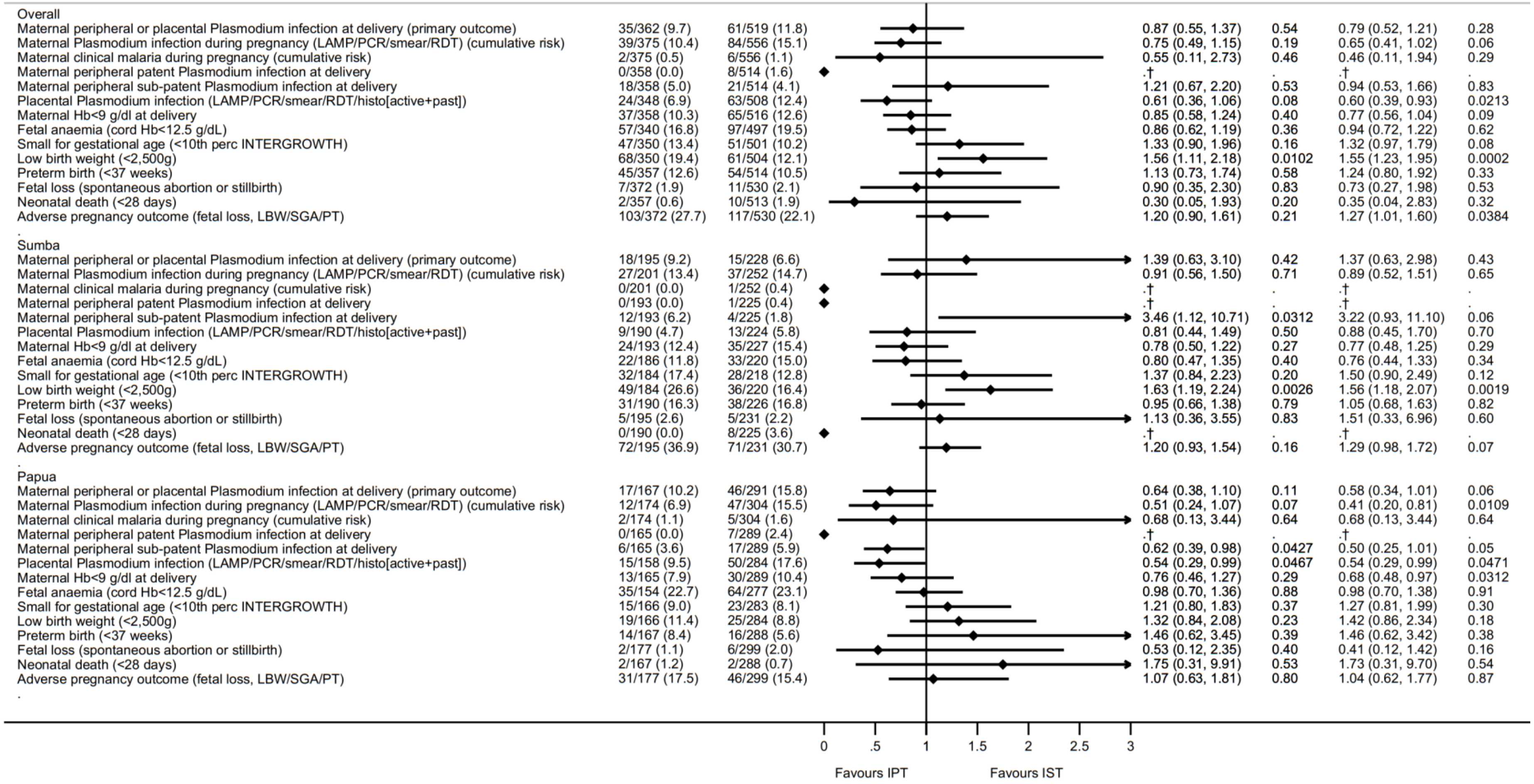

RR=Relative Risk, IRR=Incidence rate ratio, Adjusted RR or IRR obtained using the same covariates as in Figure 2 of the main text. * Data represents n/N (%) except for the data for the incidence per 100 person-years which represent the number of women with an event, the number of events, the follow-up person time and in brackets, the incidence rate per 100 person-years. † RR/IRR and p-value could not be computed because of zero events in at least one of the arms.

eFigure 8: Malaria at delivery by treatment group and site in the intention-to-treat population (IPT vs SST)

IPT vs SST	IPT n/N (%)	SST, n/N (%)	Crude RR (95% CI)	P-value	Adjusted RR (95% CI)	P-value
**Overall**						
Maternal peripheral Plasmodium infection at delivery (RDT)	4/524 (0.8)	14/631 (2.2)	0.43 (0.11, 1.62)	0.21	0.47 (0.17, 1.28)	0.14
Maternal peripheral Plasmodium infection at delivery (smear)	2/524 (0.4)	23/630 (3.7)	0.13 (0.03, 0.56)	0.0065	0.11 (0.03, 0.43)	0.0013
Maternal peripheral Plasmodium infection at delivery (LAMP/PCR)	29/523 (5.5)	70/627 (11.2)	0.50 (0.33, 0.77)	0.0014	0.54 (0.35, 0.81)	0.0031
Maternal peripheral Plasmodium infection at delivery (LAMP/PCR/smear/RDT)	32/525 (6.1)	83/633 (13.1)	0.50 (0.32, 0.79)	0.0028	0.52 (0.34, 0.78)	0.0017
Maternal peripheral P.falciparum infection at delivery (PCR confirmed)‡	23/524 (4.4)	58/633 (9.2)	0.51 (0.29, 0.91)	0.0214	0.53 (0.30, 0.93)	0.0257
Maternal peripheral P.vivax mono-infection at delivery (PCR confirmed)‡	8/491 (1.6)	23/574 (4.0)	0.35 (0.16, 0.73)	0.0057	0.41 (0.19, 0.87)	0.0196
Maternal peripheral patent Plasmodium infection at delivery	1/523 (0.2)	14/627 (2.2)	0.09 (0.01, 0.60)	0.0134	0.09 (0.01, 0.58)	0.0113
Maternal peripheral sub-patent Plasmodium infection at delivery	28/523 (5.4)	56/627 (8.9)	0.60 (0.39, 0.93)	0.0219	0.65 (0.43, 0.99)	0.0434
Maternal peripheral or placental patent Plasmodium infection at delivery	3/528 (0.6)	18/633 (2.8)	0.22 (0.07, 0.71)	0.0119	0.22 (0.08, 0.65)	0.0059
Maternal peripheral or placental sub-patent Plasmodium infection at delivery	55/528 (10.4)	98/633 (15.5)	0.67 (0.49, 0.92)	0.0141	0.73 (0.56, 0.97)	0.0281
.						
**Sumba**						
Maternal peripheral Plasmodium infection at delivery (RDT)	0/254 (0.0)	0/289 (0.0)	.†	.	.†	.
Maternal peripheral Plasmodium infection at delivery (smear)	0/254 (0.0)	1/288 (0.3)	.†	.	.†	.
Maternal peripheral Plasmodium infection at delivery (LAMP/PCR)	17/253 (6.7)	29/284 (10.2)	0.66 (0.36, 1.22)	0.18	0.66 (0.36, 1.21)	0.18
Maternal peripheral Plasmodium infection at delivery (LAMP/PCR/smear/RDT)	17/255 (6.7)	29/290 (10.0)	0.67 (0.36, 1.24)	0.20	0.67 (0.36, 1.24)	0.20
Maternal peripheral P.falciparum infection at delivery (PCR confirmed)‡	13/254 (5.1)	21/290 (7.2)	0.72 (0.33, 1.55)	0.39	0.75 (0.35, 1.59)	0.45
Maternal peripheral P.vivax mono-infection at delivery (PCR confirmed)‡	4/225 (1.8)	8/249 (3.2)	0.54 (0.15, 1.90)	0.34	0.51 (0.15, 1.68)	0.27
Maternal peripheral patent Plasmodium infection at delivery	0/253 (0.0)	1/284 (0.4)	.†	.	.†	.
Maternal peripheral sub-patent Plasmodium infection at delivery	17/253 (6.7)	28/284 (9.9)	0.68 (0.37, 1.26)	0.22	0.68 (0.37, 1.25)	0.21
Maternal peripheral or placental patent Plasmodium infection at delivery	0/256 (0.0)	1/290 (0.3)	.†	.	.†	.
Maternal peripheral or placental sub-patent Plasmodium infection at delivery	29/256 (11.3)	45/290 (15.5)	0.73 (0.46, 1.14)	0.16	0.76 (0.50, 1.16)	0.20
.						
**Papua**						
Maternal peripheral Plasmodium infection at delivery (RDT)	4/270 (1.5)	14/342 (4.1)	0.39 (0.12, 1.24)	0.11	0.50 (0.18, 1.42)	0.19
Maternal peripheral Plasmodium infection at delivery (smear)	2/270 (0.7)	22/342 (6.4)	0.11 (0.03, 0.44)	0.0018	0.11 (0.03, 0.42)	0.0014
Maternal peripheral Plasmodium infection at delivery (LAMP/PCR)	12/270 (4.4)	41/343 (12.0)	0.37 (0.25, 0.56)	<.0001	0.44 (0.29, 0.67)	<.0001
Maternal peripheral Plasmodium infection at delivery (LAMP/PCR/smear/RDT)	15/270 (5.6)	54/343 (15.7)	0.35 (0.21, 0.60)	<.0001	0.41 (0.27, 0.61)	<.0001
Maternal peripheral P.falciparum infection at delivery (PCR confirmed)‡	10/270 (3.7)	37/343 (10.8)	0.35 (0.16, 0.73)	0.0053	0.43 (0.22, 0.85)	0.0145
Maternal peripheral P.vivax mono-infection at delivery (PCR confirmed)‡	4/266 (1.5)	15/325 (4.6)	0.26 (0.11, 0.66)	0.0043	0.33 (0.13, 0.85)	0.0211
Maternal peripheral patent Plasmodium infection at delivery	1/270 (0.4)	13/343 (3.8)	0.10 (0.02, 0.60)	0.0123	0.09 (0.02, 0.56)	0.0099
Maternal peripheral sub-patent Plasmodium infection at delivery	11/270 (4.1)	28/343 (8.2)	0.51 (0.32, 0.81)	0.0046	0.65 (0.42, 1.00)	0.0474
Maternal peripheral or placental patent Plasmodium infection at delivery	3/272 (1.1)	17/343 (5.0)	0.21 (0.07, 0.64)	0.0056	0.21 (0.07, 0.62)	0.0047
Maternal peripheral or placental sub-patent Plasmodium infection at delivery	26/272 (9.6)	53/343 (15.5)	0.62 (0.41, 0.94)	0.0255	0.73 (0.50, 1.06)	0.10
.						

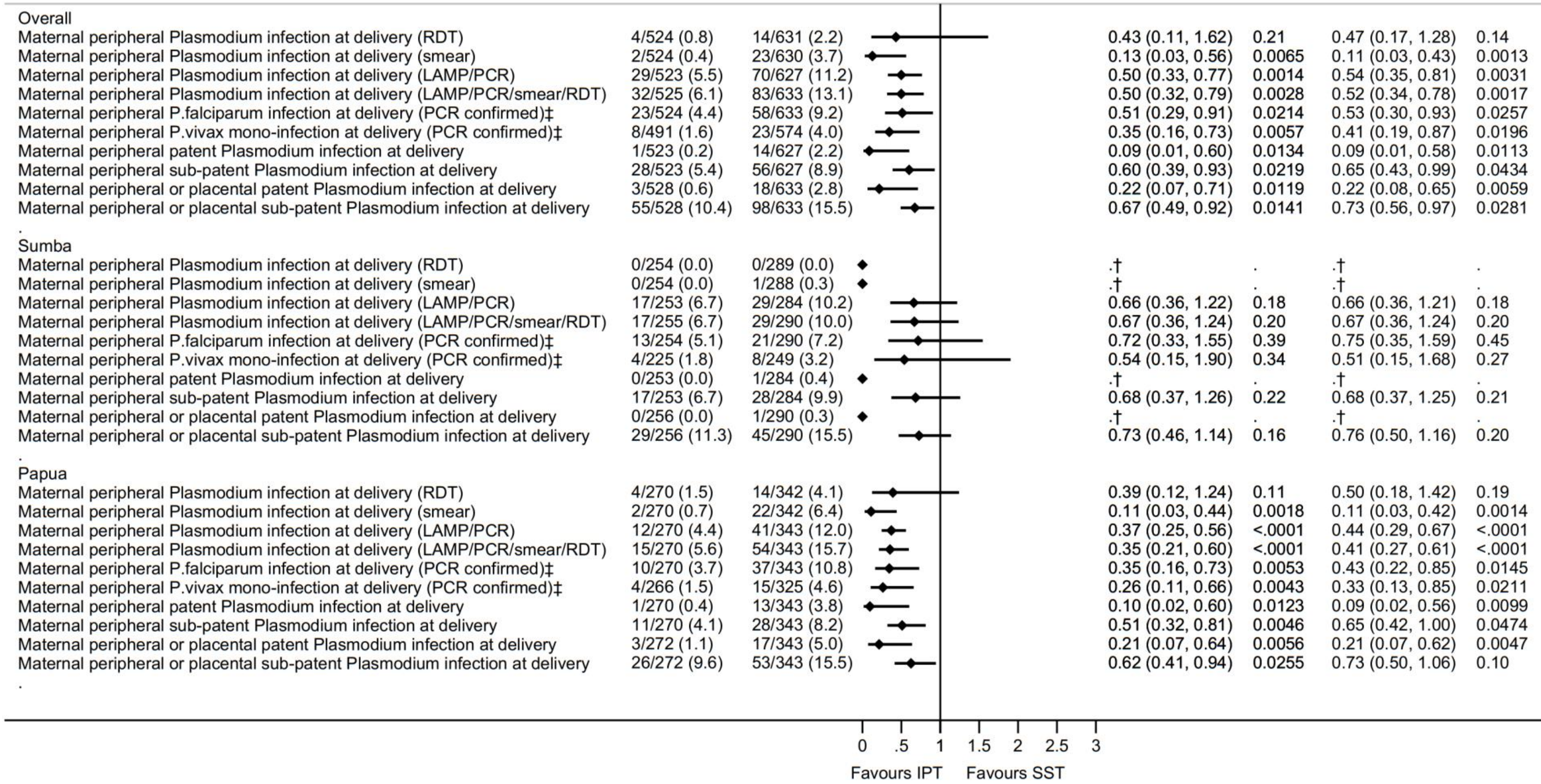

RR=Relative Risk. * Data for represents the n/N (%). Adjusted RR obtained using the same covariates as outlined in Figure 2, under post-hoc adjusted analysis. † RR and p-value could not be computed because of zero events in at least one of the arms. ‡ outcome variable defined post-hoc.

eFigure 9: Malaria at delivery by treatment group and site in the intention-to-treat population (IST vs SST)

IST vs SST	IST n/N (%)	SST, n/N (%)	Crude RR (95% CI)	P-value	Adjusted RR (95% CI)	P-value
**Overall**						
Maternal peripheral Plasmodium infection at delivery (RDT)	7/710 (1.0)	14/631 (2.2)	0.49 (0.14, 1.66)	0.25	0.75 (0.28, 1.99)	0.57
Maternal peripheral Plasmodium infection at delivery (smear)	13/709 (1.8)	23/630 (3.7)	0.52 (0.22, 1.23)	0.14	0.58 (0.31, 1.10)	0.10
Maternal peripheral Plasmodium infection at delivery (LAMP/PCR)	39/708 (5.5)	70/627 (11.2)	0.49 (0.33, 0.72)	0.0002	0.65 (0.45, 0.95)	0.0258
Maternal peripheral Plasmodium infection at delivery (LAMP/PCR/smear/RDT)	43/710 (6.1)	83/633 (13.1)	0.44 (0.29, 0.67)	<.0001	0.58 (0.40, 0.85)	0.0055
Maternal peripheral P.falciparum infection at delivery (PCR confirmed)‡	32/710 (4.5)	58/633 (9.2)	0.45 (0.28, 0.74)	0.0016	0.59 (0.35, 0.99)	0.0453
Maternal peripheral P.vivax mono-infection at delivery (PCR confirmed)‡	10/650 (1.5)	23/574 (4.0)	0.31 (0.19, 0.53)	<.0001	0.47 (0.30, 0.73)	0.0007
Maternal peripheral patent Plasmodium infection at delivery	11/708 (1.6)	14/627 (2.2)	0.68 (0.28, 1.65)	0.39	0.82 (0.36, 1.88)	0.64
Maternal peripheral sub-patent Plasmodium infection at delivery	28/708 (4.0)	56/627 (8.9)	0.49 (0.35, 0.67)	<.0001	0.63 (0.42, 0.96)	0.0295
Maternal peripheral or placental patent Plasmodium infection at delivery	12/713 (1.7)	18/633 (2.8)	0.57 (0.24, 1.37)	0.21	0.70 (0.33, 1.51)	0.36
Maternal peripheral or placental sub-patent Plasmodium infection at delivery	65/713 (9.1)	98/633 (15.5)	0.59 (0.45, 0.78)	0.0002	0.71 (0.54, 0.94)	0.0159
.						
**Sumba**						
Maternal peripheral Plasmodium infection at delivery (RDT)	1/284 (0.4)	0/289 (0.0)	.†	.	.†	.
Maternal peripheral Plasmodium infection at delivery (smear)	2/284 (0.7)	1/288 (0.3)	2.03 (0.20, 21.14)	0.55	2.03 (0.20, 21.14)	0.55
Maternal peripheral Plasmodium infection at delivery (LAMP/PCR)	7/282 (2.5)	29/284 (10.2)	0.24 (0.13, 0.46)	<.0001	0.23 (0.12, 0.47)	<.0001
Maternal peripheral Plasmodium infection at delivery (LAMP/PCR/smear/RDT)	8/284 (2.8)	29/290 (10.0)	0.28 (0.14, 0.56)	0.0003	0.27 (0.13, 0.56)	0.0004
Maternal peripheral P.falciparum infection at delivery (PCR confirmed)‡	6/284 (2.1)	21/290 (7.2)	0.29 (0.12, 0.71)	0.0068	0.25 (0.10, 0.62)	0.0026
Maternal peripheral P.vivax mono-infection at delivery (PCR confirmed)‡	2/234 (0.9)	8/249 (3.2)	0.27 (0.06, 1.25)	0.09	0.38 (0.08, 1.81)	0.22
Maternal peripheral patent Plasmodium infection at delivery	1/282 (0.4)	1/284 (0.4)	1.01 (0.07, 15.17)	0.99	1.01 (0.07, 15.17)	0.99
Maternal peripheral sub-patent Plasmodium infection at delivery	6/282 (2.1)	28/284 (9.9)	0.21 (0.11, 0.43)	<.0001	0.21 (0.10, 0.45)	<.0001
Maternal peripheral or placental patent Plasmodium infection at delivery	1/285 (0.4)	1/290 (0.3)	1.02 (0.07, 15.38)	0.99	1.02 (0.07, 15.38)	0.99
Maternal peripheral or placental sub-patent Plasmodium infection at delivery	18/285 (6.3)	45/290 (15.5)	0.41 (0.23, 0.72)	0.0021	0.41 (0.21, 0.77)	0.0062
.						
**Papua**						
Maternal peripheral Plasmodium infection at delivery (RDT)	6/426 (1.4)	14/342 (4.1)	0.34 (0.12, 1.00)	0.05	0.64 (0.27, 1.52)	0.32
Maternal peripheral Plasmodium infection at delivery (smear)	11/425 (2.6)	22/342 (6.4)	0.41 (0.21, 0.79)	0.0078	0.41 (0.21, 0.78)	0.0066
Maternal peripheral Plasmodium infection at delivery (LAMP/PCR)	32/426 (7.5)	41/343 (12.0)	0.67 (0.48, 0.94)	0.0216	0.91 (0.62, 1.35)	0.65
Maternal peripheral Plasmodium infection at delivery (LAMP/PCR/smear/RDT)	35/426 (8.2)	54/343 (15.7)	0.52 (0.34, 0.79)	0.0022	0.77 (0.50, 1.20)	0.25
Maternal peripheral P.falciparum infection at delivery (PCR confirmed)‡	26/426 (6.1)	37/343 (10.8)	0.56 (0.33, 0.94)	0.0287	0.93 (0.55, 1.58)	0.80
Maternal peripheral P.vivax mono-infection at delivery (PCR confirmed)‡	8/416 (1.9)	15/325 (4.6)	0.28 (0.16, 0.50)	<.0001	0.51 (0.35, 0.75)	0.0006
Maternal peripheral patent Plasmodium infection at delivery	10/426 (2.3)	13/343 (3.8)	0.66 (0.33, 1.31)	0.23	0.66 (0.33, 1.29)	0.22
Maternal peripheral sub-patent Plasmodium infection at delivery	22/426 (5.2)	28/343 (8.2)	0.66 (0.48, 0.89)	0.0076	1.02 (0.67, 1.54)	0.94
Maternal peripheral or placental patent Plasmodium infection at delivery	11/428 (2.6)	17/343 (5.0)	0.55 (0.28, 1.05)	0.07	0.54 (0.28, 1.04)	0.07
Maternal peripheral or placental sub-patent Plasmodium infection at delivery	47/428 (11.0)	53/343 (15.5)	0.72 (0.54, 0.95)	0.0219	0.92 (0.69, 1.24)	0.58
.						

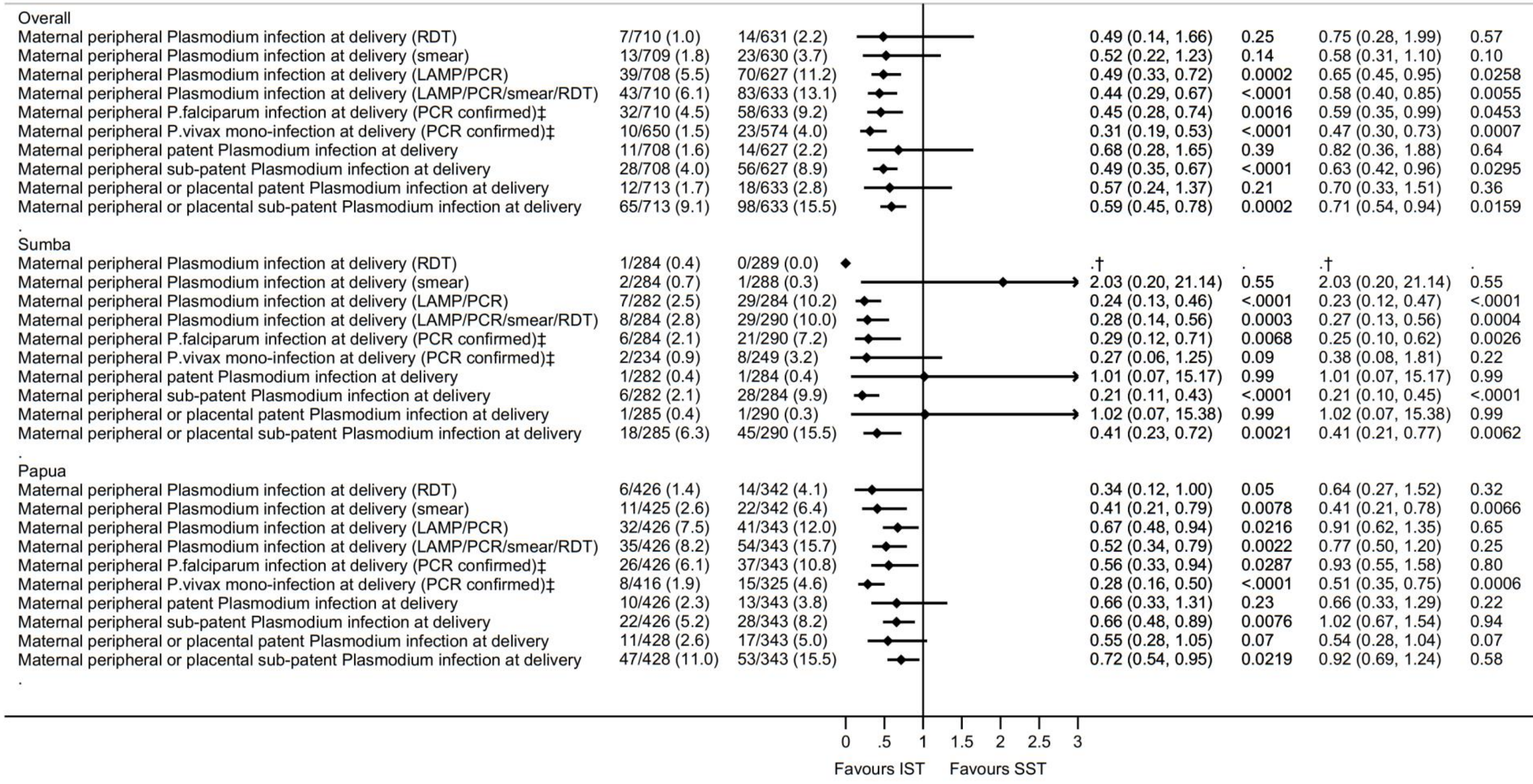

RR=Relative Risk. * Data for represents the n/N (%). Adjusted RR obtained using the same covariates as outlined in Figure 2, under post-hoc adjusted analysis. † RR and p-value could not be computed because of zero events in at least one of the arms. ‡ outcome variable defined post-hoc.

eFigure 10: Malaria at delivery by treatment group and site in the intention-to-treat population (IPT vs IST)

IPT vs IST	IPT n/N (%)	IST, n/N (%)	Crude RR (95% CI)	P-value	Adjusted RR (95% CI)	P-value
**Overall**						
Maternal peripheral Plasmodium infection at delivery (RDT)	4/524 (0.8)	7/710 (1.0)	0.89 (0.28, 2.79)	0.84	0.62 (0.21, 1.88)	0.40
Maternal peripheral Plasmodium infection at delivery (smear)	2/524 (0.4)	13/709 (1.8)	0.25 (0.06, 1.06)	0.06	0.19 (0.05, 0.83)	0.0272
Maternal peripheral Plasmodium infection at delivery (LAMP/PCR)	29/523 (5.5)	39/708 (5.5)	1.02 (0.63, 1.66)	0.92	0.82 (0.53, 1.27)	0.37
Maternal peripheral Plasmodium infection at delivery (LAMP/PCR/smear/RDT)	32/525 (6.1)	43/710 (6.1)	1.14 (0.70, 1.87)	0.59	0.88 (0.57, 1.37)	0.57
Maternal peripheral P.falciparum infection at delivery (PCR confirmed)‡	23/524 (4.4)	32/710 (4.5)	1.13 (0.62, 2.07)	0.69	0.89 (0.50, 1.57)	0.69
Maternal peripheral P.vivax mono-infection at delivery (PCR confirmed)‡	8/491 (1.6)	10/650 (1.5)	1.10 (0.49, 2.47)	0.82	0.87 (0.39, 1.96)	0.75
Maternal peripheral patent Plasmodium infection at delivery	1/523 (0.2)	11/708 (1.6)	0.13 (0.02, 0.92)	0.0416	0.11 (0.02, 0.75)	0.0239
Maternal peripheral sub-patent Plasmodium infection at delivery	28/523 (5.4)	28/708 (4.0)	1.23 (0.81, 1.87)	0.33	1.03 (0.68, 1.56)	0.89
Maternal peripheral or placental patent Plasmodium infection at delivery	3/528 (0.6)	12/713 (1.7)	0.38 (0.11, 1.28)	0.12	0.32 (0.11, 0.96)	0.0418
Maternal peripheral or placental sub-patent Plasmodium infection at delivery	55/528 (10.4)	65/713 (9.1)	1.14 (0.81, 1.60)	0.46	1.03 (0.77, 1.40)	0.83
.						
**Sumba**						
Maternal peripheral Plasmodium infection at delivery (RDT)	0/254 (0.0)	1/284 (0.4)	.†	.	.†	.
Maternal peripheral Plasmodium infection at delivery (smear)	0/254 (0.0)	2/284 (0.7)	.†	.	.†	.
Maternal peripheral Plasmodium infection at delivery (LAMP/PCR)	17/253 (6.7)	7/282 (2.5)	2.73 (1.27, 5.90)	0.0105	2.81 (1.17, 6.76)	0.0207
Maternal peripheral Plasmodium infection at delivery (LAMP/PCR/smear/RDT)	17/255 (6.7)	8/284 (2.8)	2.37 (1.05, 5.34)	0.0372	2.48 (1.02, 6.04)	0.0449
Maternal peripheral P.falciparum infection at delivery (PCR confirmed)‡	13/254 (5.1)	6/284 (2.1)	2.45 (0.89, 6.72)	0.08	2.96 (1.08, 8.11)	0.0344
Maternal peripheral P.vivax mono-infection at delivery (PCR confirmed)‡	4/225 (1.8)	2/234 (0.9)	1.99 (0.37, 10.88)	0.43	1.35 (0.21, 8.78)	0.75
Maternal peripheral patent Plasmodium infection at delivery	0/253 (0.0)	1/282 (0.4)	.†	.	.†	.
Maternal peripheral sub-patent Plasmodium infection at delivery	17/253 (6.7)	6/282 (2.1)	3.19 (1.40, 7.29)	0.0060	3.30 (1.29, 8.43)	0.0125
Maternal peripheral or placental patent Plasmodium infection at delivery	0/256 (0.0)	1/285 (0.4)	.†	.	.†	.
Maternal peripheral or placental sub-patent Plasmodium infection at delivery	29/256 (11.3)	18/285 (6.3)	1.79 (0.95, 3.38)	0.07	1.87 (0.92, 3.81)	0.09
.						
**Papua**						
Maternal peripheral Plasmodium infection at delivery (RDT)	4/270 (1.5)	6/426 (1.4)	1.15 (0.40, 3.31)	0.80	0.78 (0.28, 2.17)	0.63
Maternal peripheral Plasmodium infection at delivery (smear)	2/270 (0.7)	11/425 (2.6)	0.27 (0.07, 1.14)	0.07	0.27 (0.07, 1.10)	0.07
Maternal peripheral Plasmodium infection at delivery (LAMP/PCR)	12/270 (4.4)	32/426 (7.5)	0.55 (0.38, 0.81)	0.0025	0.48 (0.30, 0.78)	0.0026
Maternal peripheral Plasmodium infection at delivery (LAMP/PCR/smear/RDT)	15/270 (5.6)	35/426 (8.2)	0.68 (0.43, 1.07)	0.09	0.53 (0.33, 0.83)	0.0061
Maternal peripheral P.falciparum infection at delivery (PCR confirmed)‡	10/270 (3.7)	26/426 (6.1)	0.62 (0.31, 1.24)	0.17	0.46 (0.22, 0.98)	0.0452
Maternal peripheral P.vivax mono-infection at delivery (PCR confirmed)‡	4/266 (1.5)	8/416 (1.9)	0.92 (0.33, 2.57)	0.88	0.64 (0.22, 1.85)	0.41
Maternal peripheral patent Plasmodium infection at delivery	1/270 (0.4)	10/426 (2.3)	0.15 (0.02, 0.99)	0.0485	0.14 (0.02, 0.93)	0.0420
Maternal peripheral sub-patent Plasmodium infection at delivery	11/270 (4.1)	22/426 (5.2)	0.78 (0.55, 1.11)	0.16	0.64 (0.38, 1.06)	0.08
Maternal peripheral or placental patent Plasmodium infection at delivery	3/272 (1.1)	11/428 (2.6)	0.39 (0.12, 1.25)	0.11	0.39 (0.12, 1.22)	0.11
Maternal peripheral or placental sub-patent Plasmodium infection at delivery	26/272 (9.6)	47/428 (11.0)	0.87 (0.59, 1.29)	0.49	0.79 (0.53, 1.19)	0.26
.						

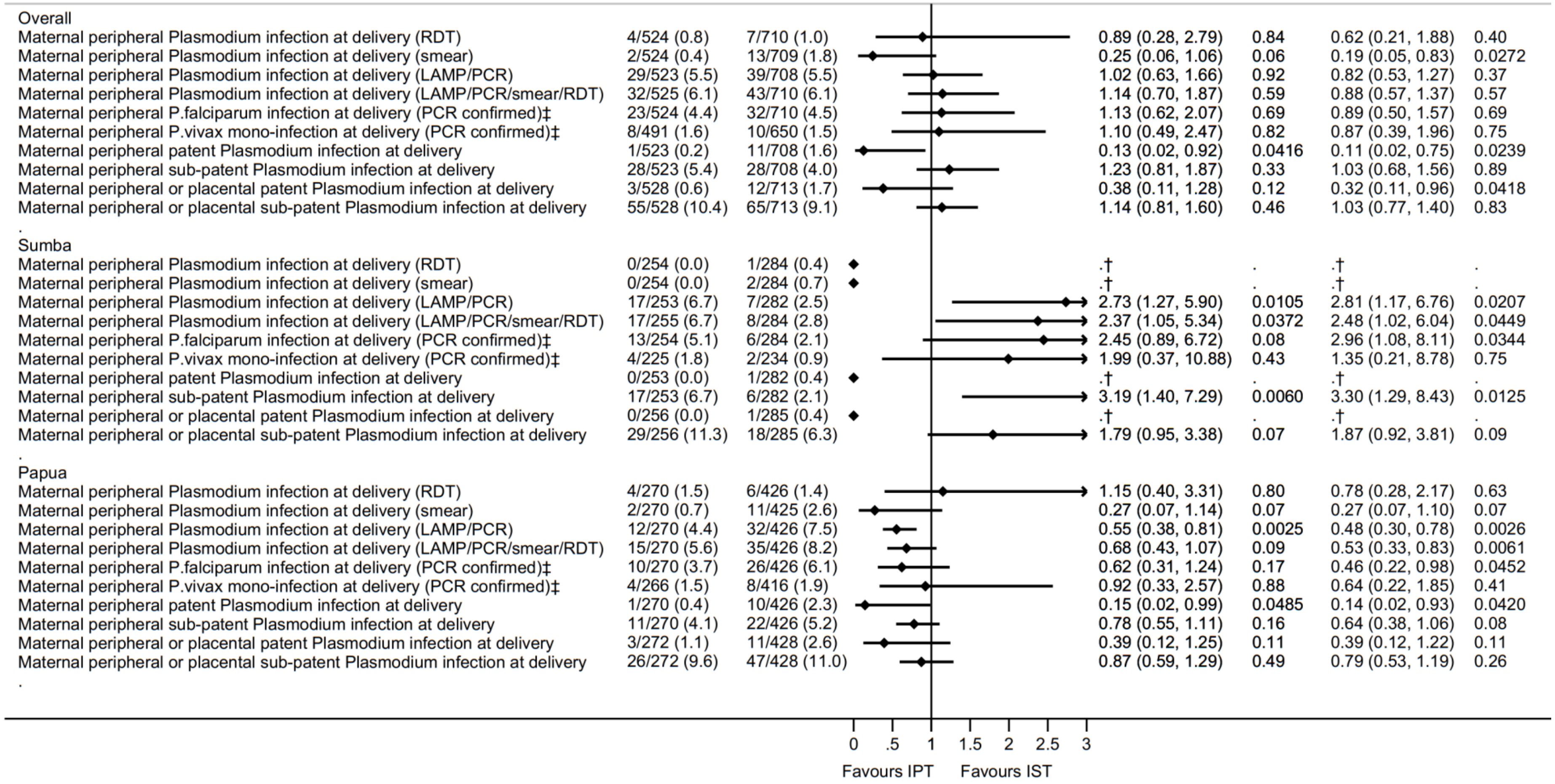

RR=Relative Risk. * Data for represents the n/N (%). Adjusted RR obtained using the same covariates as outlined in Figure 2, under post-hoc adjusted analysis. † RR and p-value could not be computed because of zero events in at least one of the arms. ‡ outcome variable defined post-hoc.

eFigure 11: Placental malaria by treatment group and site in the intention-to-treat population (IPT vs SST)

IPT vs SST	IPT n/N (%)	SST, n/N (%)	Crude RR (95% CI)	P-value	Adjusted RR (95% CI)	P-value
Overall						
Placental Plasmodium infection (LAMP/PCR/smear/RDT/histo[active])	35/500 (7.0)	69/610 (11.3)	0.63 (0.40, 1.00)	0.0496	0.72 (0.49, 1.04)	0.08
Placental Plasmodium infection (LAMP/PCR/smear/RDT/histo[active+past])	40/500 (8.0)	95/610 (15.6)	0.58 (0.36, 0.94)	0.0258	0.51 (0.35, 0.75)	0.0007
Placental malaria (smear)‡	3/498 (0.6)	13/605 (2.1)	0.33 (0.10, 1.16)	0.08	0.33 (0.11, 1.05)	0.06
Placental malaria (RDT:HRP2 or pLDH)‡	2/499 (0.4)	17/608 (2.8)	0.15 (0.04, 0.67)	0.0127	0.15 (0.04, 0.57)	0.0049
Placental malaria (LAMP/PCR)‡	20/492 (4.1)	36/603 (6.0)	0.68 (0.42, 1.09)	0.11	0.75 (0.45, 1.26)	0.28
Placental malaria (histology: active-any)	15/488 (3.1)	25/590 (4.2)	0.71 (0.38, 1.33)	0.29	0.80 (0.46, 1.39)	0.43
Placental malaria (histology: active-acute)	11/488 (2.3)	7/590 (1.2)	1.95 (0.85, 4.47)	0.11	2.43 (1.05, 5.67)	0.0390
Placental malaria (histology: past)	6/488 (1.2)	31/590 (5.3)	0.32 (0.13, 0.82)	0.0172	0.21 (0.10, 0.45)	<.0001
Placental malaria (histology: active-chronic)	4/488 (0.8)	18/590 (3.1)	0.28 (0.10, 0.74)	0.0103	0.25 (0.09, 0.68)	0.0065
Placental malaria (histology: active or past)	21/488 (4.3)	56/590 (9.5)	0.58 (0.31, 1.09)	0.09	0.44 (0.26, 0.76)	0.0029
.						
Sumba						
Placental Plasmodium infection (LAMP/PCR/smear/RDT/histo[active])	13/249 (5.2)	19/283 (6.7)	0.79 (0.41, 1.52)	0.47	0.82 (0.43, 1.56)	0.54
Placental Plasmodium infection (LAMP/PCR/smear/RDT/histo[active+past])	15/249 (6.0)	20/283 (7.1)	0.90 (0.49, 1.66)	0.74	0.94 (0.52, 1.72)	0.84
Placental malaria (smear)‡	0/249 (0.0)	0/281 (0.0)	.†	.	.†	.
Placental malaria (RDT:HRP2 or pLDH)‡	0/249 (0.0)	0/282 (0.0)	.†	.	.†	.
Placental malaria (LAMP/PCR)‡	9/242 (3.7)	13/278 (4.7)	0.80 (0.41, 1.57)	0.51	0.84 (0.43, 1.66)	0.62
Placental malaria (histology: active-any)	5/242 (2.1)	6/270 (2.2)	0.93 (0.28, 3.08)	0.90	0.90 (0.27, 2.98)	0.86
Placental malaria (histology: active-chronic)	1/242 (0.4)	2/270 (0.7)	0.56 (0.06, 5.71)	0.63	0.56 (0.06, 5.71)	0.63
Placental malaria (histology: past)	3/242 (1.2)	1/270 (0.4)	3.94 (0.44, 35.55)	0.22	4.37 (0.54, 35.25)	0.17
Placental malaria (histology: active-acute)	4/242 (1.7)	4/270 (1.5)	1.11 (0.26, 4.79)	0.88	1.07 (0.25, 4.62)	0.93
Placental malaria (histology: active or past)	8/242 (3.3)	7/270 (2.6)	1.31 (0.50, 3.44)	0.58	1.32 (0.51, 3.44)	0.57
.						
Papua						
Placental Plasmodium infection (LAMP/PCR/smear/RDT/histo[active])	22/251 (8.8)	50/327 (15.3)	0.57 (0.33, 1.00)	0.0481	0.69 (0.41, 1.17)	0.17
Placental Plasmodium infection (LAMP/PCR/smear/RDT/histo[active+past])	25/251 (10.0)	75/327 (22.9)	0.43 (0.26, 0.69)	0.0006	0.44 (0.27, 0.73)	0.0014
Placental malaria (smear)‡	3/249 (1.2)	13/324 (4.0)	0.30 (0.10, 0.92)	0.0351	0.29 (0.10, 0.87)	0.0263
Placental malaria (RDT:HRP2 or pLDH)‡	2/250 (0.8)	17/326 (5.2)	0.15 (0.04, 0.54)	0.0034	0.17 (0.04, 0.67)	0.0113
Placental malaria (LAMP/PCR)‡	11/250 (4.4)	23/325 (7.1)	0.61 (0.31, 1.21)	0.16	0.79 (0.36, 1.73)	0.56
Placental malaria (histology: active-any)	10/246 (4.1)	19/320 (5.9)	0.68 (0.34, 1.37)	0.28	0.70 (0.37, 1.35)	0.29
Placental malaria (histology: active-acute)	7/246 (2.8)	3/320 (0.9)	3.00 (1.15, 7.83)	0.0244	3.01 (1.17, 7.72)	0.0222
Placental malaria (histology: active-chronic)	3/246 (1.2)	16/320 (5.0)	0.23 (0.08, 0.72)	0.0116	0.22 (0.07, 0.69)	0.0090
Placental malaria (histology: past)	3/246 (1.2)	30/320 (9.4)	0.12 (0.05, 0.27)	<.0001	0.11 (0.05, 0.26)	<.0001
Placental malaria (histology: active or past)	13/246 (5.3)	49/320 (15.3)	0.32 (0.18, 0.55)	<.0001	0.31 (0.18, 0.55)	<.0001
.						

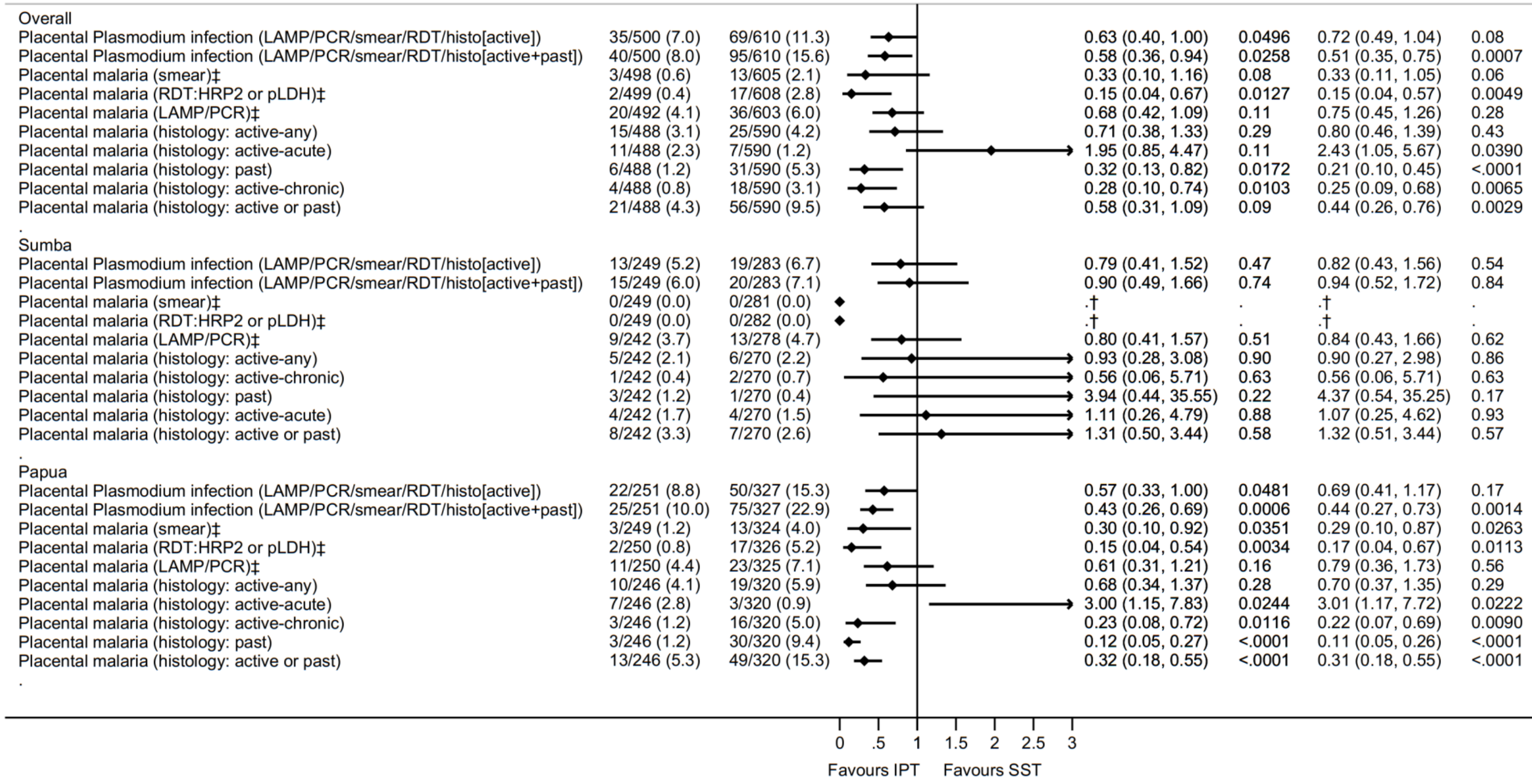

RR=Relative Risk. * Data for represents the n/N (%). Adjusted RR obtained using the same covariates as outlined in Figure 2, under post-hoc adjusted analysis. † RR and p-value could not be computed because of zero events in at least one of the arms. ‡ outcome variable defined post-hoc.

eFigure 12: Placental malaria by treatment group and site in the intention-to-treat population (IST vs SST)

IST vs SST	IST n/N (%)	SST, n/N (%)	Crude RR (95% CI)	P-value	Adjusted RR (95% CI)	P-value
Overall						
Placental Plasmodium infection (LAMP/PCR/smear/RDT/histo[active])	52/694 (7.5)	69/610 (11.3)	0.65 (0.44, 0.95)	0.0278	0.85 (0.66, 1.10)	0.22
Placental Plasmodium infection (LAMP/PCR/smear/RDT/histo[active+past])	95/694 (13.7)	95/610 (15.6)	0.82 (0.54, 1.25)	0.35	0.95 (0.78, 1.16)	0.61
Placental malaria (smear)‡	7/686 (1.0)	13/605 (2.1)	0.47 (0.15, 1.45)	0.19	0.59 (0.25, 1.37)	0.22
Placental malaria (RDT:HRP2 or pLDH)‡	10/692 (1.4)	17/608 (2.8)	0.50 (0.20, 1.29)	0.15	0.55 (0.25, 1.24)	0.15
Placental malaria (LAMP/PCR)‡	30/678 (4.4)	36/603 (6.0)	0.75 (0.47, 1.19)	0.22	0.96 (0.59, 1.55)	0.86
Placental malaria (histology: active-any)	19/672 (2.8)	25/590 (4.2)	0.68 (0.41, 1.10)	0.12	0.74 (0.49, 1.11)	0.15
Placental malaria (histology: active-chronic)	14/672 (2.1)	18/590 (3.1)	0.67 (0.32, 1.44)	0.31	0.56 (0.29, 1.06)	0.07
Placental malaria (histology: past)	45/672 (6.7)	31/590 (5.3)	1.28 (0.60, 2.72)	0.52	1.04 (0.77, 1.40)	0.80
Placental malaria (histology: active-acute)	5/672 (0.7)	7/590 (1.2)	0.59 (0.20, 1.72)	0.33	0.90 (0.27, 2.98)	0.87
Placental malaria (histology: active or past)	64/672 (9.5)	56/590 (9.5)	0.96 (0.55, 1.67)	0.88	0.96 (0.71, 1.30)	0.79
.						
Sumba						
Placental Plasmodium infection (LAMP/PCR/smear/RDT/histo[active])	13/279 (4.7)	19/283 (6.7)	0.70 (0.36, 1.36)	0.30	0.74 (0.34, 1.59)	0.44
Placental Plasmodium infection (LAMP/PCR/smear/RDT/histo[active+past])	16/279 (5.7)	20/283 (7.1)	0.87 (0.50, 1.51)	0.61	0.85 (0.46, 1.55)	0.59
Placental malaria (smear)‡	1/276 (0.4)	0/281 (0.0)	.†	.	.†	.
Placental malaria (RDT:HRP2 or pLDH)‡	1/278 (0.4)	0/282 (0.0)	.†	.	.†	.
Placental malaria (LAMP/PCR)‡	9/271 (3.3)	13/278 (4.7)	0.71 (0.28, 1.84)	0.48	0.68 (0.23, 2.01)	0.49
Placental malaria (histology: active-any)	4/273 (1.5)	6/270 (2.2)	0.66 (0.22, 1.98)	0.46	0.83 (0.22, 3.14)	0.79
Placental malaria (histology: active-acute)	3/273 (1.1)	4/270 (1.5)	0.75 (0.20, 2.79)	0.66	1.44 (0.33, 6.25)	0.63
Placental malaria (histology: past)	3/273 (1.1)	1/270 (0.4)	3.72 (0.43, 32.09)	0.23	2.37 (0.24, 23.56)	0.46
Placental malaria (histology: active-chronic)	1/273 (0.4)	2/270 (0.7)	0.48 (0.04, 5.30)	0.55	0.48 (0.04, 5.30)	0.55
Placental malaria (histology: active or past)	7/273 (2.6)	7/270 (2.6)	1.05 (0.43, 2.57)	0.91	1.04 (0.39, 2.76)	0.94
.						
Papua						
Placental Plasmodium infection (LAMP/PCR/smear/RDT/histo[active])	39/415 (9.4)	50/327 (15.3)	0.62 (0.44, 0.86)	0.0049	0.83 (0.59, 1.15)	0.26
Placental Plasmodium infection (LAMP/PCR/smear/RDT/histo[active+past])	79/415 (19.0)	75/327 (22.9)	0.84 (0.69, 1.02)	0.08	0.90 (0.70, 1.14)	0.37
Placental malaria (smear)‡	6/410 (1.5)	13/324 (4.0)	0.36 (0.13, 1.03)	0.06	0.36 (0.13, 1.00)	0.05
Placental malaria (RDT:HRP2 or pLDH)‡	9/414 (2.2)	17/326 (5.2)	0.43 (0.21, 0.90)	0.0240	0.54 (0.26, 1.13)	0.10
Placental malaria (LAMP/PCR)‡	21/407 (5.2)	23/325 (7.1)	0.74 (0.46, 1.19)	0.21	1.22 (0.64, 2.35)	0.55
Placental malaria (histology: active-any)	15/399 (3.8)	19/320 (5.9)	0.64 (0.40, 1.03)	0.07	0.69 (0.39, 1.21)	0.20
Placental malaria (histology: active-chronic)	13/399 (3.3)	16/320 (5.0)	0.68 (0.39, 1.20)	0.18	0.64 (0.33, 1.24)	0.18
Placental malaria (histology: past)	42/399 (10.5)	30/320 (9.4)	1.09 (0.77, 1.54)	0.63	0.96 (0.73, 1.27)	0.79
Placental malaria (histology: active-acute)	2/399 (0.5)	3/320 (0.9)	0.45 (0.09, 2.24)	0.33	0.45 (0.09, 2.24)	0.33
Placental malaria (histology: active or past)	57/399 (14.3)	49/320 (15.3)	0.93 (0.76, 1.13)	0.46	0.89 (0.65, 1.21)	0.44
.						

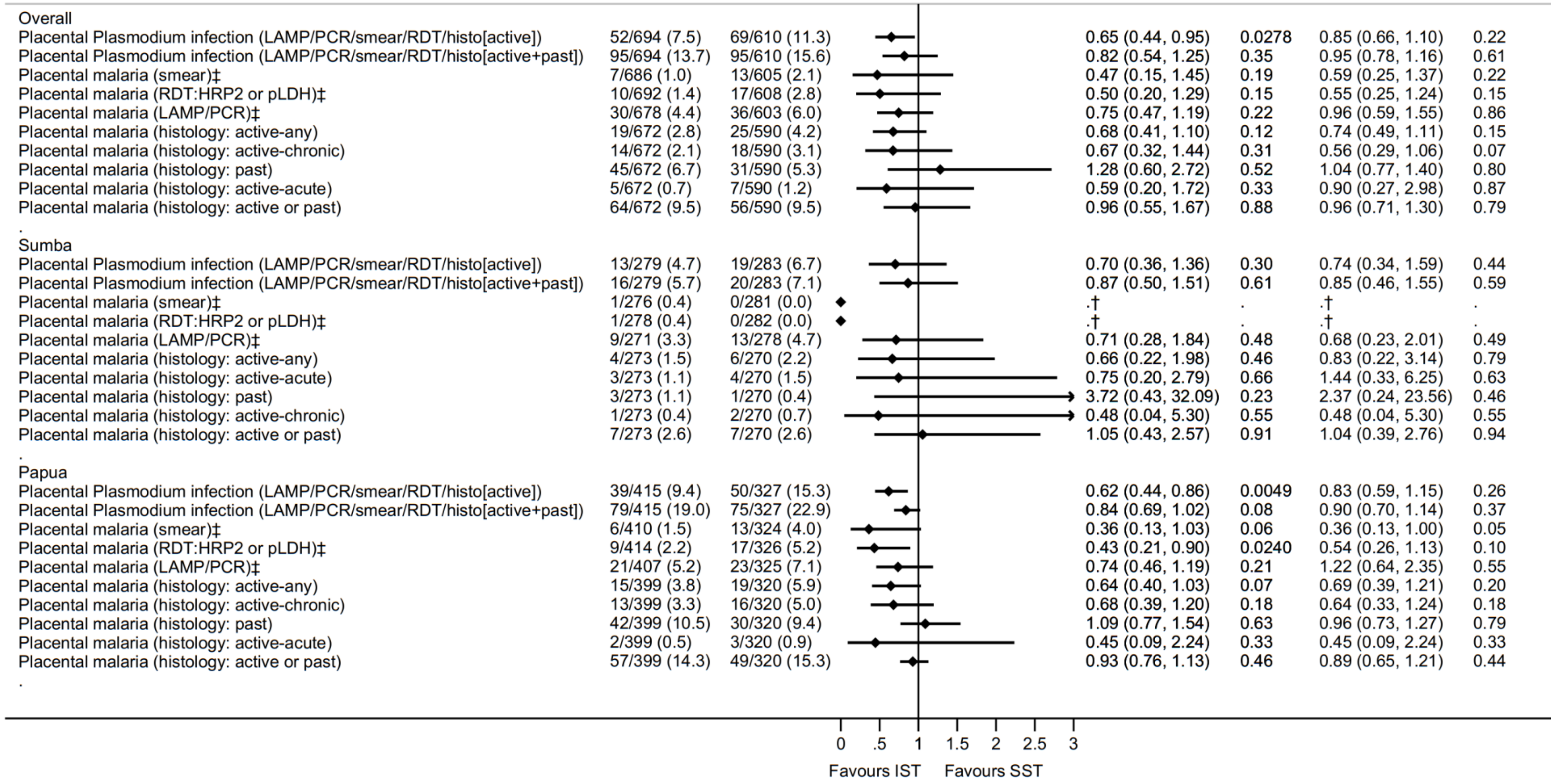

RR=Relative Risk. * Data for represents the n/N (%). Adjusted RR obtained using the same covariates as outlined in Figure 2, under post-hoc adjusted analysis. † RR and p-value could not be computed because of zero events in at least one of the arms. ‡ outcome variable defined post-hoc.

eFigure 13: Placental malaria by treatment group and site in the intention-to-treat population (IPT vs IST)

IPT vs IST	IPT n/N (%)	IST, n/N (%)	Crude RR (95% CI)	P-value	Adjusted RR (95% CI)	P-value
Overall						
Placental Plasmodium infection (LAMP/PCR/smear/RDT/histo[active])	35/500 (7.0)	52/694 (7.5)	0.97 (0.61, 1.55)	0.90	0.85 (0.61, 1.17)	0.31
Placental Plasmodium infection (LAMP/PCR/smear/RDT/histo[active+past])	40/500 (8.0)	95/694 (13.7)	0.71 (0.44, 1.16)	0.17	0.54 (0.39, 0.75)	0.0002
Placental malaria (smear)‡	3/498 (0.6)	7/686 (1.0)	0.71 (0.18, 2.81)	0.62	0.57 (0.17, 1.90)	0.36
Placental malaria (RDT:HRP2 or pLDH)‡	2/499 (0.4)	10/692 (1.4)	0.30 (0.07, 1.30)	0.11	0.28 (0.07, 1.09)	0.07
Placental malaria (LAMP/PCR)‡	20/492 (4.1)	30/678 (4.4)	0.91 (0.53, 1.55)	0.72	0.79 (0.51, 1.23)	0.29
Placental malaria (histology: active-any)	15/488 (3.1)	19/672 (2.8)	1.06 (0.54, 2.07)	0.87	1.08 (0.61, 1.91)	0.79
Placental malaria (histology: active-chronic)	4/488 (0.8)	14/672 (2.1)	0.41 (0.13, 1.25)	0.12	0.44 (0.15, 1.33)	0.15
Placental malaria (histology: active-acute)	11/488 (2.3)	5/672 (0.7)	3.32 (1.17, 9.43)	0.0246	2.70 (0.92, 7.89)	0.07
Placental malaria (histology: past)	6/488 (1.2)	45/672 (6.7)	0.25 (0.11, 0.58)	0.0012	0.20 (0.10, 0.41)	<.0001
Placental malaria (histology: active or past)	21/488 (4.3)	64/672 (9.5)	0.60 (0.32, 1.13)	0.11	0.46 (0.28, 0.75)	0.0020
.						
Sumba						
Placental Plasmodium infection (LAMP/PCR/smear/RDT/histo[active])	13/249 (5.2)	13/279 (4.7)	1.12 (0.55, 2.28)	0.75	1.11 (0.53, 2.32)	0.79
Placental Plasmodium infection (LAMP/PCR/smear/RDT/histo[active+past])	15/249 (6.0)	16/279 (5.7)	1.04 (0.59, 1.83)	0.89	1.11 (0.61, 2.02)	0.72
Placental malaria (smear)‡	0/249 (0.0)	1/276 (0.4)	.†	.	.†	.
Placental malaria (RDT:HRP2 or pLDH)‡	0/249 (0.0)	1/278 (0.4)	.†	.	.†	.
Placental malaria (LAMP/PCR)‡	9/242 (3.7)	9/271 (3.3)	1.12 (0.47, 2.66)	0.80	1.23 (0.48, 3.15)	0.66
Placental malaria (histology: active-any)	5/242 (2.1)	4/273 (1.5)	1.40 (0.38, 5.10)	0.61	1.08 (0.28, 4.17)	0.91
Placental malaria (histology: past)	3/242 (1.2)	3/273 (1.1)	1.06 (0.29, 3.86)	0.93	1.84 (0.60, 5.66)	0.29
Placental malaria (histology: active-chronic)	1/242 (0.4)	1/273 (0.4)	1.16 (0.07, 18.15)	0.92	1.16 (0.07, 18.15)	0.92
Placental malaria (histology: active-acute)	4/242 (1.7)	3/273 (1.1)	1.50 (0.33, 6.78)	0.60	0.74 (0.19, 2.95)	0.67
Placental malaria (histology: active or past)	8/242 (3.3)	7/273 (2.6)	1.25 (0.49, 3.17)	0.65	1.27 (0.50, 3.26)	0.61
.						
Papua						
Placental Plasmodium infection (LAMP/PCR/smear/RDT/histo[active])	22/251 (8.8)	39/415 (9.4)	0.93 (0.54, 1.61)	0.79	0.84 (0.52, 1.34)	0.45
Placental Plasmodium infection (LAMP/PCR/smear/RDT/histo[active+past])	25/251 (10.0)	79/415 (19.0)	0.51 (0.31, 0.83)	0.0068	0.49 (0.32, 0.76)	0.0013
Placental malaria (smear)‡	3/249 (1.2)	6/410 (1.5)	0.84 (0.21, 3.43)	0.81	0.81 (0.20, 3.21)	0.76
Placental malaria (RDT:HRP2 or pLDH)‡	2/250 (0.8)	9/414 (2.2)	0.36 (0.10, 1.25)	0.11	0.32 (0.08, 1.23)	0.10
Placental malaria (LAMP/PCR)‡	11/250 (4.4)	21/407 (5.2)	0.83 (0.40, 1.74)	0.63	0.65 (0.39, 1.08)	0.10
Placental malaria (histology: active-any)	10/246 (4.1)	15/399 (3.8)	1.05 (0.50, 2.23)	0.89	1.02 (0.46, 2.29)	0.96
Placental malaria (histology: active-acute)	7/246 (2.8)	2/399 (0.5)	6.74 (1.45, 31.29)	0.0149	6.74 (1.46, 31.12)	0.0146
Placental malaria (histology: past)	3/246 (1.2)	42/399 (10.5)	0.11 (0.05, 0.23)	<.0001	0.12 (0.06, 0.26)	<.0001
Placental malaria (histology: active-chronic)	3/246 (1.2)	13/399 (3.3)	0.34 (0.10, 1.15)	0.08	0.35 (0.10, 1.20)	0.09
Placental malaria (histology: active or past)	13/246 (5.3)	57/399 (14.3)	0.34 (0.20, 0.58)	<.0001	0.35 (0.21, 0.60)	<.0001
.						

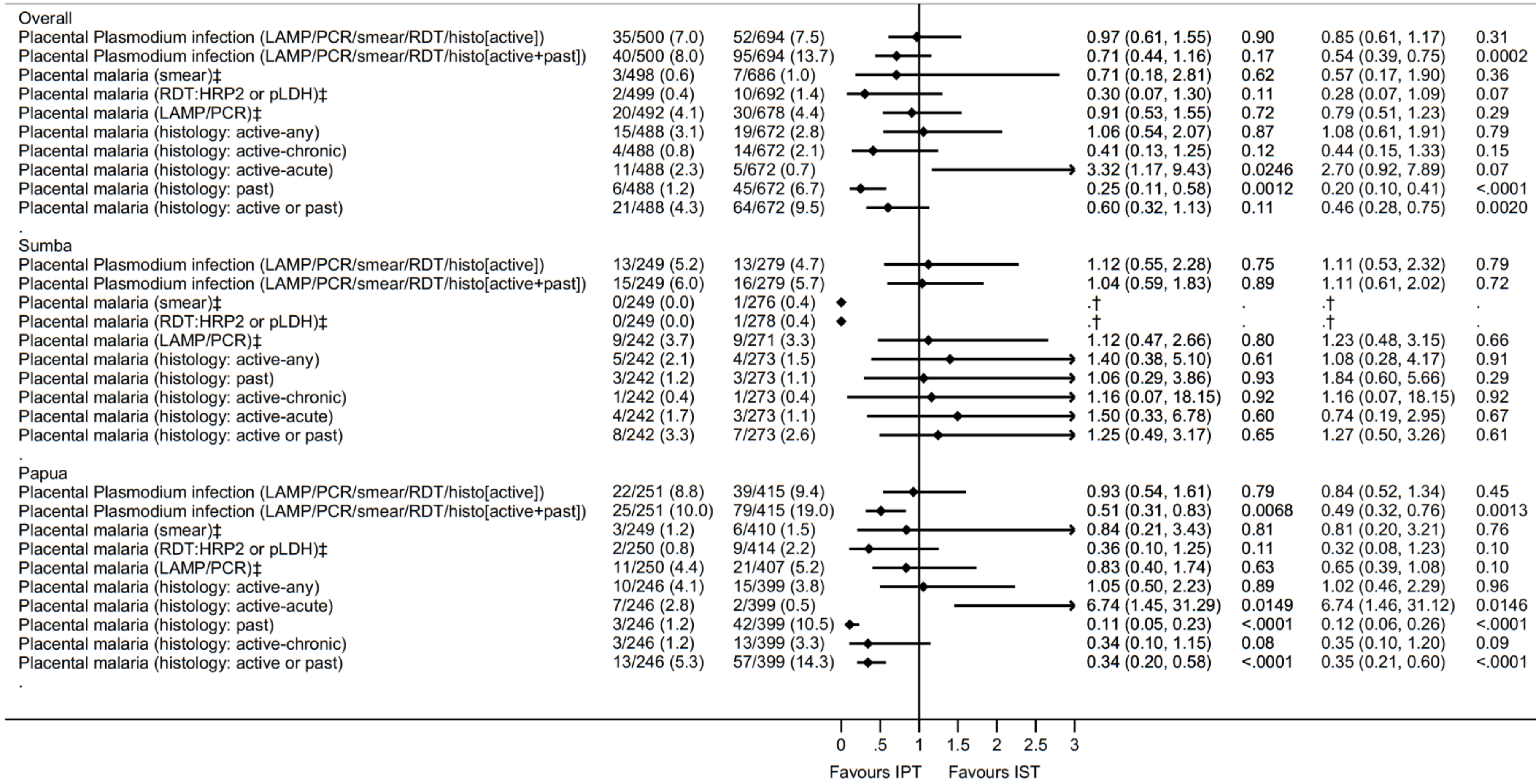

RR=Relative Risk. * Data for represents the n/N (%). Adjusted RR obtained using the same covariates as outlined in Figure 2, under post-hoc adjusted analysis. † RR and p-value could not be computed because of zero events in at least one of the arms. ‡ outcome variable defined post-hoc.

eFigure 14: Malaria and non-malaria sick visits during pregnancy by treatment group in the intention-to-treat population (IPT vs SST)

IPT vs SST	IPT n/N (%) or N, events/PY (IR)	SST, n/N (%) or N, events/PY (IR)	Crude RR or IRR (95% CI)	P-value	Adjusted RR or IRR (95% CI)	P-value
1. Overall						
Maternal Plasmodium infection during pregnancy (LAMP/PCR/smear/RDT) (cumulative risk)	53/595 (8.9)	118/680 (17.4)	0.60 (0.39, 0.92)	0.0177	0.60 (0.40, 0.89)	0.0107
Maternal Plasmodium infection during pregnancy (LAMP/PCR/smear/RDT) (incidence/100 py)*	595, 56/156.7 (35.7)	680, 146/187.1 (78.0)	0.55 (0.35, 0.85)	0.0077	0.57 (0.38, 0.85)	0.0055
Maternal Plasmodium infection during pregnancy (fever-RDT) (cumulative risk)	2/595 (0.3)	9/680 (1.3)	0.33 (0.07, 1.64)	0.18	0.33 (0.07, 1.64)	0.18
Maternal Plasmodium infection during pregnancy (fever-RDT) (incidence/100 py)*	595, 2/156.7 (1.3)	680, 9/187.1 (4.8)	0.29 (0.06, 1.45)	0.13	0.43 (0.09, 1.95)	0.27
Maternal Plasmodium infection during pregnancy (smear) (cumulative risk)	4/595 (0.7)	24/680 (3.5)	0.22 (0.05, 0.99)	0.0478	0.26 (0.06, 1.10)	0.07
Maternal Plasmodium infection during pregnancy (smear) (incidence/100 py)*	595, 4/156.7 (2.6)	680, 47/187.1 (25.1)	0.11 (0.03, 0.49)	0.0035	0.13 (0.04, 0.41)	0.0006
Maternal Plasmodium infection during pregnancy (LAMP/PCR) (cumulative risk)	49/594 (8.2)	106/679 (15.6)	0.60 (0.39, 0.94)	0.0249	0.64 (0.43, 0.96)	0.0313
Maternal Plasmodium infection during pregnancy (LAMP/PCR) (incidence/100 py)*	594, 52/156.7 (33.2)	679, 122/187.1 (65.2)	0.60 (0.38, 0.95)	0.0281	0.62 (0.41, 0.94)	0.0229
Maternal patent Plasmodium infection during pregnancy (cumulative risk)	0/594 (0.0)	23/679 (3.4)	.†	.	.†	.
Maternal patent Plasmodium infection during pregnancy (incidence/100 py)*	594, 0/156.7 (0.0)	679, 25/187.1 (13.4)	.†	.	.†	.
Maternal sub-patent Plasmodium infection during pregnancy (cumulative risk)	49/594 (8.2)	86/679 (12.7)	0.70 (0.45, 1.09)	0.11	0.74 (0.50, 1.10)	0.14
Maternal sub-patent Plasmodium infection during pregnancy (incidence/100 py)*	594, 52/156.7 (33.2)	679, 95/187.1 (50.8)	0.71 (0.45, 1.13)	0.14	0.74 (0.50, 1.11)	0.15
Maternal clinical malaria during pregnancy (cumulative risk)	2/595 (0.3)	13/680 (1.9)	0.22 (0.05, 0.98)	0.0476	0.21 (0.05, 0.82)	0.0252
Maternal clinical malaria during pregnancy (incidence/100py)*	595, 2/156.7 (1.3)	680, 13/187.1 (6.9)	0.23 (0.05, 1.04)	0.06	0.25 (0.06, 0.98)	0.0466
Maternal non-malaria sick visits during pregnancy (cumulative risk)	45/595 (7.6)	53/680 (7.8)	0.99 (0.62, 1.58)	0.97	1.02 (0.63, 1.65)	0.94
Maternal non-malaria sick visits during pregnancy (incidence/100py)*	595, 49/156.7 (31.3)	680, 61/187.1 (32.6)	0.97 (0.61, 1.54)	0.90	0.97 (0.61, 1.54)	0.89
Maternal all-cause sick visits during pregnancy (cumulative risk)	47/595 (7.9)	64/680 (9.4)	0.89 (0.56, 1.39)	0.60	0.91 (0.57, 1.45)	0.69
Maternal all-cause sick visits during pregnancy (incidence/100py)*	595, 51/156.7 (32.5)	680, 74/187.1 (39.5)	0.87 (0.56, 1.36)	0.54	0.89 (0.56, 1.40)	0.60
2. Sumba						
Maternal Plasmodium infection during pregnancy (LAMP/PCR/smear/RDT) (cumulative risk)	36/276 (13.0)	40/324 (12.3)	1.05 (0.62, 1.78)	0.85	1.08 (0.70, 1.68)	0.72
Maternal Plasmodium infection during pregnancy (LAMP/PCR/smear/RDT) (incidence/100 py)*	276, 39/72.0 (54.2)	324, 45/84.8 (53.1)	1.02 (0.59, 1.74)	0.95	1.04 (0.66, 1.66)	0.86
Maternal Plasmodium infection during pregnancy (fever-RDT) (cumulative risk)	0/276 (0.0)	0/324 (0.0)	.†	.	.†	.
Maternal Plasmodium infection during pregnancy (fever-RDT) (incidence/100 py)*	276, 0/72.0 (0.0)	324, 0/84.8 (0.0)	.†	.	.†	.
Maternal Plasmodium infection during pregnancy (smear) (cumulative risk)	0/276 (0.0)	2/324 (0.6)	.†	.	.†	.
Maternal Plasmodium infection during pregnancy (smear) (incidence/100 py)*	276, 0/72.0 (0.0)	324, 2/84.8 (2.4)	.†	.	.†	.
Maternal Plasmodium infection during pregnancy (LAMP/PCR) (cumulative risk)	36/275 (13.1)	40/323 (12.4)	1.05 (0.62, 1.79)	0.84	1.09 (0.71, 1.68)	0.69
Maternal Plasmodium infection during pregnancy (LAMP/PCR) (incidence/100 py)*	275, 39/72.0 (54.2)	323, 45/84.8 (53.1)	1.02 (0.60, 1.74)	0.95	1.05 (0.66, 1.66)	0.84
Maternal patent Plasmodium infection during pregnancy (cumulative risk)	0/275 (0.0)	2/323 (0.6)	.†	.	.†	.
Maternal patent Plasmodium infection during pregnancy (incidence/100 py)*	275, 0/72.0 (0.0)	323, 2/84.8 (2.4)	.†	.	.†	.
Maternal sub-patent Plasmodium infection during pregnancy (cumulative risk)	36/275 (13.1)	38/323 (11.8)	1.11 (0.65, 1.89)	0.70	1.13 (0.72, 1.77)	0.60
Maternal sub-patent Plasmodium infection during pregnancy (incidence/100 py)*	275, 39/72.0 (54.2)	323, 42/84.8 (49.5)	1.09 (0.63, 1.89)	0.75	1.12 (0.70, 1.79)	0.63
Maternal clinical malaria during pregnancy (cumulative risk)	0/276 (0.0)	0/324 (0.0)	.†	.	.†	.
Maternal clinical malaria during pregnancy (incidence/100py)*	276, 0/72.0 (0.0)	324, 0/84.8 (0.0)	.†	.	.†	.
Maternal non-malaria sick visits during pregnancy (cumulative risk)	26/276 (9.4)	24/324 (7.4)	1.26 (0.69, 2.33)	0.45	1.21 (0.65, 2.25)	0.56
Maternal non-malaria sick visits during pregnancy (incidence/100py)*	276, 28/72.0 (38.9)	324, 27/84.8 (31.9)	1.25 (0.72, 2.17)	0.42	1.24 (0.70, 2.20)	0.46
Maternal all-cause sick visits during pregnancy (cumulative risk)	26/276 (9.4)	24/324 (7.4)	1.26 (0.69, 2.33)	0.45	1.20 (0.65, 2.22)	0.56
Maternal all-cause sick visits during pregnancy (incidence/100py)*	276, 28/72.0 (38.9)	324, 27/84.8 (31.9)	1.25 (0.72, 2.16)	0.43	1.23 (0.70, 2.17)	0.47
3. Papua						
Maternal Plasmodium infection during pregnancy (LAMP/PCR/smear/RDT) (cumulative risk)	17/319 (5.3)	78/356 (21.9)	0.23 (0.14, 0.39)	<.0001	0.23 (0.14, 0.39)	<.0001
Maternal Plasmodium infection during pregnancy (LAMP/PCR/smear/RDT) (incidence/100 py)*	319, 17/84.7 (20.1)	356, 101/102.4 (98.7)	0.20 (0.12, 0.34)	<.0001	0.23 (0.15, 0.37)	<.0001
Maternal Plasmodium infection during pregnancy (fever-RDT) (cumulative risk)	2/319 (0.6)	9/356 (2.5)	0.22 (0.05, 1.00)	0.05	0.23 (0.05, 1.01)	0.05
Maternal Plasmodium infection during pregnancy (fever-RDT) (incidence/100 py)*	319, 2/84.7 (2.4)	356, 9/102.4 (8.8)	0.23 (0.05, 1.04)	0.06	0.26 (0.06, 1.19)	0.08
Maternal Plasmodium infection during pregnancy (smear) (cumulative risk)	4/319 (1.3)	22/356 (6.2)	0.22 (0.05, 0.97)	0.0448	0.22 (0.05, 0.97)	0.0450
Maternal Plasmodium infection during pregnancy (smear) (incidence/100 py)*	319, 4/84.7 (4.7)	356, 45/102.4 (44.0)	0.12 (0.03, 0.47)	0.0027	0.13 (0.04, 0.46)	0.0013
Maternal Plasmodium infection during pregnancy (LAMP/PCR) (cumulative risk)	13/319 (4.1)	66/356 (18.5)	0.20 (0.12, 0.33)	<.0001	0.20 (0.12, 0.33)	<.0001
Maternal Plasmodium infection during pregnancy (LAMP/PCR) (incidence/100 py)*	319, 13/84.7 (15.3)	356, 77/102.4 (75.2)	0.20 (0.12, 0.34)	<.0001	0.22 (0.12, 0.40)	<.0001
Maternal patent Plasmodium infection during pregnancy (cumulative risk)	0/319 (0.0)	21/356 (5.9)	.†	.	.†	.
Maternal patent Plasmodium infection during pregnancy (incidence/100 py)*	319, 0/84.7 (0.0)	356, 23/102.4 (22.5)	.†	.	.†	.
Maternal sub-patent Plasmodium infection during pregnancy (cumulative risk)	13/319 (4.1)	48/356 (13.5)	0.28 (0.17, 0.46)	<.0001	0.30 (0.18, 0.51)	<.0001
Maternal sub-patent Plasmodium infection during pregnancy (incidence/100 py)*	319, 13/84.7 (15.3)	356, 53/102.4 (51.8)	0.28 (0.17, 0.49)	<.0001	0.30 (0.17, 0.54)	<.0001
Maternal clinical malaria during pregnancy (cumulative risk)	2/319 (0.6)	13/356 (3.7)	0.16 (0.04, 0.67)	0.0120	0.16 (0.04, 0.67)	0.0120
Maternal clinical malaria during pregnancy (incidence/100py)*	319, 2/84.7 (2.4)	356, 13/102.4 (12.7)	0.18 (0.04, 0.76)	0.0198	0.16 (0.04, 0.67)	0.0121
Maternal non-malaria sick visits during pregnancy (cumulative risk)	19/319 (6.0)	29/356 (8.1)	0.69 (0.29, 1.64)	0.40	0.58 (0.26, 1.31)	0.19
Maternal non-malaria sick visits during pregnancy (incidence/100py)*	319, 21/84.7 (24.8)	356, 34/102.4 (33.2)	0.64 (0.25, 1.65)	0.36	0.53 (0.22, 1.29)	0.16
Maternal all-cause sick visits during pregnancy (cumulative risk)	21/319 (6.6)	40/356 (11.2)	0.57 (0.26, 1.23)	0.15	0.53 (0.26, 1.06)	0.07
Maternal all-cause sick visits during pregnancy (incidence/100py)*	319, 23/84.7 (27.1)	356, 47/102.4 (45.9)	0.53 (0.23, 1.22)	0.14	0.46 (0.20, 1.02)	0.06

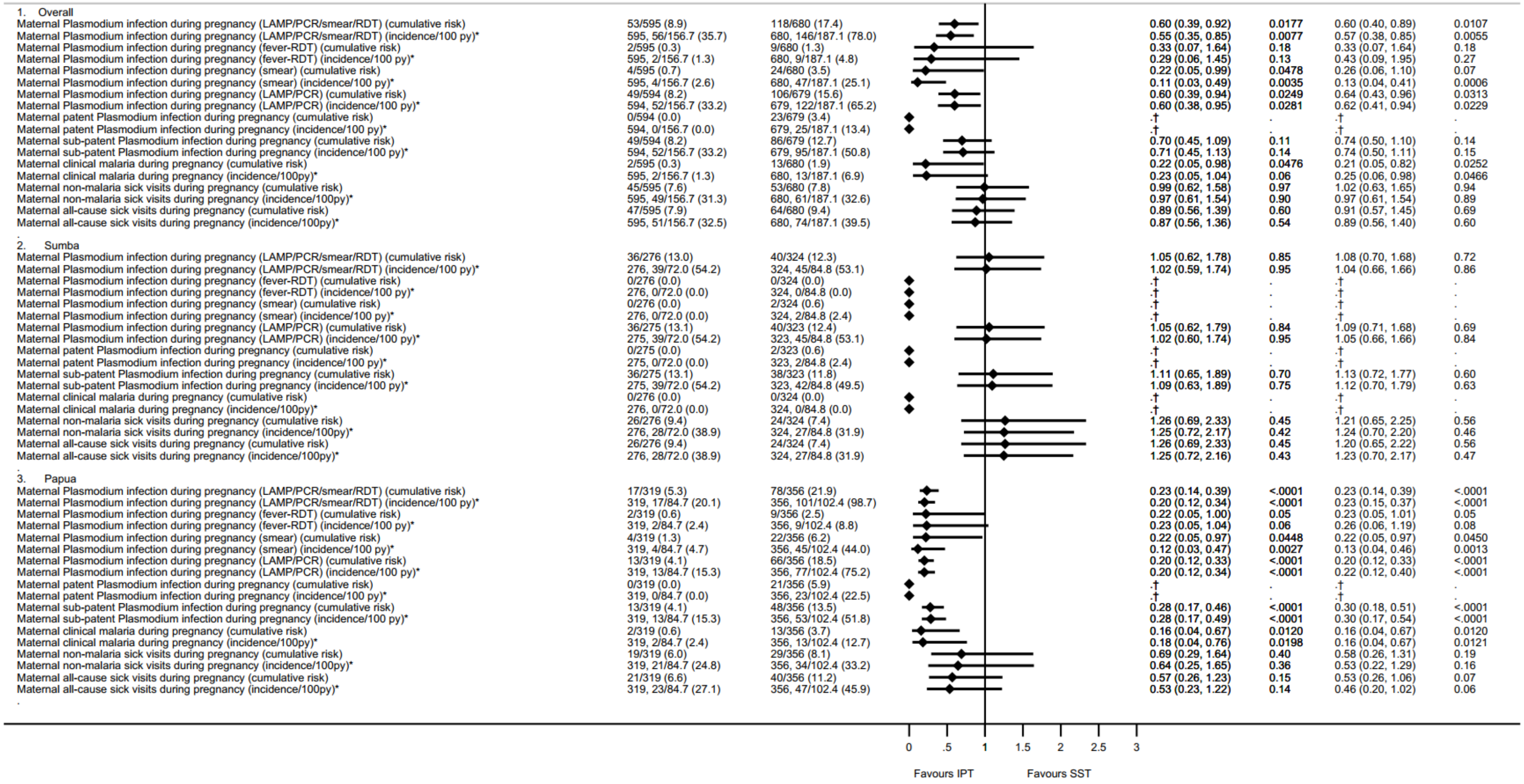

RR=Relative Risk, IRR=Incidence rate ratio, Adjusted RR or IRR obtained using the same covariates as in Figure 2 of the main text, RDT=Rapid diagnostic test for malaria, fever-RDT=RDT taken among women with document fever or a history of fever in the last 48 hours in all 3 arms. * Data represents n/N (%) except for the data for the incidence per 100 person-years which represent the number of women with an event, the number of events, the follow-up person time and in brackets, the incidence rate per 100 person-years. † RR/IRR and p-value could not be computed because of zero events in at least one of the arms.

eFigure 15: Malaria and non-malaria sick visits during pregnancy by treatment group in the intention-to-treat population (IST vs SST)

IST vs SST	IST n/N (%) or N, events/PY (IR)	SST, n/N (%) or N, events/PY (IR)	Crude RR or IRR (95% CI)	P-value	Adjusted RR (95% CI)	P-value
1. Overall						
Maternal Plasmodium infection during pregnancy (LAMP/PCR/smear/RDT) (cumulative risk)	120/786 (15.3)	118/680 (17.4)	0.86 (0.59, 1.27)	0.45	1.13 (0.74, 1.75)	0.57
Maternal Plasmodium infection during pregnancy (LAMP/PCR/smear/RDT) (incidence/100 py)*	786, 153/222.2 (68.8)	680, 146/187.1 (78.0)	0.87 (0.59, 1.29)	0.49	1.24 (0.83, 1.83)	0.29
Maternal Plasmodium infection during pregnancy (fever-RDT) (cumulative risk)	3/786 (0.4)	9/680 (1.3)	0.39 (0.09, 1.68)	0.21	0.39 (0.09, 1.68)	0.21
Maternal Plasmodium infection during pregnancy (fever-RDT) (incidence/100 py)*	786, 4/222.2 (1.8)	680, 9/187.1 (4.8)	0.43 (0.10, 1.94)	0.27	0.90 (0.08, 10.13)	0.93
Maternal Plasmodium infection during pregnancy (smear) (cumulative risk)	22/786 (2.8)	24/680 (3.5)	0.83 (0.34, 2.03)	0.69	1.49 (0.57, 3.92)	0.42
Maternal Plasmodium infection during pregnancy (smear) (incidence/100 py)*	786, 41/222.2 (18.4)	680, 47/187.1 (25.1)	0.69 (0.31, 1.52)	0.35	1.38 (0.65, 2.89)	0.40
Maternal Plasmodium infection during pregnancy (LAMP/PCR) (cumulative risk)	98/786 (12.5)	106/679 (15.6)	0.81 (0.54, 1.20)	0.29	1.05 (0.67, 1.64)	0.84
Maternal Plasmodium infection during pregnancy (LAMP/PCR) (incidence/100 py)*	786, 119/222.2 (53.5)	679, 122/187.1 (65.2)	0.84 (0.56, 1.27)	0.41	1.16 (0.73, 1.83)	0.54
Maternal patent Plasmodium infection during pregnancy (cumulative risk)	17/786 (2.2)	23/679 (3.4)	0.67 (0.26, 1.74)	0.41	1.24 (0.40, 3.84)	0.72
Maternal patent Plasmodium infection during pregnancy (incidence/100 py)*	786, 19/222.2 (8.5)	679, 25/187.1 (13.4)	0.71 (0.27, 1.91)	0.50	1.59 (0.38, 6.56)	0.52
Maternal sub-patent Plasmodium infection during pregnancy (cumulative risk)	87/786 (11.1)	86/679 (12.7)	0.86 (0.58, 1.27)	0.46	1.12 (0.74, 1.69)	0.61
Maternal sub-patent Plasmodium infection during pregnancy (incidence/100 py)*	786, 100/222.2 (45.0)	679, 95/187.1 (50.8)	0.88 (0.59, 1.30)	0.51	1.13 (0.73, 1.75)	0.58
Maternal clinical malaria during pregnancy (cumulative risk)	8/786 (1.0)	13/680 (1.9)	0.56 (0.20, 1.58)	0.27	0.82 (0.29, 2.30)	0.70
Maternal clinical malaria during pregnancy (incidence/100py)*	786, 9/222.2 (4.0)	680, 13/187.1 (6.9)	0.66 (0.23, 1.84)	0.43	1.11 (0.28, 4.37)	0.88
Maternal non-malaria sick visits during pregnancy (cumulative risk)	66/786 (8.4)	53/680 (7.8)	1.14 (0.73, 1.79)	0.56	1.29 (0.79, 2.12)	0.31
Maternal non-malaria sick visits during pregnancy (incidence/100py)*	786, 74/222.2 (33.3)	680, 61/187.1 (32.6)	1.09 (0.71, 1.69)	0.70	1.30 (0.81, 2.11)	0.28
Maternal all-cause sick visits during pregnancy (cumulative risk)	73/786 (9.3)	64/680 (9.4)	1.06 (0.69, 1.62)	0.79	1.22 (0.75, 1.96)	0.43
Maternal all-cause sick visits during pregnancy (incidence/100py)*	786, 83/222.2 (37.3)	680, 74/187.1 (39.5)	1.06 (0.70, 1.60)	0.80	1.31 (0.82, 2.10)	0.26
2. Sumba						
Maternal Plasmodium infection during pregnancy (LAMP/PCR/smear/RDT) (cumulative risk)	46/338 (13.6)	40/324 (12.3)	1.11 (0.65, 1.90)	0.71	1.31 (0.80, 2.15)	0.28
Maternal Plasmodium infection during pregnancy (LAMP/PCR/smear/RDT) (incidence/100 py)*	338, 54/87.8 (61.5)	324, 45/84.8 (53.1)	1.19 (0.69, 2.06)	0.54	1.42 (0.82, 2.44)	0.21
Maternal Plasmodium infection during pregnancy (fever-RDT) (cumulative risk)	2/338 (0.6)	0/324 (0.0)	.†	.	.†	.
Maternal Plasmodium infection during pregnancy (fever-RDT) (incidence/100 py)*	338, 3/87.8 (3.4)	324, 0/84.8 (0.0)	.†	.	.†	.
Maternal Plasmodium infection during pregnancy (smear) (cumulative risk)	5/338 (1.5)	2/324 (0.6)	2.62 (0.41, 16.67)	0.31	2.62 (0.41, 16.67)	0.31
Maternal Plasmodium infection during pregnancy (smear) (incidence/100 py)*	338, 5/87.8 (5.7)	324, 2/84.8 (2.4)	2.40 (0.39, 14.83)	0.35	4.62 (0.60, 35.76)	0.14
Maternal Plasmodium infection during pregnancy (LAMP/PCR) (cumulative risk)	46/338 (13.6)	40/323 (12.4)	1.11 (0.65, 1.89)	0.71	1.31 (0.80, 2.14)	0.28
Maternal Plasmodium infection during pregnancy (LAMP/PCR) (incidence/100 py)*	338, 54/87.8 (61.5)	323, 45/84.8 (53.1)	1.19 (0.68, 2.06)	0.54	1.42 (0.83, 2.44)	0.21
Maternal patent Plasmodium infection during pregnancy (cumulative risk)	5/338 (1.5)	2/323 (0.6)	2.61 (0.41, 16.65)	0.31	5.60 (0.53, 59.08)	0.15
Maternal patent Plasmodium infection during pregnancy (incidence/100 py)*	338, 6/87.8 (6.8)	323, 2/84.8 (2.4)	2.91 (0.44, 19.25)	0.27	6.82 (0.73, 63.79)	0.09
Maternal sub-patent Plasmodium infection during pregnancy (cumulative risk)	44/338 (13.0)	38/323 (11.8)	1.10 (0.65, 1.89)	0.72	1.27 (0.78, 2.06)	0.33
Maternal sub-patent Plasmodium infection during pregnancy (incidence/100 py)*	338, 48/87.8 (54.6)	323, 42/84.8 (49.5)	1.12 (0.66, 1.90)	0.68	1.28 (0.76, 2.14)	0.35
Maternal clinical malaria during pregnancy (cumulative risk)	2/338 (0.6)	0/324 (0.0)	.†	.	.†	.
Maternal clinical malaria during pregnancy (incidence/100py)*	338, 3/87.8 (3.4)	324, 0/84.8 (0.0)	.†	.	.†	.
Maternal non-malaria sick visits during pregnancy (cumulative risk)	39/338 (11.5)	24/324 (7.4)	1.50 (0.78, 2.90)	0.23	1.74 (0.92, 3.30)	0.09
Maternal non-malaria sick visits during pregnancy (incidence/100py)*	338, 42/87.8 (47.8)	324, 27/84.8 (31.9)	1.49 (0.80, 2.76)	0.21	1.94 (1.09, 3.43)	0.0240
Maternal all-cause sick visits during pregnancy (cumulative risk)	40/338 (11.8)	24/324 (7.4)	1.55 (0.81, 2.97)	0.19	1.89 (1.01, 3.53)	0.0470
Maternal all-cause sick visits during pregnancy (incidence/100py)*	338, 45/87.8 (51.2)	324, 27/84.8 (31.9)	1.62 (0.88, 2.97)	0.12	2.20 (1.27, 3.83)	0.0051
3. Papua						
Maternal Plasmodium infection during pregnancy (LAMP/PCR/smear/RDT) (cumulative risk)	74/448 (16.5)	78/356 (21.9)	0.67 (0.41, 1.09)	0.10	0.67 (0.41, 1.09)	0.10
Maternal Plasmodium infection during pregnancy (LAMP/PCR/smear/RDT) (incidence/100 py)*	448, 99/134.4 (73.7)	356, 101/102.4 (98.7)	0.65 (0.40, 1.06)	0.08	1.04 (0.64, 1.69)	0.87
Maternal Plasmodium infection during pregnancy (fever-RDT) (cumulative risk)	1/448 (0.2)	9/356 (2.5)	0.10 (0.02, 0.49)	0.0046	0.09 (0.02, 0.46)	0.0037
Maternal Plasmodium infection during pregnancy (fever-RDT) (incidence/100 py)*	448, 1/134.4 (0.7)	356, 9/102.4 (8.8)	0.10 (0.02, 0.43)	0.0021	0.11 (0.02, 0.47)	0.0032
Maternal Plasmodium infection during pregnancy (smear) (cumulative risk)	17/448 (3.8)	22/356 (6.2)	0.57 (0.27, 1.18)	0.13	0.57 (0.27, 1.18)	0.13
Maternal Plasmodium infection during pregnancy (smear) (incidence/100 py)*	448, 36/134.4 (26.8)	356, 45/102.4 (44.0)	0.53 (0.29, 0.95)	0.0336	1.12 (0.61, 2.07)	0.71
Maternal Plasmodium infection during pregnancy (LAMP/PCR) (cumulative risk)	52/448 (11.6)	66/356 (18.5)	0.55 (0.32, 0.96)	0.0343	0.55 (0.32, 0.96)	0.0344
Maternal Plasmodium infection during pregnancy (LAMP/PCR) (incidence/100 py)*	448, 65/134.4 (48.4)	356, 77/102.4 (75.2)	0.55 (0.31, 1.00)	0.0484	0.83 (0.41, 1.68)	0.60
Maternal patent Plasmodium infection during pregnancy (cumulative risk)	12/448 (2.7)	21/356 (5.9)	0.42 (0.19, 0.93)	0.0326	0.42 (0.19, 0.93)	0.0326
Maternal patent Plasmodium infection during pregnancy (incidence/100 py)*	448, 13/134.4 (9.7)	356, 23/102.4 (22.5)	0.38 (0.17, 0.89)	0.0246	0.85 (0.25, 2.89)	0.80
Maternal sub-patent Plasmodium infection during pregnancy (cumulative risk)	43/448 (9.6)	48/356 (13.5)	0.64 (0.36, 1.13)	0.12	0.77 (0.43, 1.38)	0.38
Maternal sub-patent Plasmodium infection during pregnancy (incidence/100 py)*	448, 52/134.4 (38.7)	356, 53/102.4 (51.8)	0.67 (0.37, 1.20)	0.18	0.88 (0.48, 1.59)	0.66
Maternal clinical malaria during pregnancy (cumulative risk)	6/448 (1.3)	13/356 (3.7)	0.39 (0.18, 0.88)	0.0230	0.39 (0.18, 0.88)	0.0230
Maternal clinical malaria during pregnancy (incidence/100py)*	448, 6/134.4 (4.5)	356, 13/102.4 (12.7)	0.35 (0.16, 0.78)	0.0106	0.39 (0.18, 0.88)	0.0230
Maternal non-malaria sick visits during pregnancy (cumulative risk)	27/448 (6.0)	29/356 (8.1)	0.73 (0.49, 1.09)	0.13	0.63 (0.43, 0.92)	0.0160
Maternal non-malaria sick visits during pregnancy (incidence/100py)*	448, 32/134.4 (23.8)	356, 34/102.4 (33.2)	0.71 (0.47, 1.06)	0.09	0.60 (0.40, 0.91)	0.0169
Maternal all-cause sick visits during pregnancy (cumulative risk)	33/448 (7.4)	40/356 (11.2)	0.64 (0.43, 0.97)	0.0328	0.62 (0.43, 0.88)	0.0076
Maternal all-cause sick visits during pregnancy (incidence/100py)*	448, 38/134.4 (28.3)	356, 47/102.4 (45.9)	0.62 (0.42, 0.91)	0.0150	0.70 (0.42, 1.17)	0.17

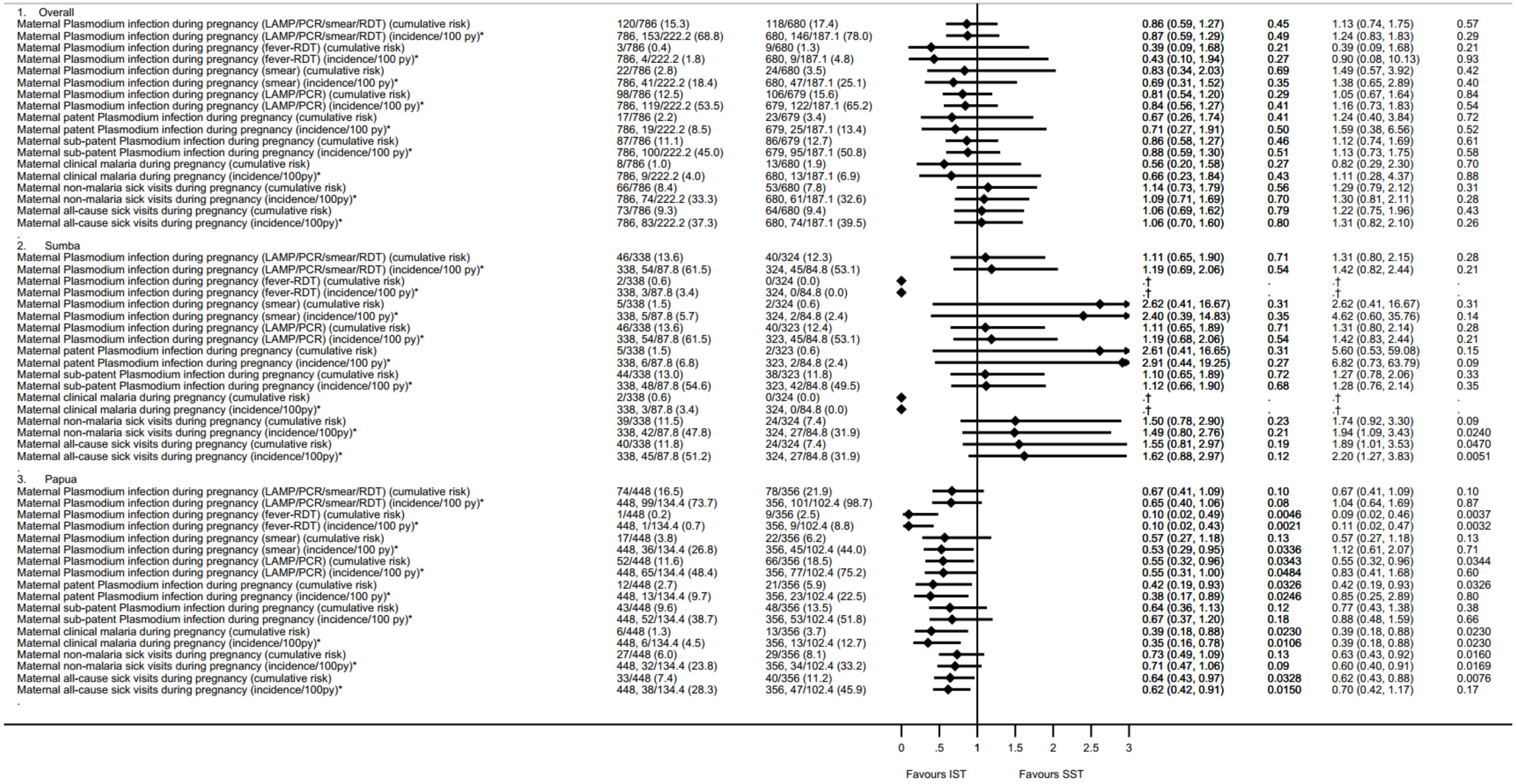

RR=Relative Risk, IRR=Incidence rate ratio, Adjusted RR or IRR obtained using the same covariates as in Figure 2 of the main text, RDT=Rapid diagnostic test for malaria, fever-RDT=RDT taken among women with document fever or a history of fever in the last 48 hours in all 3 arms. * Data represents n/N (%) except for the data for the incidence per 100 person-years which represent the number of women with an event, the number of events, the follow-up person time and in brackets, the incidence rate per 100 person-years. † RR/IRR and p-value could not be computed because of zero events in at least one of the arms.

eFigure 16: Malaria and non-malaria sick visits during pregnancy by treatment group in the intention-to-treat population (IPT vs IST)

IPT vs IST	IPT n/N (%) or N, events/PY (IR)	IST, n/N (%) or N, events/PY (IR)	Crude RR or IRR (95% CI)	P-value	Adjusted RR (95% CI)	P-value
1. Overall						
Maternal Plasmodium infection during pregnancy (LAMP/PCR/smear/RDT) (cumulative risk)	53/595 (8.9)	120/786 (15.3)	0.69 (0.48, 1.00)	0.0497	0.53 (0.34, 0.82)	0.0044
Maternal Plasmodium infection during pregnancy (LAMP/PCR/smear/RDT) (incidence/100 py)*	595, 56/156.7 (35.7)	786, 153/222.2 (68.8)	0.63 (0.43, 0.93)	0.0193	0.46 (0.30, 0.70)	0.0003
Maternal Plasmodium infection during pregnancy (fever-RDT) (cumulative risk)	2/595 (0.3)	3/786 (0.4)	0.84 (0.15, 4.82)	0.84	0.84 (0.15, 4.82)	0.84
Maternal Plasmodium infection during pregnancy (fever-RDT) (incidence/100 py)*	595, 2/156.7 (1.3)	786, 4/222.2 (1.8)	0.68 (0.11, 4.07)	0.67	0.48 (0.05, 5.07)	0.54
Maternal Plasmodium infection during pregnancy (smear) (cumulative risk)	4/595 (0.7)	22/786 (2.8)	0.26 (0.06, 1.21)	0.09	0.18 (0.04, 0.82)	0.0266
Maternal Plasmodium infection during pregnancy (smear) (incidence/100 py)*	595, 4/156.7 (2.6)	786, 41/222.2 (18.4)	0.16 (0.04, 0.69)	0.0140	0.09 (0.03, 0.31)	<.0001
Maternal Plasmodium infection during pregnancy (LAMP/PCR) (cumulative risk)	49/594 (8.2)	98/786 (12.5)	0.75 (0.50, 1.11)	0.14	0.61 (0.39, 0.96)	0.0304
Maternal Plasmodium infection during pregnancy (LAMP/PCR) (incidence/100 py)*	594, 52/156.7 (33.2)	786, 119/222.2 (53.5)	0.71 (0.46, 1.10)	0.12	0.53 (0.34, 0.84)	0.0071
Maternal patent Plasmodium infection during pregnancy (cumulative risk)	0/594 (0.0)	17/786 (2.2)	.†	.	.†	.
Maternal patent Plasmodium infection during pregnancy (incidence/100 py)*	594, 0/156.7 (0.0)	786, 19/222.2 (8.5)	.†	.	.†	.
Maternal sub-patent Plasmodium infection during pregnancy (cumulative risk)	49/594 (8.2)	87/786 (11.1)	0.81 (0.55, 1.20)	0.29	0.67 (0.45, 0.99)	0.0435
Maternal sub-patent Plasmodium infection during pregnancy (incidence/100 py)*	594, 52/156.7 (33.2)	786, 100/222.2 (45.0)	0.81 (0.53, 1.23)	0.32	0.66 (0.44, 0.99)	0.0423
Maternal clinical malaria during pregnancy (cumulative risk)	2/595 (0.3)	8/786 (1.0)	0.33 (0.07, 1.50)	0.15	0.26 (0.06, 1.18)	0.08
Maternal clinical malaria during pregnancy (incidence/100py)*	595, 2/156.7 (1.3)	786, 9/222.2 (4.0)	0.31 (0.07, 1.36)	0.12	0.22 (0.04, 1.21)	0.08
Maternal non-malaria sick visits during pregnancy (cumulative risk)	45/595 (7.6)	66/786 (8.4)	0.87 (0.54, 1.40)	0.56	0.79 (0.45, 1.37)	0.40
Maternal non-malaria sick visits during pregnancy (incidence/100py)*	595, 49/156.7 (31.3)	786, 74/222.2 (33.3)	0.89 (0.54, 1.47)	0.65	0.74 (0.43, 1.29)	0.29
Maternal all-cause sick visits during pregnancy (cumulative risk)	47/595 (7.9)	73/786 (9.3)	0.84 (0.54, 1.30)	0.43	0.75 (0.44, 1.27)	0.28
Maternal all-cause sick visits during pregnancy (incidence/100py)*	595, 51/156.7 (32.5)	786, 83/222.2 (37.3)	0.83 (0.52, 1.30)	0.41	0.68 (0.40, 1.15)	0.15
2. Sumba						
Maternal Plasmodium infection during pregnancy (LAMP/PCR/smear/RDT) (cumulative risk)	36/276 (13.0)	46/338 (13.6)	0.95 (0.62, 1.45)	0.81	0.83 (0.52, 1.31)	0.42
Maternal Plasmodium infection during pregnancy (LAMP/PCR/smear/RDT) (incidence/100 py)*	276, 39/72.0 (54.2)	338, 54/87.8 (61.5)	0.86 (0.54, 1.37)	0.52	0.74 (0.46, 1.19)	0.21
Maternal Plasmodium infection during pregnancy (fever-RDT) (cumulative risk)	0/276 (0.0)	2/338 (0.6)	.†	.	.†	.
Maternal Plasmodium infection during pregnancy (fever-RDT) (incidence/100 py)*	276, 0/72.0 (0.0)	338, 3/87.8 (3.4)	.†	.	.†	.
Maternal Plasmodium infection during pregnancy (smear) (cumulative risk)	0/276 (0.0)	5/338 (1.5)	.†	.	.†	.
Maternal Plasmodium infection during pregnancy (smear) (incidence/100 py)*	276, 0/72.0 (0.0)	338, 5/87.8 (5.7)	.†	.	.†	.
Maternal Plasmodium infection during pregnancy (LAMP/PCR) (cumulative risk)	36/275 (13.1)	46/338 (13.6)	0.95 (0.62, 1.46)	0.83	0.83 (0.53, 1.32)	0.43
Maternal Plasmodium infection during pregnancy (LAMP/PCR) (incidence/100 py)*	275, 39/72.0 (54.2)	338, 54/87.8 (61.5)	0.86 (0.54, 1.37)	0.52	0.74 (0.46, 1.19)	0.22
Maternal patent Plasmodium infection during pregnancy (cumulative risk)	0/275 (0.0)	5/338 (1.5)	.†	.	.†	.
Maternal patent Plasmodium infection during pregnancy (incidence/100 py)*	275, 0/72.0 (0.0)	338, 6/87.8 (6.8)	.†	.	.†	.
Maternal sub-patent Plasmodium infection during pregnancy (cumulative risk)	36/275 (13.1)	44/338 (13.0)	1.00 (0.66, 1.52)	0.98	0.89 (0.58, 1.36)	0.59
Maternal sub-patent Plasmodium infection during pregnancy (incidence/100 py)*	275, 39/72.0 (54.2)	338, 48/87.8 (54.6)	0.98 (0.64, 1.51)	0.92	0.88 (0.56, 1.37)	0.57
Maternal clinical malaria during pregnancy (cumulative risk)	0/276 (0.0)	2/338 (0.6)	.†	.	.†	.
Maternal clinical malaria during pregnancy (incidence/100py)*	276, 0/72.0 (0.0)	338, 3/87.8 (3.4)	.†	.	.†	.
Maternal non-malaria sick visits during pregnancy (cumulative risk)	26/276 (9.4)	39/338 (11.5)	0.84 (0.51, 1.40)	0.51	0.69 (0.37, 1.29)	0.25
Maternal non-malaria sick visits during pregnancy (incidence/100py)*	276, 28/72.0 (38.9)	338, 42/87.8 (47.8)	0.84 (0.50, 1.41)	0.51	0.64 (0.34, 1.20)	0.17
Maternal all-cause sick visits during pregnancy (cumulative risk)	26/276 (9.4)	40/338 (11.8)	0.82 (0.50, 1.34)	0.42	0.64 (0.35, 1.17)	0.14
Maternal all-cause sick visits during pregnancy (incidence/100py)*	276, 28/72.0 (38.9)	338, 45/87.8 (51.2)	0.77 (0.47, 1.27)	0.31	0.56 (0.31, 1.02)	0.06
3. Papua						
Maternal Plasmodium infection during pregnancy (LAMP/PCR/smear/RDT) (cumulative risk)	17/319 (5.3)	74/448 (16.5)	0.35 (0.22, 0.56)	<.0001	0.35 (0.22, 0.55)	<.0001
Maternal Plasmodium infection during pregnancy (LAMP/PCR/smear/RDT) (incidence/100 py)*	319, 17/84.7 (20.1)	448, 99/134.4 (73.7)	0.31 (0.19, 0.49)	<.0001	0.22 (0.14, 0.34)	<.0001
Maternal Plasmodium infection during pregnancy (fever-RDT) (cumulative risk)	2/319 (0.6)	1/448 (0.2)	2.28 (0.30, 17.21)	0.42	2.48 (0.33, 18.92)	0.38
Maternal Plasmodium infection during pregnancy (fever-RDT) (incidence/100 py)*	319, 2/84.7 (2.4)	448, 1/134.4 (0.7)	2.41 (0.35, 16.77)	0.37	2.42 (0.42, 13.97)	0.32
Maternal Plasmodium infection during pregnancy (smear) (cumulative risk)	4/319 (1.3)	17/448 (3.8)	0.39 (0.09, 1.76)	0.22	0.39 (0.09, 1.77)	0.22
Maternal Plasmodium infection during pregnancy (smear) (incidence/100 py)*	319, 4/84.7 (4.7)	448, 36/134.4 (26.8)	0.22 (0.05, 0.90)	0.0347	0.12 (0.04, 0.40)	0.0006
Maternal Plasmodium infection during pregnancy (LAMP/PCR) (cumulative risk)	13/319 (4.1)	52/448 (11.6)	0.36 (0.23, 0.56)	<.0001	0.36 (0.23, 0.56)	<.0001
Maternal Plasmodium infection during pregnancy (LAMP/PCR) (incidence/100 py)*	319, 13/84.7 (15.3)	448, 65/134.4 (48.4)	0.36 (0.22, 0.60)	<.0001	0.26 (0.15, 0.47)	<.0001
Maternal patent Plasmodium infection during pregnancy (cumulative risk)	0/319 (0.0)	12/448 (2.7)	.†	.	.†	.
Maternal patent Plasmodium infection during pregnancy (incidence/100 py)*	319, 0/84.7 (0.0)	448, 13/134.4 (9.7)	.†	.	.†	.
Maternal sub-patent Plasmodium infection during pregnancy (cumulative risk)	13/319 (4.1)	43/448 (9.6)	0.43 (0.28, 0.67)	0.0002	0.39 (0.24, 0.63)	<.0001
Maternal sub-patent Plasmodium infection during pregnancy (incidence/100 py)*	319, 13/84.7 (15.3)	448, 52/134.4 (38.7)	0.43 (0.26, 0.70)	0.0007	0.35 (0.20, 0.59)	<.0001
Maternal clinical malaria during pregnancy (cumulative risk)	2/319 (0.6)	6/448 (1.3)	0.43 (0.09, 1.99)	0.28	0.43 (0.09, 1.99)	0.28
Maternal clinical malaria during pregnancy (incidence/100py)*	319, 2/84.7 (2.4)	448, 6/134.4 (4.5)	0.48 (0.11, 2.18)	0.34	0.43 (0.09, 1.99)	0.28
Maternal non-malaria sick visits during pregnancy (cumulative risk)	19/319 (6.0)	27/448 (6.0)	0.94 (0.39, 2.29)	0.89	0.93 (0.37, 2.38)	0.89
Maternal non-malaria sick visits during pregnancy (incidence/100py)*	319, 21/84.7 (24.8)	448, 32/134.4 (23.8)	0.91 (0.36, 2.34)	0.85	0.88 (0.33, 2.35)	0.80
Maternal all-cause sick visits during pregnancy (cumulative risk)	21/319 (6.6)	33/448 (7.4)	0.88 (0.41, 1.92)	0.75	0.85 (0.37, 1.97)	0.71
Maternal all-cause sick visits during pregnancy (incidence/100py)*	319, 23/84.7 (27.1)	448, 38/134.4 (28.3)	0.87 (0.39, 1.95)	0.73	0.65 (0.27, 1.58)	0.34

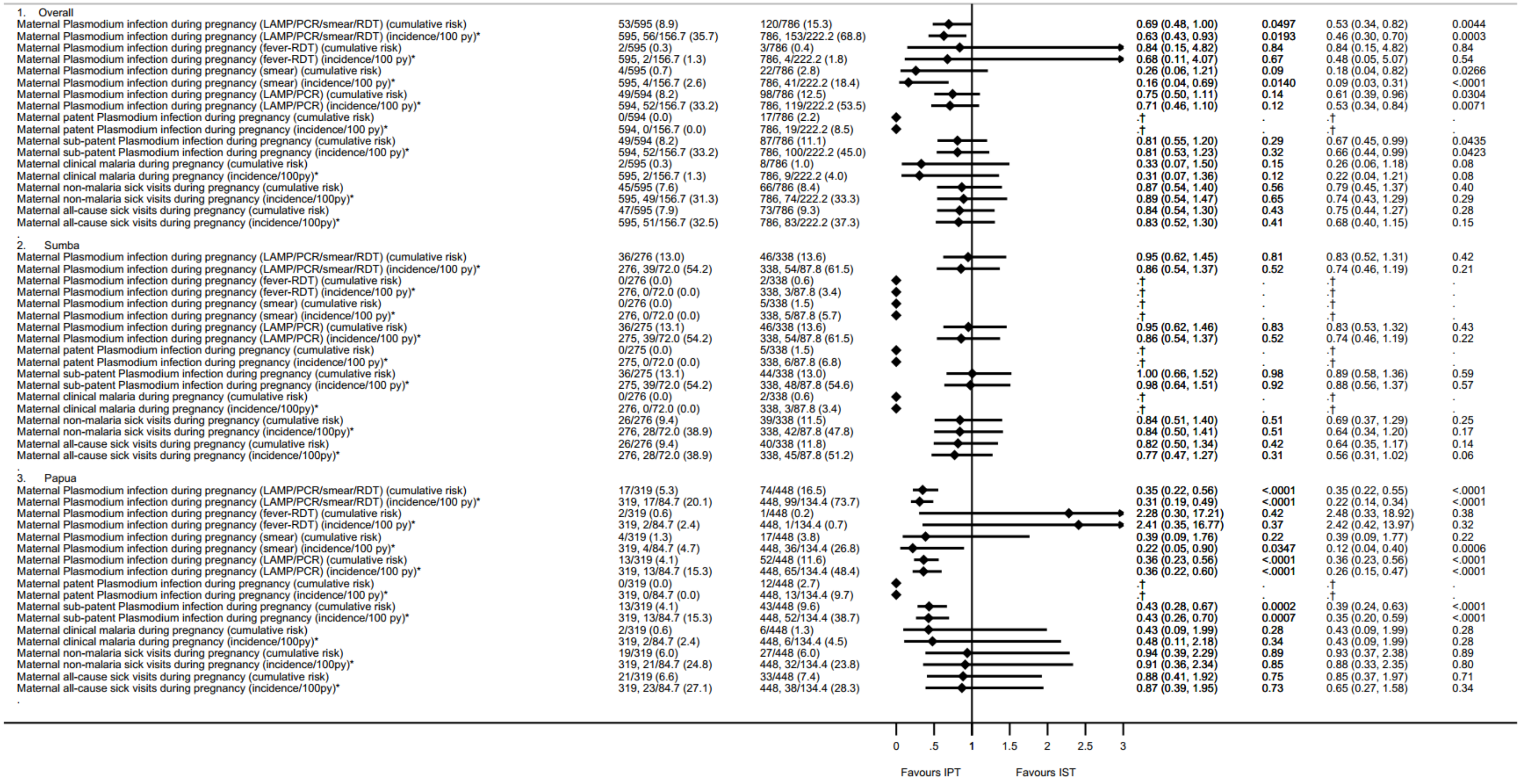

RR=Relative Risk, IRR=Incidence rate ratio, Adjusted RR or IRR obtained using the same covariates as in Figure 2 of the main text, RDT=Rapid diagnostic test for malaria, fever-RDT=RDT taken among women with document fever or a history of fever in the last 48 hours in all 3 arms. * Data represents n/N (%) except for the data for the incidence per 100 person-years which represent the number of women with an event, the number of events, the follow-up person time and in brackets, the incidence rate per 100 person-years. † RR/IRR and p-value could not be computed because of zero events in at least one of the arms.

eFigure 17: Secondary outcomes newborn: birth outcomes and neonatal follow-up (IPT vs SST))

IPT vs SST	IPT n/N (%)	SST, n/N (%)	Crude RR (95% CI)	P-value	Adjusted RR (95% CI)	P-value
Overall						
Congenital malaria (LAMP/PCR/smear/RDT): (age <=7 days)	20/497 (4.0)	28/610 (4.6)	0.88 (0.51, 1.53)	0.66	0.93 (0.57, 1.52)	0.77
Fetal anaemia (cord Hb<12.5 g/dL)	85/487 (17.5)	139/603 (23.1)	0.75 (0.55, 1.01)	0.06	0.79 (0.63, 0.99)	0.0431
Small for gestational age (<10th perc INTERGROWTH)	69/512 (13.5)	71/625 (11.4)	1.18 (0.86, 1.61)	0.32	1.25 (0.98, 1.60)	0.08
Low birth weight (<2,500g)	93/512 (18.2)	86/626 (13.7)	1.35 (0.97, 1.87)	0.07	1.40 (1.05, 1.86)	0.0207
Preterm birth (<37 weeks)	61/524 (11.6)	70/635 (11.0)	1.06 (0.74, 1.53)	0.74	1.13 (0.81, 1.56)	0.47
Adverse livebirth outcome (LBW/SGA/PT)	140/524 (26.7)	142/635 (22.4)	1.20 (0.94, 1.53)	0.14	1.27 (1.05, 1.55)	0.0153
Fetal loss (spontaneous abortion or stillbirth)	13/561 (2.3)	9/662 (1.4)	1.72 (0.68, 4.32)	0.25	1.76 (0.71, 4.34)	0.22
Adverse pregnancy outcome (fetal loss, LBW/SGA/PT)	153/561 (27.3)	151/663 (22.8)	1.23 (0.97, 1.55)	0.09	1.27 (1.06, 1.53)	0.0112
Perinatal death	15/536 (2.8)	12/642 (1.9)	1.50 (0.68, 3.32)	0.32	1.54 (0.71, 3.32)	0.28
Neonatal death (<28 days)	5/524 (1.0)	7/634 (1.1)	0.82 (0.17, 3.90)	0.80	0.94 (0.21, 4.25)	0.93
Infant mortality by end of follow-up (6-8 weeks of age)	6/524 (1.1)	8/635 (1.3)	0.91 (0.25, 3.34)	0.89	1.01 (0.29, 3.53)	0.98
Adverse pregnancy outcome (fetal loss, LBW/SGA/PT, neonatal death by 4 weeks)‡	153/561 (27.3)	153/663 (23.1)	1.22 (0.96, 1.54)	0.10	1.26 (1.04, 1.52)	0.0175
Adverse pregnancy outcome (fetal loss, LBW/SGA/PT, infant death by 6-8 weeks)‡	154/561 (27.5)	153/663 (23.1)	1.22 (0.97, 1.55)	0.09	1.26 (1.04, 1.52)	0.0155
.						
Sumba						
Congenital malaria (LAMP/PCR/smear/RDT): (age <=7 days)	13/247 (5.3)	14/283 (4.9)	1.09 (0.60, 1.99)	0.78	1.08 (0.62, 1.90)	0.78
Fetal anaemia (cord Hb<12.5 g/dL)	30/243 (12.3)	47/278 (16.9)	0.73 (0.46, 1.16)	0.18	0.73 (0.46, 1.14)	0.16
Small for gestational age (<10th perc INTERGROWTH)	40/243 (16.5)	37/287 (12.9)	1.32 (0.90, 1.95)	0.16	1.38 (0.94, 2.02)	0.10
Low birth weight (<2,500g)	62/243 (25.5)	42/287 (14.6)	1.77 (1.29, 2.42)	0.0004	1.84 (1.35, 2.51)	<.0001
Preterm birth (<37 weeks)	37/250 (14.8)	32/289 (11.1)	1.33 (0.86, 2.07)	0.21	1.38 (0.88, 2.16)	0.16
Adverse livebirth outcome (LBW/SGA/PT)	86/250 (34.4)	71/289 (24.6)	1.41 (1.10, 1.80)	0.0062	1.45 (1.14, 1.85)	0.0028
Fetal loss (spontaneous abortion or stillbirth)	8/258 (3.1)	4/293 (1.4)	2.25 (0.67, 7.57)	0.19	2.29 (0.66, 7.96)	0.19
Adverse pregnancy outcome (fetal loss, LBW/SGA/PT)	94/258 (36.4)	75/293 (25.6)	1.43 (1.13, 1.79)	0.0024	1.45 (1.16, 1.83)	0.0014
Perinatal death	7/257 (2.7)	4/292 (1.4)	1.98 (0.56, 7.03)	0.29	1.99 (0.56, 7.07)	0.29
Neonatal death (<28 days)	0/250 (0.0)	0/288 (0.0)	.†	.	.†	.
Infant mortality by end of follow-up (6-8 weeks of age)	1/250 (0.4)	0/288 (0.0)	.†	.	.†	.
Adverse pregnancy outcome (fetal loss, LBW/SGA/PT, neonatal death by 4 weeks)‡	94/258 (36.4)	75/293 (25.6)	1.43 (1.13, 1.79)	0.0024	1.45 (1.16, 1.83)	0.0013
Adverse pregnancy outcome (fetal loss, LBW/SGA/PT, infant death by 6-8 weeks)‡	95/258 (36.8)	75/293 (25.6)	1.44 (1.15, 1.81)	0.0016	1.47 (1.17, 1.84)	0.0009
.						
Papua						
Congenital malaria (LAMP/PCR/smear/RDT): (age <=7 days)	7/250 (2.8)	14/327 (4.3)	0.66 (0.28, 1.55)	0.34	0.77 (0.34, 1.76)	0.53
Fetal anaemia (cord Hb<12.5 g/dL)	55/244 (22.5)	92/325 (28.3)	0.79 (0.59, 1.06)	0.11	0.77 (0.56, 1.04)	0.09
Small for gestational age (<10th perc INTERGROWTH)	29/269 (10.8)	34/338 (10.1)	1.08 (0.71, 1.63)	0.73	1.28 (0.84, 1.96)	0.25
Low birth weight (<2,500g)	31/269 (11.5)	44/339 (13.0)	0.84 (0.51, 1.40)	0.50	0.72 (0.40, 1.31)	0.28
Preterm birth (<37 weeks)	24/274 (8.8)	38/346 (11.0)	0.77 (0.41, 1.46)	0.43	0.77 (0.40, 1.47)	0.43
Adverse livebirth outcome (LBW/SGA/PT)	54/274 (19.7)	71/346 (20.5)	0.90 (0.59, 1.39)	0.64	0.92 (0.53, 1.58)	0.75
Fetal loss (spontaneous abortion or stillbirth)	5/303 (1.7)	5/369 (1.4)	1.08 (0.29, 3.96)	0.91	1.42 (0.36, 5.56)	0.62
Adverse pregnancy outcome (fetal loss, LBW/SGA/PT)	59/303 (19.5)	76/370 (20.5)	0.90 (0.60, 1.33)	0.58	0.93 (0.58, 1.48)	0.75
Perinatal death	8/279 (2.9)	8/350 (2.3)	1.13 (0.41, 3.07)	0.82	1.50 (0.60, 3.74)	0.39
Neonatal death (<28 days)	5/274 (1.8)	7/346 (2.0)	0.86 (0.22, 3.45)	0.84	0.86 (0.21, 3.43)	0.82
Infant mortality by end of follow-up (6-8 weeks of age)	5/274 (1.8)	8/347 (2.3)	0.79 (0.22, 2.86)	0.72	0.79 (0.22, 2.85)	0.72
Adverse pregnancy outcome (fetal loss, LBW/SGA/PT, neonatal death by 4 weeks)‡	59/303 (19.5)	78/370 (21.1)	0.88 (0.59, 1.30)	0.52	0.90 (0.57, 1.43)	0.67
Adverse pregnancy outcome (fetal loss, LBW/SGA/PT, infant death by 6-8 weeks)‡	59/303 (19.5)	78/370 (21.1)	0.88 (0.59, 1.30)	0.52	0.90 (0.57, 1.43)	0.67
.						

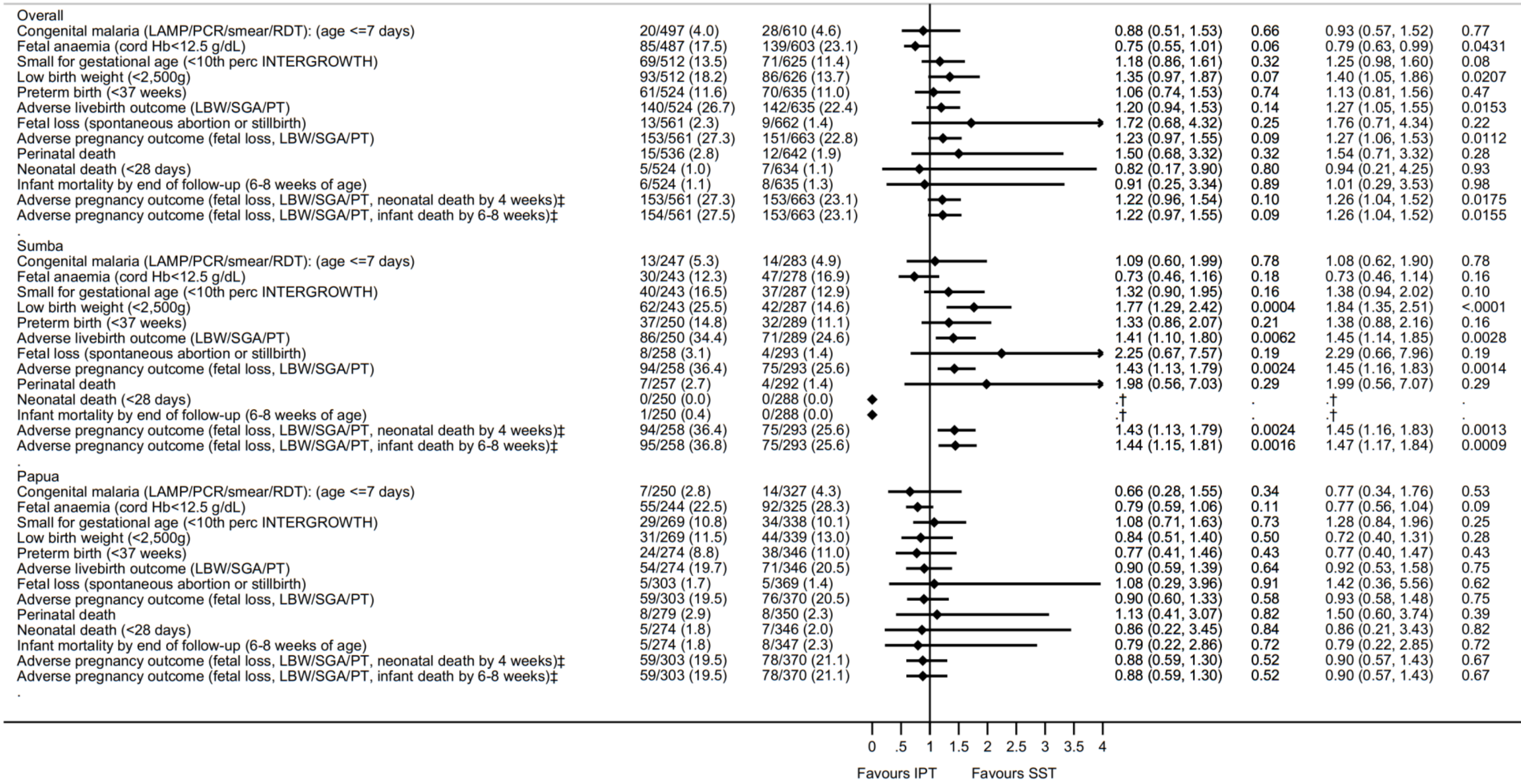

RR=Relative Risk. * Data for represents the n/N (%). Adjusted RR obtained using the same covariates as outlined in Figure 2, under post-hoc adjusted analysis. † RR and p-value could not be computed because of zero events in at least one of the arms. ‡ outcome variable defined post-hoc.

eFigure 18: Secondary outcomes newborn: birth outcomes and neonatal follow-up (IST vs SST)

IST vs SST	IST n/N (%)	SST, n/N (%)	Crude RR (95% CI)	P-value	Adjusted RR (95% CI)	P-value
Overall						
Congenital malaria (LAMP/PCR/smear/RDT): (age <=7 days)	22/691 (3.2)	28/610 (4.6)	0.74 (0.40, 1.35)	0.32	0.88 (0.51, 1.53)	0.66
Fetal anaemia (cord Hb<12.5 g/dL)	130/680 (19.1)	139/603 (23.1)	0.81 (0.62, 1.07)	0.14	0.81 (0.67, 0.99)	0.0342
Small for gestational age (<10th perc INTERGROWTH)	69/695 (9.9)	71/625 (11.4)	0.88 (0.63, 1.22)	0.44	0.90 (0.67, 1.21)	0.48
Low birth weight (<2,500g)	81/698 (11.6)	86/626 (13.7)	0.88 (0.63, 1.24)	0.47	1.00 (0.75, 1.34)	1.00
Preterm birth (<37 weeks)	73/713 (10.2)	70/635 (11.0)	1.00 (0.70, 1.43)	0.99	0.99 (0.71, 1.37)	0.94
Adverse livebirth outcome (LBW/SGA/PT)	145/713 (20.3)	142/635 (22.4)	0.95 (0.73, 1.24)	0.71	0.99 (0.79, 1.23)	0.91
Foetal loss (spontaneous abortion or stillbirth)	16/752 (2.1)	9/662 (1.4)	1.62 (0.64, 4.10)	0.31	1.60 (0.61, 4.20)	0.34
Adverse pregnancy outcome (fetal loss, LBW/SGA/PT)	161/752 (21.4)	151/663 (22.8)	1.00 (0.77, 1.28)	0.98	1.01 (0.83, 1.25)	0.89
Perinatal death	25/726 (3.4)	12/642 (1.9)	1.94 (0.90, 4.17)	0.09	2.12 (1.04, 4.34)	0.0391
Neonatal death (<28 days)	15/712 (2.1)	7/634 (1.1)	2.14 (0.78, 5.84)	0.14	2.76 (0.97, 7.81)	0.06
Infant mortality by end of follow-up (6-8 weeks of age)	16/712 (2.2)	8/635 (1.3)	1.89 (0.79, 4.55)	0.15	2.19 (0.90, 5.34)	0.08
Adverse pregnancy outcome (fetal loss, LBW/SGA/PT, neonatal death by 4 weeks)‡	168/752 (22.3)	153/663 (23.1)	1.03 (0.80, 1.32)	0.82	1.06 (0.86, 1.30)	0.61
Adverse pregnancy outcome (fetal loss, LBW/SGA/PT, infant death by 6-8 weeks)‡	169/752 (22.5)	153/663 (23.1)	1.03 (0.81, 1.32)	0.80	1.06 (0.86, 1.30)	0.60
.						
Sumba						
Congenital malaria (LAMP/PCR/smear/RDT): (age <=7 days)	15/279 (5.4)	14/283 (4.9)	1.10 (0.59, 2.06)	0.76	1.14 (0.64, 2.03)	0.65
Foetal anaemia (cord Hb<12.5 g/dL)	35/274 (12.8)	47/278 (16.9)	0.76 (0.53, 1.08)	0.13	0.77 (0.52, 1.14)	0.19
Small for gestational age (<10th perc INTERGROWTH)	35/276 (12.7)	37/287 (12.9)	1.00 (0.68, 1.48)	0.98	0.96 (0.67, 1.39)	0.84
Low birth weight (<2,500g)	44/278 (15.8)	42/287 (14.6)	1.08 (0.76, 1.54)	0.67	1.19 (0.85, 1.68)	0.31
Preterm birth (<37 weeks)	46/287 (16.0)	32/289 (11.1)	1.45 (0.95, 2.20)	0.08	1.38 (0.88, 2.16)	0.16
Adverse livebirth outcome (LBW/SGA/PT)	81/287 (28.2)	71/289 (24.6)	1.16 (0.88, 1.51)	0.29	1.09 (0.85, 1.41)	0.49
Foetal loss (spontaneous abortion or stillbirth)	8/295 (2.7)	4/293 (1.4)	1.98 (0.60, 6.50)	0.26	1.57 (0.49, 5.07)	0.45
Adverse pregnancy outcome (fetal loss, LBW/SGA/PT)	89/295 (30.2)	75/293 (25.6)	1.18 (0.92, 1.52)	0.18	1.11 (0.87, 1.42)	0.38
Perinatal death	14/293 (4.8)	4/292 (1.4)	3.48 (1.15, 10.55)	0.0273	2.88 (0.99, 8.39)	0.05
Neonatal death (<28 days)	9/286 (3.1)	0/288 (0.0)	.†	.	.†	.
Infant mortality by end of follow-up (6-8 weeks of age)	9/286 (3.1)	0/288 (0.0)	.†	.	.†	.
Adverse pregnancy outcome (fetal loss, LBW/SGA/PT, neonatal death by 4 weeks)‡	93/295 (31.5)	75/293 (25.6)	1.24 (0.97, 1.58)	0.08	1.17 (0.92, 1.48)	0.20
Adverse pregnancy outcome (fetal loss, LBW/SGA/PT, infant death by 6-8 weeks)‡	93/295 (31.5)	75/293 (25.6)	1.24 (0.97, 1.58)	0.08	1.17 (0.92, 1.48)	0.20
.						
Papua						
Congenital malaria (LAMP/PCR/smear/RDT): (age <=7 days)	7/412 (1.7)	14/327 (4.3)	0.40 (0.16, 1.02)	0.06	0.50 (0.19, 1.29)	0.15
Fetal anaemia (cord Hb<12.5 g/dL)	95/406 (23.4)	92/325 (28.3)	0.82 (0.62, 1.09)	0.17	0.78 (0.61, 1.00)	0.06
Small for gestational age (<10th perc INTERGROWTH)	34/419 (8.1)	34/338 (10.1)	0.81 (0.50, 1.31)	0.38	0.89 (0.57, 1.39)	0.61
Low birth weight (<2,500g)	37/420 (8.8)	44/339 (13.0)	0.66 (0.37, 1.19)	0.17	0.56 (0.28, 1.12)	0.10
Preterm birth (<37 weeks)	27/426 (6.3)	38/346 (11.0)	0.57 (0.36, 0.90)	0.0159	0.56 (0.35, 0.90)	0.0170
Adverse livebirth outcome (LBW/SGA/PT)	64/426 (15.0)	71/346 (20.5)	0.70 (0.44, 1.11)	0.13	0.71 (0.37, 1.36)	0.30
Fetal loss (spontaneous abortion or stillbirth)	8/457 (1.8)	5/369 (1.4)	1.27 (0.31, 5.14)	0.74	2.04 (0.56, 7.41)	0.28
Adverse pregnancy outcome (fetal loss, LBW/SGA/PT)	72/457 (15.8)	76/370 (20.5)	0.75 (0.49, 1.14)	0.17	0.77 (0.44, 1.37)	0.38
Perinatal death	11/433 (2.5)	8/350 (2.3)	1.06 (0.34, 3.31)	0.92	1.61 (0.58, 4.50)	0.36
Neonatal death (<28 days)	6/426 (1.4)	7/346 (2.0)	0.66 (0.21, 2.04)	0.47	0.65 (0.21, 2.02)	0.46
Infant mortality by end of follow-up (6-8 weeks of age)	7/426 (1.6)	8/347 (2.3)	0.72 (0.30, 1.70)	0.45	0.72 (0.30, 1.69)	0.44
Adverse pregnancy outcome (fetal loss, LBW/SGA/PT, neonatal death by 4 weeks)‡	75/457 (16.4)	78/370 (21.1)	0.76 (0.50, 1.14)	0.18	0.78 (0.45, 1.37)	0.39
Adverse pregnancy outcome (fetal loss, LBW/SGA/PT, infant death by 6-8 weeks)‡	76/457 (16.6)	78/370 (21.1)	0.77 (0.51, 1.15)	0.20	0.79 (0.45, 1.38)	0.41
.						

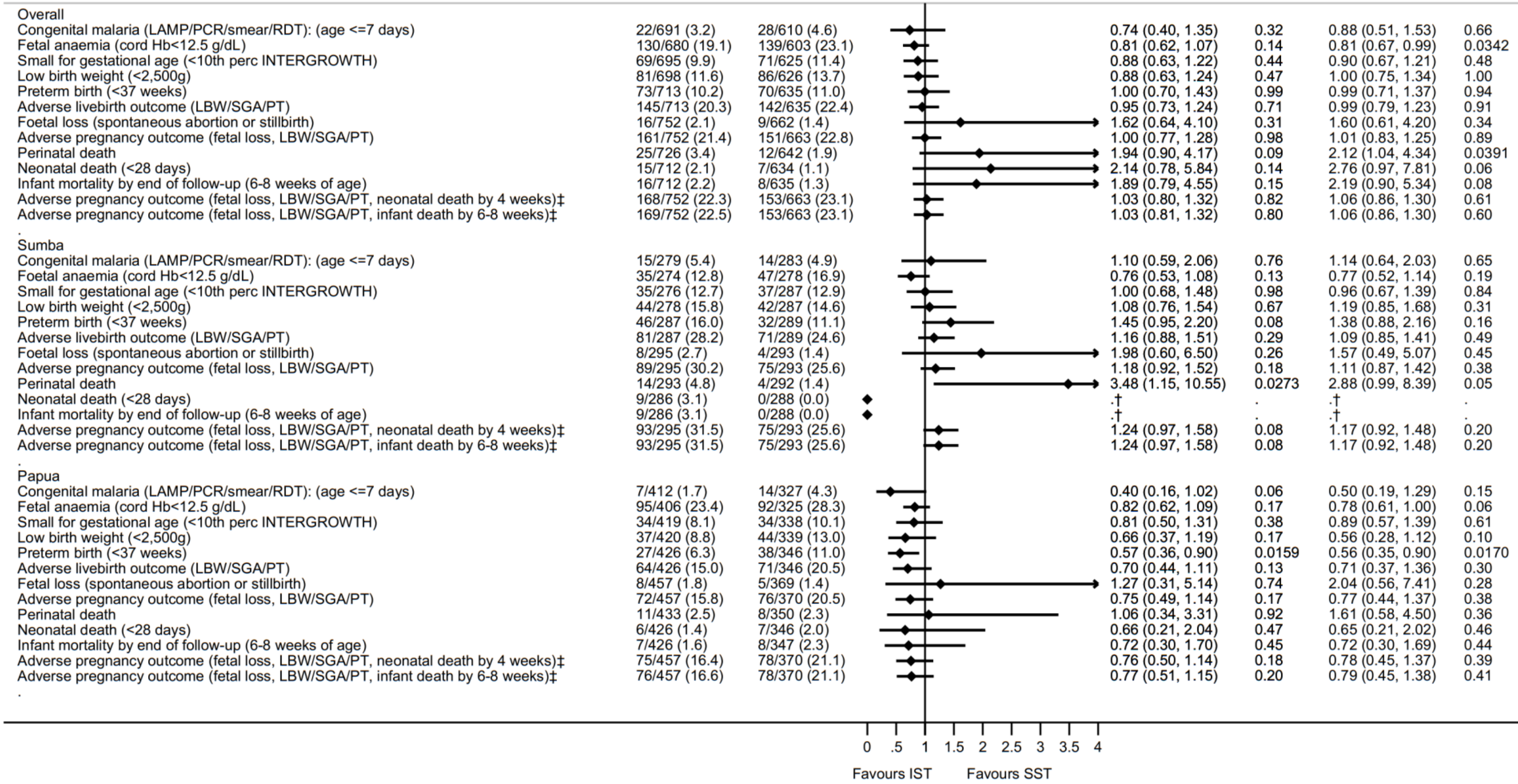

RR=Relative Risk. * Data for represents the n/N (%). Adjusted RR obtained using the same covariates as outlined in Figure 2, under post-hoc adjusted analysis. † RR and p-value could not be computed because of zero events in at least one of the arms. ‡ outcome variable defined post-hoc.

eFigure 19: Secondary outcomes newborn: birth outcomes and neonatal follow-up (IPT vs IST)

IPT vs IST	IPT n/N (%)	IST, n/N (%)	Crude RR (95% CI)	P-value	Adjusted RR (95% CI)	P-value
Overall						
Congenital malaria (LAMP/PCR/smear/RDT): (age <=7 days)	20/497 (4.0)	22/691 (3.2)	1.20 (0.61, 2.37)	0.59	1.05 (0.57, 1.95)	0.88
Fetal anaemia (cord Hb<12.5 g/dL)	85/487 (17.5)	130/680 (19.1)	0.92 (0.66, 1.27)	0.61	0.97 (0.78, 1.21)	0.78
Small for gestational age (<10th perc INTERGROWTH)	69/512 (13.5)	69/695 (9.9)	1.34 (0.97, 1.86)	0.08	1.39 (1.06, 1.81)	0.0152
Low birth weight (<2,500g)	93/512 (18.2)	81/698 (11.6)	1.53 (1.09, 2.14)	0.0140	1.40 (1.09, 1.80)	0.0093
Preterm birth (<37 weeks)	61/524 (11.6)	73/713 (10.2)	1.07 (0.73, 1.56)	0.74	1.14 (0.81, 1.61)	0.45
Adverse livebirth outcome (LBW/SGA/PT)	140/524 (26.7)	145/713 (20.3)	1.26 (0.97, 1.64)	0.09	1.29 (1.06, 1.58)	0.0121
Fetal loss (spontaneous abortion or stillbirth)	13/561 (2.3)	16/752 (2.1)	1.06 (0.51, 2.20)	0.87	1.10 (0.49, 2.47)	0.82
Adverse pregnancy outcome (fetal loss, LBW/SGA/PT)	153/561 (27.3)	161/752 (21.4)	1.23 (0.95, 1.60)	0.12	1.25 (1.03, 1.53)	0.0247
Perinatal death	15/536 (2.8)	25/726 (3.4)	0.77 (0.40, 1.51)	0.45	0.72 (0.36, 1.45)	0.36
Neonatal death (<28 days)	5/524 (1.0)	15/712 (2.1)	0.38 (0.10, 1.51)	0.17	0.34 (0.09, 1.33)	0.12
Infant mortality by end of follow-up (6-8 weeks of age)	6/524 (1.1)	16/712 (2.2)	0.48 (0.16, 1.48)	0.20	0.46 (0.15, 1.44)	0.18
Adverse pregnancy outcome (fetal loss, LBW/SGA/PT, neonatal death by 4 weeks)‡	153/561 (27.3)	168/752 (22.3)	1.18 (0.91, 1.53)	0.20	1.19 (0.98, 1.45)	0.08
Adverse pregnancy outcome (fetal loss, LBW/SGA/PT, infant death by 6-8 weeks)‡	154/561 (27.5)	169/752 (22.5)	1.19 (0.92, 1.54)	0.19	1.20 (0.98, 1.46)	0.08
.						
Sumba						
Congenital malaria (LAMP/PCR/smear/RDT): (age <=7 days)	13/247 (5.3)	15/279 (5.4)	0.99 (0.47, 2.06)	0.97	0.95 (0.48, 1.87)	0.88
Fetal anaemia (cord Hb<12.5 g/dL)	30/243 (12.3)	35/274 (12.8)	0.97 (0.57, 1.64)	0.90	0.94 (0.54, 1.65)	0.83
Small for gestational age (<10th perc INTERGROWTH)	40/243 (16.5)	35/276 (12.7)	1.32 (0.90, 1.93)	0.16	1.43 (0.95, 2.16)	0.09
Low birth weight (<2,500g)	62/243 (25.5)	44/278 (15.8)	1.64 (1.21, 2.21)	0.0014	1.54 (1.17, 2.04)	0.0023
Preterm birth (<37 weeks)	37/250 (14.8)	46/287 (16.0)	0.92 (0.65, 1.31)	0.64	1.00 (0.65, 1.55)	0.99
Adverse livebirth outcome (LBW/SGA/PT)	86/250 (34.4)	81/287 (28.2)	1.22 (0.97, 1.53)	0.09	1.33 (1.04, 1.69)	0.0219
Fetal loss (spontaneous abortion or stillbirth)	8/258 (3.1)	8/295 (2.7)	1.14 (0.45, 2.90)	0.79	1.46 (0.47, 4.57)	0.51
Adverse pregnancy outcome (fetal loss, LBW/SGA/PT)	94/258 (36.4)	89/295 (30.2)	1.20 (0.96, 1.51)	0.10	1.30 (1.02, 1.67)	0.0351
Perinatal death	7/257 (2.7)	14/293 (4.8)	0.57 (0.23, 1.40)	0.22	0.69 (0.24, 2.00)	0.50
Neonatal death (<28 days)	0/250 (0.0)	9/286 (3.1)	.†	.	.†	.
Infant mortality by end of follow-up (6-8 weeks of age)	1/250 (0.4)	9/286 (3.1)	0.16 (0.03, 1.04)	0.05	0.16 (0.02, 1.20)	0.08
Adverse pregnancy outcome (fetal loss, LBW/SGA/PT, neonatal death by 4 weeks)‡	94/258 (36.4)	93/295 (31.5)	1.15 (0.93, 1.43)	0.20	1.24 (0.98, 1.58)	0.08
Adverse pregnancy outcome (fetal loss, LBW/SGA/PT, infant death by 6-8 weeks)‡	95/258 (36.8)	93/295 (31.5)	1.17 (0.94, 1.45)	0.16	1.26 (0.99, 1.60)	0.06
.						
Papua						
Congenital malaria (LAMP/PCR/smear/RDT): (age <=7 days)	7/250 (2.8)	7/412 (1.7)	1.64 (0.59, 4.55)	0.34	1.55 (0.56, 4.25)	0.40
Fetal anaemia (cord Hb<12.5 g/dL)	55/244 (22.5)	95/406 (23.4)	0.96 (0.73, 1.26)	0.76	0.98 (0.76, 1.26)	0.85
Small for gestational age (<10th perc INTERGROWTH)	29/269 (10.8)	34/419 (8.1)	1.33 (0.88, 2.01)	0.17	1.44 (1.00, 2.08)	0.05
Low birth weight (<2,500g)	31/269 (11.5)	37/420 (8.8)	1.28 (0.73, 2.23)	0.39	1.29 (0.71, 2.32)	0.41
Preterm birth (<37 weeks)	24/274 (8.8)	27/426 (6.3)	1.36 (0.74, 2.50)	0.32	1.36 (0.73, 2.53)	0.33
Adverse livebirth outcome (LBW/SGA/PT)	54/274 (19.7)	64/426 (15.0)	1.29 (0.82, 2.01)	0.27	1.29 (0.83, 2.01)	0.26
Fetal loss (spontaneous abortion or stillbirth)	5/303 (1.7)	8/457 (1.8)	0.85 (0.30, 2.37)	0.75	0.69 (0.25, 1.94)	0.49
Adverse pregnancy outcome (fetal loss, LBW/SGA/PT)	59/303 (19.5)	72/457 (15.8)	1.20 (0.80, 1.81)	0.38	1.20 (0.79, 1.81)	0.39
Perinatal death	8/279 (2.9)	11/433 (2.5)	1.06 (0.38, 2.96)	0.92	0.93 (0.36, 2.42)	0.88
Neonatal death (<28 days)	5/274 (1.8)	6/426 (1.4)	1.32 (0.32, 5.44)	0.70	1.31 (0.32, 5.42)	0.71
Infant mortality by end of follow-up (6-8 weeks of age)	5/274 (1.8)	7/426 (1.6)	1.11 (0.31, 3.94)	0.87	1.10 (0.31, 3.91)	0.88
Adverse pregnancy outcome (fetal loss, LBW/SGA/PT, neonatal death by 4 weeks)‡	59/303 (19.5)	75/457 (16.4)	1.16 (0.78, 1.73)	0.46	1.15 (0.76, 1.75)	0.50
Adverse pregnancy outcome (fetal loss, LBW/SGA/PT, infant death by 6-8 weeks)‡	59/303 (19.5)	76/457 (16.6)	1.15 (0.78, 1.69)	0.49	1.15 (0.76, 1.72)	0.52
.						

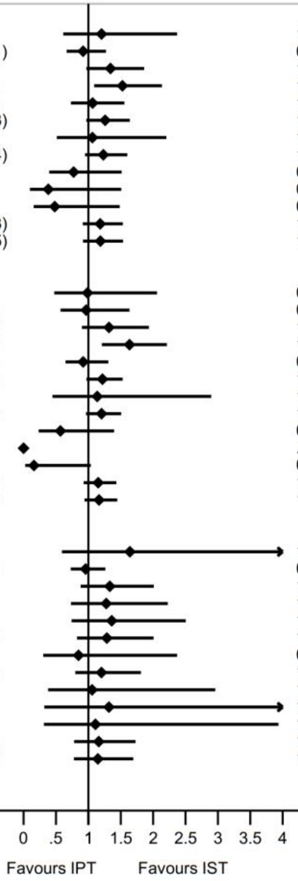

RR=Relative Risk. * Data for represents the n/N (%). Adjusted RR obtained using the same covariates as outlined in Figure 2, under post-hoc adjusted analysis. † RR and p-value could not be computed because of zero events in at least one of the arms. ‡ outcome variable defined post-hoc.

Cardiac Monitoring results

Results (text)

Between 16 Nov 2015 and 21 July 2016, 33 pregnant women in their 2nd and 3rd trimester were recruited. All weighed between 36 and 75 kg and thus received 3 tablets containing a total of 960 mg piperaquine and 120 mg dihydroartemisinin. The mean (SD) dose of piperaquine in mg/kg received was 16.7 (2.2) (range 13.2-21.8). The mean dose in mg/kg declined slightly with successive monthly courses as women gained weight over the course of the pregnancy, yet remained within the 36-75 weight band.

Except for one woman, all women were afebrile at the time of drug administration; the one woman who was febrile, had an axillary temperature (37.7 °C) at enrolment (first course) and had *P. falciparum* parasites on the malaria smear. Out of the 33 women, 5, 5, 13, 9 and 1 received 1, 2, 3, 4, or 5 courses respectively. A total of 126 ECGs were taken in triplicate. There were no clinical cardiac adverse events

Overall, the best correction of the QT interval for heart rate was obtained with Bazett's formula. The heart rate differed slightly during the course of pregnancy, and was highest during the first baseline visit resulting in a lower QTc value with Fridericia's method (eTable 14). The Pearson correlation coefficients for the correlation between QTc interval and RR interval or heart rate (HR) were: QTcF and HR: r=-0.39, p<0.0001; QTcF and RR: r= 0.36, p<0.0001, QTcB and HR: r=0.06, p=0.530; QTcB and RR: r= 0.09, p=0.321.

The mean (SD) QTcF was 408ms (15) at baseline and increased to 435ms (18), 4-6 hours after the last dose of the first course (eTable 14), representing a mean increase of 27.1ms (95% CI 19.6-20.2) (range -9 to 69.7), p<0.0001 (paired t-test). The corresponding values with Bazett's method were generally higher for the absolute QTc values, but lower for the mean increase compared to baseline: The mean (SD) QTcB was 437ms (15) at baseline and increased to 457ms (20) at Tmax (eTable 14), representing a mean increase of 20.0ms (95% CI 13.7-25.0) (range -14 to 55).

Overall the mean (SD) increase in QTcF across all courses was 20ms (19.6) (range -20 to 70.7) and this was 14.8(17.6) (range -25.3-63.3) with Bazett's method.

There was no evidence that the mean increase in QTcF or QTcB increased with subsequent courses (eTable 14) and eFigure 20).

With Fridericia's methods, two women had QTcF values exceeding 480ms and none had values above 500 ms. Among the two women with values exceeding 480ms, this occurred after the first course of DP in one woman, when her QTcF was 484ms compared to 426ms at baseline, a 58ms (13.6%) increase. In the other woman the QTcF increased from 418 at baseline to 443 after the first course, and 475 and 489 after the 2nd and 3rd course respectively, which was a 24, 57 and 71ms increase compared to baseline. Both women had a normal sinus rhythm and no other abnormalities on the ECG. Neither of these women received a subsequent course. The latter woman was the only woman who showed a consistent increase in the QTcF values with each subsequent course. All other women showed no change or a relative decline in the magnitude of QTcF prolongation with each subsequent course.

With Bazett's method there were 7 women with QTc values above 480 ms; None at baseline, 4 after the 1st course (12.1%), 2 after the 2nd (7.1%) and 1 after the 3rd course (4.4%). Among 2 of these the

value exceeded 500 ms. These were the same two women with values exceeding 480 ms with the Fridericia's method.

There was no statistically significant correlation between the increase in QTc interval following each dose and age, haemoglobin concentration, axillary temperature, or dose in mg/kg received (i.e. bodyweight). The Pearson correlation coefficients for the correlation between QTc interval and age, haemoglobin, body temperature and dose in mg/kg respectively were: QTcF: r=-0.1176 (p=0.2589); r= -0.04 (p=0.8240), r= 0.09 (=0.3932); r= 0.02 (p=0.8289), and QTcB: r=-0.04 (p=0.6793); r= -0.03 (p=0.8895), r= 0.07 (=0.5232); r= -0.05 (p=0.6472). ms=milliseconds

Conclusion cardiac monitoring

DP was associated with a mean prolongation of 20ms of the QTcF interval and 15ms of the QTcB, in pregnant women receiving DP for IPT in Papua Indonesia. Sensitivity analysis showed that Bazett's method resulted in better rate correction of the QT values than Fridericia's method. There were no clinical cardiac adverse events. There was one woman with successive asymptomatic increases in QTc interval with subsequent courses, but overall there was no evidence that the QTc prolongation increases with successive number of monthly courses of DP with either Fridericia's or Bazett's method.

eTable 14: Cardiac monitoring; mean and mean change in QTcF and QTcB at baseline and 4 to 6 hours after the third dose of each course

			Fridericia's method				Bazett's method			
			QTcF in ms		Δ in QTcF from baseline* in ms		QTcB in ms		Δ in QTcB from baseline* in ms	
DP course	Day and time ECG	Number of women	Mean (SD)	Range	Mean (95% CI)	p-value	Mean (SD)	Range	mean (95% CI)	p-value
1st	0 (baseline)	33	408 (15)	386-436	Reference		437 (15)	413-466	Reference	
1st	2+4h	33	435 (18)	410-484	27 (20, 34)	<0.0001	457 (20)	423-510	20 (14, 26)	<0.0001
2nd	2+4h	28	430 (19)	389-475	22 (15, 29)	<0.0001	453 (19)	407-499	17 (11, 24)	<0.0001
3rd	2+4h	23	421 (21)	390-489	14 (6, 2)	0.0014	447 (23)	397-513	11 (3, 9)	0.0064
4th	2+4h	10	412 (19)	381-452	7 (-5, 9)	0.21	434 (17)	409-468	1 (-9, 11)	0.8
5th	2+4h	1	398	NA	-3	-	421	NA	3	-

DP=dihydroartemisinin-piperaquine, ECG=Electrocardiogram, QTc QT interval on ECG corrected using the Fridericia method (QTcF) or the Bazett's method (QTcB), ms=milliseconds, SD=standard deviation, 2+4h=Day 2 plus 4 to 6 hours after the third (last) dose of each course of DP (Tmax), NA=not applicable

** Δ=change in QTc from baseline in ms. Baseline was the QTc interval taken just before the first dose of the first course of DP

eFigure 20: Cardiac monitoring; mean (95% CI) QTc at baseline (before the first course of IPT-DP) and 4-6 hours after the last dose of each course by visit number (left panel) and mean (95% CI) change in QTc after each course compared to baseline using Fridericia's method (top panels) and Bazett's method (bottom panels)

Fridericia's method

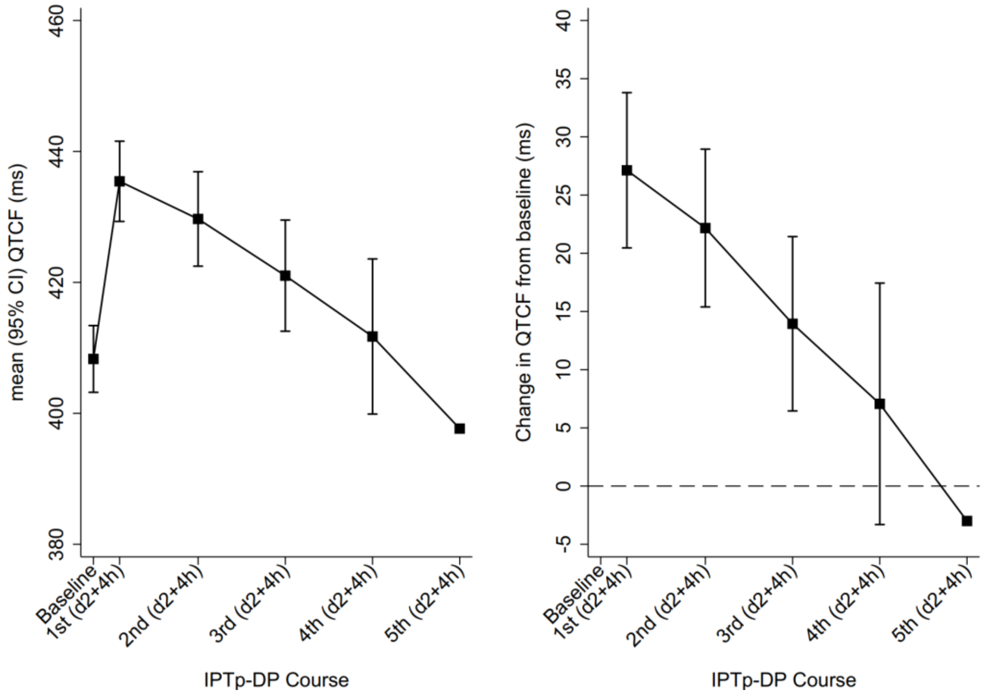

Bazett's method

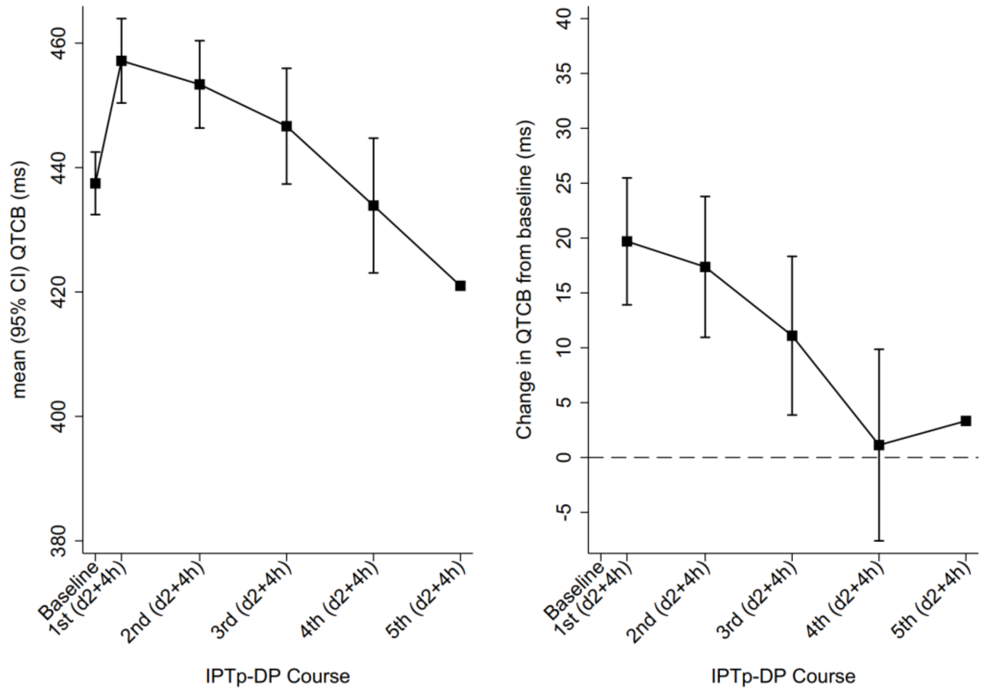

The solid line depicts the mean and the error bars the 95% confidence intervals

QTcF=QT interval on ECG corrected using Fridericia method, QTcB=QT interval on ECG corrected using Bazett's method; D2=day 2; 4h=4 to 6 hours after the last dose of each course (Tmax); ms=milliseconds

eReferences

1. Elyazar IR, Gething PW, Patil AP, et al. Plasmodium falciparum malaria endemicity in Indonesia in 2010. *PloS one* 2011; **6**(6): e21315.
2. Elyazar IR, Gething PW, Patil AP, et al. Plasmodium vivax malaria endemicity in Indonesia in 2010. *PloS one* 2012; **7**(5): e37325.
3. Pava Z, Burdam FH, Handayuni I, et al. Submicroscopic and Asymptomatic Plasmodium Parasitaemia Associated with Significant Risk of Anaemia in Papua, Indonesia. *PloS one* 2016; **11**(10): e0165340.
4. Syafruddin D, Krisin, Asih P, et al. Seasonal prevalence of malaria in West Sumba district, Indonesia. *Malaria journal* 2009; **8**: 8.
5. Ahmed R, Levy EI, Maratina SS, et al. Performance of four HRP-2/pLDH combination rapid diagnostic tests and field microscopy as screening tests for malaria in pregnancy in Indonesia: a cross-sectional study. *Malaria journal* 2015; **14**(1): 420.
6. Ratcliff A, Siswantoro H, Kenangalem E, et al. Therapeutic response of multidrug-resistant Plasmodium falciparum and P. vivax to chloroquine and sulfadoxine-pyrimethamine in southern Papua, Indonesia. *Trans R Soc Trop Med Hyg* 2007; **101**(4): 351-9.
7. Sumawinata IW, Bernadeta, Leksana B, et al. Very high risk of therapeutic failure with chloroquine for uncomplicated Plasmodium falciparum and P. vivax malaria in Indonesian Papua. *The American journal of tropical medicine and hygiene* 2003; **68**(4): 416-20.
8. Ratcliff A, Siswantoro H, Kenangalem E, et al. Two fixed-dose artemisinin combinations for drug-resistant falciparum and vivax malaria in Papua, Indonesia: an open-label randomised comparison. *Lancet* 2007; **369**(9563): 757-65.
9. Tjitra E, Suprianto S, Anstey NM. Higher gametocyte prevalence following failure of treatment of Plasmodium falciparum malaria with sulfadoxine-pyrimethamine and the combination of chloroquine plus sulfadoxine-pyrimethamine: implications for progression of anti-folate resistance. *Trans R Soc Trop Med Hyg* 2002; **96**(4): 434-7.
10. Baird JK, Basri H, Purnomo, et al. Resistance to chloroquine by Plasmodium vivax in Irian Jaya, Indonesia. *The American journal of tropical medicine and hygiene* 1991; **44**(5): 547-52.
11. Rieckmann KH, Davis DR, Hutton DC. Plasmodium vivax resistance to chloroquine? *Lancet* 1989; **2**(8673): 1183-4.
12. Price RN, Douglas NM, Anstey NM. New developments in Plasmodium vivax malaria: severe disease and the rise of chloroquine resistance. *Curr Opin Infect Dis* 2009; **22**(5): 430-5.
13. Tjitra E, Anstey NM, Sugiarto P, et al. Multidrug-resistant Plasmodium vivax associated with severe and fatal malaria: a prospective study in Papua, Indonesia. *PLoS Med* 2008; **5**(6): e128.
14. Asih PB, Marantina SS, Nababan R, et al. Distribution of Plasmodium vivax pvdhfr and pvdhps alleles and their association with sulfadoxine-pyrimethamine treatment outcomes in Indonesia. *Malaria journal* 2015; **14**: 365.
15. Poespoprodjo JR, Kenangalem E, Wafom J, et al. Therapeutic Response to Dihydroartemisinin-Piperaquine for P. falciparum and P. vivax Nine Years after Its Introduction in Southern Papua, Indonesia. *The American journal of tropical medicine and hygiene* 2018; **98**(3): 677-82.
16. Asih PB, Rogers WO, Susanti AI, et al. Seasonal distribution of anti-malarial drug resistance alleles on the island of Sumba, Indonesia. *Malaria journal* 2009; **8**: 222.
17. Menard D, Khim N, Beghain J, et al. A Worldwide Map of Plasmodium falciparum K13-Propeller Polymorphisms. *N Engl J Med* 2016; **374**(25): 2453-64.
18. Noel-Weiss J, Courant G, Woodend AK. Physiological weight loss in the breastfed neonate: a systematic review. *Open medicine : a peer-reviewed, independent, open-access journal* 2008; **2**(4): e99-e110.

19. Flaherman VJ, Kuzniewicz MW, Li S, Walsh E, McCulloch CE, Newman TB. First-day weight loss predicts eventual weight nadir for breastfeeding newborns. *Archives of disease in childhood Fetal and neonatal edition* 2013; **98**(6): F488-92.
20. Greenwood BM, Greenwood AM, Snow RW, Byass P, Bennett S, Hatib-N'Jie AB. The effects of malaria chemoprophylaxis given by traditional birth attendants on the course and outcome of pregnancy. *Trans R Soc Trop Med Hyg* 1989; **83**(5): 589-94.
21. D'Alessandro U, Langerock P, Bennett S, Francis N, Cham K, Greenwood BM. The impact of a national impregnated bed net programme on the outcome of pregnancy in primigravidae in The Gambia. *Trans R Soc Trop Med Hyg* 1996; **90**(5): 487-92.
22. Villar J, Cheikh Ismail L, Victora CG, et al. International standards for newborn weight, length, and head circumference by gestational age and sex: the Newborn Cross-Sectional Study of the INTERGROWTH-21st Project. *Lancet* 2014; **384**(9946): 857-68.
23. World Health Organisation, Foundation for Innovative New Diagnostics, US Centers for Disease Control and Prevention. Malaria Rapid Diagnostic Test Performance: Summary results of WHO product testing of malaria RDTs: rounds 1-6 (2008–2015). Geneva: World Health Organisation, 2015.
24. Ismail MR, Ordi J, Menendez C, et al. Placental pathology in malaria: a histological, immunohistochemical, and quantitative study. *Hum Pathol* 2000; **31**(1): 85-93.
25. Polley SD, Gonzalez IJ, Mohamed D, et al. Clinical evaluation of a loop-mediated amplification kit for diagnosis of imported malaria. *The Journal of infectious diseases* 2013; **208**(4): 637-44.
26. Hopkins H, Gonzalez IJ, Polley SD, et al. Highly sensitive detection of malaria parasitemia in a malaria-endemic setting: performance of a new loop-mediated isothermal amplification kit in a remote clinic in Uganda. *The Journal of infectious diseases* 2013; **208**(4): 645-52.
27. Kamau E, Alemayehu S Fau - Feghali KC, Feghali Kc Fau - Saunders D, Saunders D Fau - Ockenhouse CF, Ockenhouse CF. Multiplex qPCR for detection and absolute quantification of malaria. (1932-6203 (Electronic)).
28. Shokoples SE, Ndao M Fau - Kowalewska-Grochowska K, Kowalewska-Grochowska K Fau - Yanow SK, Yanow SK. Multiplexed real-time PCR assay for discrimination of Plasmodium species with improved sensitivity for mixed infections. (1098-660X (Electronic)).
29. Singh B, Bobogare A Fau - Cox-Singh J, Cox-Singh J Fau - Snounou G, Snounou G Fau - Abdullah MS, Abdullah Ms Fau - Rahman HA, Rahman HA. A genus- and species-specific nested polymerase chain reaction malaria detection assay for epidemiologic studies. (0002-9637 (Print)).
30. Desai M, Gutman J, L'Lanziva A, et al. Intermittent screening and treatment or intermittent preventive treatment with dihydroartemisinin-piperaquine versus intermittent preventive treatment with sulfadoxine-pyrimethamine for the control of malaria during pregnancy in western Kenya: an open-label, three-group, randomised controlled superiority trial. *Lancet* 2015; **386**(10012): 2507-19.
31. Madanitsa M, Kalilani L, Mwapasa V, et al. Scheduled Intermittent Screening with Rapid Diagnostic Tests and Treatment with Dihydroartemisinin-Piperaquine versus Intermittent Preventive Therapy with Sulfadoxine-Pyrimethamine for Malaria in Pregnancy in Malawi: An Open-Label Randomized Controlled Trial. *PLoS Med* 2016; **13**(9): e1002124.
32. Rijken MJ, McGready R, Phyo AP, et al. Pharmacokinetics of dihydroartemisinin and piperaquine in pregnant and nonpregnant women with uncomplicated falciparum malaria. *Antimicrobial agents and chemotherapy* 2011; **55**(12): 5500-6.
33. Hoyt J, Landuwulang CUR, Ansariadi, et al. Intermittent screening and treatment or intermittent preventive treatment compared to current policy of single screening and treatment for the prevention of malaria in pregnancy in Eastern Indonesia: acceptability among health providers and pregnant women. *Malaria journal* 2018; **17**(1): 341.
34. Hill J, Landuwulang CUR, Ansariadi, et al. Evaluation of the national policy of single screening and treatment for the prevention of malaria in pregnancy in two districts in Eastern Indonesia: health provider perceptions. *Malaria journal* 2018; **17**(1): 309.

35. Webster J, Ansariadi, Burdam FH, et al. Evaluation of the implementation of single screening and treatment for the control of malaria in pregnancy in Eastern Indonesia: a systems effectiveness analysis. *Malaria journal* 2018; **17**(1): 310.
36. Hayes RJ, Moulton LH. Cluster Randomised Trials, 2nd Edition: Chapman and Hall, CRC Press, Taylor and Francis group; 2017.
37. Altman DG, Bland JM. Interaction revisited: the difference between two estimates. *Bmj* 2003; **326**(7382): 219.

STATISTICAL ANALYSIS PLAN

Trial title: Intermittent Screening and Treatment (IST) or Intermittent Preventive Therapy (IPT) for the Control of Malaria in Pregnancy in Indonesia: an open label cluster-randomised ndomized controlled superiority trial.
Short Title: STOPMIP Indonesia

**Study Identifiers:**			
LSTM REC 12.28	Eijkman Institute REC: 57<br>Indonesian MoH NIHRD: LB.02.01/5.2/KE.059/2-13	Trial registration: ISRCTN: 34010937	Protocol Version v3.0-18Jun15

Chief Investigator: Prof Feiko ter Kuile, LSTM (Feiko.terkuile@lstmed.ac.uk)
Co-Principal Investigator: Dr. Rukhsana Ahmed, LSTM (Rukhsana.ahmed@lstmed.ac.uk); Dr Din Syafruddin, EI (din@eijkman,go.id)
Trial statistician: Prof Brian Faragher, LSTM (Brian.Faragher@lstmed.ac.uk); other supporting trial statisticians: Dr Tao Chen, LSTM (Tao.Chen@lstmed.ac.uk), Prof Duolao Wang, LSTM (Duolao.Wang@lstmed.ac.uk)
Co-Investigators: Jeanne Poespoprodjo, Puji Asih, Rintis Noviyanti, Jenny Hill, Jayne Webster, Prof Richard Price
Trial statistician: Prof Brian Faragher, LSTM ()
Pharmocovigilance Officer: Cheryl Pace, LSTM (pv@lstmed.ac.uk)
Data Team: James Smedley (lead); Theda Lukito (site Data Manager)
SAP authors: Rukhsana Ahmed, Carole Khairallah; Brian Faragher; Feiko ter Kuile

Funder: MRC/DFID/Wellcome Trust Joint Global Health Trial Scheme (JGHT 165)

Sponsor: Liverpool School of Tropical Medicine (LSTM)

**SAP version History:**			
Version Date	SAP version #	Details of Changes	Signature CI
24 Mar 2017	1.1	Original	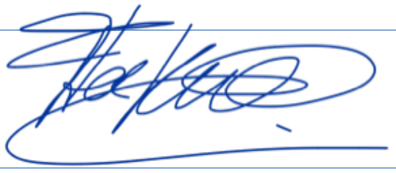

Confidentiality Statement: This document contains confidential information that must not be disclosed to anyone other than the sponsor, the investigator team, host institution, relevant ethics committees and regulatory authorities

Signatures

	Signature	Date
Prof Brian Faragher<br>(Trial Statistician)		03 April 2017
Prof Feiko ter Kuile<br>(Chief Investigator)		24 March 2017
Prof Tim Peto<br>(Statistician: DMEC)		02 April 2017

………………………………………………………………………………………………………

Table of Contents

1. List of abbreviations 5
2. Introduction 6
2.1. Overview design 6
2.2. Trial objectives: 6
2.3. Sample size 6
2.3.1. Initial sample size 6
2.3.2. New sample size (protocol v 3.0) 7
2.4. Randomisation and allocation 7
3. Purpose of the analysis plan 8
4. Definitions 8
4.1. Malaria infection endpoint definitions 8
4.1.1. Booking visit 8
4.1.2. Antenatal (during pregnancy, after enrolment, before delivery) 8
4.1.3. Delivery 9
4.1.4. Infant 9
4.1.5. Other malaria definitions 10
4.1.6. Molecular definition of malaria infection detected by LAMP and PCR data 10
4.2. Morbidity endpoint definitions 11
4.3. Definitions for other Endpoints 13
4.4. Definitions for other variables 14
5. Study Outcomes 15
5.1. Primary outcome 15
5.2. Secondary efficacy outcomes 15
5.2.1. Antenatal (from 1 day after enrolment to 1 day prior to delivery) 15
5.2.2. At delivery mother 16
5.2.3. At delivery newborn 16
5.2.4. After delivery infant 17
5.3. Safety outcomes 17
5.4. Tolerability and compliance outcomes 17
6. Analytical population 17
6.1. Efficacy 17
6.1.1. Intention to treat analysis (ITT) 17
6.1.2. Per protocol Population (PP); 18
6.2. Safety Population 18
7. General analytical approach 18
7.1. Reporting guidelines 18
7.2. Data Pooling and standalone estimates 18
7.3. Pre-scheduled stopping of study participants and use of data 19
7.4. Missing data 19
7.4.1. Endpoints 19
7.4.2. Covariates 19
7.5. Linear regression analysis for adjusted analysis of dichotomous outcomes 19
7.5.1. Risk ratios or odds ratios? 19
7.5.2. Log binomial regression and alternative strategies in case of non-convergence 20
7.6. Reporting conventions 20
7.6.1. Descriptive statistics 20

7.6.2. Measures of associations and P-value reporting 20
8. Participant disposition and Flow chart 20
9. Baseline data summaries 21
9.1. Demographic, clinical and laboratory measures 21
9.2. Measures of Social Economic Status (SES) Asset index. 21
10. Efficacy analyses 21
10.1. Measure of associations 21
10.1.1. Binary outcomes 21
10.1.2. Continuous outcomes 21
10.2. Forest plots of efficacy parameters 22
10.3. Primary efficacy endpoint analysis 22
10.3.1. Crude and adjusted effect estimates 22
10.3.2. Adjustment for baseline independent variables in the multiple regression models 22
10.3.3. Variable specification 22
10.4. Secondary efficacy analysis of primary outcomes 23
10.4.1. Subgroup analysis of the primary outcome 23
10.4.2. Sensitivity analysis 23
10.4.3. Effect stratified by gravidity 23
10.5. Secondary efficacy outcomes 24
10.5.1. Outcomes at delivery 24
10.5.2. Count data outcomes 24
10.5.3. Continuous outcomes 24
11. Safety analysis 24
11.1. Further Adverse Events analysis. 24
12. Other analysis 25
12.1. Number of intervention visits 25
12.2. Compliance with study drug 25
12.3. Treatment response 25
13. References 25
14. Tables and Figures 26
14.1. List of tables 26
14.2. Patient disposition (Flow chart) 28
15. Versioning manuscript 29
15.1. Word document 29
15.2. Endnote 29
16. Appendices 30
16.1. Appendix 1: CONSORT 2010 checklist of information to include when reporting a cluster randomised trial 30

1. List of abbreviations

AE	Adverse event
AQ-AS	Artesunate-amodiaquine
CRF	Case record form
DHP	Dihydroartemisinin-piperaquine
FGD	Focus group discussion
Hb	Haemoglobin
HIV	Human immunodeficiency virus
HRP-2	Histidine-rich protein two
IPTp	Intermittent preventive therapy in pregnancy
ISTp	Intermittent screening and treatment in pregnancy
ISTp-DHP	Intermittent screening and treatment in pregnancy, screening and treating malaria cases with dihydroartemisinin-piperaquine
ITN	Insecticide treated (bed) net
LMP	Last menstrual period
LAMP	Loop-mediated isothermal amplification
LSTM	Liverpool School of Tropical Medicine
MiP	Malaria in Pregnancy
PI	Principal Investigator
PCR	Polymerase chain reaction
P. falciparum	*Plasmodium falciparum*
PfEMP1	*P. falciparum* erythrocyte membrane protein 1
pLDH	Plasmodium lactate dehydrogenase
RDT	Rapid diagnostic test (for malaria)
SAE	Serious adverse event
SSTp	Single screening and treatment in pregnancy
SUSAR	Suspected unexpected adverse reaction
VSA	Variant surface antigens
WHO	World Health Organization

2. Introduction

2.1. Overview design

This is an open-label three-arm parallel-group matched cluster-randomised controlled superiority trial conducted in two rural sites in Eastern Indonesia with low levels of *P.falciparum* and *P.vivax* malaria comparing the efficacy, safety and cost-effectiveness of intermittent screening and treatment (ISTp) and intermittent preventive therapy (IPTp) with the current single screening and treatment (SSTp) strategy. Dihydroartemisinin-piperaquine (DHP) is used in all three arms. The trial is designed to detect 50% reduction in any malaria infection at delivery (peripheral or placental, any species, detected by microscopy, RDT, histology [acute/chronic] or PCR/LAMP) from 10% to 5% in women at delivery. The unit of randomization is antenatal-clinics. The initial study design (protocol v2.0) required 26 clusters of 41 women per cluster/arm for an overall sample size of 3198 women. The revised sample size requires a total of 2279 women from 26 clusters per arm; 989 women in Sumba (57 clusters) and 1290 in Timika (21 clusters). It is open label because it will not be possible to blind the participants to their allocation, although laboratory staff undertaking trial related diagnostic tests will be blinded. Health service related studies are conducted to assess the acceptability of the 3 interventions and the feasibility of screening policies. This study will collect data which will allow an analysis of the cost-effectiveness of the three different strategies proposed.

2.2. Trial objectives:

1. To compare the efficacy if IPTp -DHP or ISTp-DHP with RDTs in the 2nd and 3rd trimester with the current strategy of SSTp-DHP to reduce the risk of any malaria infection at delivery among women protected by long lasting insecticide treated nets (LLINs) in areas with relatively low *P.falciparum* and *P.vivax* transmission in eastern Indonesia.
2. To estimate the acceptability, feasibility and cost effectiveness of each of SSTp-DHP, ISTp-DHP and IPTp-DHP within the randomised control trial.

2.3. Sample size

2.3.1. *Initial sample size*

The unit of randomisation is the clinic providing antenatal care (Puskesmas and Posyandu). The average number of women per clinic for the study period was estimated to be 58 (i.e. 29 pregnancies per year). We estimate that 41 of the 58 (70%) women would fulfil the eligibility criteria and provide informed consent, and that 80% of them (33) would contribute to the primary endpoint. The remaining 20% will be lost or have incomplete delivery data. The study was designed to detect at least a 50% reduction in malaria infection at delivery, from 10.0% in the SSTp group (pooled prevalence, Sumba and Papua, 2009) to 5.0%. In the previous surveys in Sumba and Papua, the Intracluster Coefficient (ICC) for the primary endpoint was 0.002 (data from 50 clusters). The number of clusters needed per arm was 23, based on 41 women per cluster resulting in 33 completed deliveries, 90% power, 2-sided alpha of 0.025, and an assumed ICC of 0.002. To adjust for 13% efficiency loss due to varying cluster sizes, 26 clusters will be included (i.e. 26x41=1,066 women per arm, and 3198 overall). The same sample size would have 80% power to detect 50% difference if the prevalence of malaria was only 7.5%, or if the ICC was 0.013.

2.3.2. *New sample size (protocol v 3.0)*

The trial in Sumba site was ended after recruitment of 989 women. New sample size calculations were conducted to determine the sample required for completion of the trial using recruitment in Timika site only. Power calculations were performed using Stata Metapower to estimate the sample size required for the trial to achieve at least 80% power using 10,000 simulations with the data from Sumba treated as 1 trial with 989 women, and data from Timika collected till January 2015 as another trial. The Timika extension was simulated as a new trial. Blinded data pooled across the 3 arms for each site was used to obtain observed estimates of the pooled frequency of the primary endpoint and ICC value. The prevalence of the primary endpoints used in the new sample size calculation were 4.1% in Sumba and 24% in Timika. The observed ICC value was 0.0005. All analysis was conducted blinded. No interim analysis of the effect size was conducted.

A total of 2279 women; 989 women in Sumba (57 clusters) and 1290 in Timika (24 clusters) would achieve approximately 81% power to detect a 50% reduction in malaria infection from approximately 15% in the control arm to 7.5% in any of the intervention arms, using an alpha of 0.0167 to allow for 3 comparisons (compared to 0.025 in the original study which allowed for only 2 comparisons), and allowing for 13% efficiency loss due to varying cluster size and 20% loss to follow-up up. The new sample size would also have 87% power to detect a 50% reduction in Timika alone if the average prevalence of infection in the control arm is at least 24%.

2.4. Randomisation and allocation

The ANC clinics constituted the units of randomisation. A 1:1:1 allocation ratio was used. To minimize imbalances across treatment groups with respect to baseline malaria prevalence and risk factors for malaria, multivariate matching was used, based on malaria indicators available, such as the prevalence of positive RDTs or microscopy at antenatal visit in the 12-month period prior to the trial (ANC registry data), geographical area and clinic size (prior annual number of new ANC attendees); in this way, the 78 eligible clinics was blocked into 26 sets of 3 matched clusters.

The trial statistician at LSTM computer-generated lists of sets of triple-matched clusters and forwarded these to the trial site in Indonesia. The allocation of clusters to each of the three study arms were done as a public event. District Health Officials and village elders were asked to draw opaque sealed envelope from a box. Each sealed envelope contained the allocation, and after drawing the envelopes, they were opened and allocation recorded and study arm assigned. Signed envelopes containing the final list of clinic names and their allocation were sent to the trial statistician and a copy kept in the trial site in the TMF.

Minimization of selection and confounding bias is achieved through central block randomisation taking baseline data on malaria risk into account. The matched design with clinics as the unit of randomisation will minimize contamination between individual women and avoid allocation errors. The endpoints (malaria infection, birth weight, etc.) are measured at delivery, most of which take place in the health facility. The primary outcome, malaria infection is an objective verifiable measure, performed by laboratory staff unaware of the randomisation allocation.

3. Purpose of the analysis plan

The purpose of this document is to outline the statistical analysis plan for the STOPMiP trial. The primary objective of this trial was to compare the effectiveness in reducing malaria infection at delivery of IPTp-DHP or ISTp-DHP with RDTs in the second and third trimesters against the strategy of SSTp-DHP. The target study population was women protected by long lasting insecticide treated nets (LLIN) in areas with relatively low to moderate transmission of *P.falciparum* and *P.vivax* in Indonesia.

The SAP is based on the version of the amended protocol (V3.0 18June15) and approved by the Research Ethics Committees of the Liverpool School of Tropical Medicine (Sponsor) and the Eijkman Institute (collaborating institute and primary ethics committee in Indonesia). One interim analysis was planned half-way (when delivery numbers reached 50% of initial sample size) for assessing whether to stop the trial early (or one of the study arms) due to safety, efficacy or futility. With ending of recruitment in Sumba site before 50% deliveries of the total sample size was reached and recalculation of sample size for Timika site with single site option, the interim analysis stated in the protocol version 2.0 was dropped. A secondary analysis to compare the two interventions ISTp and IPTp was added. As the study design uses matched cluster randomisation methodology, all analyses will use GEE multi-level random effect log-binomial for binary and Poisson or negative binomial models for count data (primary outcome, incidence rates, event occurrences), with adjustment both for intra-cluster correlation at the level of randomisation (clinic) and for important covariates. Although the primary purpose of the matching in the randomisation is to optimise the balance between the study groups, covariate adjustment will be made for matching variables where appropriate (provided perfect matching was not achieved).

4. Definitions

4.1. Malaria infection endpoint definitions

For all the definitions of malaria infection at delivery reference is made to any species of *Plasmodium* (*falciparum*, *vivax*, *malariae*, *ovale*, *knowelsi*, etc.).

4.1.1. *Booking visit*

1. Booking visit maternal malaria infection: infection detected in peripheral blood at the first antenatal visit, (yes/no)
    a. Standard microscopy
    b. PCR/LAMP
    c. Microscopy and PCR/LAMP
    d. RDT with fever/ history of fever
    - Excludes RDTs with no fever as this was a routine part of the SSTp and ISTp intervention but not for IPTp.

4.1.2. *Antenatal (during pregnancy, after enrolment, before delivery)*

2. Antenatal maternal malaria, (yes/no): any plasmodium detected in the peripheral blood of the mother prior to time of delivery by either
    a. Standard microscopy at all scheduled antenatal visits or unscheduled visits
    b. PCR/LAMP at all scheduled antenatal visits or unscheduled visits

c. RDT at only unscheduled visits or at a scheduled visit with fever/ history of fever
- Excludes all RDTs at scheduled visits and scheduled visits with no fever as this was a routine part of the SSTp and ISTp intervention but not for IPTp.

3. Antenatal 3rd trimester peripheral malaria infection mother at last scheduled visit in the 3rd trimester , (yes/no)
   a. Antenatal peripheral malaria infection mother detected during the last scheduled antenatal visit in the 3rd trimester, before delivery.
   b. Otherwise the same diagnostic criteria are used as for antenatal peripheral malaria infection mother, described for antenatal malaria above

4.1.3. *Delivery*

4. Maternal malaria (at delivery); (yes/no) any species of plasmodium detected in maternal peripheral blood either by
   a. RDT (pLDH or HRP2 or both bands)
   b. peripheral blood smear microscopy
   c. PCR/LAMP
   - This includes RDT as this was taken routinely in all study arms
5. Placental malaria infection (any), (yes/no): Any *Plasmodium* species detected in the placental blood or biopsy tissue by either
   a. Placental incision smear microscopy (standard microscopy)
   b. RDT (pLDH or HRP2 or both)
   c. Placental malaria by histology (active or past infection)
   d. PCR/LAMP
6. Placental malaria by histology (active), (yes/no)
   a. Active infection (acute or chronic)
   - Note past infections or no infections will be considered negative
   - Considers maternal placental blood only and includes active infections only
7. Placental malaria by histology (any), (yes/no); either
   a. Past infection (pigment in fibrin detected in the absence of asexual parasites)
   b. Active infection (acute or chronic) (asexual parasite present)
   - Considers maternal placental blood only

4.1.4. *Infant*

8. Cord blood malaria, (yes/no): any plasmodium species detected in cord blood the new born at birth by either
   a. RDT (pLDH or HRP2 or both)
   b. Standard smear microscopy
   c. PCR/LAMP
9. Congenital malaria, (yes/no): any plasmodium species detected in cord blood or peripheral blood of the new born at birth or within 7 days (168 hours) after birth, by either
   a. RDT (pLDH or HRP2 or both)
   b. Standard microscopy
   c. PCR/LAMP

10. Infant malaria, (count): Fever, or any other symptom that triggered the diagnostic tests for malaria plus any plasmodium species detected between days 1 and end of infant follow-up (week-6-8 postnatal) detected by either.
    a. RDT (pLDH or HRP2 or both)
    b. Standard smear microscopy
    c. PCR/LAMP

4.1.5. *Other malaria definitions*

11. Sub-patent maternal malaria (yes/no)
    a. PCR/LAMP positive, and
    b. Microscopy and RDT negative; or either test negative if the one test is missing (exclude missing results)
    c. Note; cannot be defined if both microscopy and RDT results are missing.
12. Patent peripheral malaria infection, (yes/no)
    a. Microscopy and/or RDT positive, and
    b. PCR/LAMP positive
    - Note; cannot be defined if both microscopy and RDT results are missing; can be defined if PCR/LAMP results are missing.
13. Sub-patent placental malaria infection, (yes/no)
    a. PCR/LAMP positive and/ or Placental histology active infection positive and
    b. Microscopy and RDT negative **(**or one of the two if the other is missing)
    - Note: Cannot be defined if microscopy and RDT results are missing;
14. Patent placental malaria infection, (yes/no)
    a. Microscopy and/or RDT positive and
    b. PCR/LAMP and/ or placental histology active infection
    - Note; Cannot be defined if both microscopy and RDT results are missing;
    - Note: Can be defined if either or both PCR/LAMP and histology are missing
15. Clinical malaria, (yes/no)
    a. Documented fever (>=37.5°C), or recent history of fever in the past 48 hours, or other symptoms of acute illness that resulted in a women seeking care or alerting the study team to request a home visit, and
    b. Maternal malaria patent infection detectable by Microscopy or RDT
    - Excludes immediate follow-up visits related to the primary episode; if not defined, use 14 days exclusion period for that endpoint
16. Asexual parasite density by microscopy
    a. Parasite density expressed per mm3, quantified against 300 leucocytes on assumed white blood cell count of 8000/mm3.
    b. The parasite density is defined by natural log transformation of the above count

4.1.6. *Molecular definition of malaria infection detected by LAMP and PCR data*

The study involves quantitative and nested PCR confirmation of all LAMP positive samples and a random sample of 5% of the LAMP negatives. The following algorithm will be used to define LAMP/PCR positivity (see Figure 1 Definition of PCR/LAMP positivity):

Figure 1 Definition of PCR/LAMP positivity

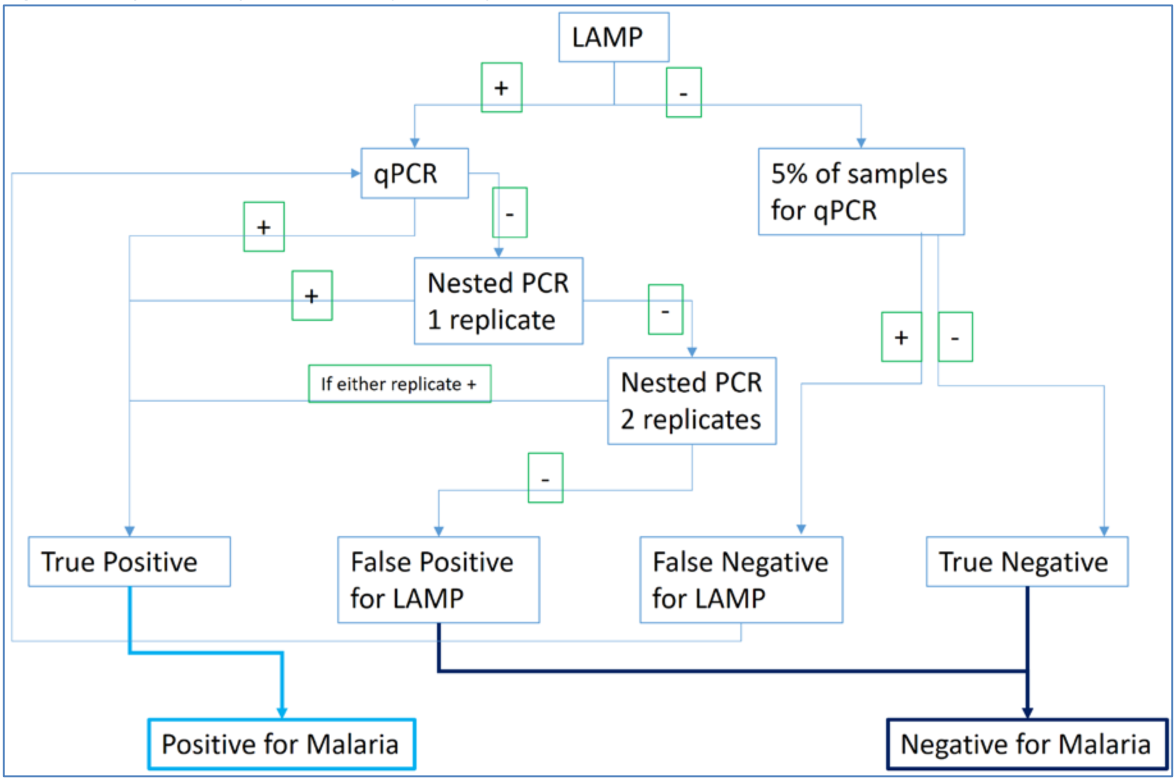

4.2. Morbidity endpoint definitions

1. Birthweight
    a. Uncorrected birthweight (grams) (continuous) weight taken within 24 hours of birth using digital scales (precision +/-10grams) in live singleton babies. Birthweights taken more than 24 hours after delivery will not be considered because of the physiological fall in birth weight in breastfed infants occurring in the first days following delivery.[1, 2]
    b. Corrected birthweight (grams) (continuous); weight taken within 7 days (168 hours) after birth in live singleton babies. Birthweights taken more than 24 hours after delivery will be corrected for the physiological fall in birth weight in breastfed infants occurring in the first days following delivery.[1, 2]
        i. Birth weights taken 24-48h hours, and 48-168 hours after delivery will be corrected by a factor +2% and +4%, respectively to obtain the estimated weight at birth.[3, 4]
        ii. Birth weights within 24 hours will not need to be corrected.
2. Newborn Gestational age (days) (continuous): derived gestational age at booking in days based on gestational age assessment methods at booking assessed in order of priority as follows:
    a. By gestational age from Ballard score estimated within 96 hours of delivery
    b. By Last Menstrual Period if known and if Ballard examination is not available
    c. By fundal height measurement if no other measure of gestational age is available.

3. Maternal Gestational age (days) (continuous): Gestational age at booking (or any other visit) is the newborn gestational age in days, minus the time in days between the date of delivery and date of enrolment/or visit.
4. Gestational trimester
    a. 2nd trimester: Gestational age from 112- 195 days (14-27 weeks) inclusive
    b. 3rd trimester: Gestational age from 196 days (28 weeks) onwards
5. Birthweight (corrected) for gestational age percentile (WgAP) or Z-scores (WgAZ) (continuous variable)
    a. Gender specific reference INTERGROWTH-21st Newborn birth weight standards and Z scores will be used to calculate percentile or a Z score for Birth weight-for-gestational age.[5]
6. Low-birth-weight (LBW), (yes/no): corrected birth weight under 2,500 grams
7. Low-birth-weight (LBW-uncorr), (yes/no): uncorrected birth weight under 2,500 grams
8. Preterm birth (PTB), (yes/no): spontaneous birth before 259 days (37 weeks) gestation
9. Small-for-Gestational Age (SGA), (yes/no): foetal weight <10th percentile of gestation age (see birth weight for gestational age for reference population)
10. Miscarriage, (yes/no): Loss of foetus before 196 days (28 weeks) gestation (inclusive).
11. Induced abortions, (yes/no): Intentional loss of a foetus before 196 days (28 weeks) gestation.
12. Still birth, (yes/no): Loss of foetus >= 196 days (28 weeks) gestation or later showing no signs of life.
13. Foetal loss, (yes/no): Stillbirth or miscarriage
14. Adverse live-birth outcome, (yes/no): composite endpoint defined as having a birth that fulfils the criteria for either:
    a. LBW or
    b. Preterm birth or
    c. SGA
15. Adverse any-birth outcome, (yes/no): composite endpoint defined as having a birth that fulfils the criteria for either:
    a. LBW
    b. Preterm birth
    c. SGA
    d. Still birth
    e. (Spontaneous) miscarriage
16. Perinatal death, (yes/no): still birth **or** death within 7 days of birth
17. Neonatal death, (yes/no): death within 28 days of birth (defined as ‘1’ month or earlier for pragmatic reasons).
18. Early Infant mortality (yes/no): death from birth to end of follow-up (about 6 to 8 weeks after birth).
19. Maternal death, (yes/no): The death of a woman while pregnant or within 42 days of termination of pregnancy
20. Non-malaria sick-clinic visits, maternal (count)
    a. Documented fever (>=37.5 ^{o}C), or recent history of fever in the last 48 hours, or other symptoms of acute illness that resulted in a woman seeking care or alerting the study team to request a home visit
    b. No evidence of peripheral malaria infection by RDT or microscopy

- Excludes immediate follow-up visits related to the primary episode; if not defined, use 14 days' exclusion period for that endpoint
- Note: these events are mutually exclusive of clinical malaria (i.e. all-cause sick-clinic visits minus sick clinic visits due to clinical malaria = non-malaria sick-clinic visits)
- Delivery visits will be ignored in the clinical visits analysis hence ignoring the placental information

21. Non-malaria sick-clinic visits, infant (count) (same as for maternal) resulting in seeking care for an infant)
22. All-cause sick-clinic visits, maternal (count)
    - The sum of Clinical malaria and non-malaria sick-clinic visits with fever or history of fever in last 48 hours
    - Excludes immediate follow-up visits related to the primary episode; if not defined, use 14 days' exclusion period for that endpoint
23. All-cause sick-clinic visits, infant (count)
    - The sum of Clinical malaria and non-malaria sick-clinic visits
    - Excludes immediate follow-up visits related to the primary episode; if not defined, use 14 days' exclusion period for that endpoint
24. Maternal anaemia, (yes/no): Hb<11.0 g/dL (measured by HemoCue (Angelhom, Sweden), either venous or capillary blood).
25. Maternal Moderate to severe anaemia, (yes/no): Hb<9.0 g/dL (measured on Hemocue, either venous or capillary blood) (used to provide adequate power as Hb< 8 g/dL is rare, and to be in the midpoint between any anaemia (above) and severe anaemia (below)
26. Maternal severe anaemia, (yes/no): Hb<7.0 g/dL
27. Fetal anaemia, (yes/no): Hb<12,5 g/dL in umbilical cord blood at birth, which is 2 standard deviations below the mean cord Hb in developed countries[6]
28. Congenital malformations, (yes/no): Physical abnormality of live born baby detected at delivery or newly noted abnormality during the infant visits (7 days or 6-8 weeks post-natal).
29. Neonatal jaundice, (yes/no): Reported presence of jaundice in neonate within first seven days of life.

4.3. Definitions for other Endpoints

1. Treatment Compliance with IPTp-DHP (yes/no):
    a. With each course: took all tablets on each of the 3 daily doses of DHP
    b. With the overall regimen: Attended all scheduled visits until delivery [exclude visits that could not have occurred because the woman delivered before that scheduled visit date]
2. Treatment compliance with SSTp or ISTp (yes/no):
    a. With each course: took all tablets on each of the 3 daily doses of DHP
    b. With the overall regimen: Attended all scheduled visits until delivery and took all doses as required [exclude visits that could not have occurred because the woman delivered before that scheduled visit date].
3. Approved alternate treatment: Receipt of either quinine or clindamycin or DHP (national programme) for treatment of symptomatic malaria at a scheduled or unscheduled visit

4. Treatment compliance (quintiles): Treatment compliance will be defined as a percentage (total number of tablets taken/total number of tablets expected)*100, and divided into 3 equal groups (tertiles).
5. Treatment compliance (continuous): Treatment compliance will be defined as a percentage (total number of tablets taken/total number of tablets expected)*100, and treated as a continuous variable.
6. Regimen compliance will be defined as a percentage of the number of scheduled visits attended (total number of scheduled visits attended/total number of scheduled visits expected by gestational age at enrolment and delivery)*100, and then ranked into 5 equal groups (quintiles).
    a. Exclude visits that could not have occurred because the woman delivered before that scheduled visit date.
7. DHP Day-1 dose intolerance (%): Vomited DHP on day-1 and did not tolerate a repeat dose (vomited again) or was not given a repeat dose (where day-1 is the first dose day).
8. DHP Day-2 dose intolerance (%): Vomited DHP on day-2 and did not tolerate a repeat dose (vomited again) or was not given a repeat dose.
9. DHP Day-3 dose intolerance (%): Vomited DHP on day-3 and did not tolerate a repeat dose (vomited again) or was not given a repeat dose.
10. DHP course intolerance (%): DHP Day-1, or Day-2 or Day-3 dose intolerance.
11. Day-1 regimen intolerance risk (%):DHP Day-1 dose intolerance at least once.
12. DHP regimen intolerance (%): DHP course intolerance at least once

4.4. Definitions for other variables

1. Season (tertiles): Each pregnancy will be defined to have occurred in the predominantly rainy vs dry season using rainfall data collected in the study area. This will be done by categorising the women into three equal groups based on the mean daily, weekly or monthly rainfall during the 6-month period prior to the date of delivery (i.e. during the 2nd and 3rd trimester of pregnancy). This can include rainfall data prior to her enrolment in the study.
2. Gravidity will be computed and triangulated from the various variables in the enrolment questionnaire and categorised into nominal (not ordinal) categorical variables. The nominal variable will be used because the relationship between gravidity and the primary outcomes is not linear. The following categories will be used:
    a. Gravidity by number, (G1, G2, G3, G4+):
        i. First pregnancy (G1)
        ii. Second pregnancy (G2)
        iii. Third pregnancy (G3)
        iv. Fourth pregnancy G4+
        - Computed based on the combination of variables in the booking form (‘Primi yes/no, gravid, previous livebirths, stillbirths and miscarriages).
    b. Pauci-Gravidae-2 (G1+G2)
        i. first and second pregnancies (G1-G2)
    c. Multigravidae (G3+)
        - Third or more pregnancies

- Computed based on the combination of variables in the booking form ('Primi yes/no, gravid, previous livebirths, stillbirths and miscarriages).

3. Educational status:
    a. no schooling
    b. low (primary and primary no completed)
    c. medium (junior and senior high)
    d. high (academy or university)
    - if data is incomplete, this will be changed into (yes/no) primary school completed, junior high completed, senior high/academy/university completed.
4. Socio-Economic Status (SES), (quintiles): Categories will be based on the combination of ownership of household items, materials used for the floor, roof, walls of house and use of fuel, type of toilets and drinking water source in the socioeconomic CRFs and ranked according to World Bank wealth index score.
5. Study site: (Sumba, Timika)
6. Study clusters: will be based on the ANC of enrolment in each study site and not the place of delivery.
7. Place of residence (Urban, rural, not-known): will be based on information provided in the enrolment forms.
8. Place of delivery: is categorised to hospital, Puskesmas, home, private clinic and others (Pustu or Polindes or on the road/vehicle)
9. ITN use at enrolment: binary (yes/no): a single variable which takes into account the responses to this question in booking visit CRF
10. ITN use during pregnancy: binary (yes /no); a single variable which takes into account the responses to the question at scheduled visits, such as if a woman answers less than 50% of the time during pregnancy that she slept under a bednet the previous night than she is considered as a "non-user" vs. a "user" who slept under a bednet more or equal 50% of the time during pregnancy.
11. Beetlenut use: will be categorised into low, moderate or high by tertiles
12. Cigarette smoking: will be categorised to low, moderate or high using tertiles

5. Study Outcomes

5.1. Primary outcome

The primary endpoint will be the presence or absence of malarial infection (any species) at delivery (yes/no) and a composite of either

1. Placental malaria (placental blood or tissue) by microscopy or RDT or histology (acute/chronic) or PCR/LAMP, **or**
2. Maternal malaria (maternal blood) by microscopy or RDT or PCR/LAMP

5.2. Secondary efficacy outcomes

5.2.1. *Antenatal (from 1 day after enrolment to 1 day prior to delivery)*

1. Maternal malaria by PCR/LAMP (count)

2. Maternal malaria by RDT (ISTP arm only) (count)
3. Maternal malaria by microscopy (count)
4. Maternal malaria by any test (see antenatal maternal malaria under definitions) (count)
5. Clinical malaria (count)
3. Non-malaria sick-clinic visits (count)
4. All-cause sick-clinic visits (count)
6. Maternal malaria by PCR/LAMP (at least once [yes/no] [cumulative risk])
7. Maternal malaria by RDT (ISTP arm only) (at least once [yes/no] [cumulative risk])
8. Maternal malaria by microscopy (at least once [yes/no] [cumulative risk])
9. Maternal malaria by any test (see antenatal maternal malaria under definitions) (at least once [yes/no] [cumulative risk])
10. Clinical malaria (at least once [yes/no] [cumulative risk])
11. Non-malaria sick-clinic visits (at least once [yes/no] [cumulative risk])
12. All-cause sick-clinic visits (at least once [yes/no] [cumulative risk])

5.2.2. *At delivery mother*

1. Malaria infection-any (yes/no) (primary endpoint plus past infections)
2. Placental malaria-any (any species, any test) (yes/no)
3. Placental malaria-active (any species, any test, histology active only) (yes/no)
4. Placental malaria-species (categorical): no malaria; Pf, Pv, mixed Pf/Pv; other species. PCR/LAMP confirmed if available otherwise microscopy.
5. Placental malaria-histo (any), (categorical-1): None, Past infection, Acute, Chronic
6. Maternal malarial by PCR/LAMP alone (yes/no)
7. Maternal malarial by microscopy alone (yes/no)
8. Maternal malarial by RDT alone (yes/no)
9. Maternal malaria-any (any test) (yes/no)
10. Maternal gametocytaemia (yes/no)
11. Maternal Asexual parasite density (continuous)
12. Maternal haemoglobin (g/dL) (continuous) (exclude sample taken after delivery)
13. Any anaemia (Hb <11.0 g/dL) (yes/no)
14. Moderate severe anaemia (Hb <9.0 g/dL) (yes/no)
15. Severe anaemia (Hb<7.0 g/dL) (yes/no)

5.2.3. *At delivery newborn*

1. LBW (corrected) (yes/no)
2. LBW (uncorrected) (yes/no)
3. Birthweight-corrected (gram), (continuous)
4. Birthweight-uncorrected (gram), (continuous)
5. Preterm birth (yes/no)
6. Gestational age (week), (continuous)
7. SGA (yes/no)
8. Birthweight for gestational age (Zscores) (corrected birthweight), (continuous)
9. Birthweight for gestational age (Zscores) (uncorrected birthweight), (continuous)
10. Birth outcome (categorical); live birth, stillbirth, spontaneous abortion, induced abortion (yes/no)

11. Foetal loss (yes/no)
12. Adverse live-birth (LBW/SGA/PT) (yes/no)
13. Adverse any birth (LBW/SGA/PT/FL) (yes/no)
14. Foetal Hb at delivery (g/dL), (continuous)
15. Foetal anaemia (yes/no)

5.2.4. *After delivery infant*

1. Infant clinical malaria (count)
2. Infant non-malaria sick-clinic visits (count)
3. Infant All-cause sick-clinic visits (count)
4. Infant clinical malaria (at least once [yes/no] [cumulative risk])
5. Infant non-malaria sick-clinic visits (at least once [yes/no] [cumulative risk])
6. Infant All-cause sick-clinic visits (at least once [yes/no] [cumulative risk])
7. Perinatal mortality (yes/no)
8. Neonatal mortality (yes/no)
9. Infant mortality by end of follow-up (about 6 to 8 weeks after birth) (yes/no)

5.3. Safety outcomes

1. Maternal serious adverse events (SAEs) during pregnancy (count),
    a. Overall
    b. by system organ class (SOC) and preferred term
2. Maternal non-serious adverse events (SAEs) during pregnancy (count)
3. Maternal deaths (yes/no)
4. Infant SAEs by end of follow-up period (count)
    c. Overall
    d. by system organ class and preferred term
5. Infant non-serious adverse events (SAEs) during follow-up (count)
6. Congenital malformations (yes/no) detected at from birth to end of follow-up at 6-8 weeks

5.4. Tolerability and compliance outcomes

1. Compliance with each study course (see definitions)
2. Compliance with overall intervention regimen (see definitions)
3. DHP course intolerance (count).
4. DHP regimen intolerance (yes/no) (DHP course intolerance at least once)

6. Analytical population

6.1. Efficacy

6.1.1. *Intention to treat analysis (ITT)*

The unit of analysis will be individual women (participants). The primary analyses will be based on the ITT principle, so will include all randomised women not considered screening failures and for whom there is an outcome.

6.1.2. *Per protocol Population (PP);*

Per protocol population will be defined as:

1. All women not considered screening failure and received either:
    a. The study intervention and took all of the study doses on each occasion when measured; or
    b. An approved alternative treatment for symptomatic malaria according to protocol that replaced the need for the scheduled intervention; or
    c. Received the potential number of scheduled visits prior to delivery
    - Note: 'potential' visits implies that visits that were scheduled to occur after the observed delivery date are not considered as missing visits (i.e. a woman enrolled at 20 weeks and who came again at 24 and 28 weeks, but delivered at 30 weeks will fulfil the criteria of per protocol even though she will have missed the 32 and 36 weeks visits).

    AND
2. Women who contributed information to the specific endpoint investigated

Women will be excluded from the per protocol population if they used prohibited medication.

6.2. Safety Population

All women who received at least one dose of study drug Eurartesim in IPTp arm or in ISTp or SSTp (if malaria-positive), and have completed sufficient follow-up to provide information on potential adverse events, defined as attendance of the next scheduled study visit from the last dose of investigational product received. We would separately account for women who received DHP under the national programme during unscheduled visits.

7. General analytical approach

7.1. Reporting guidelines

We will follow the Consolidated Standards of Reporting Trials (CONSORT) 2010 statement; extension to cluster randomised trials guidelines for reporting of clinical trials (http://www.consort-statement.org/).

7.2. Data Pooling and standalone estimates

Effect estimates will be computed and presented as a single summary pooled estimate for both sites, with appropriate adjustment for site differences, and in addition for each site separately. All effect estimates will take the cluster design into account.

It is anticipated that the prevalence of the primary outcome may be low or even zero in some clusters, which could affect the ability of some analysis methods to converge (see also section 7.5.2 below). Should this happen, a cluster-level analysis will be performed using linear regression with weighting to account for varying cluster size. If this also fails, consideration will be given, as a last resort, to combining proximate clusters with similar geographical and demographic properties within the same

study arm; any such combinations will be done incrementally to ensure that this process is minimised as far as possible.

7.3. Pre-scheduled stopping of study participants and use of data

In case the intervention was stopped before the pre-scheduled end, either by a decision of the study woman herself, or by the study team, and data was collected after stopping the intervention, the information will be included in the full analyses set.

7.4. Missing data

Missing data will be dealt with differently for the primary endpoint and the independent variables as follows.

7.4.1. *Endpoints*

Missing data on the primary and secondary endpoints will not be imputed.

7.4.2. *Covariates*

Missing values for covariates will be imputed for the covariate adjusted analysis of primary endpoint.

If the missing data for all pre-selected covariates is less than 5% of observations, missing values for these covariates will be imputed by means of multiple imputations (10 multiple datasets will be created) using the SAS procedure MI or similar procedures. Missing data will be assumed missing at random (MAR), (probability that an observation is missing can depend on the observed values of the individual, but not on the missing variable values of the individual). Imputations will be done on continuous as well as categorical variables. If categorical variables are created from continuous variables the imputations will be conducted on the continuous variable. We will first investigate the Missing Completely at Random (MCAR) assumption by modelling the probability of missing data on treatment assignment and other independent variables. If any of the independent variables are significant then missing data depends on covariates, a violation of MCAR. Then missing data will be assumed MAR. Results derived from multiple under MAR imputation and complete-cases analysis without multiple imputation will be compared in a sensitivity analyses. Models under Missing Not At Random assumption (selection and pattern mixture) will not be done. Focus will be on MAR assumption and how its violation can be investigated in a sensitivity analysis.

7.5. Linear regression analysis for adjusted analysis of dichotomous outcomes

7.5.1. *Risk ratios or odds ratios?*

Because the study outcome will be common in some strata the odds ratio is not likely to approximate the risk ratio and be further from 1 than the risk ratio (i.e. more extreme). Because risk ratios are easy to interpret and because odds ratios are sometimes misinterpreted as risk ratios, the study will use risk ratios as the measure of relative association for dichotomous outcomes to assist the public health interpretation of the findings.

7.5.2. *Log binomial regression and alternative strategies in case of non-convergence*

The primary linear regression analysis method to obtain risk ratios and corresponding 95% Confidence Intervals (CI) for dichotomous variables will be log binomial regression (PROC GENMOD in SAS, GLM in Stata). A well-known limitation of log-binomial regression is problems with convergence. If a model does not converge with the default syntax for log-binomial regression, we will use generalized linear models (GLM) with a log link or COPY-method. The advantage of COPY-method over 'robust Poisson method' is that it produces correct approximation of maximum likelihood estimates (MLE) and can be run using the existing PROC GENMOD procedure that had failed on the original data. There is a COPY-method SAS MACRO for executing the method and will be used if there is a need to use COPY-Method. The MACRO first runs the PROC GENMOD procedure on the original dataset. If no convergence occurs, it automatically switches to the COPY-method and MLE set to 1000 copies. If problems of convergence are encountered with this method, Cheung's modified OLS method will also be attempted [7]; in addition, consideration will be given to using zero-inflated Poisson regression methods As stated above (section 7.2), if the convergence problems are identified as being caused by low or zero incidence of the outcome measure, as a last resort consideration will be given to combining and/or excluding proximate clusters with similar geographical and demographic properties within the same study arm.

7.6. Reporting conventions

7.6.1. *Descriptive statistics*

Variables will be checked for the presence of outliers, using tabulation and box plots. Continuous variables with an approximately normal distribution will be summarised by their mean, standard deviation and skewed continuous variables by their median and the interquartile range (25th percentile to 75th percentile). Parasite densities will be log-transformed and expressed as the geometric mean (95% CI). Categorical variables will be summarised by their frequency and percentage.

Means, standard deviations and any other statistics other than quartiles will be reported to one decimal place greater than the original unit of measure. Quartiles, such as median, or minimum and maximum will use the same number of decimal places as the original data. Estimated parameters, not on the same scale as raw observations (e.g. regression coefficients) will be reported to three significant figures.

7.6.2. *Measures of associations and P-value reporting*

Analyses will be conducted at either the 5% or 2.5% significance level, as appropriate, allowing for multiple testing of two intervention arms compared to the control. Estimates and their 95% confidence intervals (CI) will be produced using SAS 9.3 or v 9.4 (version may change) or SPSS v 22 or Stata v13 or v14. We will also report p-values. P-values ≥0.01 will be reported to four decimal places in the analysis; p-values less than 0.0001 will be reported as '<0.0001', as per The Lancet's convention.

8. Participant disposition and Flow chart

A flow chart will be drawn up showing the number clusters allocated to each study arm per site and the number of women screened, enrolled, and followed-up in each study arm, and the number contributing to the primary analysis and per-protocol. The number screened and not enrolled and the

reasons for non-enrolment will be reported, as well as the number and reasons of women who were lost for follow up, or who were withdrawn from study for safety reason or because of death.

9. Baseline data summaries

9.1. Demographic, clinical and laboratory measures

All baseline characteristics will be summarised by intervention group and overall. No inference testing will be conducted on the baseline variables, but marked differences (e.g. >10% relative difference) will be noted and taken into account in the post-hoc multiple regression analyses.

9.2. Measures of Social Economic Status (SES) Asset index.

The educational, income and socio-economic status parameters will be summarised in table form. To develop a single measure of SES index Principal Component Analysis (PCA) will be used to generate scores for ranking. PCA is a multivariate data analysis technique and it will reduce the dimension of this pool of variables to a smaller set of principal components capturing as much information (variability) from the data as possible. The summary SES index will be added to the baseline table.

10. Efficacy analyses

10.1. Measure of associations

10.1.1. *Binary outcomes*

For binary endpoints, the following will be calculated:

10.1.1.1. Unadjusted

1. Crude (unadjusted) prevalence data (numerator, denominator, % per arm)
2. Crude risk ratio (RR) (95% CI)
3. P-value for the crude RR

10.1.1.2. Adjusted

4. Adjusted RR (95% CI)
5. P-value for adjusted RR

We will also report the RR values as relative risk reduction (RRR), which will be calculated as (RRR=100%x[1-RR]) and can be expressed as a percentage.

10.1.2. *Continuous outcomes*

Differences in continuous endpoints will be assessed by linear regression analysis. For continuous endpoints, the following will be calculated:

10.1.2.1. Unadjusted

1. Crude mean and SD per arm
2. Crude mean difference (95% CI)

3. P-value for crude mean difference

10.1.2.2. Adjusted

4. Adjusted mean difference (95% CI)
5. P-value for adjusted mean difference

10.2. Forest plots of efficacy parameters

Results will be presented using forest plots for dichotomous and continuous variables. The graphics component will represent primary measure of association, i.e. the crude and adjusted Relative Risk (Reduction) and the adjusted and crude mean difference. In addition, columns with number of events and women per group, and Risk Difference (RD) (dichotomous variables) and the number of women, mean (SD) per group, and crude mean difference (95%) (Continuous variables) will be added. Forests plots will show results by site (Timika and Sumba), and overall (summary estimate stratified by site).

10.3. Primary efficacy endpoint analysis

10.3.1. *Crude and adjusted effect estimates*

The primary analyses will be ITT using the full analytical population. The primary measures of association are the risk ratio (RR) (95% CI) between the two groups obtained using the generalized linear regression models with binomial distribution and log link function.

The primary efficacy endpoint will be any malarial infection at delivery as defined in outcomes.

Both the crude (unadjusted) risk ratio (RR) and the adjusted RR will be computed using the generalised linear regression model. In the first model the response variable is the primary endpoint variable (yes/no) and the independent variable is treatment group. In the second model, additional independent variables will be included to adjust for potential confounding (overall and stratified by gravidity). The independent variables for adjustment are given in Section 10.3.3. The cluster variables will be included in all models.

10.3.2. *Adjustment for baseline independent variables in the multiple regression models*

The aim of the modelling is to obtain a valid estimate of an exposure-disease relationship i.e. a valid measure of the treatment effect of ISTp-DHP or IPTp-DHP relative to the control arm, adjusted for confounding. We will use the same independent co-variates in each multivariate model to allow for consistency across the models. The variables will be categorised into groups as indicated below (section 10.3.3).

10.3.3. *Variable specification*

Variables that will be included will include variables that are likely to be prognostic for the primary outcome but are not in the causal pathway, as predefined on the basis of the literature, and variables that are possibly prognostic for the primary outcome. All analyses will include the variable for ANC as the cluster variable.

The following variables will be considered a priori:

1. Gravidity (G1/2, G3+)
2. Site (Sumba, Timika)
3. Season during pregnancy (defined by rainfall in 6 months prior to delivery)
4. ITN use during pregnancy (if < 90%, otherwise there will be insufficient variation)
5. Malaria status at enrolment (pos/neg by PCR/LAMP or microscopy if PCR/LAMP not available)
6. Social Economic Status (tertile)
7. Corrected gestational age at booking (<=median,>median)

10.4. Secondary efficacy analysis of primary outcomes

10.4.1. *Subgroup analysis of the primary outcome*

We will include an interaction between treatment group and the factors in Section 10.3.3, page 22 in separate models to assess to what extent the effect of the intervention on the primary endpoints is influenced by these variables. We will also include number of intervention courses/visit received.as tertiles or another definition, e.g. <=2, 3, >=4 visits, subject to the observed distribution of visits. Because the study was not designed to have sufficient power for tests for interaction terms in these subgroup analysis, we will interpret the results cautiously. Results will be presented as forest plots.

10.4.2. *Sensitivity analysis*

10.4.2.1. Multiple Imputation for handling missing data in potential confounders

The results of the estimate (95% CI) obtained from multiple imputation for the missing covariate data statistical models will be compared with the complete-case (i.e. participants with missing covariate data are excluded) estimate (95% CI). The primary analysis reported will be the complete-case estimate, irrespective of whether the MI and complete-case estimates differ. Nonetheless the differences will be explicitly explained.

10.4.2.2. Corrected birth weights

The results of the statistical models using uncorrected birthweight will be compared with the initial results using corrected birthweights in a sensitivity analyses. Three different sensitivity analyses will be conducted: 1 using birthweight collected within 24 hours of birth (these are all uncorrected by definition), and one using all birthweight collected within 1 week, but without correction, and one using uncorrected birthweights collected within 1 week, but using timing of measurement as co-variate. In an event that there are differences between these results (e.g. >10% relative difference in effect estimate [e.g. RR 1.4 vs RR 1.6]) the results without correction will be taken as the final results. If the difference is <10% the corrected birthweight will be used (as this results in a bigger sample and minimizes the potential for overestimation of the frequency of small for gestational age). Any differences will be explicitly explained.

10.4.3. *Effect stratified by gravidity*

The primary efficacy outcomes will also be analysed stratified by gravidity (G1/2 vs G3+).

10.5. Secondary efficacy outcomes

10.5.1. *Outcomes at delivery*

The secondary efficacy outcomes outlined in Section outcomes will be analysed using similar crude and adjusted analysis. For the modelling approaches, the same independent variables as identified in the models for the two primary endpoints will be used for adjustment. Results will be expressed identical to the methods described above for the primary outcomes.

10.5.2. *Count data outcomes*

For the secondary efficacy outcomes that are count of episodes during follow up, these outcomes will be analysed using Poisson regression with the time of follow up as an offset. The incidence rate ratio for the treatment group effect will be estimated and its 95% CI presented.

In an event of over dispersion, then the Negative binomial regression model will be fitted to the data instead of the Poisson regression model. In an event where the number of episodes is very small and there are lots of zero episodes then a log binomial model will be fitted to the data where the dependent variable will be defined as (0=no episodes, 1=one or more episodes). A zero-inflated Poisson regression model will also be fitted to the data in an event of a lot of zero adverse events. All these models will be compared using the Akaike Information Criteria (AIC). A model with a smaller AIC will be considered as the final model under these conditions.

Both crude and adjusted analyses will be conducted similar to the primary endpoints. Variables considered for the full models will be the same as those for the primary endpoints.

10.5.3. *Continuous outcomes*

Similar considerations will be used for the assessment of continuous variables results expressed as mean differences (95% CIs) calculated by multiple linear regression models with independent variables as treatment group.

11. Safety analysis

For each safety outcome, the number of events and incidence of SAEs will be tabulated by system organ class, preferred term and by severity and causal relationship with the study drugs and compared between the three groups (IPTp-DHP versus ISTp or SSTp) using the appropriate count data regression model to estimate the treatment effect and its 95% confidence interval (CI) computed similar to the secondary efficacy outcomes.

Safety endpoint in the ISTP and SSTp arms will be reported by DHP exposure and non-exposure to DHP.

11.1. Further Adverse Events analysis.

All adverse events will be categorised as serious or non-serious. The total number of adverse events will be a count outcome and will be analysed using similar methods for count data as above. The independent variables in these analyses will be serious (yes/no) and treatment category, either: (1 =

IPTp, 2= ISTp, 3= SSTp- arm and tested positive and received the drug, 4= ISTp; 5 =SSTp arm and tested negative and never received the drug).

12. Other analysis

12.1. Number of intervention visits

Because the study was designed to allow variation between the number of scheduled visits as a function of the gestational age at enrolment (4+ scheduled follow-up visits for women who were enrolled early in pregnancy and 3 for women enrolled later in pregnancy), we will explore the difference in treatment effect on the primary endpoint between 3 and 4+ scheduled visits among the per protocol population. This will be done by including interactions between the number of scheduled visits (3 vs 4+) and treatment group so that an estimate of the comparison between the two treatment groups is estimated for each of these two strata.

12.2. Compliance with study drug

For definitions of treatment and regimen compliance measures see Section 3.7, Definitions for other variables, page 14. Further exploratory analysis will be conducted of the distribution and impact of regimen compliance on the primary endpoints. This will be done by including interactions between regimen compliance and treatment group so that an estimate of the comparison between the two treatment groups is estimated at each level of compliance. This will be done using quintiles of the regimen compliance variable to explore the shape of the relationship, as well as a continuous variable (0 to 100%). This analysis will exclude women who delivered prior to their last scheduled pre-natal visit. We will look at determinants of compliance, including whether dose intolerance is a predictor of subsequent compliance.

12.3. Treatment response

The percent (%) of women who were parasitaemic at each visit and are still parasitaemic at the next visits (defined as within 63 days inclusive [i.e. 9 weeks, the time typically used in extended in-vivo tests) will be compared between women in IPTp-DHP and ISTp arm using survival analysis and the hazard ratio (95% CI CI) reported for 28 (+/- 3 days) (about 1 visit later), 42 days (+/- 3 days) and 63 days (=/- 3 days; i.e. about 2 months). Only fully treatment adherent women of IPTp arm will be included in this analysis (for definition, see Section 3.7, Definitions for other variables, section 3.8 page 14).

13. References

1. Noel-Weiss J, Courant G, Woodend AK. Physiological weight loss in the breastfed neonate: a systematic review. Open medicine : a peer-reviewed, independent, open-access journal **2008**; 2(4): e99-e110.
2. Flaherman VJ, Kuzniewicz MW, Li S, Walsh E, McCulloch CE, Newman TB. First-day weight loss predicts eventual weight nadir for breastfeeding newborns. Arch Dis Child Fetal Neonatal Ed **2013**; 98(6): F488-92.
3. Greenwood BM, Greenwood AM, Snow RW, Byass P, Bennett S, Hatib-N'Jie AB. The effects of malaria chemoprophylaxis given by traditional birth attendants on

the course and outcome of pregnancy. Trans R Soc Trop Med Hyg **1989**; 83(5): 589-94.
4. D'Alessandro U, Langerock P, Bennett S, Francis N, Cham K, Greenwood BM. The impact of a national impregnated bed net programme on the outcome of pregnancy in primigravidae in The Gambia. Trans R Soc Trop Med Hyg **1996**; 90(5): 487-92.
5. Schmiegelow C, Scheike T, Oesterholt M, et al. Development of a fetal weight chart using serial trans-abdominal ultrasound in an East African population: a longitudinal observational study. PLoS One **2012**; 7(9): e44773.
6. Brabin B. Fetal anaemia in malarious areas: its causes and significance. Ann Trop Paediatr **1992**; 12(3): 303-10.
7. Cheung YB. A modified least-squares regression approach to the estimation of risk difference. Am J Epidemiol **2007**; 166(11): 1337-44.

14. Tables and Figures

14.1. List of tables

**Section 1**	**Patient disposition**
Table 1.1	Patient disposition by treatment
Table 1.2	Patient disposition by gravidity group and study arm
**Section 2**	**Baseline information**
Table 2.1	Demographic characteristics of patients
Table 2.2	Bednet use at enrolment and at delivery, and IRS and place from where bednet received
Table 2.3	Obstetric History
Table 2.4	Information copied from ANC card
Table 2.6	Medical history/ illness symptoms
Table 2.7	Prior and concomitant medication
Table 2.8	Physical examination
Table 2.9	RDT result and drug intake
Table 2.10	Social economic status
**Section 3**	**Medication**
Table 3.1	Prior medication
Table 3.2	Concomitant medication
**Section 4**	**Follow-up information**
Table 4.1	Antenatal visit: physical examination
Table 4.2	Morbidity: history of fever last 24 hours, documented fever during visit
Table 4.3	Maternal postnatal visit (scheduled): ITN use, physical examination
Table 4.3	Infant visit (scheduled): visit number, ITN use, morbidity, physical exam
**Section 5**	**Delivery information**
Table 5.1	Delivery visit: ITN use, morbidity, physical exam, place of delivery, mode of delivery, Person attending delivery
Table 5.2	Newborn: Outcome of delivery, baby measurements, Physical abnormalities
Table 5.3	Ballard score: neuromuscular score, physical maturity score, total score, gestational age
Table 5.3	Delivery malaria smear test (maternal)

Table 5.4	Baby surface examination
Table 5.5	Neonatal/infant death: gestational age, gender study arm, illness prior to death
**Section 6**	**RDT and Blood samples**
Table 6.1	RDT result by visit type (booking, scheduled visit)
Table 6.2	Haemoglobin result by visit type
Table 6.3	Blood smear result by antenatal visit
Table 6.4	PCR/LAMP result by antenatal visit
**Section 7**	**Delivery RDT and blood samples**
Table 7.1	Maternal and placental RDT results
Table 7.2	Maternal Hb at delivery and time of sampling; cord blood Hb
Table 7.3	Maternal, Placental (incision smear) and Cord blood smear results
Table 7.4	Maternal, placental, cord blood PCR/LAMP
Table 7.5	Placental histopathology results
**Section 8**	**Adverse events (note: all adverse events tables will be done for overall, by study arm and by exposure and non-exposure to study drug)**
Table 8.1.1	Summary of adverse event: Mothers
Table 8.1.2	Summary of adverse event by system organ class and preferred term: Mothers
Table8.1.3	Summary of serious adverse event by system organ class and preferred term: Mothers
Table 8.1.4	Summary of adverse event by severity, system organ class and preferred term: Mothers
Table 8.1.5	Summary of adverse event by causality, system organ class and preferred term: Mothers
Table 8.2.1	Summary of adverse event: infants
Table 8.2.2	Summary of adverse event by system organ class and preferred term: infants
Table 8.2.3	Summary of serious adverse event by system organ class and preferred term: infants
Table 8.2.4	Summary of adverse event by severity, system organ class and preferred term: infants
Table 8.2.5	Summary of adverse event by causality, system organ class and preferred term: infants
**Section 9**	**Efficacy**
Table 9.1	Primary outcome analysis
Table 9.2	Secondary efficacy outcomes
Table 9.3	Secondary safety outcomes
Table9.4	Malaria infection endpoint definitions
Table 9.5	Morbidity endpoint definitions
**Section 10**	**Covariate adjusted analysis**
Table 10.1	Primary outcome analysis
**Section 11**	**Subgroup analysis**
Table 11.1	Subgroup analysis of primary outcome

14.2. Patient disposition (Flow chart)

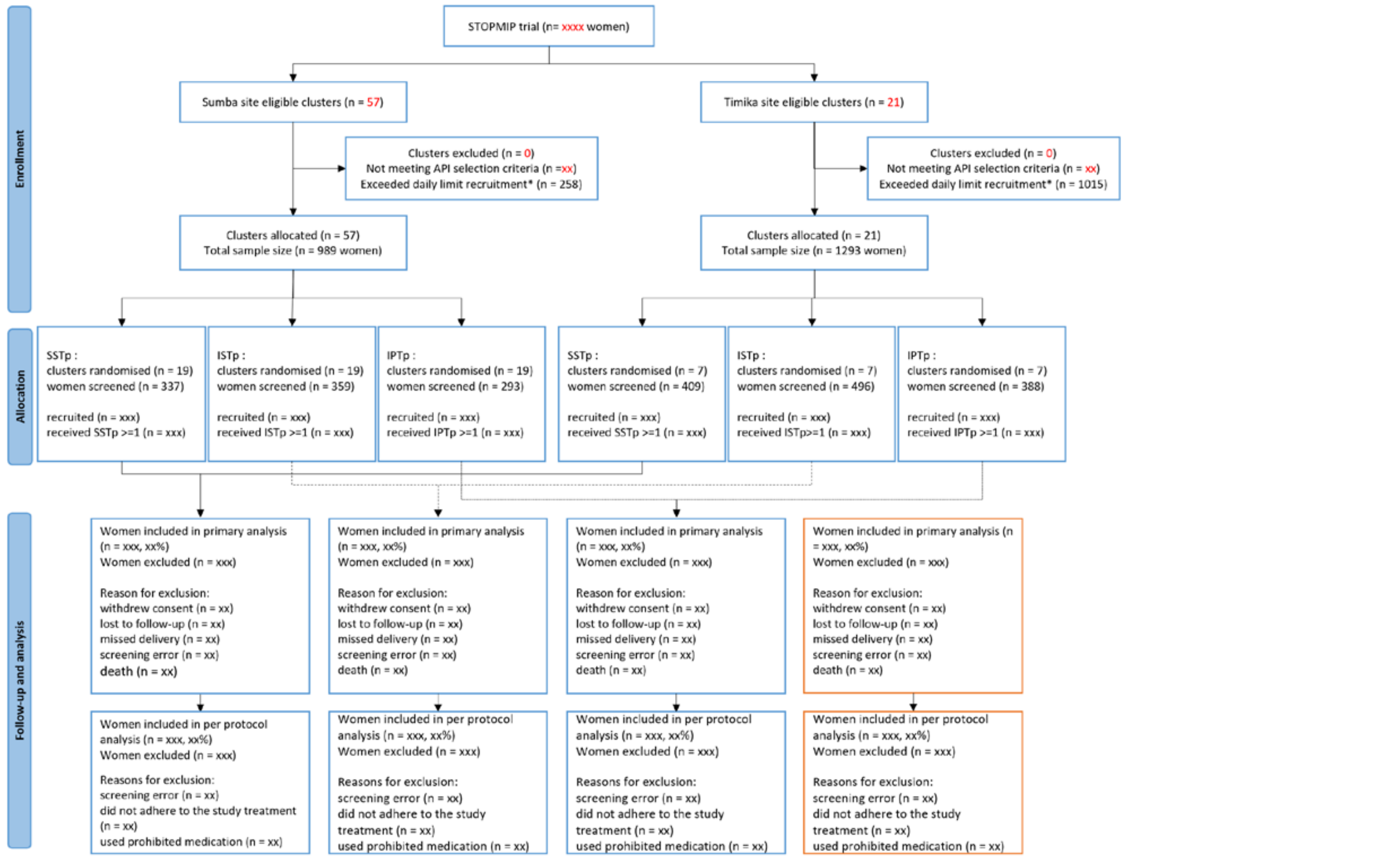

* The number of recruitment per day per cluster was limited to keep the follow-up numbers manageable in subsequent visits and to spread out reaching the target sample size over the target period.

15. Versioning manuscript

15.1. Word document

The manuscript drafts will be called SMI_manuscript-[yy-mm-dd].Docx. SMI stands for STOPMIP Indonesia. Versioning is done by use of date in the file name (e.g. 'SMI_manuscript_2015-04-13.docx'). If colleagues comment on a draft, they add their initials at the end of the version they comment on; e.g. 'SMI_manuscript_2015-04-13_RA.docx').

15.2. Endnote

The endnote file will be called 'SMI-endnote.enl', and will have no versioning because endnote embeds the library in the word file.

16. Appendices

16.1. Appendix 1: CONSORT 2010 checklist of information to include when reporting a cluster randomised trial

Section/Topic	Item No	Standard Checklist item	Extension for cluster designs	Page No *
**Title and abstract**				
	1a	Identification as a randomised trial in the title	Identification as a cluster randomised trial in the title	
	1b	Structured summary of trial design, methods, results, and conclusions (for specific guidance see CONSORT for abstracts)[i, ii]	See table 2	
**Introduction**				
**Background and objectives**	2a	Scientific background and explanation of rationale	Rationale for using a cluster design	
	2b	Specific objectives or hypotheses	Whether objectives pertain to the the cluster level, the individual participant level or both	
**Methods**				
**Trial design**	3a	Description of trial design (such as parallel, factorial) including allocation ratio	Definition of cluster and description of how the design features apply to the clusters	
	3b	Important changes to methods after trial commencement (such as eligibility criteria), with reasons		
**Participants**	4a	Eligibility criteria for participants	Eligibility criteria for clusters	
	4b	Settings and locations where the data were collected		
**Interventions**	5	The interventions for each group with sufficient details to allow replication, including how and when	Whether interventions pertain to the cluster level, the individual participant level or both	

		they were actually administered	
**Outcomes**	6a	Completely defined pre-specified primary and secondary outcome measures, including how and when they were assessed	Whether outcome measures pertain to the cluster level, the individual participant level or both
	6b	Any changes to trial outcomes after the trial commenced, with reasons	
**Sample size**	7a	How sample size was determined	Method of calculation, number of clusters(s) (and whether equal or unequal cluster sizes are assumed), cluster size, a coefficient of intracluster correlation (ICC or *k*), and an indication of its uncertainty
	7b	When applicable, explanation of any interim analyses and stopping guidelines	
**Randomisation:**			
**Sequence generation**	8a	Method used to generate the random allocation sequence	
	8b	Type of randomisation; details of any restriction (such as blocking and block size)	Details of stratification or matching if used
**Allocation concealment mechanism**	9	Mechanism used to implement the random allocation sequence (such as sequentially numbered containers), describing any steps taken to conceal the sequence until interventions were assigned	Specification that allocation was based on clusters rather than individuals and whether allocation concealment (if any) was at the cluster level, the individual participant level or both
**Implementation**	10	Who generated the random allocation sequence, who enrolled participants, and who assigned participants to interventions	Replace by 10a, 10b and 10c

	10a		Who generated the random allocation sequence, who enrolled clusters, and who assigned clusters to interventions
	10b		Mechanism by which individual participants were included in clusters for the purposes of the trial (such as complete enumeration, random sampling)
	10c		From whom consent was sought (representatives of the cluster, or individual cluster members, or both), and whether consent was sought before or after randomisation
**Blinding**	11a	If done, who was blinded after assignment to interventions (for example, participants, care providers, those assessing outcomes) and how	
	11b	If relevant, description of the similarity of interventions	
**Statistical methods**	12a	Statistical methods used to compare groups for primary and secondary outcomes	How clustering was taken into account
	12b	Methods for additional analyses, such as subgroup analyses and adjusted analyses	
**Results**			
**Participant flow (a diagram is strongly recommended)**	13a	For each group, the numbers of participants who were randomly assigned, received intended treatment, and were analysed for the primary outcome	For each group, the numbers of clusters that were randomly assigned, received intended treatment, and were analysed for the primary outcome

	13b	For each group, losses and exclusions after randomisation, together with reasons	For each group, losses and exclusions for both clusters and individual cluster members
**Recruitment**	14a	Dates defining the periods of recruitment and follow-up	
	14b	Why the trial ended or was stopped	
**Baseline data**	15	A table showing baseline demographic and clinical characteristics for each group	Baseline characteristics for the individual and cluster levels as applicable for each group
**Numbers analysed**	16	For each group, number of participants (denominator) included in each analysis and whether the analysis was by original assigned groups	For each group, number of clusters included in each analysis
**Outcomes and estimation**	17a	For each primary and secondary outcome, results for each group, and the estimated effect size and its precision (such as 95% confidence interval)	Results at the individual or cluster level as applicable and a coefficient of intracluster correlation (ICC or k) for each primary outcome
	17b	For binary outcomes, presentation of both absolute and relative effect sizes is recommended	
**Ancillary analyses**	18	Results of any other analyses performed, including subgroup analyses and adjusted analyses, distinguishing pre-specified from exploratory	
**Harms**	19	All important harms or unintended effects in each group (for specific guidance see CONSORT for harms[iii])	
**Discussion**			
**Limitations**	20	Trial limitations, addressing sources of potential bias,	

		imprecision, and, if relevant, multiplicity of analyses	
**Generalisability**	21	Generalisability (external validity, applicability) of the trial findings	Generalisability to clusters and/or individual participants (as relevant)
**Interpretation**	22	Interpretation consistent with results, balancing benefits and harms, and considering other relevant evidence	
**Other information**			
**Registration**	23	Registration number and name of trial registry	
**Protocol**	24	Where the full trial protocol can be accessed, if available	
**Funding**	25	Sources of funding and other support (such as supply of drugs), role of funders	

---

[i] Hopewell S, Clarke M, Moher D, Wager E, Middleton P, Altman DG, et al. CONSORT for reporting randomised trials in journal and conference abstracts. *Lancet* 2008, 371:281-283

[ii] Hopewell S, Clarke M, Moher D, Wager E, Middleton P, Altman DG at al (2008) CONSORT for reporting randomized controlled trials in journal and conference abstracts: explanation and elaboration. *PLoS Med* 5(1): e20

[iii] Ioannidis JP, Evans SJ, Gotzsche PC, O'Neill RT, Altman DG, Schulz K, Moher D. Better reporting of harms in randomized trials: an extension of the CONSORT statement. *Ann Intern Med* 2004; 141(10):781-788.
